# Altered Mitochondrial Respiration Is Associated With Loss of Nuclear‐Encoded OXPHOS Genes in Parasitic Broomrapes

**DOI:** 10.1002/ece3.71737

**Published:** 2025-07-06

**Authors:** Liming Cai, Robert K. Jansen, Justin C. Havird

**Affiliations:** ^1^ Department of Integrative Biology The University of Texas at Austin Austin Texas USA

**Keywords:** cytonuclear conflict, high‐resolution respirometry, O2K, relaxed selection

## Abstract

Parasitic plants, characterized by their dependency on host organisms for nutrients, have displayed far‐reaching alterations in physiology and genetics. While significant gene losses and relaxed selection have been documented in the nuclear and plastid genomes, how parasitism impacts the molecular evolution and function of mitochondria has remained controversial. One of the main culprits hindering our understanding in this area is the lack of knowledge on nuclear‐encoded mitochondrial‐targeted genes (N‐mt), which encode most mitochondrial oxidative phosphorylation (OXPHOS) proteins. By conducting a comprehensive survey of N‐mt genes across angiosperms, we demonstrated significant gene losses and occasional horizontal transfers associated with relaxed selection unique to holoparasitic Orobanchaceae. These putative losses and transfers have the potential to affect mitochondrial function directly and cause cytonuclear incompatibility because of breakdown between co‐evolved protein complexes from mitochondrial and nuclear genomes. Our physiological assessments using high‐resolution respirometry revealed that despite genetic alterations, holoparasitic Orobanchaceae maintained OXPHOS capacity but relied more on the fully nuclear‐encoded succinate dehydrogenase (complex II). Our results document the first example of biased loss of nuclear‐encoded OXPHOS genes without accompanying mitochondrial‐encoded gene loss in parasitic plants, expanding on previous studies and elucidating the mechanisms underlying the preservation of OXPHOS function despite genomic reduction.

## Graphical abstract

The holoparasitic tribe Orobancheae demonstrated significant gene losses in their nuclear‐encoded mitochondrial OXPHOS genes. Despite these losses, high resolution respirometry revealed robust mitochondrial respiration in Orobancheae by shifting activities to fully nuclear encoded alternative complexes.

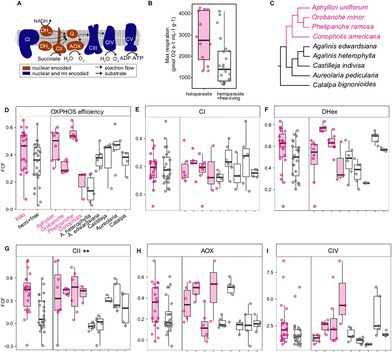

## 1 Introduction

Mitochondria fuel energy metabolism for nearly all eukaryotes, including plants. Chemical energy is converted to ATP via a series of orchestrated oxidative phosphorylation enzymes (OXPHOS) and accessory proteins that transport electrons, pump protons, and ultimately convert ADP to ATP. In most eukaryotes, this process is carried out by five core protein complexes I–V (CI‐CV) on the inner mitochondrial membrane, including NADH dehydrogenase (CI), succinate dehydrogenase (CII), cytochrome c reductase (CIII), cytochrome c oxidase (CIV), and ATP synthase (CV). These enzymes vary greatly in size and genetic makeup, with many containing chimeric assemblies of mitochondrial and nuclear‐encoded proteins. Consequently, these chimeric OXPHOS complexes are not only hotspots of concerted evolution between the nuclear and mitochondrial genomes, but also vulnerable targets of genetic incompatibility (i.e., cytonuclear conflict) (Havird, Forsythe, et al. 2019; Sloan et al. 2018, 2017). Such incompatibilities come with strong fitness costs and can play a major role in creating reproductive barriers in yeasts, fishes, and angiosperms (Lee et al. 2008; Luo et al. 2013; Moran et al. 2024). A potentially more widespread and less well‐documented consequence of cytonuclear conflict is compromised or altered mitochondrial respiration (Havird, Noe, et al. 2019; Weaver et al. 2020). In plants, the electron transfer chain is further branched with alternative electron entry and exit via the alternative NAD(P)H dehydrogenases (NDs) and alternative oxidase (AOX). These redundant complexes can alter OXPHOS function in response to environmental stressors and even compensate for OXPHOS dysfunction caused by cytonuclear conflict (Siedow and Girvin 1980; Sweetman et al. 2019; Vanlerberghe 2013). For example, the extremely high mitochondrial substitution rates of *Silene* (Caryophyllaceae) have led to lower efficiencies in chimeric OXPHOS enzymes such as CI and CIV, along with increased reliance on alternative NDs and AOX for mitochondrial respiration (Weaver et al. 2020).

Cytonuclear conflict may stem from the proliferation of incompatible mitochondrial mutations that can be attributed to changes in population dynamics, DNA repair machinery, mutational processes, life history strategies, or a combination of these phenomena (Havird et al. 2017; Zwonitzer et al. 2024). The evolution of parasitism, in particular, may trigger abrupt changes in energy metabolism that lead to shifts in how chimeric versus alternative OXPHOS enzymes are used. The most well‐characterized and enthralling examples come from parasitic microbial eukaryotes—A wide range of mitochondrial functions have been documented from fully aerobic organelles to mitosomes stripped of OXPHOS and restricted to Fe‐S biosynthesis (Hjort et al. 2010; John et al. 2019; Mathur et al. 2021; Santos et al. 2018). In at least one case the obligate symbiont *Monocercomonoides* has lost its mitochondria completely (Karnkowska et al. 2016). In photosynthetic organisms, comparable mitochondrial alterations have been documented especially among parasitic algae (Hancock et al. 2010; Reyes‐Prieto et al. 2002; Smith et al. 2010; Smith and Lee 2014). Among flowering plants, the most well‐studied example comes from the mistletoe *Viscum* (Santalaceae). Their CI genes have been lost completely along with highly diverged sequences for the other mitochondrial genes, which is indicative of relaxed selection on mitochondrial DNA (Petersen et al. 2015; Senkler et al. 2018; Skippington et al. 2017, 2015). These changes have diminished mitochondrial respiration function fivefold, leading to an increased dependence on alternative pathways via NDs and AOX (Maclean et al. 2018).

The exciting discoveries in mistletoes have sparked a rush of investigations on parasitic and mycoheterotrophic plants. Yet none showed comparable levels of gene loss (Zervas et al. 2019). Instead, more subtle genomic changes have been found in mostly non‐coding regions, including structural rearrangements, horizontal gene transfers, repeat expansion, nucleotide composition bias, and substitution rate elevation (Fan et al. 2016; Petersen et al. 2019; Sanchez‐Puerta et al. 2017; Shtratnikova et al. 2020; Xi et al. 2013; Yu et al. 2023). The most substantial alterations involve the *Lophophytum* (Balanophoraceae) mitogenome, where more than 80% of the mitochondrial‐encoded protein‐coding genes are replaced by horizontally transferred genes (Gatica‐Soria et al. 2024). Even in this extreme case, mitochondrial respiration appears to experience no disruption (Gatica‐Soria et al. 2024). Thus, many have concluded that the association between parasitism and functional genomic degradation is restricted to the plastid and nuclear genomes and not mitochondria (Fan et al. 2016), with mistletoes being an exception rather than the rule.

However, previous studies neglected the majority (77%) of OXPHOS proteins encoded by nuclear genes along with an even larger repertoire of mitochondrial assembly factors and genes involved in DNA replication, repair, and recombination (RRR) that may impact the overall integrity of the mitochondrial genome and function (Sloan 2015; Smith and Keeling 2015). Furthermore, functional verifications on OXPHOS respiration are outstandingly lacking except for *Viscum* and very recently, in Balanophoraceae (Gatica‐Soria et al. 2024; McLean et al. 2018). The impact of parasitism on mitochondrial respiration cannot be fully understood without a comprehensive survey of the content of OXPHOS genes in both the mitochondrion and nucleus, ideally combined with thorough functional assessments.

To remedy this knowledge gap, we used comparative genomics and high‐resolution respirometry to study the link between nuclear‐encoded mitochondrial‐targeted gene (N‐mt) content, mitochondrial respiration, and parasitism in the flowering plant family Orobanchaceae (the broomrapes). These ca. 2000 species span the entire spectrum of plant parasitism from free‐living species to holoparasites that rely completely on hosts for water and nutrients. In particular, the transition to holoparasitism has evolved three times independently (McNeal et al. 2013), providing a unique comparative framework to investigate the link between life history strategy and organellar function. In contrast to the well‐characterized plastid genome degradation of Orobanchaceae (e.g., (Wicke et al. 2016)), mitochondria have received less attention. Mitochondrial genomes from approximately a dozen Orobanchaceae hemiparasites and holoparasites have a complete and canonical set of core genes with only slightly elevated substitution rates in some lineages (Fan et al. 2016; Zervas et al. 2019). Widespread horizontal gene transfer has been reported in mitochondria, but cases are restricted to non‐coding regions such as the mobile group I intron of the *cox1* gene (Fan et al. 2016). Yet, given their fundamental changes in energy metabolism, the overall trend of genomic degradation in parasites, together with their strongly host‐dependent reproductive strategy that may bottleneck the population (Cai 2023; Cai et al. 2021), we hypothesize relaxed selection in the nuclear‐encoded mitochondrial OXPHOS genes of parasitic Orobanchaceae. This may lead to changes as extreme as the entire loss of CI in *Viscum*, or more subtle signatures such as accelerated rates in N‐mt genes.

Here, we conducted a comprehensive survey of N‐mt genes across angiosperms, focusing on species in Orobanchaceae spanning parasitic life history strategies. We demonstrated frequent losses and significant relaxed selection of N‐mt genes unique to one holoparasitic tribe (Orobancheae sensu Fischer (Fischer 2004)) that are otherwise rare in flowering plants. These included both OXPHOS and mitochondrial RRR genes. To explore the functional impact of these gene losses, we applied high‐resolution respirometry to isolated mitochondria from representative species to generate fine‐scale mitochondrial respiration data at the protein level. We found that parasitic lineages with substantial N‐mt gene loss tend to have increased reliance on nuclear‐encoded CII, alternative NDs, and AOX, although CI and other chimeric OXPHOS complexes maintained normal function despite gene losses.

## 2 Materials and Methods

### 2.1 Comparative Survey of N‐Mt Genes

Homologs of N‐mt genes were identified in two steps. First, a broad survey of N‐mt genes across angiosperms was conducted using an orthogroup database from our previous study (Cai et al. 2021). This database contains 23,151 orthogroups from 28 angiosperm species inferred by the similarity‐based orthology clustering program OrthoFinder v2.2.7 (Emms and Kelly 2015) (Table S1). These species have high‐quality reference genomes spanning from early‐diverging *Amborella* to representative species in rosids and asterids. To retrieve N‐mt genes, N‐mt homologs of *Arabidopsis thaliana* reported by Meyer et al. (2019) and Zhang et al. (2016) were used as baits to identify the corresponding orthogroups. We included 61 nuclear‐encoded OXPHOS proteins and 26 organellar RRR proteins (18 dual‐targeted to mitochondria and plastids, plus 8 specific to mitochondria). To assess the general evolutionary trend of other N‐mt genes not involved in OXPHOS function, we selected a subset of 7 mitochondrial ribosomal proteins as controls based on Havird et al. (2017). To mitigate false negatives, we further verified all absences by two approaches: (1) We used an alternative similarity‐based search tool—the profile hidden Markov implemented in HMMER v3.3.2 (Mistry et al. 2013)—to identify any potential homologs in coding sequences with an e‐value threshold of 1e‐30. This helps mitigate false negatives stemming from BLAST algorithms. (2) To address potential annotation errors, we searched the entire genome sequences from these 28 reference taxa using BLASTN to confirm absence or pseudogenization. Whenever possible, multiple versions of genome assemblies and annotations from the same species or close relatives from the same genus were consulted (Table S1).

**FIGURE 1:**
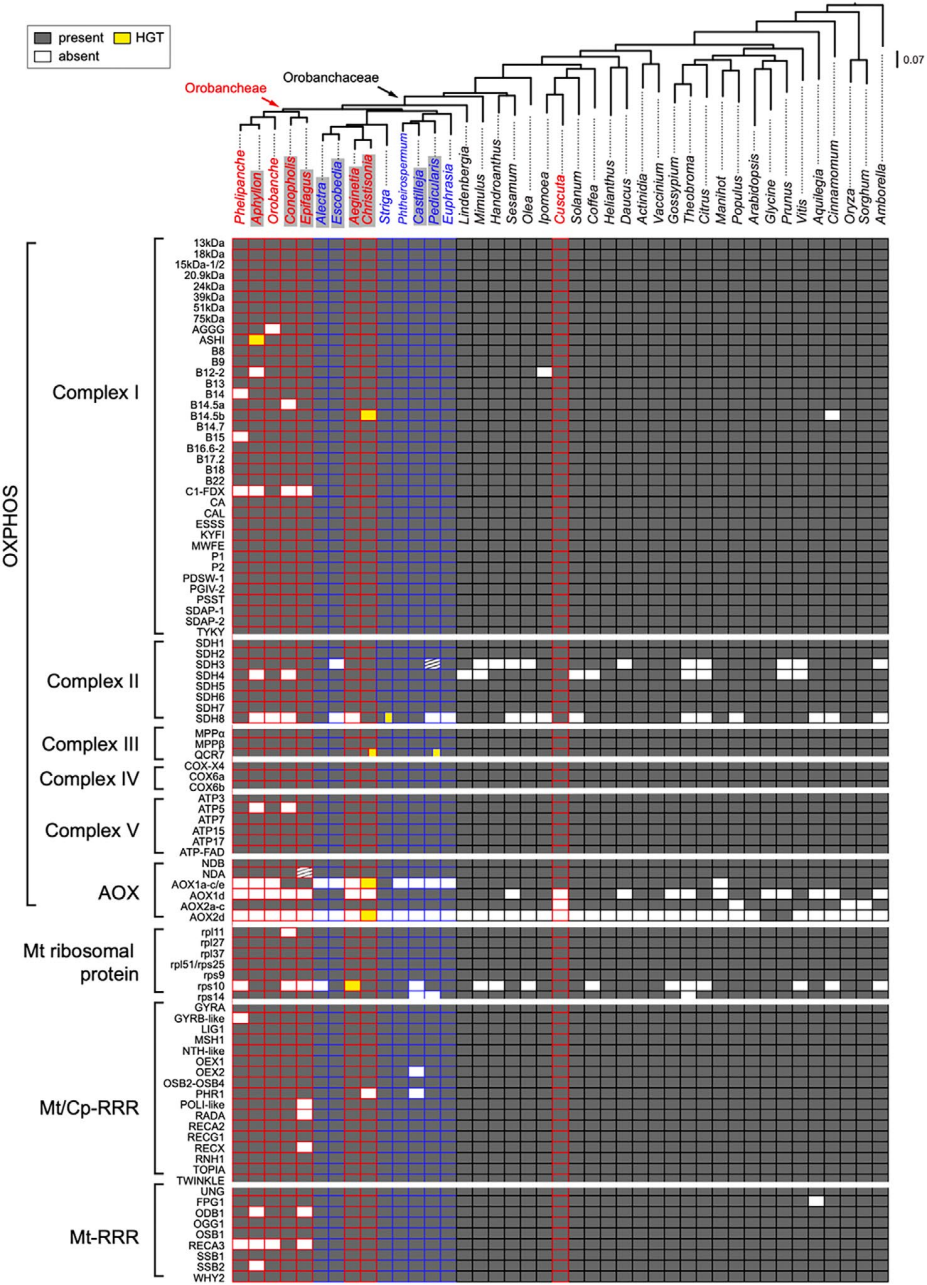
Presence and absence matrix of selected nuclear‐encoded mitochondrial (N‐mt) enzymes shows major losses in the holoparasitic tribe Orobancheae. The maximum likelihood phylogenetic tree of representative angiosperms is inferred from a concatenated DNA alignment of all N‐mt genes in IQ‐TREE. Holoparasitic lineages are highlighted in red and hemiparasitic lineages are highlighted in blue. Species with transcriptome data are marked with gray shading. Dark gray cells indicate complete or partial presence of the sequence; white cells indicate absence; white cells with horizontal stripes indicate pseudogenes with frameshifts or premature stop codons; yellow cells indicate putative horizontal gene transfer; hybrid yellow‐gray cells indicate coexistence of both native and horizontally transferred sequences.

Second, a focused search of N‐mt genes in Orobanchaceae was conducted by compiling a database using published genomes and transcriptomes from 15 species (Table S1). These included one free‐living species (*Lindenbergia philippensis*), seven hemiparasites, and seven species that represent two independent origins of holoparasitism (Figure 1). We then applied BLASTN and HMMER to identify candidate N‐mt genes as described previously. Due to the incomplete nature of transcriptomes, gene loss was determined conservatively, with any orthologous fragments counted as present (except when frameshifts or premature stop codons were confirmed manually; striped cells in Figure 1). When possible, sequence data from multiple individuals of the same species were used to verify putative gene losses in *Epifagus virginiana* (two RNA samples), *Aphyllon fasciculatum* (two RNA samples), and *Lindenbergia philippensis* (one genome and two RNA samples; Table S1).

Finally, candidate orthologous sequences from Orobanchaceae were combined with the angiosperm dataset. A phylogeny‐based approach was implemented to remove non‐orthologous sequences. To accomplish this, individual gene trees were inferred with DNA alignments, and the maximum inclusion algorithm described in Yang and Smith (2014) was applied. This algorithm iteratively cuts out the subtree with the highest number of taxa without taxon duplication. It also outputs a one‐to‐one ortholog dataset that can be directly used for species tree inference. Details on DNA alignment and phylogeny inference are described in the section ‘Species tree reconstruction’ below. All scripts used in this study are available on GitHub (https://github.com/lmcai/Orobanchaceae_O2K) and archived on Zenodo (10.5281/zenodo.15584982).

### 2.2 Species Tree Reconstruction

To infer a species tree for comparative analyses, we aligned the protein sequences of one‐to‐one orthologs using the E‐INS‐i algorithm implemented in MAFFT v7.453 (Katoh and Standley 2013). These protein alignments were then back translated to codons with pal2nal v14 (Suyama et al. 2006). Maximum Likelihood phylogenies were inferred for both individual genes and the concatenated codon alignments using IQ‐TREE v2.2.2.6 (Minh et al. 2020). A gene‐by‐gene partition was used for the concatenation analysis, and the best substitution model was determined by the built‐in ModelFinder program (Kalyaanamoorthy et al. 2017). Branch support was assessed by performing 1000 ultrafast bootstrap replicates (UFBP).

### 2.3 Verification of Missing N‐Mt Genes

In addition to applying alternative search algorithms and multiple assembly or annotation versions, we further examined whether data quality or sequence divergence may lead to observed patterns of missing genes. First, genome and transcriptome quality were assessed using BUSCO v5.7.0 based on the land plant (embryophyta_odb10) and the eukaryote (eukaryota_odb10) databases (Simão et al. 2015). Both databases were applied because the land plant BUSCO database is enriched in photosynthesis‐related genes that are prone to loss in parasitic plants (Cai et al. 2021). Therefore, the eukaryote database is more accurate than the land plant database for quality control in this case. Second, we examined the pairwise sequence divergence between *Arabidopsis* and other angiosperms to test whether missing genes may arise artificially from extreme sequence divergence. We applied the dist.dna function from the R package ape (Paradis and Schliep 2019) to calculate both the proportion of nucleotide difference (model = “raw”) as well as the sequence divergence under the F84 substitution model (model = “F84”). The concatenated DNA alignment of N‐mt genes for species tree inference was used for this calculation.

Finally, we used phylogenetic ANOVA to evaluate whether parasitic or holoparasitic Orobanchaceae demonstrated significantly higher proportions of missing BUSCOs or higher sequence divergence compared to free‐living angiosperms. The species tree inferred above and the phylANOVA function in the R package phytools (Revell 2012) were used. To further evaluate the significance of N‐mt gene loss in Orobanchaceae while accounting for data quality, we conducted a Fisher's exact test with *p*‐values corrected by the ratio of missing eukaryote BUSCOs.

### 2.4 Molecular Evolution

To prepare sequences for molecular evolution analyses, we predicted N‐terminal targeting peptides using the TargetP2.0 webserver (https://services.healthtech.dtu.dk/services/TargetP‐2.0/) (Almagro Armenteros et al. 2019) and removed these regions before downstream analyses. To test for relaxed selection on N‐mt genes in parasites, we applied the RELAX method from HyPhy v2.5.33 to both individual genes as well as concatenated sequences grouped by nine functional classes (Figure 1) (Kosakovsky Pond et al. 2020; Wertheim et al. 2015). RELAX compares the ω value (d_N_/d_S_) in the foreground branches to the background branches in a reference species tree. If the foreground branches are under relaxed selection, their ω values are expected to converge towards the neutral value 1 across all rate categories and vice versa for intensified selection (Wertheim et al. 2015). To apply this method, we subsampled the codon alignment to include a single sequence per species to facilitate the function‐based concatenation. Potential horizontal gene transfers nested outside Orobanchaceae were removed prior to downstream analyses (see Results below). When multiple copies were represented for a species, we chose the one showing minimal root‐to‐tip distance to facilitate concatenation because these copies showed the lowest sequence divergence to the outgroup and represent a conservative way to detect relaxed selection. The species tree inferred above was used as the reference. We then ran RELAX under 3 rate categories, the GTR substitution model, and constant synonymous substitution rates across sites. We tested two evolution models where either the holoparasitic tribe Orobancheae alone or Orobancheae plus the other holoparasitic lineage *Aeginetia* + *Christisonia* were selected as foreground test branches (Figure 1). The goodness of fit of these two models was evaluated using the likelihood ratio test.

### 2.5 Taxon Sampling for Respirometry Assessment

To explore the functional impact of OXPHOS gene loss, we assessed mitochondrial respirometry in representative species of Orobanchaceae. This experiment requires fresh tissue and access to a molecular lab immediately after collection. Moreover, the scarce distribution and ephemeral above‐ground phenology of many Orobanchaceae further restricted our sampling options. We therefore selected 50 individuals from nine species adjacent to our laboratory facilities in Austin, TX, and Cambridge, MA, during two field seasons from 2022 to 2023 (Table S2). All four holoparasites— *Aphyllon uniflorum* , *Orobanche minor* , *Phelipanche ramosa*, and *Conopholis americana—*are members of the tribe Orobancheae, which have experienced extensive OXPHOS gene loss (see Results). The other five hemiparasitic and free‐living species served as references where OXPHOS genes are under stronger purifying selection. These plants were carefully excavated from the soil, sometimes with their hosts to avoid stress. To account for variation in tissue types and developmental stages, we exclusively sampled floral tissues when possible. Due to the variation in phenology and the large quantity of required tissue (1 g per experiment), leaves were sampled for *Aureolaria pedicularia* (Table S2).

Besides the nine species presented in this study, respirometry assays were also performed on six hemiparasitic and free‐living species, including *Belladia trixago, Melampyrum lineare*, *Aureolaria flava* , *Triphysaria versicolor, Rehmannia glutinosa*, and *Rehmannia grandiflora*. However, experiments were inconclusive for these taxa, given that respiration rates did not significantly increase when the substrate ADP was added (see below). We attribute these negative results to organism‐ and tissue‐specific metabolic profiles that might inhibit the standard respirometry assay, rather than a real loss of mitochondrial function, and do not present respirometry data for these species here.

### 2.6 Mitochondrial Isolation and Respirometry

Wild plants were harvested and preserved on ice before being transferred to the lab. Respirometry assays followed a well‐established protocol in our lab that has been applied to a diverse array of plant and animal systems including *Silene*, crustaceans, nematodes, and mayflies (Havird, Noe, et al. 2019; Weaver et al. 2020). A detailed protocol is provided in Note S1. Briefly, 1 g of floral or leaf tissue was minced and ground in an ice‐cold mitochondrial isolation buffer following Havird, Shah, and Chicco (2019). Intact mitochondria were then isolated using differential centrifugation, and the protein content of mitochondrial isolates was quantified. Subsequently, we used the Oroboros O2K high‐resolution respirometry system (Innsbruck, Austria) with a substrate‐uncoupler inhibitor‐titration protocol to quantify seven different mitochondrial respiration states in each sample (Figure S1). We recorded the maximum respiration rate observed prior to the addition of any inhibitors, the maximum rate observed during the experiment, as well as several internally normalized statistics (see below). The calculated ADP‐driven respiration rate (in nmol O_2_ s^−1^ mL^−1^ g^−1^) was achieved after the addition of succinate. The maximum respiration rate was normalized by the protein content, and we thus excluded three species ( *Aphyllon uniflorum* , *Conopholis americana* , and *Aureolaria pedicularia* ) from maximum respiration measurements because they lacked protein quantification due to the inaccessibility of lab equipment in the field (Table S2).

### 2.7 Statistical Analyses

To examine the relative contributions of OXPHOS enzymes, six flux control factors (FCFs) were calculated (Table S3). Flux control factors are internally normalized by the ratio of respiration rate before and after the addition of electron donors or inhibitors to specific OXPHOS components (Pesta and Gnaiger 2012). Two of the FCFs are related to CI function: OXPHOS efficiency (analogous to the respiratory control ratio (Jacoby et al. 2015)), and a CI FCF induced by adding the CI inhibitor rotenone. We also calculated FCFs that described CII, CIV, and the nuclear‐encoded external NAD(P)H dehydrogenases (DH_ex_) and AOX. Linear mixed‐effects (LME) models were implemented using the lme function from the R package nlme to test for statistical significance in differences in respiratory flux control (Pinheiro et al. 2024). Species were classified into two groups: with and without N‐mt gene loss (i.e., free‐living + hemiparasitic vs. holoparasitic). To control for multiple observations within a species, we included species as a fixed factor and accession as a random factor. We log‐transformed the CIV FCF to meet the assumption of normality. We also tested for correlations among FCFs and maximum respiration rate by fitting LME models.

## 3 Results

### 3.1 Conserved N‐Mt Gene Content Across Angiosperms

The availability of high‐quality genomes in reference taxa across the angiosperm tree of life has greatly facilitated the refinement of comparative gene surveys. All reference angiosperm species outside Orobanchaceae exhibited a conventional and nearly complete set of N‐mt OXPHOS genes. The nuclear‐encoded *SDH3* and *SDH4* in CII were absent or recovered as relic, highly reduced gene fragments in 10 (38%) and 7 (27%) species, respectively (Figure 1). These absences are expected because both genes are still primarily encoded by mitochondria in many angiosperms with frequent mito‐to‐nuclear gene transfers in others (Huang et al. 2019; Mower 2020; Palmer et al. 2000). Therefore, they are not detectable in reference genomes that only include the nuclear sequences. The accessory subunit *SDH8* of CII was also frequently absent, but this is likely to be a result of its small size (46 amino acids in *Arabidopsis*) making it challenging to identify bioinformatically (Huang et al. 2019). Among the other four major complexes, CI is the largest, consisting of 52 proteins, but we found only two sporadic losses of conserved accessory membrane proteins *B14.5b* in *Cinnamomum* (Lauraceae) and *B12* in *Ipomoea* (Convolvulaceae). For the AOX complex, all species except *Manihot* (Euphorbiaceae) have at least one copy of the AOX1a‐c/e gene, but the more dispensable and redundant AOX1d was lost in 11 species. For the stress‐related AOX2, three independent losses were found in monocots, *Populus* (Salicaceae), and *Cuscuta* (Convolvulaceae). Beyond OXPHOS, the mitochondrial RRR and ribosomal proteins were universally present except for the loss of mitochondrial RRR protein *FPG1* in *Aquilegia*, the loss of *rps14* ribosomal protein in *Theobroma*, and the putative loss of nuclear *rps10* in 10 species (Figure 1). Like *SDH3* and *SDH4, rps10* can be encoded by both the nuclear and mitochondrial genomes; thus, its absence in the nuclear genome may indicate that it is encoded by the mitochondria in these species.

Despite the consistent presence of these N‐mt genes, angiosperm lineages possessed variable numbers of homologs due to historical and ongoing duplication, loss, and transfer. Such copy number variations were lineage‐ and gene‐specific. For example, the average gene copy number was three times higher in the blueberry *Vaccinium corymbosum* (*n* = 3.9) compared to all other species (*n* = 1.3). This is likely a result of the combined effects of multiple rounds of whole genome duplications as well as tandem gene duplications in *Vaccinium* (Larson et al. 2020; Wang et al. 2020). Similarly, species in asterids and rosids had multiple copies of *MPPα*, coinciding with the core eudicot hexaploidy event but may also be influenced by tandem duplication (Data S1; (Jaillon et al. 2007)).

### 3.2 Loss of N‐Mt Genes Is Associated With Relaxed Selection in Orobanchaceae

Nuclear‐mt gene losses in Orobanchaceae exhibited a strong lineage‐specific pattern that was restricted to the holoparasitic tribe Orobancheae (Figure 1). This pattern was not characteristic of all holoparasitic lineages because we found limited gene loss in a lineage consisting of *Aeginetia* and *Christisonia*. We made substantial efforts to verify this lineage‐specific gene loss pattern—first, data quality is unlikely to explain this pattern because the level of missing eukaryote BUSCOs did not show a significant difference between Orobanchaceae and other angiosperms (phylANOVA *p‐*value = 0.266) or between species with transcriptome versus genome data (phylANOVA *p‐*value = 0.185; Table S1). However, there were more fragmented eukaryote BUSCOs in Orobanchaceae (phylANOVA *p‐*value = 0.003) because within Orobanchaceae, species with transcriptomes had more fragmented eukaryote BUSCOs compared to genomic data (phylANOVA *p‐*value = 0.019). There were also more missing land plant BUSCOs in Orobanchaceae due to the evolution of parasitism (phylANOVA *p‐*value = 0.001) (Cai et al. 2021). Yet our conservative approach of counting fragmented N‐mt genes should accommodate this data quality issue. Furthermore, the holoparasitic tribe Orobancheae demonstrated significantly more losses in N‐mt genes when corrected for the background missing genes using 10,000 empirical permutations in Fisher's exact test (adjusted *p‐*value < 1e‐4). Second, Orobanchaceae did not show higher sequence divergence with *Arabidopsis* compared to other angiosperms (Table S4). The average pairwise sequence divergence between Orobanchaceae and *Arabidopsis* is 0.32 for raw nucleotide difference and 0.42 under the F84 substitution model, both of which are comparable to other angiosperms (phylANOVA *p‐*value 0.52 and 0.55, respectively). Finally, the strong phylogenetic signal of missing N‐mt genes, confined to the tribe Orobancheae, strongly supports this finding. In contrast, a full set of N‐mt genes were readily identifiable in other hemi‐ and holoparasitic species with comparable data quality to that in Orobancheae. Losses within Orobancheae were also validated by two high‐quality genomes from *Phelipanche aegyptiaca* and *Orobanche cumana*. In two species, *Epifagus virginiana* and *Aphyllon fasciculatum* , N‐mt losses were supported by multiple RNA samples from different individuals (Table S1).

Within tribe Orobancheae, gene losses were most significant in CI and CV, but were not remarkable in CII, CIII, and CIV. Most of the losses in CI were restricted to conserved accessory proteins and plant‐specific accessory proteins, and no genes encoding the CI core subunits were lost. These CI gene losses have taken place several times independently in species such as *Phelipanche aegyptiaca*, *Orobanche cumana*, *Conopholis americana* , or their common ancestors (Figure 1). These included membrane proteins *B12‐2* and *AGGG* as well as peripheral proteins *B14.5a* and *C1‐FDX* (Subrahmanian et al. 2016). Moreover, we also found two rare losses in CV located in *ATP5* which encodes the OSCP subunit of the peripheral flexible hinge of the ATP synthase (Zancani et al. 2020). Beyond OXPHOS enzymes, the holoparasitic Orobancheae also only had 91.5% of the organellar and mitochondrial‐specific RRR genes compared to other angiosperms.

Although a nearly complete set of N‐mt genes was present in *Aeginetia* and *Christisonia*, this lineage is a hotspot for horizontal gene transfer (Figure 1 and Data S1). In at least eight cases, putative host‐derived N‐mt genes either replaced or were found alongside native copies (see caveats in the Discussion). These include three repeated transfers of OXPHOS genes from Poaceae to *Christisonia* or *Aeginetia* (82, 100, and 100 UFBP in IQ‐TREE); one from Poaceae to *Pedicularis* (82 UFBP); one from Apiaceae to *Striga* (78 UFBP); one from Rosaceae to *Christisonia* (99 UFBP); and one from Asteraceae to *Aphyllon* (100 UFBP; Data S1). All putative gene transfers were restricted to OXPHOS genes, and none were identified among the RRR genes. No transfer was found in *Cuscuta australis* (Convolvulaceae), which is a holoparasitic plant from the morning glory family.

OXPHOS gene losses and horizontal transfers coincided with relaxed selection in Orobancheae. RELAX analyses on the concatenated sequences revealed that tribe Orobancheae experienced relaxed selection in OXPHOS complexes (*p‐*value < 0.0267), intensified selection in RRR proteins (*p‐*value = 0.0039), and no significant change in nuclear‐encoded mitochondrion‐targeted ribosomal proteins (*p‐*value = 0.7145; Table S5). Within OXPHOS, Orobancheae experienced significant relaxed selection in CI, CII, and CIII (*p‐*value < 0.0267) but no significant changes in CIV, CV, or AOX. An alternative model where relaxed selection took place in both tribe Orobancheae and the holoparasitic *Aeginetia* + *Christisonia* clade was not favored based on the likelihood ratio test (ΔLog(L) = 0.30, Chi‐square *p‐*value > 0.05). Gene‐specific RELAX analyses were consistent with these findings. Within OXPHOS complexes, relaxed selection in Orobancheae was detected in six CI genes, two CII genes, one CIII gene, and one CIV gene (Table S5). The RRR showed a mix of signals where relaxed selection was identified in five genes and intensified selection was found in three genes. No mitochondrial ribosomal genes demonstrated differentiated selection in Orobancheae.

### 3.3 Evolution of AOX in Orobanchaceae

**FIGURE 2:**
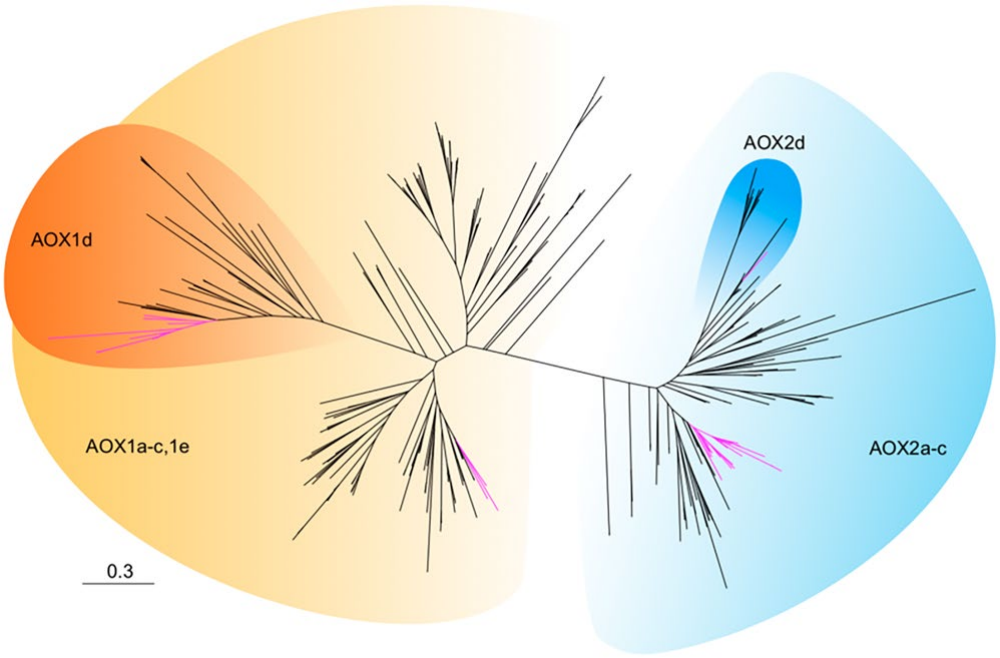
Phylogeny of AOX showing four major clades in angiosperms including AOX1a–c/1e, AOX1d, AOX2a–c, and AOX2d. The phylogeny was inferred based on protein alignments using the Maximum Likelihood method implemented in IQTREE. Branches are drawn in proportion to genetic distance (substitutions per site) and Orobanchaceae gene copies are highlighted in magenta. Note the single Orobanchaceae branch in the AOX2d subclade resulting from a horizontal gene transfer from Rosaceae to *Christisonia kwangtungensis*. A fully annotated newick tree is provided in Data S1.

Given its potential role in mitochondrial functional rescue, we investigated the evolution of AOX genes in Orobanchaceae, which are responsible for alternative respiratory pathways. We identified four clades: AOX1a–c/1e, AOX1d, AOX2a–c, and AOX2d in the two subfamilies AOX1 and AOX2, as suggested by previous investigations (Figure 2 and Data S1; (Costa et al. 2014)). In free‐living angiosperms, the AOX1a–c/1e and AOX2a–c clades are nearly universally present except that AOX2a–c is absent in monocots. The AOX1d and AOX2d clades, on the other hand, are more dispensable and are hypothesized to originate from convergent evolution for stress response in plants (Costa et al. 2014). In Orobanchaceae, widespread losses are identified in the highly conserved AOX1a–c/1e clade, but the AOX1d is relatively conserved (Data S1). AOX1a–c/1e is only recognizable in *Conopholis* + *Epifagus*, *Striga*, *Lindenbergia*, as well as *Christisonia*, the last of which is a result of gene transfer from likely Convolvulaceae (86 UFBP). This is in stark contrast with other angiosperm lineages where AOX1a–c/1e was identified in all species except *Manihot*. On the other hand, AOX2a–c is present in all Orobanchaceae with a few losses outside this family in *Cuscuta* (Convolvulaceae), *Populus* (Salicaceae), and monocots. The distinct AOX2d clade, though, was restricted to a few rosid families such as Fabaceae and Rosaceae. It is not present in any Orobanchaceae species except in the gene transfer from a Rosaceae clade including *Prunus* and *Fragaria* to *Christisonia* (Data S1).

### 3.4 Increased Reliance on Alternative OXPHOS Pathways in Species With Gene Losses

**FIGURE 3:**
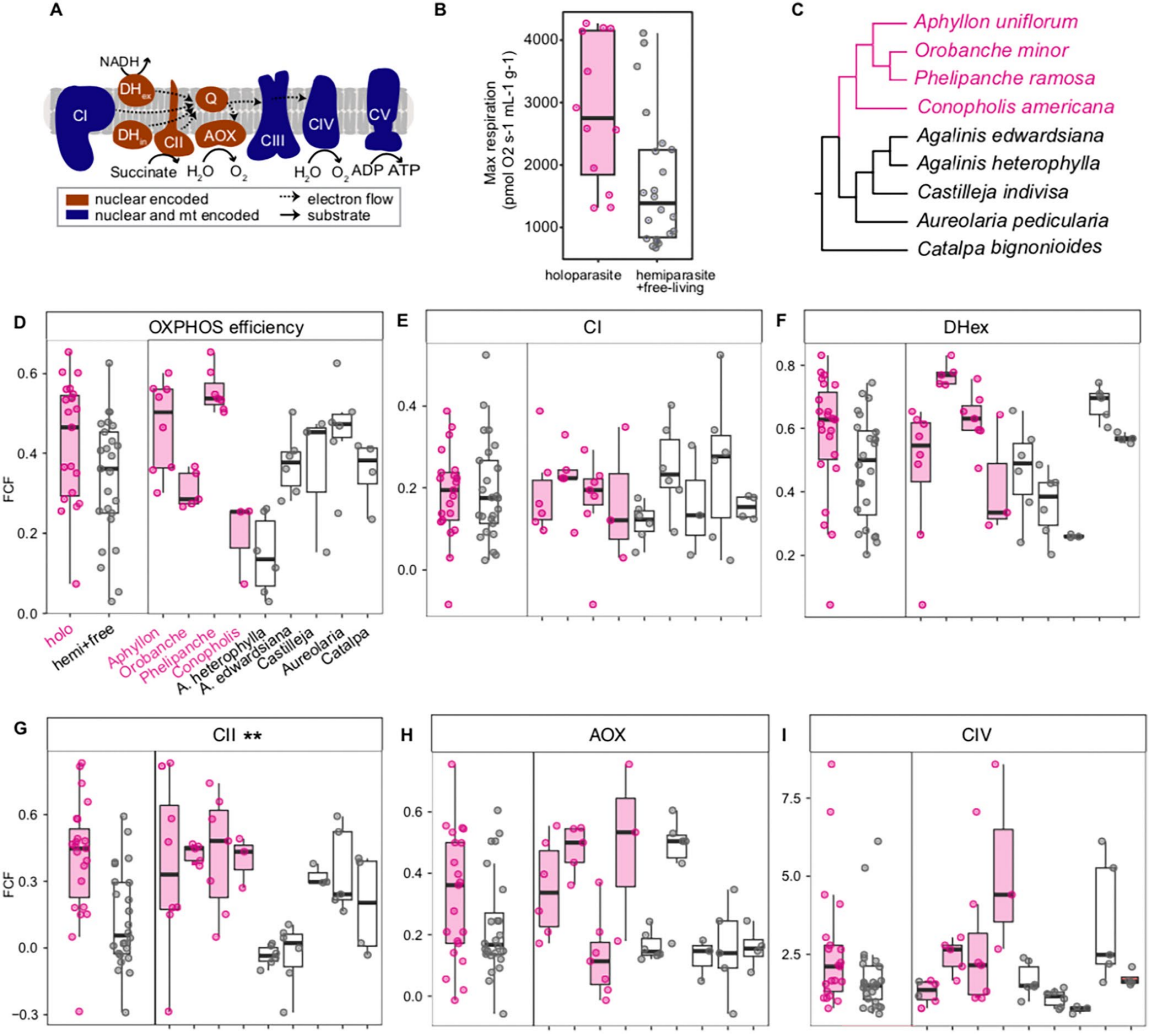
Increased reliance on nuclear‐encoded OXPHOS pathways in holoparasitic Orobanchaceae. (A) Plant mitochondrial electron transport chain components. Electrons enter through complex I (CI), CII, or the alternative NADH dehydrogenases (DH_ex/in_) and follow either the alternative oxidase pathway (AOX) or the cytochrome pathway CIII–CIV to reduce oxygen to water. CI, CIII, and CIV translocate protons to establish the proton motive force used by CV for ATP production. (B) Normalized maximum mitochondrial respiration of holoparasitic species with extensive gene loss and their close relatives. (C) Phylogenetic relationship among the nine species sampled in this study summarized from Mortimer et al. (Mortimer et al. 2022). (D–H) Comparison of flux control factors (FCF) between the two groups of species for OXPHOS efficiency (D), CI (E), DH_ex_ (H), CII (G), AOX (H), and CIV (I). Statistical differences at *p*‐value < 0.05 are marked by “**”.

Despite our findings of substantial N‐mt OXPHOS gene loss, holoparasitic Orobancheae consistently demonstrated robust mitochondrial respiration, with similar OXPHOS efficiency compared to species showing no sign of gene loss (Figure 3D; LME *p‐*value = 0.612; Data S2). The normalized maximum respiration also showed a similar trend, with holoparasites having similar maximum respiration (Figure 3B; LME *p‐*value = 0.2146). Across individual complexes, holoparasitic Orobancheae tended to have higher FCFs in the fully nuclear‐encoded complexes CII, AOX, and DH_ex_. However, this trend was only significant for CII, where holoparasitic Orobancheae demonstrated more than threefold higher CII flux (LME *p‐*value = 0.02). Differences in the two alternative complexes DH_ex_ and AOX were not statistically significant (LME *p‐*value > 0.23) but showed a consistent trend of higher flux control in holoparasites by 21.3% and 39.1%, respectively (Figure 3F,H). For the chimeric complexes CI and CIV, holoparasites had similar flux as the other taxa without gene loss (LME *p‐*value > 0.25).

Overall, holoparasitic Orobancheae with gene loss tended to show higher flux levels across all measured variables, although only reaching statistical significance for CII (Figure 3). However, this trend obscures considerable variation within and among species, and the overall trends observed between holo‐ versus hemiparasites were not uniformly distributed. For example, there was uniformly low AOX flux control in the holoparasitic *Phelipanche ramosa* and high AOX flux control in the hemiparasitic *Agalinis edwardsiana* (Figure 3H), which goes against the general trend of high AOX flux in holoparasitic Orobancheae. Unfortunately, many of the species available for O2K experiments did not have available genetic resources, making it difficult to correlate putative losses of individual N‐mt genes with OXPHOS flux.

**FIGURE 4:**
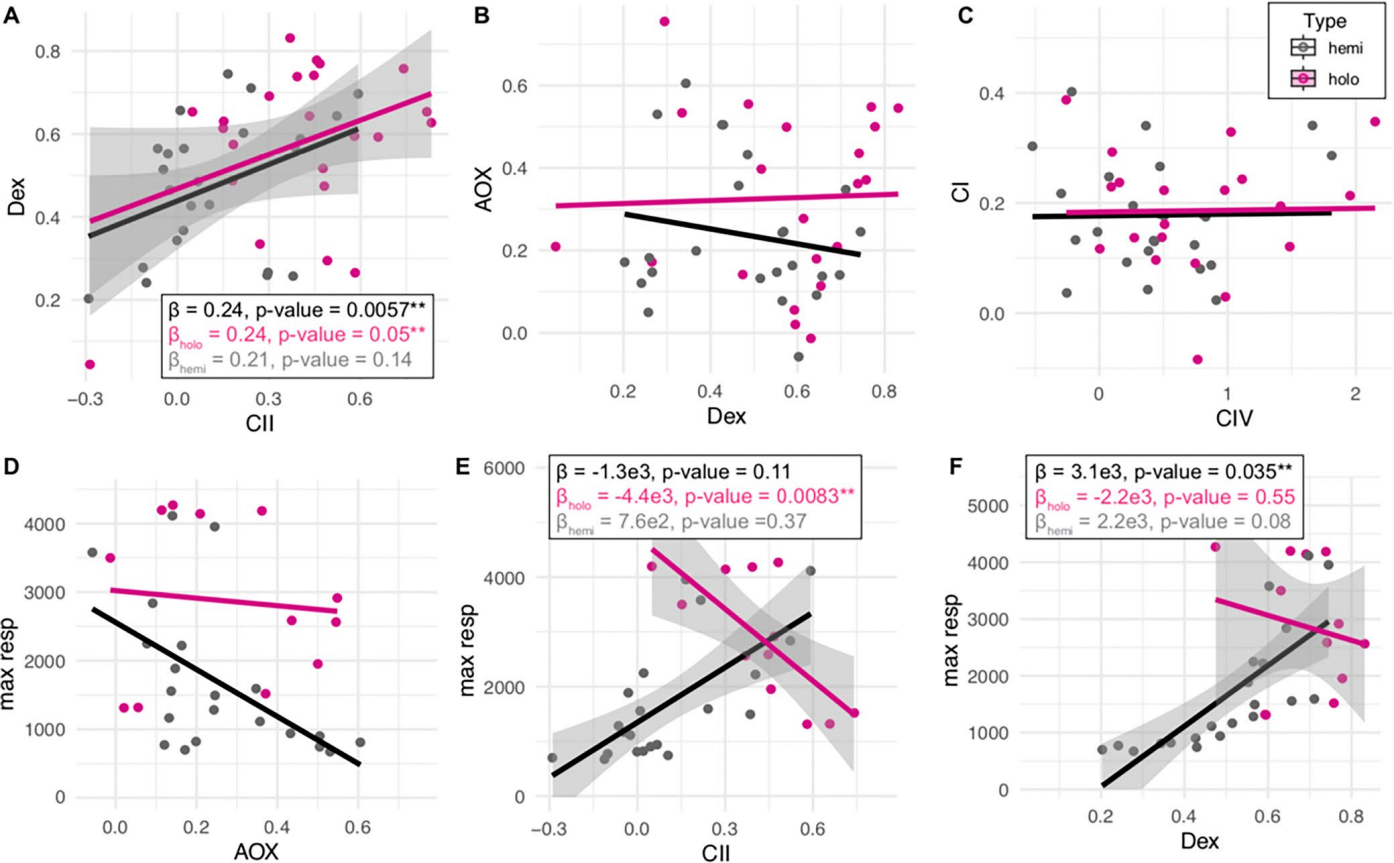
Relationships among FCF types in Orobanchaceae with different life history strategies. Magenta and black lines show the fitted linear mixed‐effects (LME) models for holoparasitic and hemiparasitic Orobanchaceae, respectively. The standard deviation, *p*‐values, and correlation coefficients *β* of LME models are only shown in significant relationships for the global (top) and partitioned datasets (middle and bottom). The six panels depict correlations of FCF between DHex and CII (A), AOX and DHex (B), CI and CIV (C), max respiration and AOX (D), max respiration and CII (E), nad max respiration and DHex (F).

Most FCFs did not show a significant correlation with each other, including comparisons among nuclear versus chimeric complexes (Figure 4). However, a significant positive correlation was identified between the fully nuclear encoded DH_ex_ and CII FCFs (*β* = 0.241, LME *p*‐value = 0.0057). Here, holo‐ and hemi‐parasites showed similar responses, with the regression coefficient *β* being 0.242 and 0.207, respectively (Figure 4A). None of the other pairwise comparisons, including nuclear versus chimeric (e.g., CI versus CII), chimeric versus chimeric (e.g., DH_ex_ versus AOX), or maximum respiration versus OXPHOS complexes (e.g., maximum respiration versus AOX), demonstrated significant global correlations (Figure 4 and Table S6). Some of the lack of correlation was likely driven by the disparate patterns shown in holo‐ versus hemi‐parasites. This was most obvious between maximum respiration rate and DH_ex_/CII flux (Figure 4E,F). In both cases, the maximum respiration rates were negatively correlated with DH_ex_ (*β* = −2.2e3; LME *p*‐value = 0.55) and CII (*β* = −4.4e3; LME *p*‐value = 0.0083) in holoparasitic Orobancheae, but a positive correlation was recovered for DH_ex_ (*β* = 2.2e3; LME *p*‐value = 0.08) and CII (*β* = 7.6e2; LME *p*‐value = 0.37) in the hemiparasitic and free‐living species.

## 4 Discussion

The parasitic lifestyle has been frequently associated with genome degradation in eukaryotes (Poulin and Randhawa 2015). In land plants, it is clearly established that both the nuclear and plastid genomes are subject to gene loss and relaxed selection when parasitism evolves (Lyko and Wicke 2021). Yet how parasitism or heterotrophism in general impacts the mitochondrial genome and function remains controversial (Cai et al. 2025). On one hand, unprecedented loss of the entire OXPHOS CI is documented in mistletoe (Skippington et al. 2015), which is a genetic hallmark of relaxed selection due to reliance on alternative respiratory pathways (Senkler et al. 2018). On the other hand, mitochondria perform multiple critical housekeeping functions including ATP production and oxidative stress response that seem to be indispensable even for parasites. In mistletoes, the mitochondrial function under genetic impact is maintained by redundant or alternative pathways that are entirely nuclear‐encoded, and the production of ATP via glycolytic oxidation does not require mitochondria (Maclean et al. 2018; Senkler et al. 2018). Our combined genetic and physiological investigation of Orobanchaceae mitochondria adds to the paradox that despite clear signatures of relaxed selection, respiratory functions can remain intact because of the recruitment of alternative electron transport pathways.

Unlike other reported cases of mitochondrial gene loss in parasites, losses and transfers in Orobanchaceae are restricted to nuclear‐encoded genes. In our companion paper focusing on mitogenome evolution, all 45 species in Orobanchaceae displayed a complete and native set of core mitochondrial‐encoded OXPHOS genes, including the holoparasitic tribe Orobancheae (Cai et al. 2025). The putative losses in N‐mt genes are restricted to conserved accessory subunits of OXPHOS complexes and account for no more than 20% of the total protein composition of each complex. Yet some of them may confer profound functional impact. For example, loss of the shared organellar RRR genes may lead to improper RRR machinery that promotes accelerated nucleotide substitutions (Guisinger et al. 2008; Park et al. 2017; Parkinson et al. 2005; Sloan et al. 2012). This was demonstrated in the plastid genome of *Geranium* (e.g., Parkinson et al. 2005) and is potentially relevant for the degenerated plastid genomes of holoparasites (Wicke et al. 2016). Widespread loss of the mitochondrial‐specific RRR like *RECA3* in tribe Orobancheae may be linked with significantly accelerated mitochondrial genome shuffling we have found in these holoparasites (Cai et al. 2025) because disruption of this gene resulted in extensive rearrangement of the mitochondrial genome in *Arabidopsis* (Miller‐Messmer et al. 2012). Moreover, we found two putative losses of *ATP5* in *Aphyllon* and *Conopholis*. In *Arabidopsis*, reduced *ATP5* expression results in stunted seedling growth in dark conditions and altered leaf shapes in light due to lower ATP levels (Robison et al. 2009). In yeasts and *Fusarium* fungi, loss of *ATP5* similarly results in severely impaired nutritional growth (Uh et al. 1990; Yang et al. 2023). In Orobanchaceae, the parasitic lifestyle may allow species to cope with these otherwise deleterious mutations, but additional experiments and genomic data are needed to verify whether they are truly lost or expressed at low levels. For example, *ATP5* encodes the OSCP subunit of ATP synthase, which is sensitive to oligomycin. Lack of oligomycin sensitivity in *Aphyllon* and *Conopholis* can thus verify the unusual loss of *ATP5* in these species. Other losses can be confirmed by Western blots to examine the size of these protein complexes and explore the possibility of directly importing host‐derived proteins or mRNAs. Demonstrating absence is challenging, but given the BUSCO evaluation, the biased phylogenetic distribution of missing genes, and the result from the RELAX analysis, we feel confident that relaxed selection on mitochondrial respiration has allowed the fixation of slightly deleterious mutations such as gene loss and horizontal gene transfer in the tribe Orobancheae. Our multi‐faceted approach to verify gene loss also sets an example for future work in non‐model organisms to utilize transcriptome data to draw reasonable conclusions from comparative genomic investigations.

Eight putative horizontal gene transfers were detected in nuclear‐encoded OXPHOS genes within Orobanchaceae, especially in *Christisonia*. Two‐thirds of these genes are transferred from Poaceae, which are frequently invoked as both donors and receivers of horizontal gene transfers in previous investigations (Dunning et al. 2019; Hibdige et al. 2021). If these transfers are representative of the rest of the nuclear genome, then the 8.2% (*n* = 5) gene transfers make *Christisonia* the species most prone to nuclear gene transfers among land plants, which is nearly six times that of *Sapria* (Rafflesiaceae) at 1.4% across its nuclear genome (Cai et al. 2021). However, these gene transfers may be enriched in OXPHOS genes rather than reflecting the genomic average. Such enrichment of gene transfers in certain metabolic pathways aligns with the ‘functional necessity’ hypothesis of gene transfer where alien copies are recruited to compensate for the loss of indispensable functions such as mitochondrial respiration (Cai 2023). Finally, there are several important caveats to consider when interpreting these gene transfers. Only four out of eight transfers have support values larger than 95 UFBP to be considered as high confidence in IQ‐TREE (Hoang et al. 2018). The moderate phylogenetic support values can be attributed to the short length and conserved sequences of these OXPHOS genes. Moreover, most gene transfers were identified using RNA sequencing and may represent host RNAs rather than gene transfers (David‐Schwartz et al. 2008). Future genomic sequencing of holoparasitic Orobanchaceae including *Christisonia* will confirm the genetic background of these putative alien N‐mt genes.

Despite widespread gene loss, horizontal gene transfers, and elevated non‐synonymous substitutions indicative of relaxed selection, mitochondria of the holoparasitic Orobancheae performed similarly to the other species in high‐resolution respirometry experiments but tended to show a higher capacity for OXPHOS flux and overall mitochondrial respiration. However, intra‐species variation was large even though these flux measurements were internally normalized. These variations could be caused by additional developmental and environmental factors that mobilized mitochondrial respiration in different ways. After accounting for these variations, the only significant difference we found was in CII capacity. Such a shift to increased CII flux may reflect a functional rescue of the compromised chimeric complexes due to gene loss, cytonuclear conflict, or overall dysfunction due to relaxed selection. For example, increased AOX respiration allows for adequate ATP production and maintenance of redox homeostasis in stressful environments (Sweetman et al. 2019; Vanlerberghe 2013), and our study is in line with previous work where similar stress may be induced via molecular changes in mitochondrial genes (Weaver et al. 2020). Species in the holoparasitic tribe Orobancheae, such as *Aphyllon* and *Orobanche*, rely heavily on AOX compared to hemiparasites. The only exception in hemiparasites that have high AOX flux is *Agalinis edwardsiana* . Unlike other angiosperms, Orobancheae may be using the more development‐related AOX2 subclade for this purpose (Figure 1) (Arnholdt‐Schmitt et al. 2006; Costa et al. 2014). Even in hemiparasitic Orobanchaceae, the stress‐related AOX1a–c/1e cannot be detected in most lineages and is replaced by the stress‐related AOX1d subclade (Clifton et al. 2006). Given that parasitic plants often actively extract water from their hosts (Atkinson and Atkinson 2020), the recruitment of AOX, especially stress‐related copies, may allow these species to rapidly produce ATP on demand to perform such energy‐intensive tasks. In free‐living plants, AOX is also frequently activated to cope with biotic and abiotic stressors such as drought and nutrient deficiency (Vanlerberghe 2013; Watanabe et al. 2008), so it may confer additional advantage during the transition to a parasitic lifestyle. Future genomic investigation of Orobanchaceae will be able to confirm the differentiated retention of AOX subfamilies and their role in mitochondrial respiration.

We also found a positive correlation between CII and DH_ex_ (Figure 4A), both of which are fully nuclear‐encoded alternative electron entries to CI. The positive correlation between CII and DH_ex_ has been shown previously in *Silene* (Havird, Noe, et al. 2019; Weaver et al. 2020) and may suggest simultaneous mobilization of alternative complexes when CI is compromised. The disparate patterns between CII/DH_ex_ and maximum respiration in holo‐ versus hemiparasites (Figure 4E,F) further corroborate the linked activities of alternative complexes and allude to the potentially different use of OXPHOS complexes in species with diverse life history strategies. Indeed, divergent signatures of selection are observed in both nuclear (Table S5) and mitochondrial‐encoded genes (Cai et al. 2025), with relaxed selection unique to holoparasitic Orobancheae.

Gene losses and transfers can disrupt coevolved protein complexes and potentially lead to functional changes driven by cytonuclear conflict. In the case of Orobanchaceae, lineages showing significant genetic alterations still maintain a robust mitochondrial respiration. This is achieved by shifting respiratory activities to fully nuclear‐encoded complexes less vulnerable to cytonuclear conflicts (i.e., CII). Our results add to the growing consensus that in plants, compromised mitochondrial respiration originating from cytonuclear conflict may be compensated by elevated activities of the alternative pathways. However, there are drastically different mechanisms underlying cytonuclear conflict, leaving divergent genetic signatures in *Silene*, *Viscum*, and in our case, Orobanchaceae. For example, the exceptionally high substitution rate of mitochondrial genes in *Silene* is a result of cellular‐level drift, especially in species with only one to two mitochondria per cell and possibly inefficient homologous repair mechanisms (Zwonitzer et al. 2024). These incompatible substitutions are partially compensated for by both corresponding mutations in nuclear genes and physiological plasticity to alternative respiratory pathways (Havird et al. 2017; Sloan et al. 2014; Weaver et al. 2020). This mechanism is in direct contrast with the pattern observed in parasitic lineages such as mistletoes and Orobanchaceae, which are likely a result of relaxed selection on OXPHOS respiration. Notably, in both lineages, genetic and physiological alterations arose after the origin of parasitism and are restricted to *Viscum* and the holoparasitic tribe Orobancheae, respectively. This indicates that the evolution of parasitism may prime specific lineages with the capacity to perform altered OXPHOS functions due to the availability of host resources. In the mistletoe *Viscum*, both nuclear and mitochondrial genes encoding CI have been lost, and this functional deficiency is partially rescued by increased activities of AOX and alternative ATP generation through cytosolic glycolysis of potentially host carbon (Maclean et al. 2018; Senkler et al. 2018). In the holoparasitic Orobancheae, we found a general trend of relaxed selection for N‐mt genes as well as their mitochondrial counterparts, but gene loss and horizontal gene transfer are restricted to N‐mt genes, but not present in mitochondria (Cai et al. 2025). Such biased rates of loss or transfer in the nucleus versus mitochondrion are also seen in the parasitic Balanophoraceae (Gatica‐Soria et al. 2024) and potentially in Rafflesiaceae (Cai personal communication). This phenomenon may represent a lag time and evolution‐in‐progress where mutational changes are reflected in the faster‐evolving nuclear genome before they are manifested in the mitochondrial genome, which is generally more slow‐evolving in plants. Our research underscores the importance of studying parasitic plants as model systems to unravel fundamental questions about mitochondrial evolution and physiological adaptation to different life‐history strategies. Specifically, determining whether N‐mt gene losses and mitochondrial respiratory changes are widespread features of parasitic plants remains an exciting direction for future comparative research.

## Author Contributions

**Liming Cai:** conceptualization (equal), data curation (lead), formal analysis (lead), funding acquisition (lead), investigation (lead), methodology (lead), project administration (lead), resources (lead), validation (lead), visualization (lead), writing – original draft (lead), writing – review and editing (lead). **Robert K. Jansen:** project administration (equal), resources (equal), supervision (equal), writing – review and editing (equal). **Justin C. Havird:** conceptualization (equal), funding acquisition (equal), methodology (equal), project administration (equal), supervision (equal), writing – review and editing (equal).

## Conflicts of Interest

The authors declare no conflicts of interest.

## Supporting information


Appendix S1.

Appendix S2.

Appendix S3.

## Data Availability

No new sequencing data were generated. All curated DNA sequences and raw data from the O2K respiratory experiments are provided in the Appendix. All scripts generated for the study can be accessed under the GitHub repository https://github.com/lmcai/Orobanchaceae O2K and Zenodo (10.5281/zenodo.15584982).
