# Supplementary material for: Altered Mitochondrial Respiration Is Associated With Loss of Nuclear‐Encoded OXPHOS Genes in Parasitic Broomrapes
Source: Ecol Evol. 2025 Jul 6;15(7):e71737. doi: 10.1002/ece3.71737 (PMC12230201; doi:10.1002/ece3.71737)
Supplement: Supplementary file 1 — Appendix S1. [file ECE3-15-e71737-s003.tgz › Orobanchaceae_O2K_Supplementary_v3/Note_S1.docx]

**Supporting Information Note 1**

**Plant mitochondrial isolation and O2K protocol**

1. **Lab preparation**

- Get a bucket or two of ice to keep cold of plant material
- Turn centrifuge on at 4°C
- Take a 50 mL tube of Mir05 buffer out of freezer and begin thawing on bench
- Make working solution of buffer with 2-mercaptoethanol (in the hood), mix, and put on ice
- Put 2 mortars and pestles on ice

1. **Isolating plant mitochondria**
2. Sterilize small scissors
   - Rinse with 70% ethanol and then DI water
3. Weigh out ~ 1g gram of rosette and cauline leaves from a plant in a weigh boat
4. Dice samples in weigh boat with scissors
   - Dice as small as possible
5. Record in notebook all the information on the plant’s tag and the weight of the leaves you used, can refer to each plant as “A-H” when labeling tubes, etc.
   - Use the Black notebook labeled “Rachel/Gina/Plant mt experiments” that should live over by the O2Ks
6. On the plant tag write “O2K” and the date
7. Grind and filter the sample
   - Keep everything on ice
   - Use a sterile mortar and pestle for each sample, put on ice to chill as long as possible before using – put a fresh mortar and pestle on ice when your done with the old one and it should have ~5-10 min to chill before you need it
   - Add the leaves and a couple squirts of the working mt plant isolation buffer with BSA/2-mercaptoethanol
   - Use mortar and pestle to grind the leaves and working solution until slushie-like – put mortar back on ice with slushie if you did dome grinding on bench
   - Place a funnel into a labeled 50mL test tube, stick in ice
   - Place a 2-layer square piece of cheesecloth on top of 1 layer of mira cloth and place both into the funnel
   - Collect the green liquid sample by placing the sample into the cloth, can use extra buffer to wash any green bits into funnel, but try to keep amount of buffer used to a minimum (should total about 4-5 mL per sample)
   - Squeeze the sample from the top down through the funnel and into the tube
   - <5mL is the ideal amount of sample to be collected
   - After each sample you’ll probably want to get a new pair of gloves since you’ll likely get Silene pesto on your gloves when squeezing
8. Separate each sample into two (or 3 if needed) 2 mL centrifuge tubes with a clean transfer pipette
9. KEEP SAMPLES ON ICE ALWAYS!!!!
10. Centrifuge samples at 4°C for 5 minutes at 2,790 rcf – mitos will be in supernant
11. Use 1000 uL pipet to carefully remove liquid from the top, transfer to a new set of labeled 2 mL tubes, can dispose of tube with pellet
12. Centrifuge samples at 4°C for 15 minutes at 12,200 rcf – mitos will be in pellet
13. Use 1000 uL pipet to carefully remove liquid from the top, can dispose of liquid, but keep tubes with pellet
14. Add 200μL plant mt isolation buffer without BSA (in fridge) to each tube
15. Use a paintbrush to resuspend the pellet carefully (use a separate paintbrush for each sample)
16. Combine each set of tubes into a single tube (i.e., use the 1000uL pipet to transfer the 200uL for each tube “A” into a single “A” tube with 400uL).
17. Centrifuge samples at 4°C for 5 minutes at 12,200 rcf – mitos will be in pellet
18. Use 1000 uL pipet to carefully remove liquid from the top, can dispose of liquid, but keep tubes with pellet
19. Resuspend the pellet in buffer without BSA (200μL), using the same paintbrushes for each sample as before
20. KEEP SAMPLES ON ICE, PUT ICE BUCKET IN FRIDGE WHEN THIS PART IS DONE
21. Clean funnels with soap and water, put on drying rack
22. **QuBit – Quantify protein per sample**

Follow the QuBit protein assay instructions to quantify the amount of protein in each sample. Briefly:

1. Make the QuBit working solution, assuming you’ll have 8 samples and 3 standards (make an extra 2 samples worth to account for pipetting errors, so 12 samples in total). Vortex to mix after all these steps.
2. Add the QuBit working solution to each sample tube (199 uL for samples, 190 uL for standards).
3. Add 1 uL of sample or 10 uL of standard to each tube, vortex.
4. Wait 15 mins (can clean up, make sodium pyruvate, etc.)
5. Vortex each tube before reading using the QuBit
6. Record the protein concentration for each sample in the notebook

- Amount of sample to be placed in O2K chamber:

$$\frac{0.25mg}{x mL of sample} = Quibit value \frac{\mathrm{mg}}{\mathrm{mL}}$$

$$x mL of sample desired= \frac{0.25 mg}{Quibit Value \frac{\mathrm{mg}}{\mathrm{mL}}}$$

$$x mL of sample \cdot1000 =x \mu L of sample desired$$

You should be adding on the order of 100uL of sample….

# **O2K Protocol**

1. Thaw the tray of substrates/inhibitors
2. Make Sodium Pyruvate – needs to be made fresh daily
   - Weigh a 1.5mL test tube
   - Place an amount of Sodium Pyruvate into the test tube and record the weight of the sample
   - Pipette Ultra Pure Water into the test tube, use equation below to calculate amount (y)
   - Vortex the solution

$x mg of Sodium Pyruvate \div0.22 =y \mu L of Ultra Pure Water$ to add

1. Turn on the O2K machine – enter your name for “user”, select defaults for O2K configuration
   - O2K control settings should be default, but temp at 25C
   - Under “sample” add details about the plant samples, protein amount you’ll be adding (should be 0.25 mg unless you don’t have enough), experiment, date, etc
   - Select layout 5a under “layout” -> Standard layouts
   - make sure lights are off – use hotkey F10 to change
2. Wash the O2K stoppers with DI water thoroughly – set aside
3. Suck out the ethanol from the chambers
4. Rinse the chambers with DI water 3 times – leaving the DI water in the chamber about 30s per rinse
5. Add 2.5 mL of thawed Mi05 to the chambers using 1000uL pipet
6. Place the stoppers back in the chambers all the way down – a little extra mir05 will come out of the top of the stoppers, suck it off but don’t put the sucker on the hole…
7. Pull the stoppers out of the chamber slowly, leaving just enough space for the fork to oxygenate until the oxygen concentration reading (blue line) is stable; consumption (red line) should be fairly stable, but not necessarily at 0
8. While stable, hold down shift and select the last 2 mins or so of stable data for each chamber. Then click on the yellow “O2 callib” button for each chamber and select that mark as the fully oxygenated standard.
9. After calibrating the 100% oxygen, push down the stoppers to close the chamber!!
10. Make sure there are no bubbles (can turn lights on briefly to check)
11. Add the mt samples to each chamber based on their Qubit concentration (see above)
12. Add substrates/inhibitors to the chamber based on the order and amounts (in uL) in the table below.

|  | **Mito** | **NAD^+^** | **CoA** | **TPP** | **M** | **P** | **G** | **ADP** | **NADH** | **S** | **ROT** | **nPG** | **ASC** | **TPMD** |
| --- | --- | --- | --- | --- | --- | --- | --- | --- | --- | --- | --- | --- | --- | --- |
| **μL** |  | 10 | 5 | 4 | 3 | 10 | 10 | 5 | 10 | 40 | 10 | 5 | 5 | 5 |
| **A** |  |  |  |  |  |  |  |  |  |  |  |  |  |  |
| **B** |  |  |  |  |  |  |  |  |  |  |  |  |  |  |

1. Suck out the chambers with a transfer pipet and put in the O2K waste bottle by the fume hood sink
2. Wash the O2K tubes and chambers with the long wash protocol:
   - Rinse the stoppers with DI water, then 70% EtOH, then 100% EtOH
   - 3 washes of the chambers with DI water
   - 3 washes with 70% ethanol
   - 3 washes with 100% ethanol
     - Leave the third ethanol in the chamber for 30 minutes
   - 3 washes with 70% ethanol
     - Leave the last 70% ethanol in the chamber
     - Place the tubes back in the chamber
     - Fill the top of the tubes with 70% ethanol
     - Put the cap back on the tubes
3. Turn the O2K machine off
