# Supplementary figures and images for: Altered Mitochondrial Respiration Is Associated With Loss of Nuclear‐Encoded OXPHOS Genes in Parasitic Broomrapes
Source: Ecol Evol. 2025 Jul 6;15(7):e71737. doi: 10.1002/ece3.71737 (PMC12230201; doi:10.1002/ece3.71737)

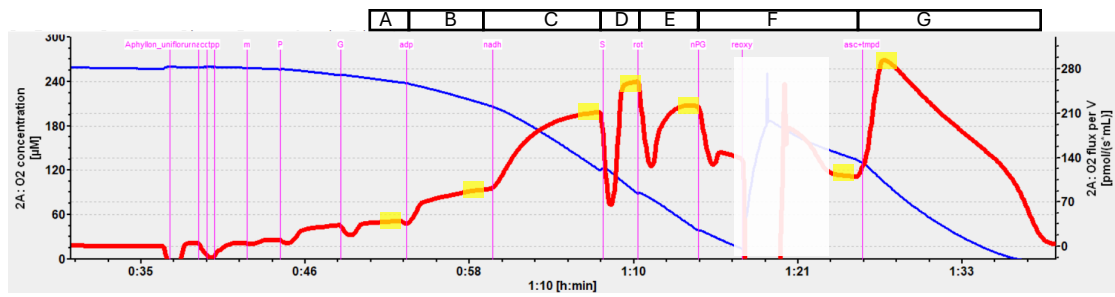

Supplement: Supplementary file 1 — Appendix S1. [file ECE3-15-e71737-s003.tgz › Orobanchaceae_O2K_Supplementary_v3/FigS1.pdf]

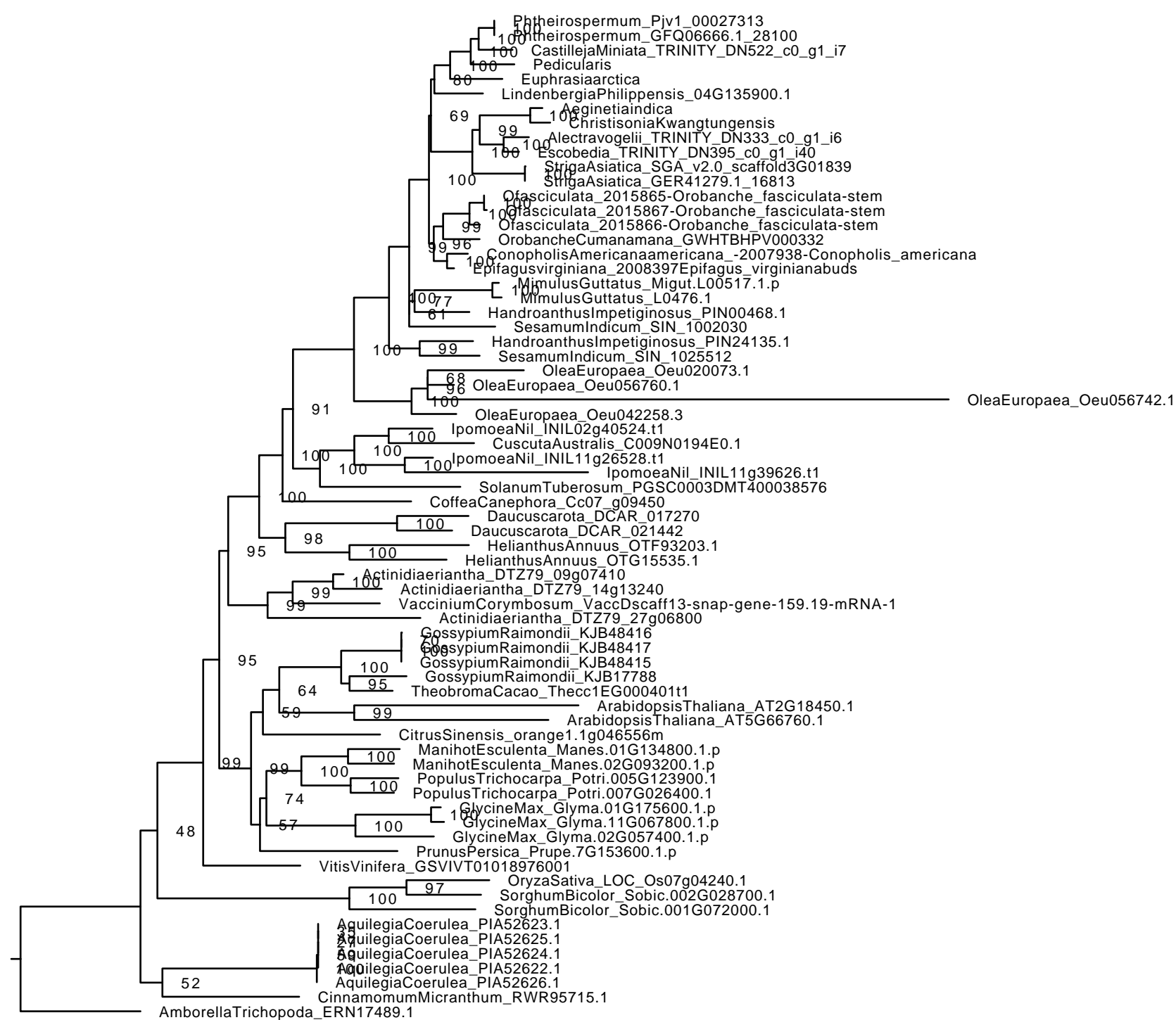

0.07

Supplement: Supplementary file 1 — Appendix S1. [file ECE3-15-e71737-s003.tgz › Orobanchaceae_O2K_Supplementary_v3/Data_S1/DataS1/CII/OG0004874.filter.fas.treefile.pdf]

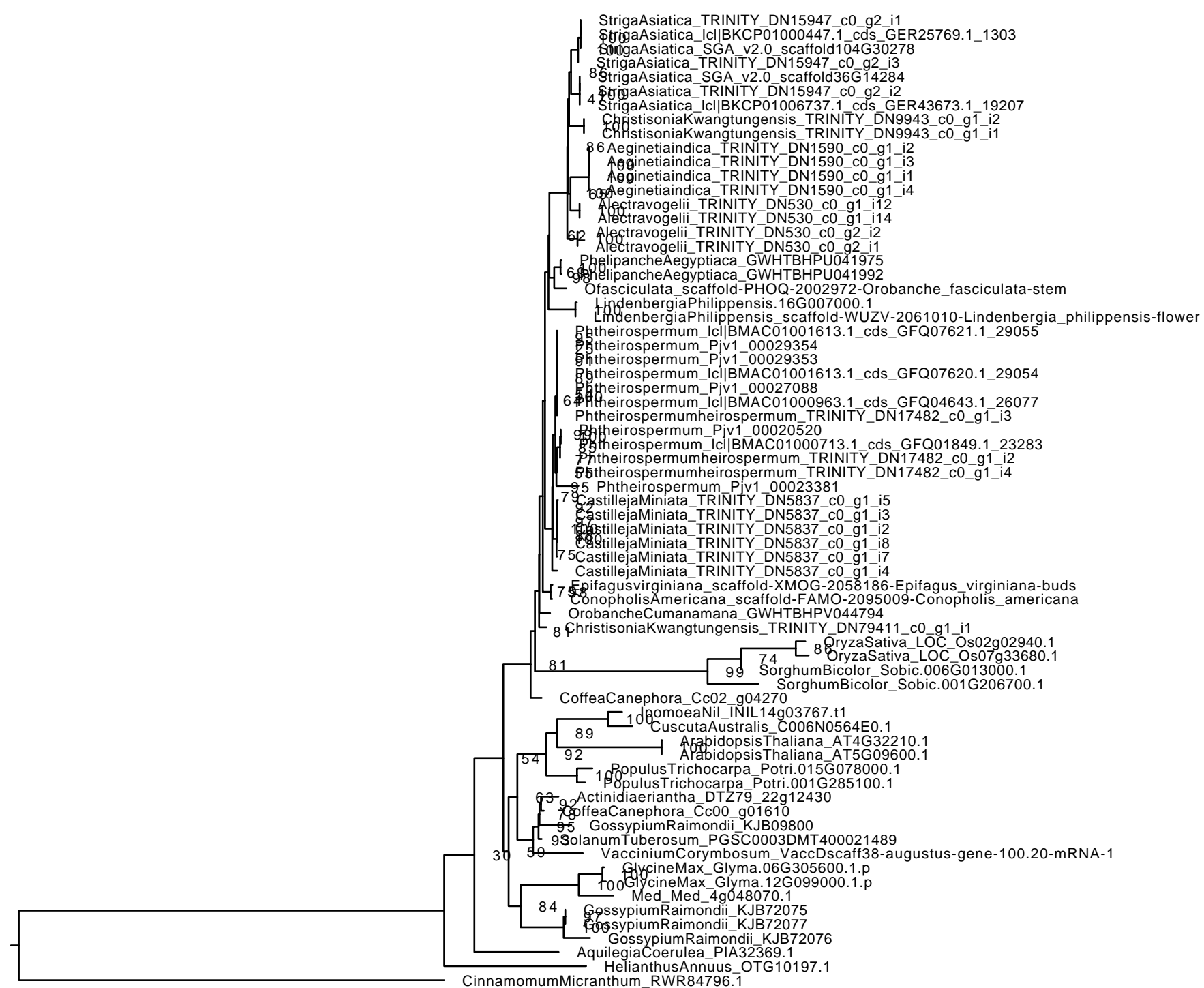

0.8

Supplement: Supplementary file 1 — Appendix S1. [file ECE3-15-e71737-s003.tgz › Orobanchaceae_O2K_Supplementary_v3/Data_S1/DataS1/CII/OG0009246.filter.fas.treefile.pdf]

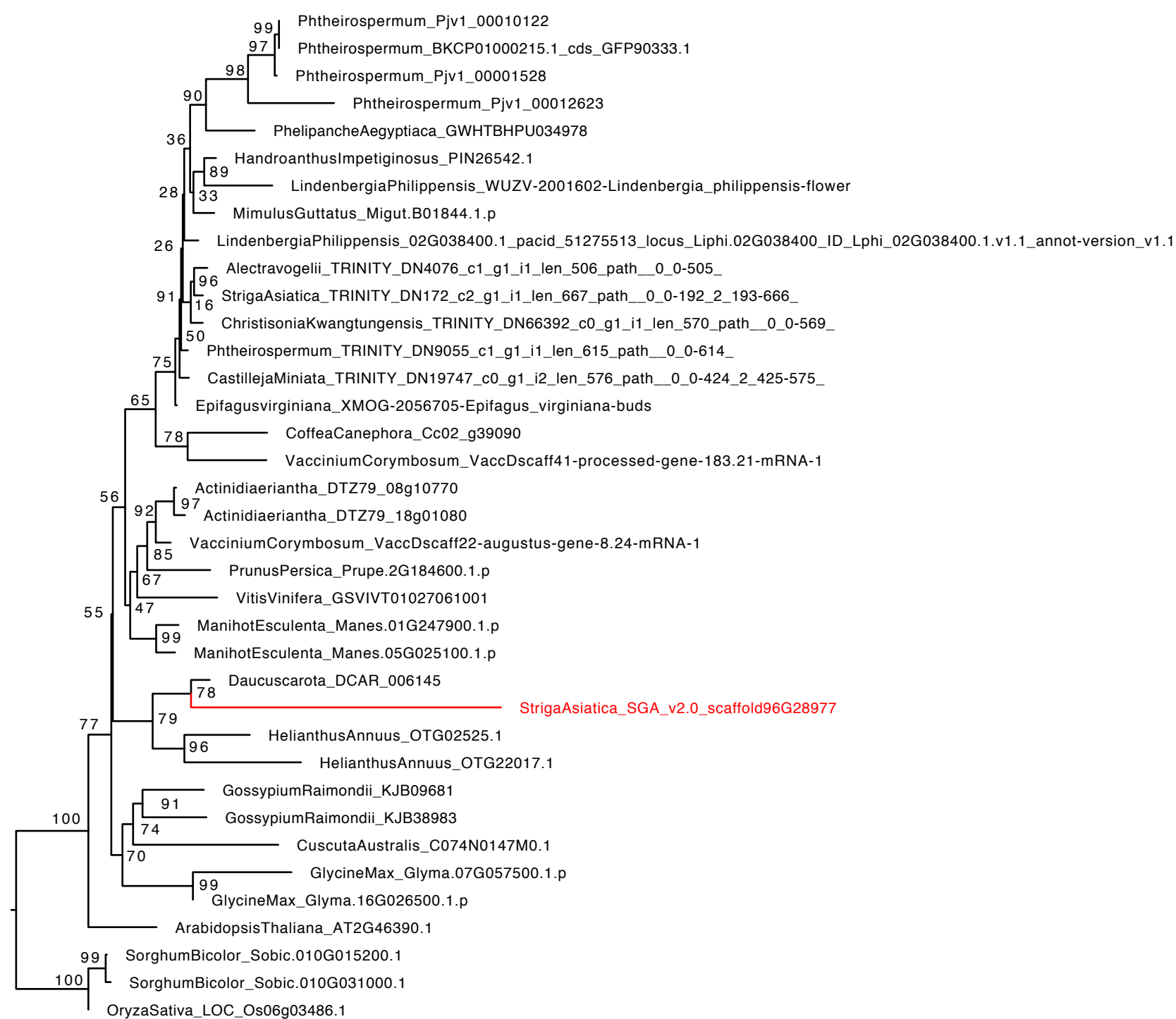

0.3

Supplement: Supplementary file 1 — Appendix S1. [file ECE3-15-e71737-s003.tgz › Orobanchaceae_O2K_Supplementary_v3/Data_S1/DataS1/CII/OG0010649.filter.fas.treefile.pdf]

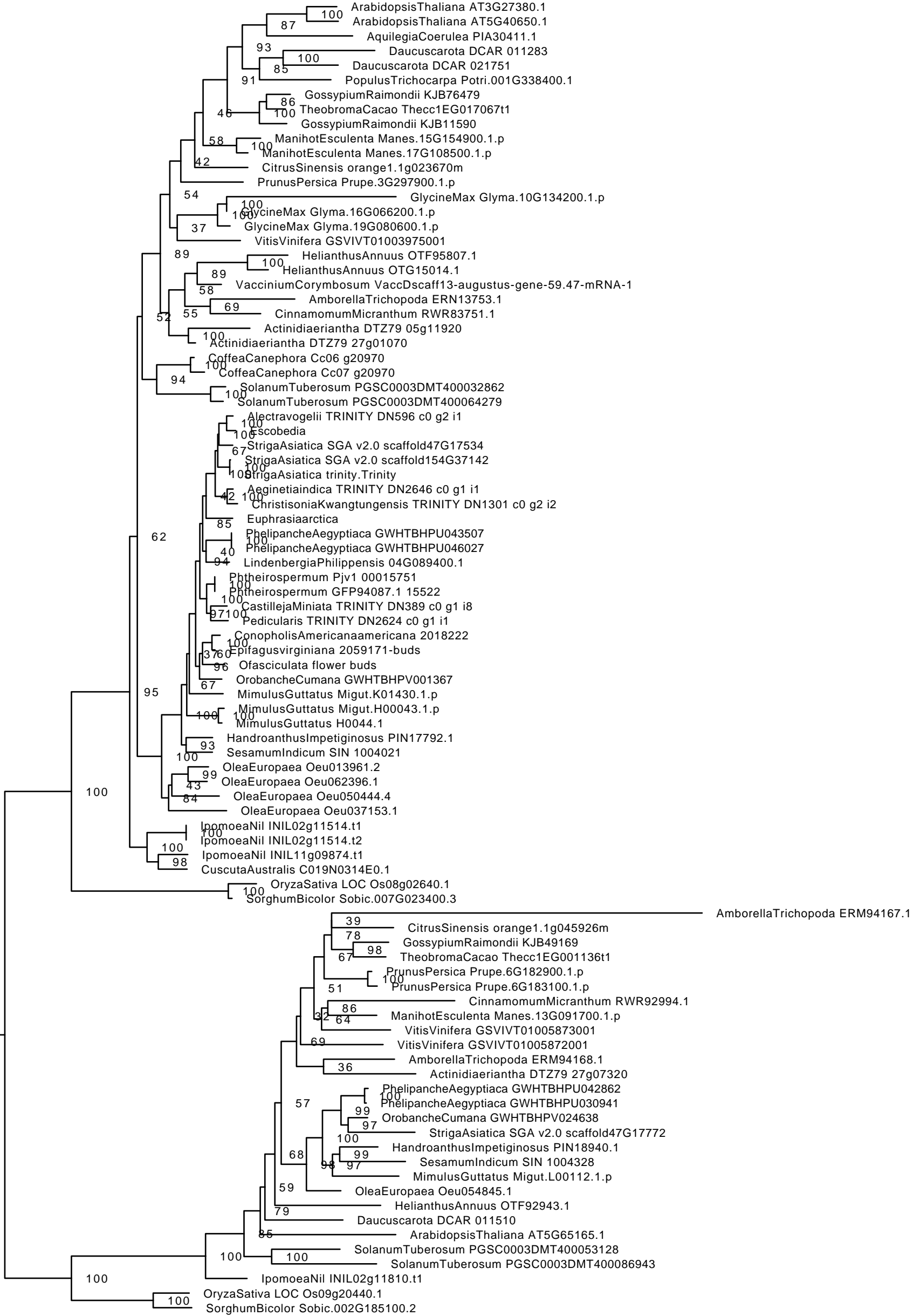

0.4

Supplement: Supplementary file 1 — Appendix S1. [file ECE3-15-e71737-s003.tgz › Orobanchaceae_O2K_Supplementary_v3/Data_S1/DataS1/CII/OG0003084.filter.fas.treefile.pdf]

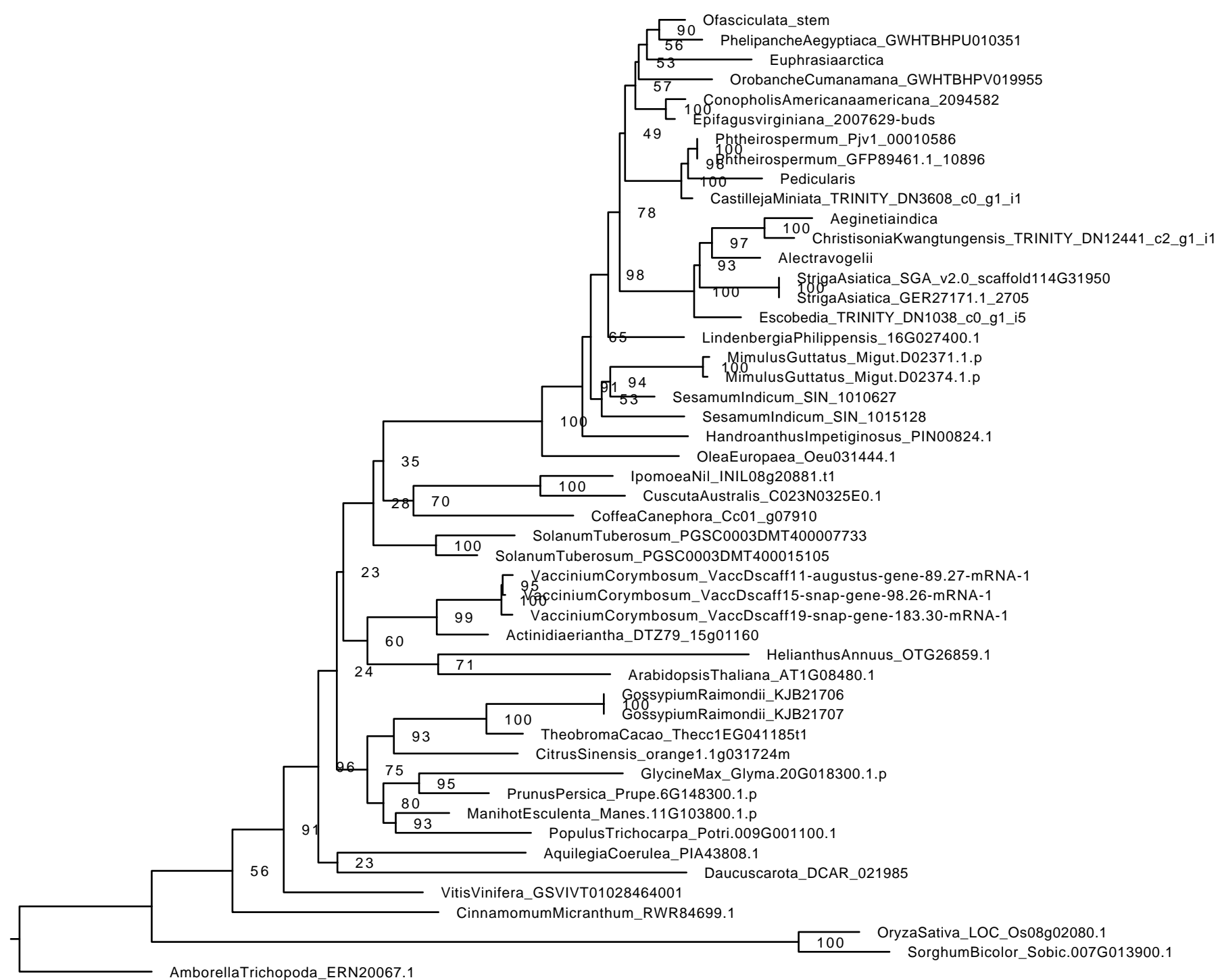

0.2

Supplement: Supplementary file 1 — Appendix S1. [file ECE3-15-e71737-s003.tgz › Orobanchaceae_O2K_Supplementary_v3/Data_S1/DataS1/CII/OG0009050.filter.fas.treefile.pdf]

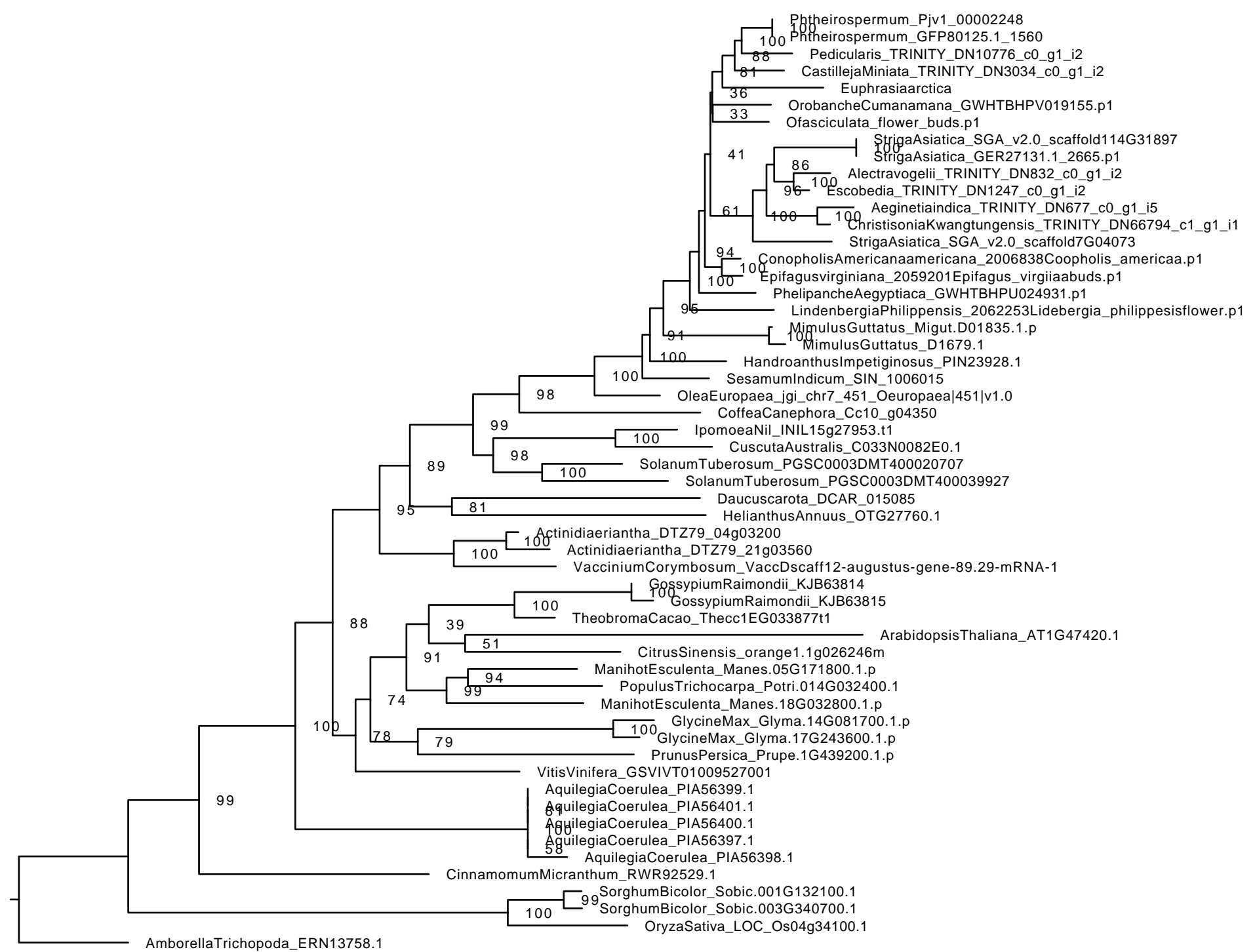

0.1

Supplement: Supplementary file 1 — Appendix S1. [file ECE3-15-e71737-s003.tgz › Orobanchaceae_O2K_Supplementary_v3/Data_S1/DataS1/CII/OG0007335.filter.fas.treefile.pdf]

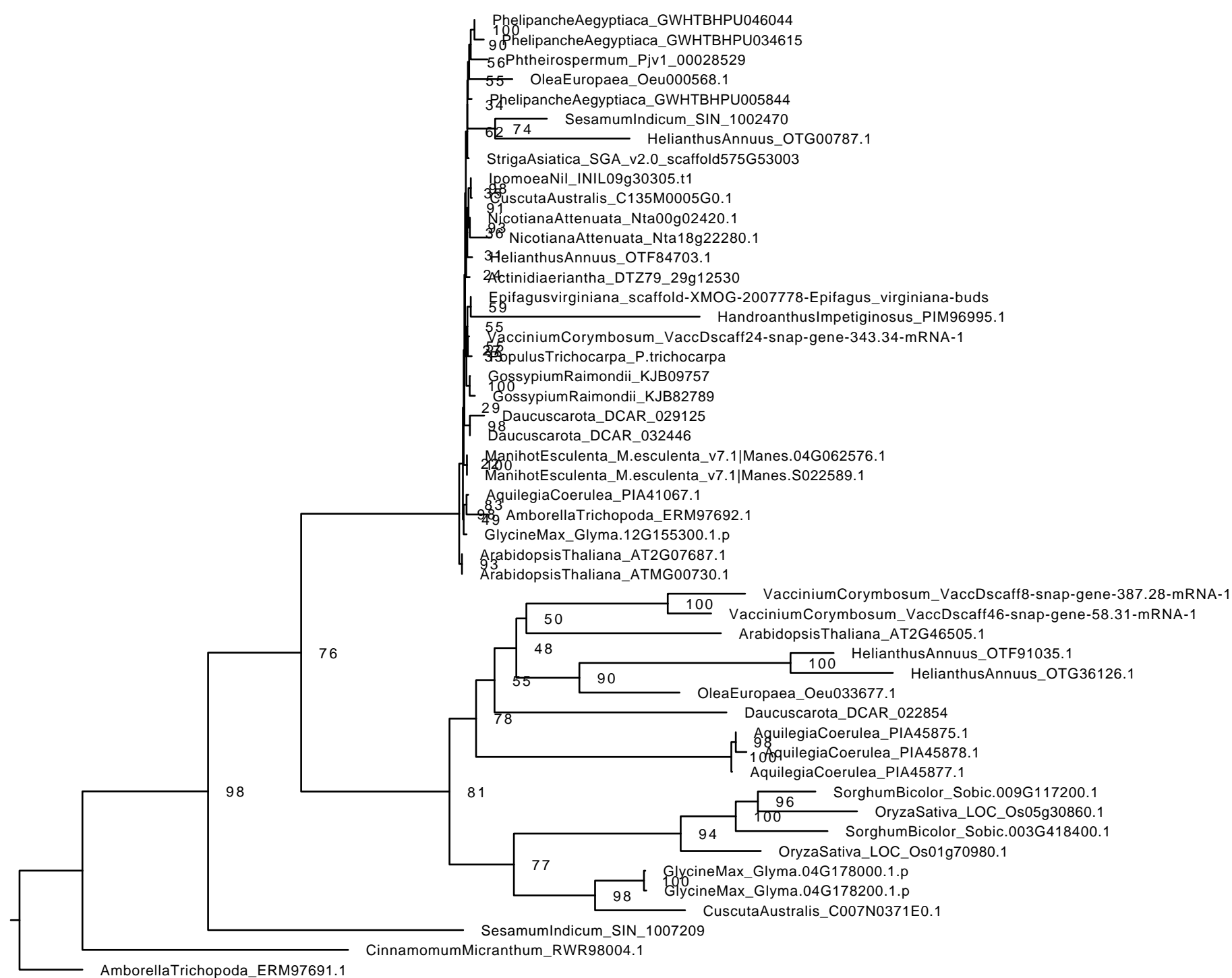

0.4

Supplement: Supplementary file 1 — Appendix S1. [file ECE3-15-e71737-s003.tgz › Orobanchaceae_O2K_Supplementary_v3/Data_S1/DataS1/CII/OG0004319.filter.fas.treefile.pdf]

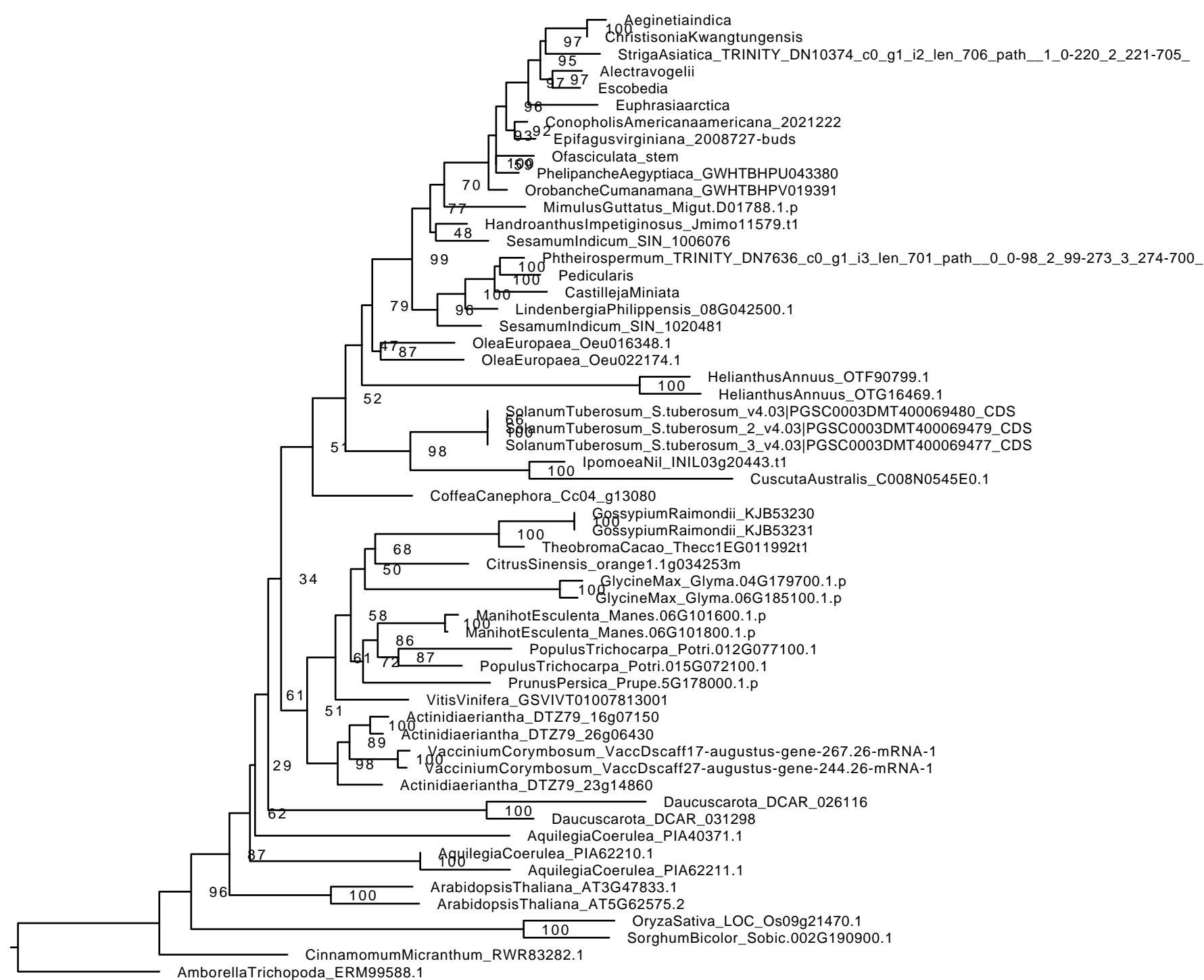

0.1

Supplement: Supplementary file 1 — Appendix S1. [file ECE3-15-e71737-s003.tgz › Orobanchaceae_O2K_Supplementary_v3/Data_S1/DataS1/CII/OG0007234.filter.fas.treefile.pdf]

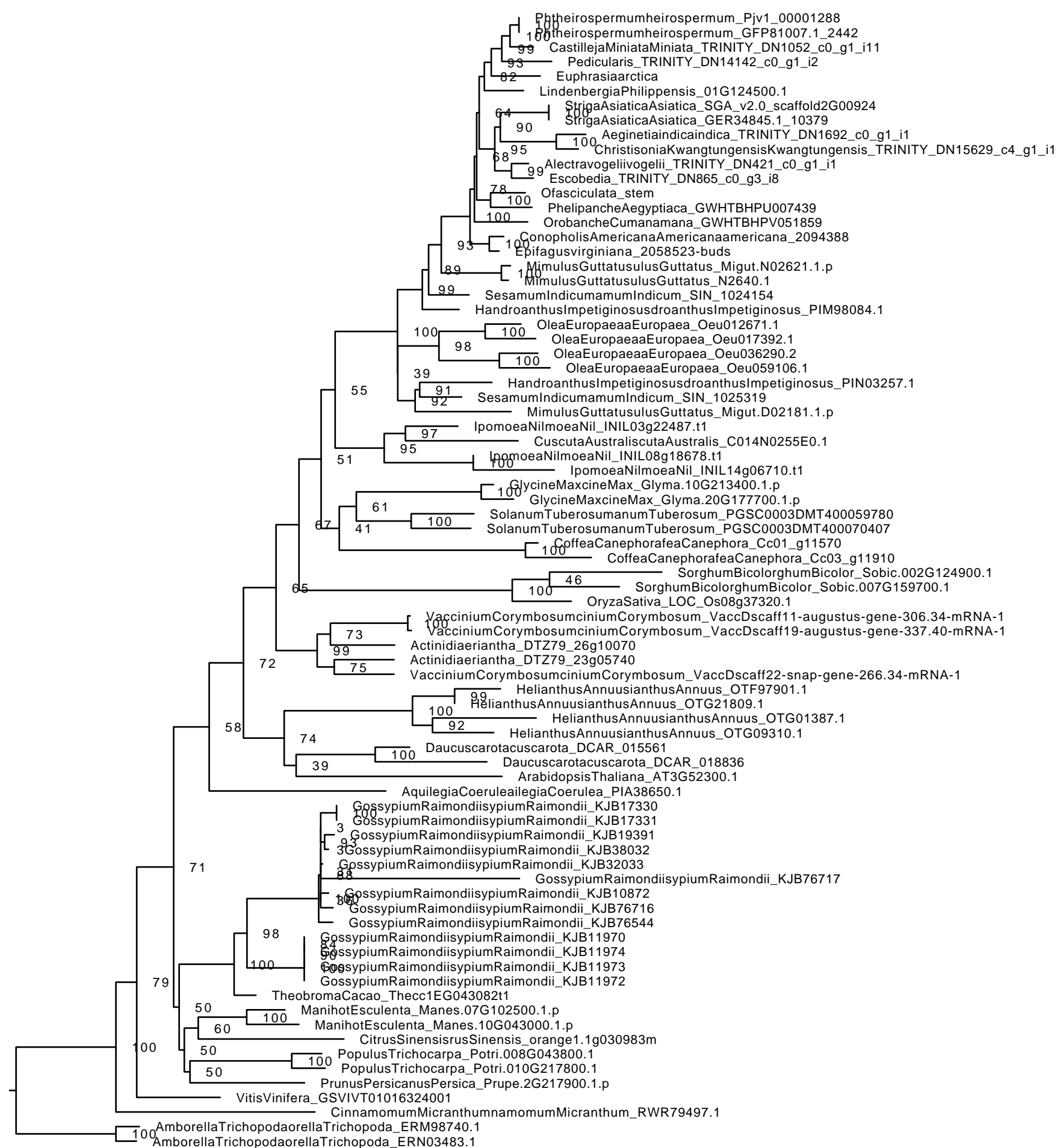

0.08

Supplement: Supplementary file 1 — Appendix S1. [file ECE3-15-e71737-s003.tgz › Orobanchaceae_O2K_Supplementary_v3/Data_S1/DataS1/CV/OG0003300.filter.fas.treefile.pdf]

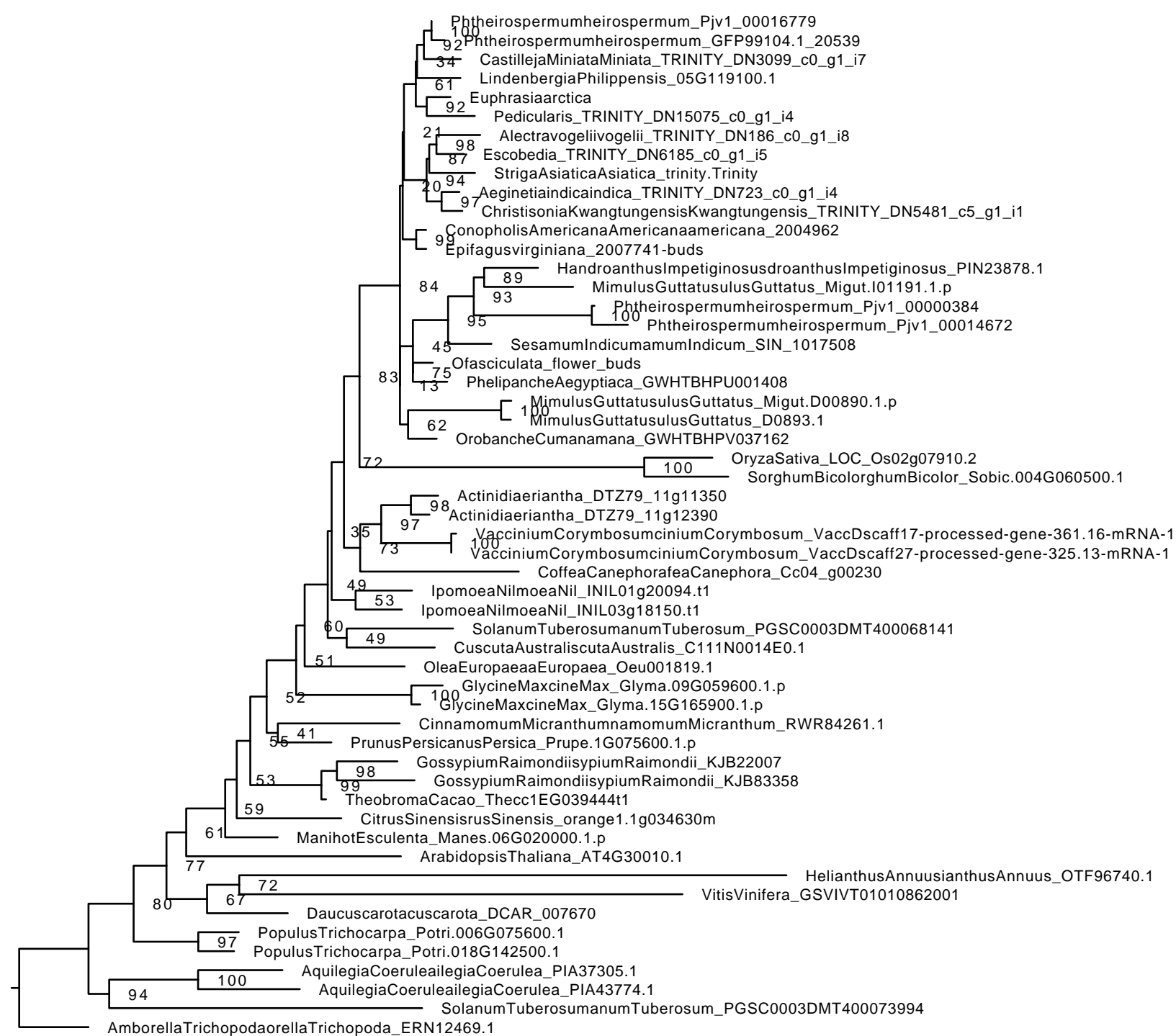

0.2

Supplement: Supplementary file 1 — Appendix S1. [file ECE3-15-e71737-s003.tgz › Orobanchaceae_O2K_Supplementary_v3/Data_S1/DataS1/CV/OG0007933.filter.fas.treefile.pdf]

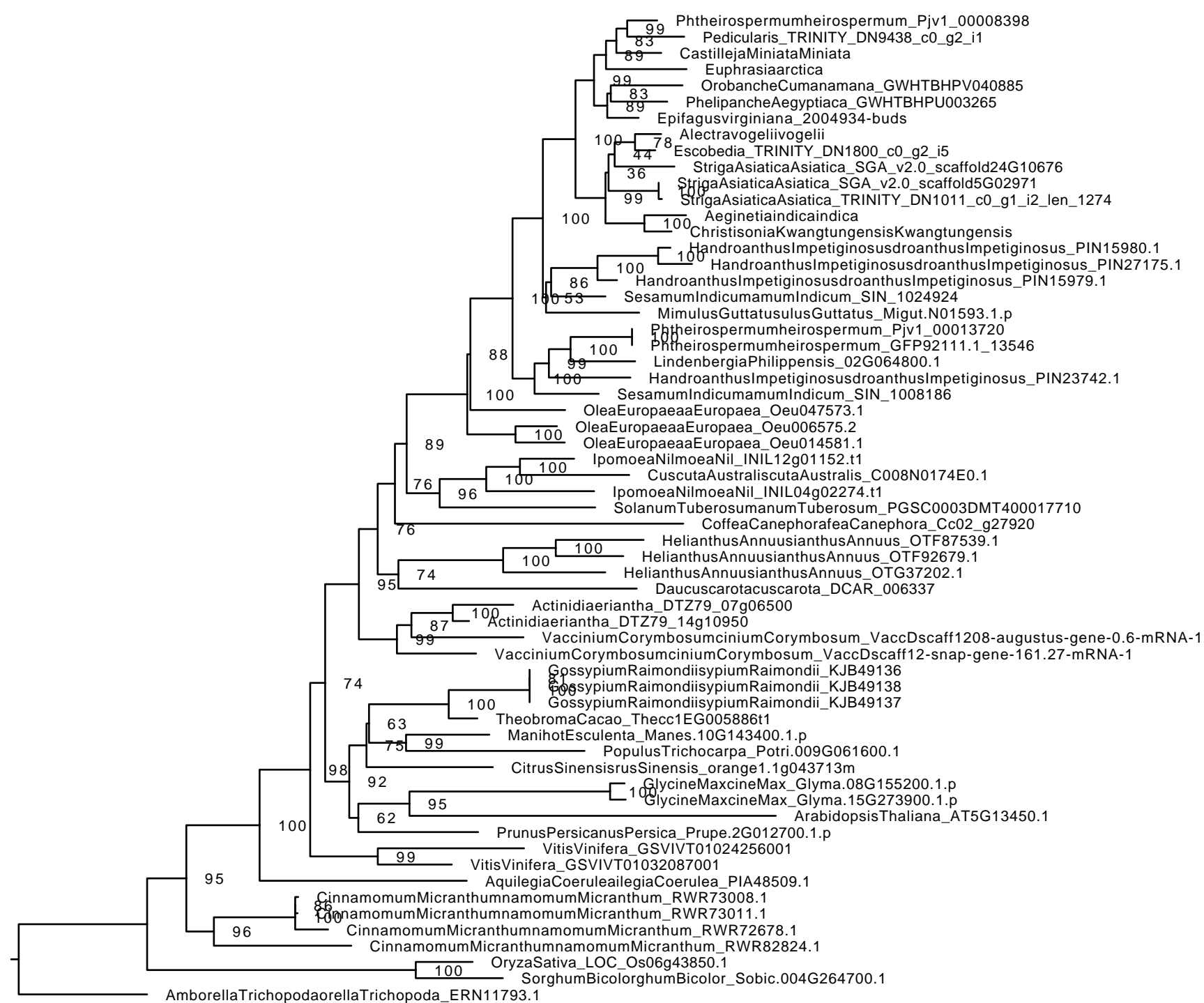

0.08

Supplement: Supplementary file 1 — Appendix S1. [file ECE3-15-e71737-s003.tgz › Orobanchaceae_O2K_Supplementary_v3/Data_S1/DataS1/CV/OG0004639.filter.fas.treefile.pdf]

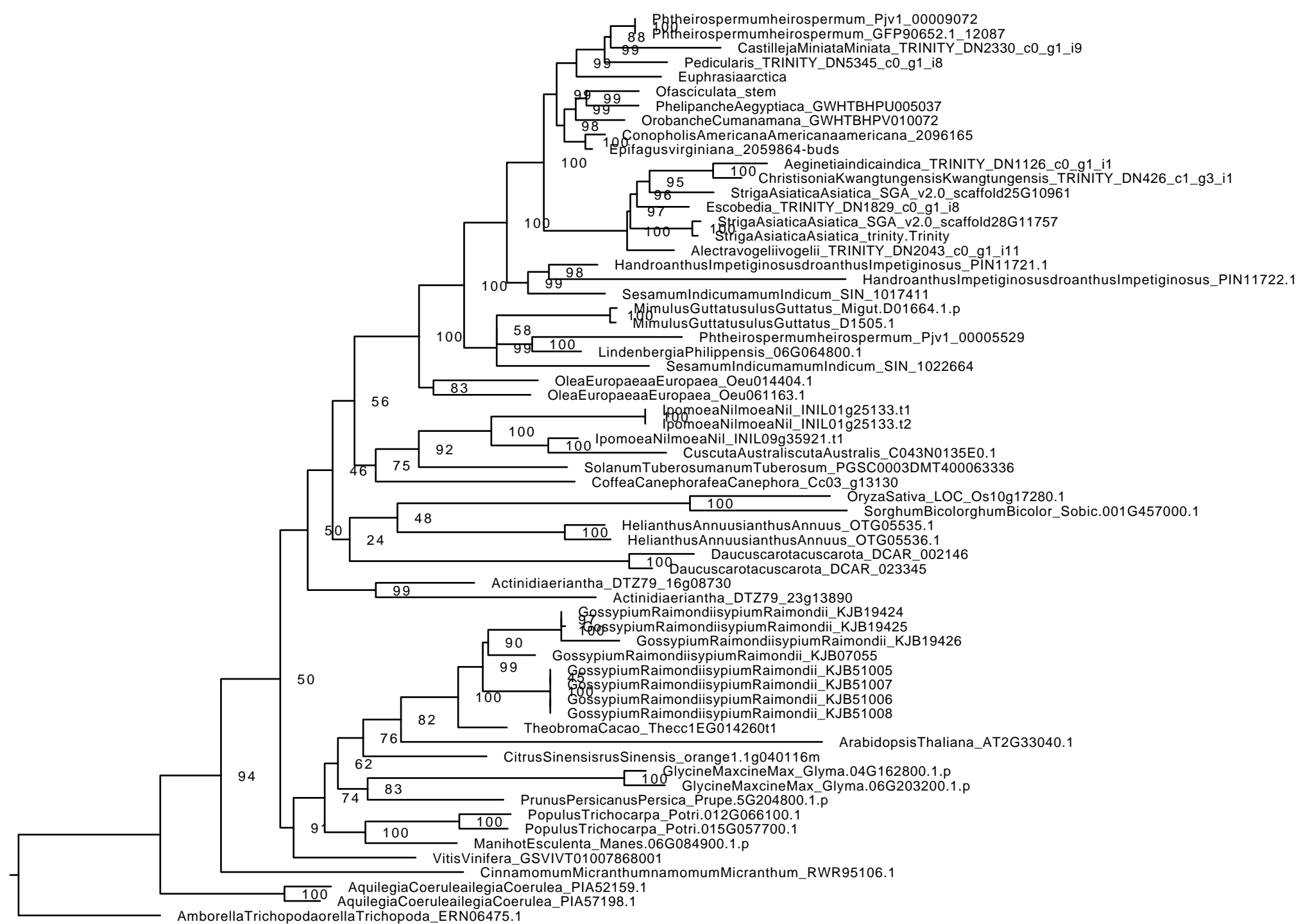

0.06

Supplement: Supplementary file 1 — Appendix S1. [file ECE3-15-e71737-s003.tgz › Orobanchaceae_O2K_Supplementary_v3/Data_S1/DataS1/CV/OG0005413.filter.fas.treefile.pdf]

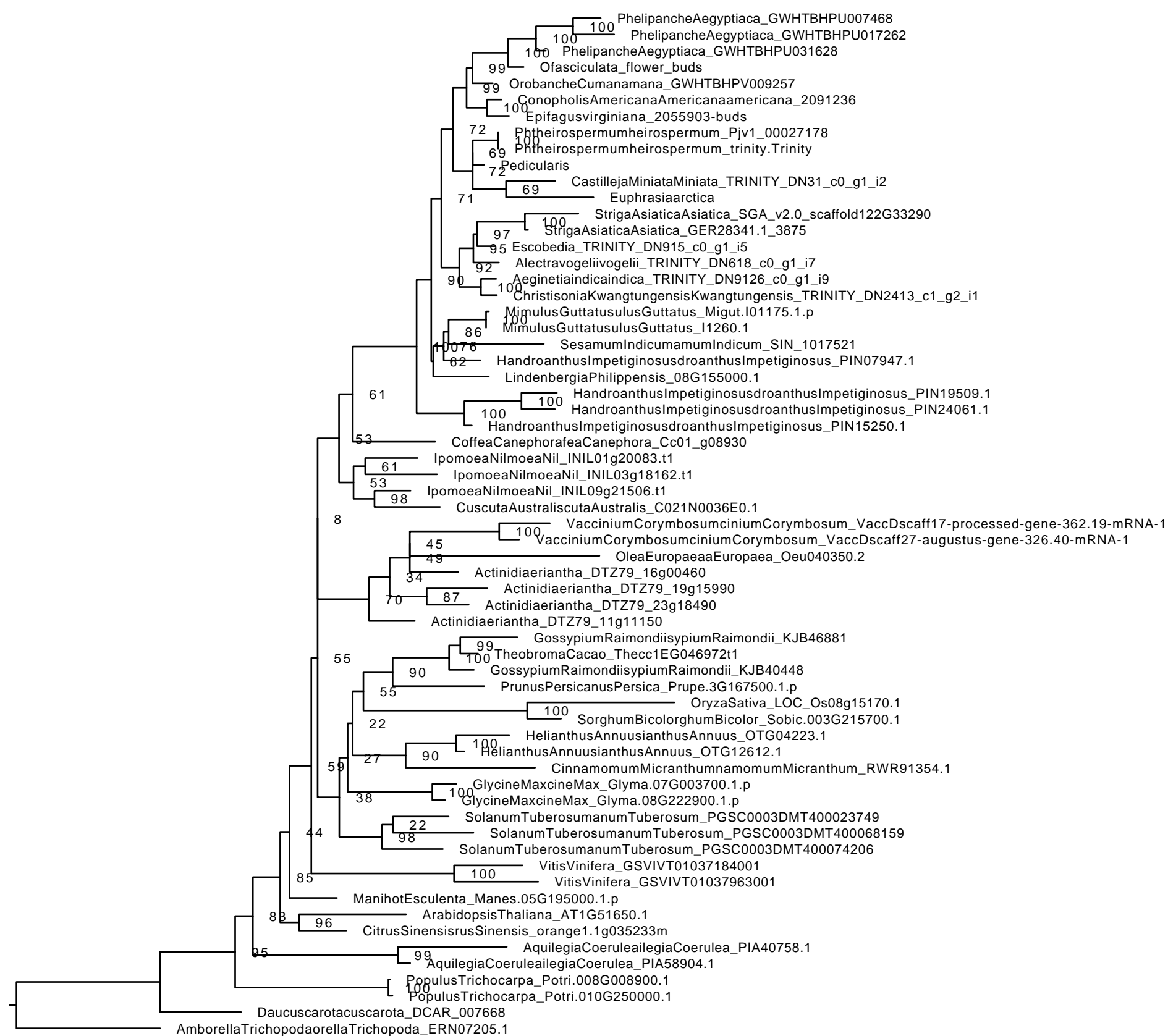

0.2

Supplement: Supplementary file 1 — Appendix S1. [file ECE3-15-e71737-s003.tgz › Orobanchaceae_O2K_Supplementary_v3/Data_S1/DataS1/CV/OG0005039.filter.fas.treefile.pdf]

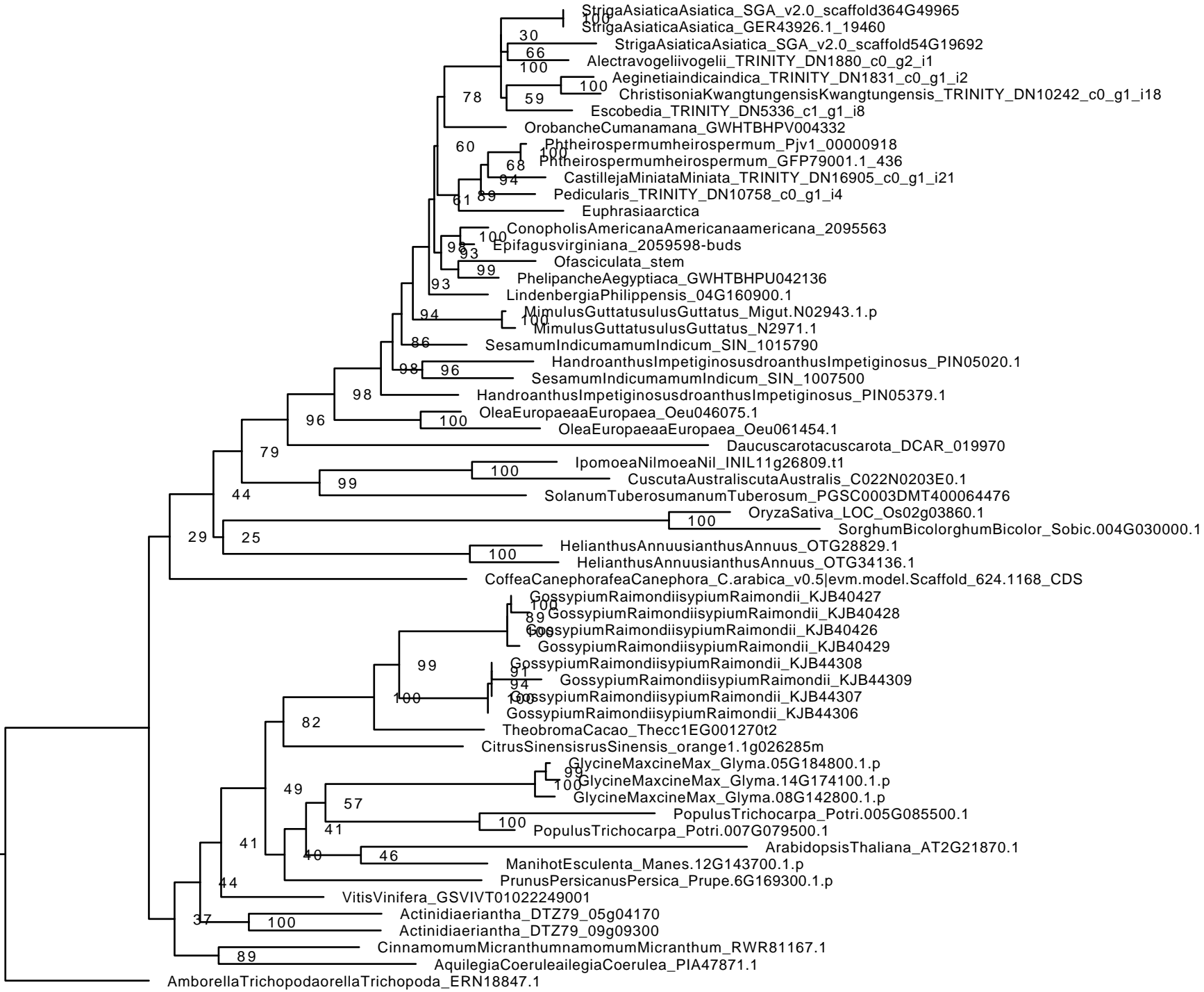

0.05

Supplement: Supplementary file 1 — Appendix S1. [file ECE3-15-e71737-s003.tgz › Orobanchaceae_O2K_Supplementary_v3/Data_S1/DataS1/CV/OG0004876.filter.fas.treefile.pdf]

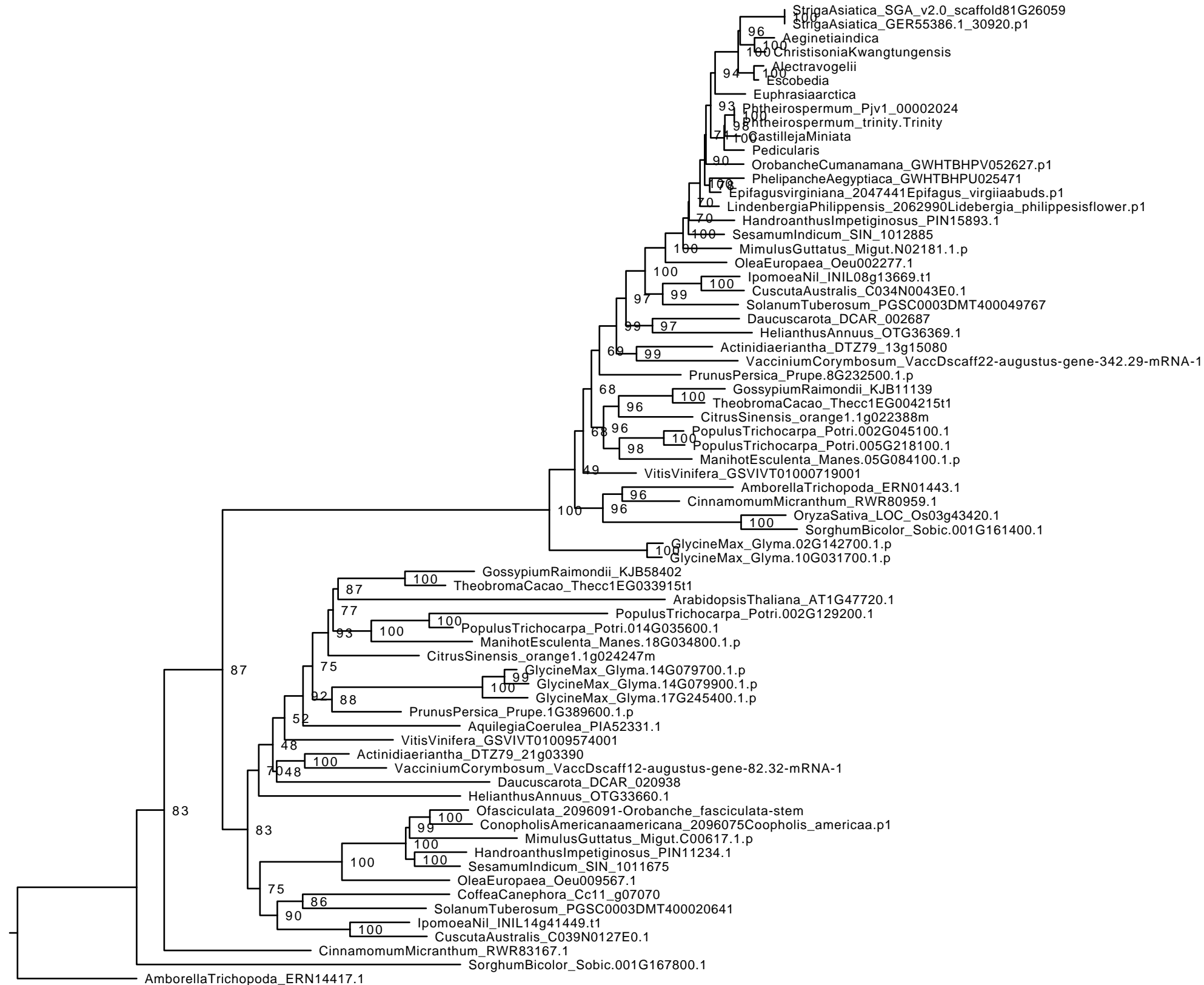

0.3

Supplement: Supplementary file 1 — Appendix S1. [file ECE3-15-e71737-s003.tgz › Orobanchaceae_O2K_Supplementary_v3/Data_S1/DataS1/RRR/OG0003987.filter.fas.treefile.pdf]

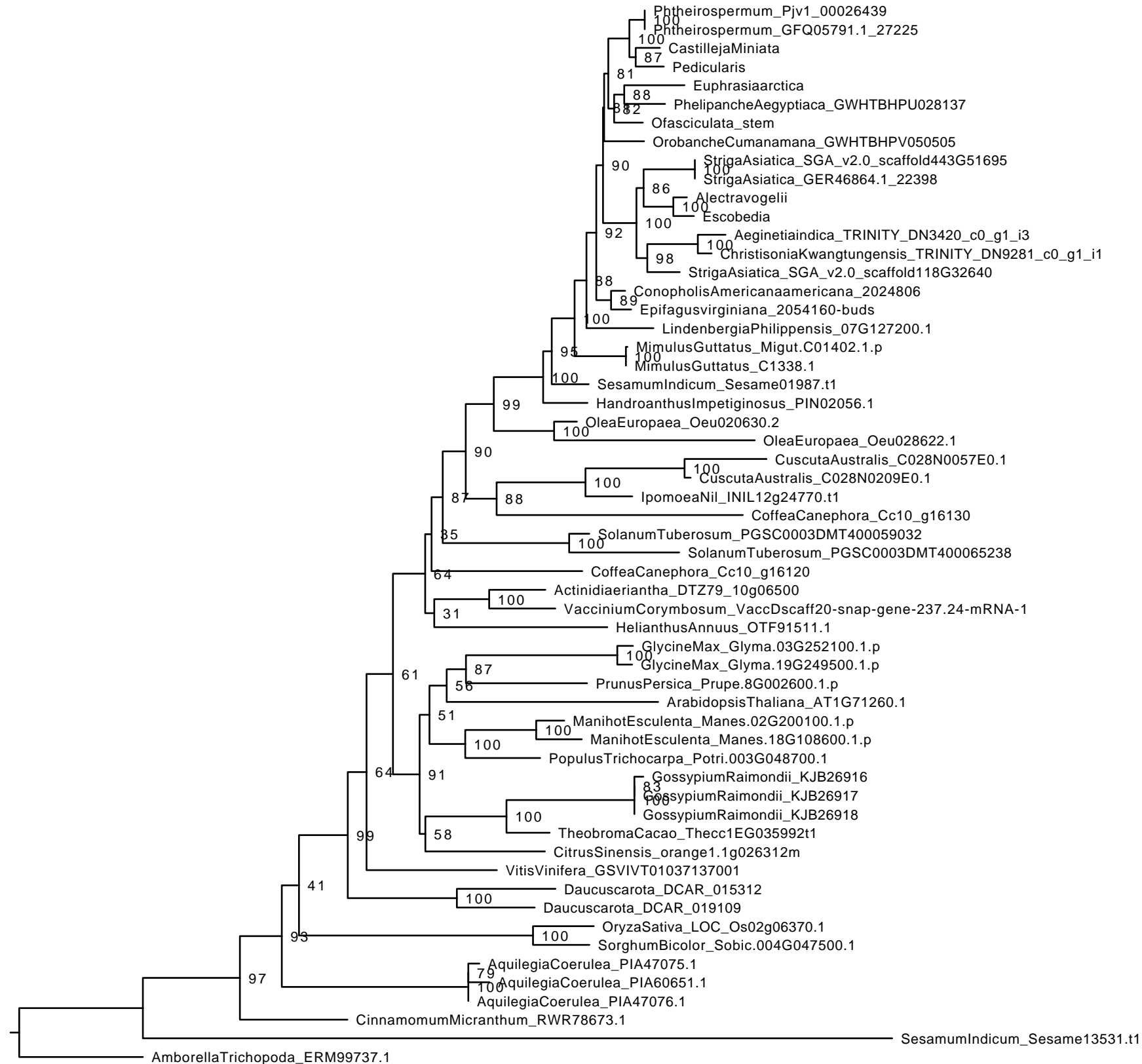

0.2

Supplement: Supplementary file 1 — Appendix S1. [file ECE3-15-e71737-s003.tgz › Orobanchaceae_O2K_Supplementary_v3/Data_S1/DataS1/RRR/OG0007649.filter.fas.treefile.pdf]

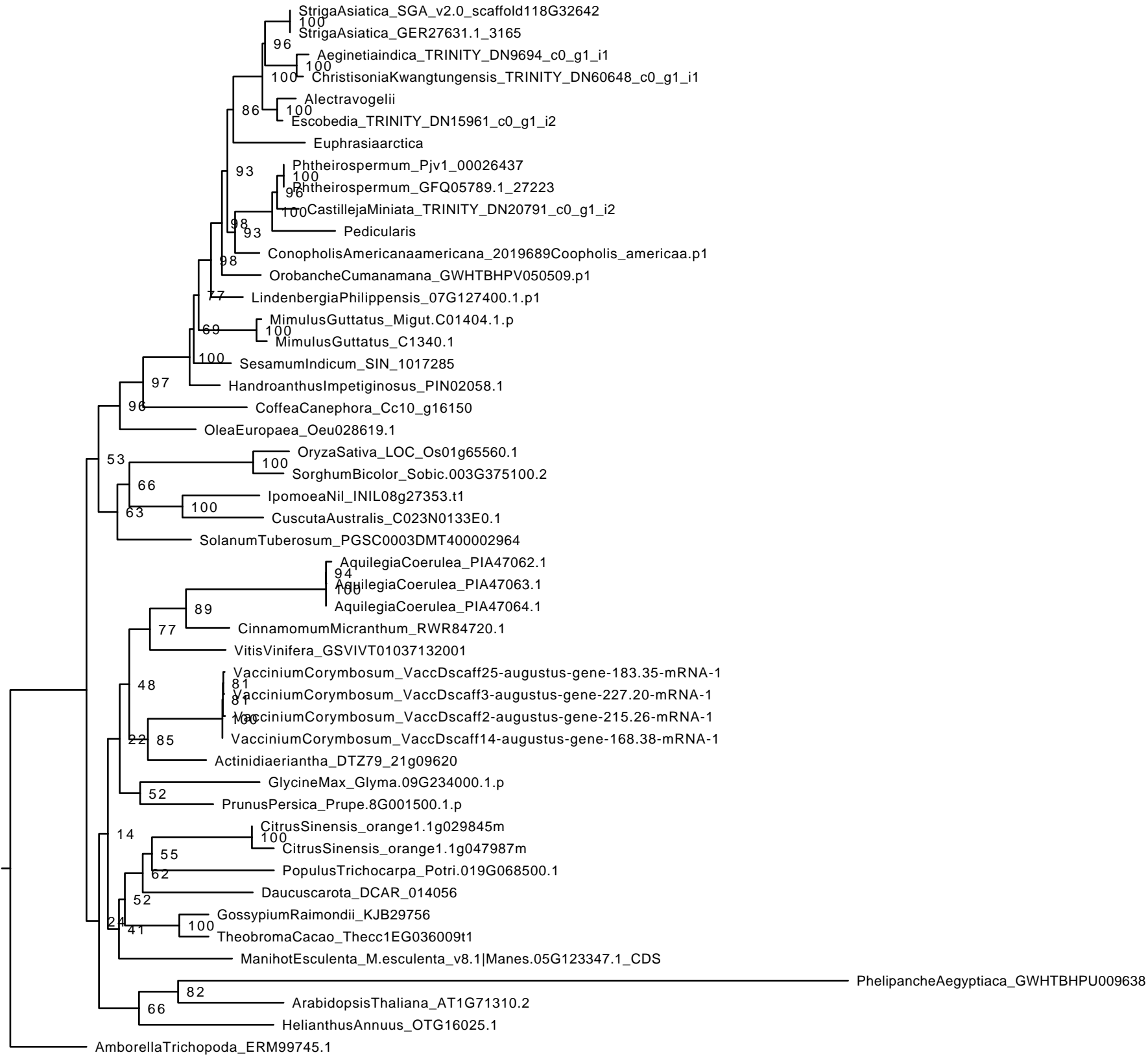

0.3

Supplement: Supplementary file 1 — Appendix S1. [file ECE3-15-e71737-s003.tgz › Orobanchaceae_O2K_Supplementary_v3/Data_S1/DataS1/RRR/OG0010048.filter.fas.treefile.pdf]

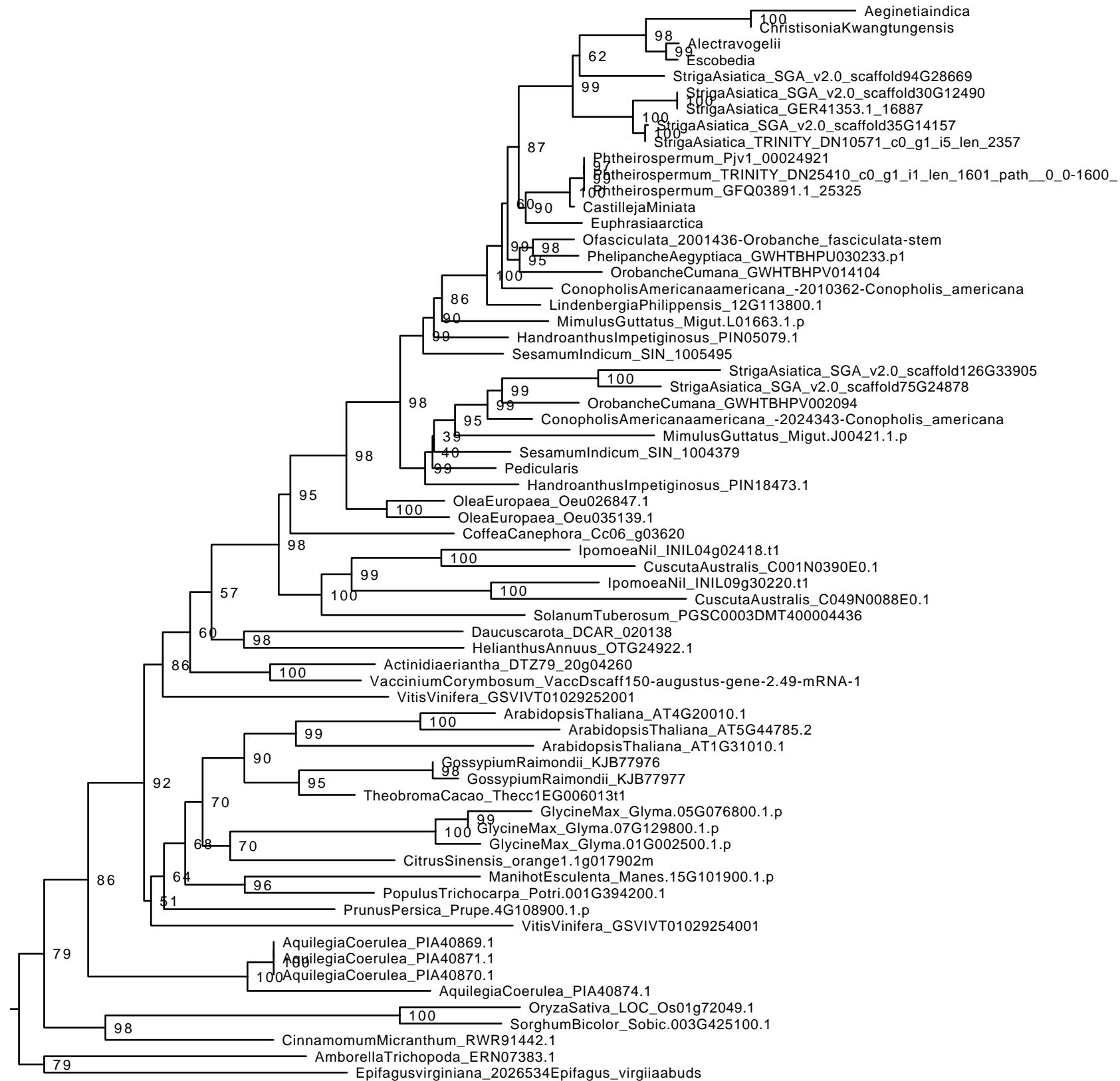

0.3

Supplement: Supplementary file 1 — Appendix S1. [file ECE3-15-e71737-s003.tgz › Orobanchaceae_O2K_Supplementary_v3/Data_S1/DataS1/RRR/OG0005683.filter.fas.treefile.pdf]

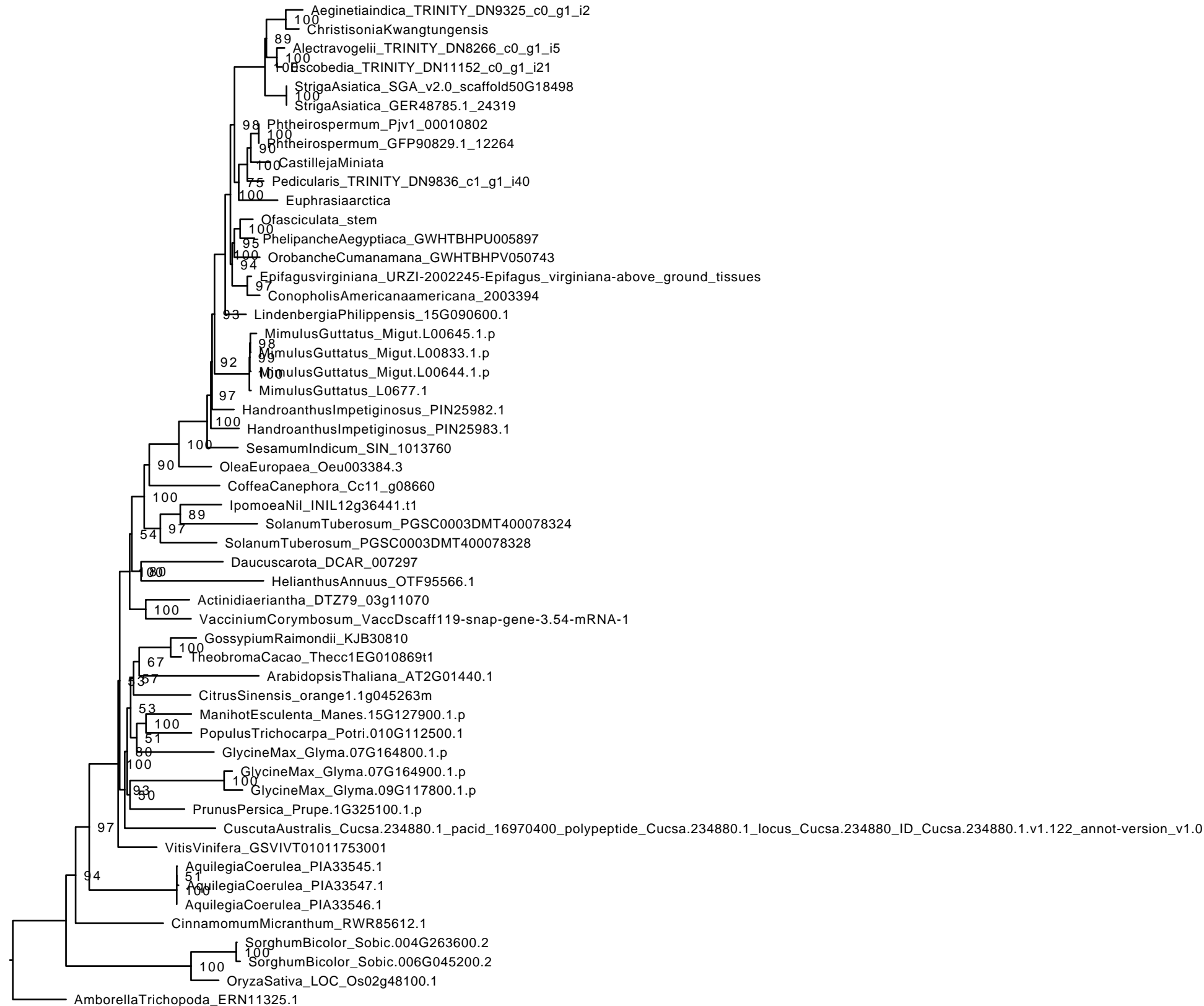

0.2

Supplement: Supplementary file 1 — Appendix S1. [file ECE3-15-e71737-s003.tgz › Orobanchaceae_O2K_Supplementary_v3/Data_S1/DataS1/RRR/OG0007320.filter.fas.treefile.pdf]
