# Supplementary material for: Altered Mitochondrial Respiration Is Associated With Loss of Nuclear‐Encoded OXPHOS Genes in Parasitic Broomrapes
Source: Ecol Evol. 2025 Jul 6;15(7):e71737. doi: 10.1002/ece3.71737 (PMC12230201; doi:10.1002/ece3.71737)
Supplement: Supplementary file 2 — Appendix S2. [file ECE3-15-e71737-s001.docx]

**Supplementary Information**

**Supplementary figures**

**
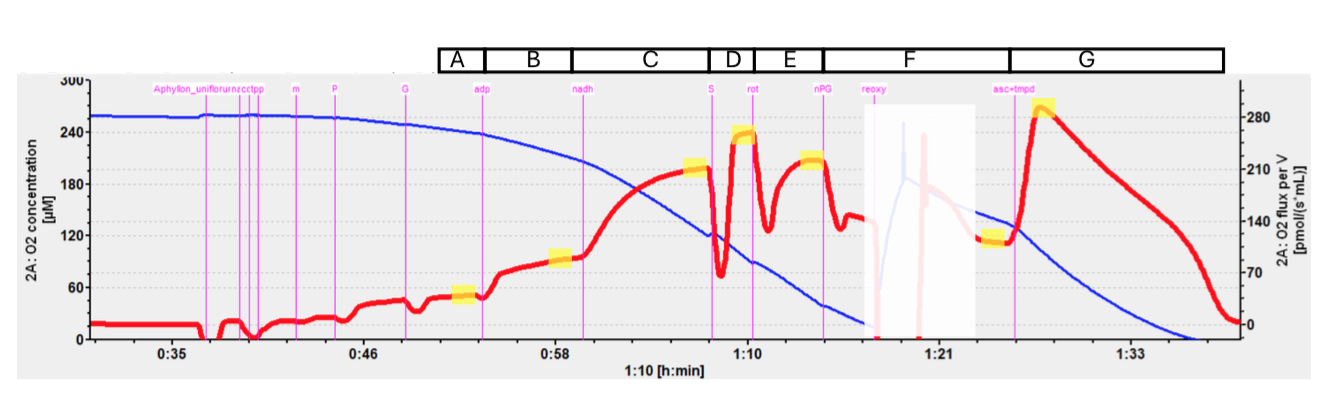
**

**Figure S1** Quantification of mitochondrial respiration using O2K high-resolution respirometry. Data were taken from an *Aphyllon uniflorum* sample that showed typical changes in oxygen concentration (blue line) and oxygen flux (red line) by adding mitochondrial respiratory substrates or inhibitors over a 1.5-hour experiment period. Values of oxygen flux were recorded (highlighted yellow regions) after the addition of substrates or inhibitors (purple labeled text) for steps A-G as defined in Table S3. Reoxygenation events (in white box) were masked to enhance clarity.

**Supplementary tables**

**Table S1** Taxon sampling, data source, and data quality evaluation for the genomic survey of nuclear-encoded mitochondrial genes in angiosperms.

**Table S2** Taxon sampling and collection localities for O2K high-resolution respirometry in Orobanchaceae.

**Table S3** Definition of respiratory flux control factors.

**Table S4** Pairwise sequence divergence to *Arabidopsis thaliana* inferred from concatenated sequence of nuclear-encoded mitochondrial genes*.*

**Table S5** Statistical significance of relaxed or intensified selection of nuclear-encoded mitochondrial proteins in tribe Orobancheae. The omega values (dN/dS) of test branches (Orobancheae) and reference branches are reported for individual genes as well as the concatenated matrix for each protein complex. Significantly relaxed selection is highlighted in orange and significantly intensified selection is highlighted in blue.

**Table S6** Pairwise correlation between selected OXPHOS FCFs under the linear mixed-effects models.

**Supplementary Notes**

**Note S1** Plant mitochondrial isolation and O2K protocol

**Supplementary data**

**Data S1** DNA codon alignments and maximum likelihood phylogeny for nuclear-encoded mitochondrial genes in angiosperm.

**Data S2** Raw oxygen flux and calculated flux control factors from fifty runs of O2K high-resolution respirometry in Orobanchaceae.
