# Supplementary material for: Altered Mitochondrial Respiration Is Associated With Loss of Nuclear‐Encoded OXPHOS Genes in Parasitic Broomrapes
Source: Ecol Evol. 2025 Jul 6;15(7):e71737. doi: 10.1002/ece3.71737 (PMC12230201; doi:10.1002/ece3.71737)
Supplement: Supplementary file 1 — Appendix S1. [file ECE3-15-e71737-s003.tgz › Orobanchaceae_O2K_Supplementary_v3/Data_S1/DataS1/RRR/OG0001482_RECA3.filter.fas.treefile.pdf]

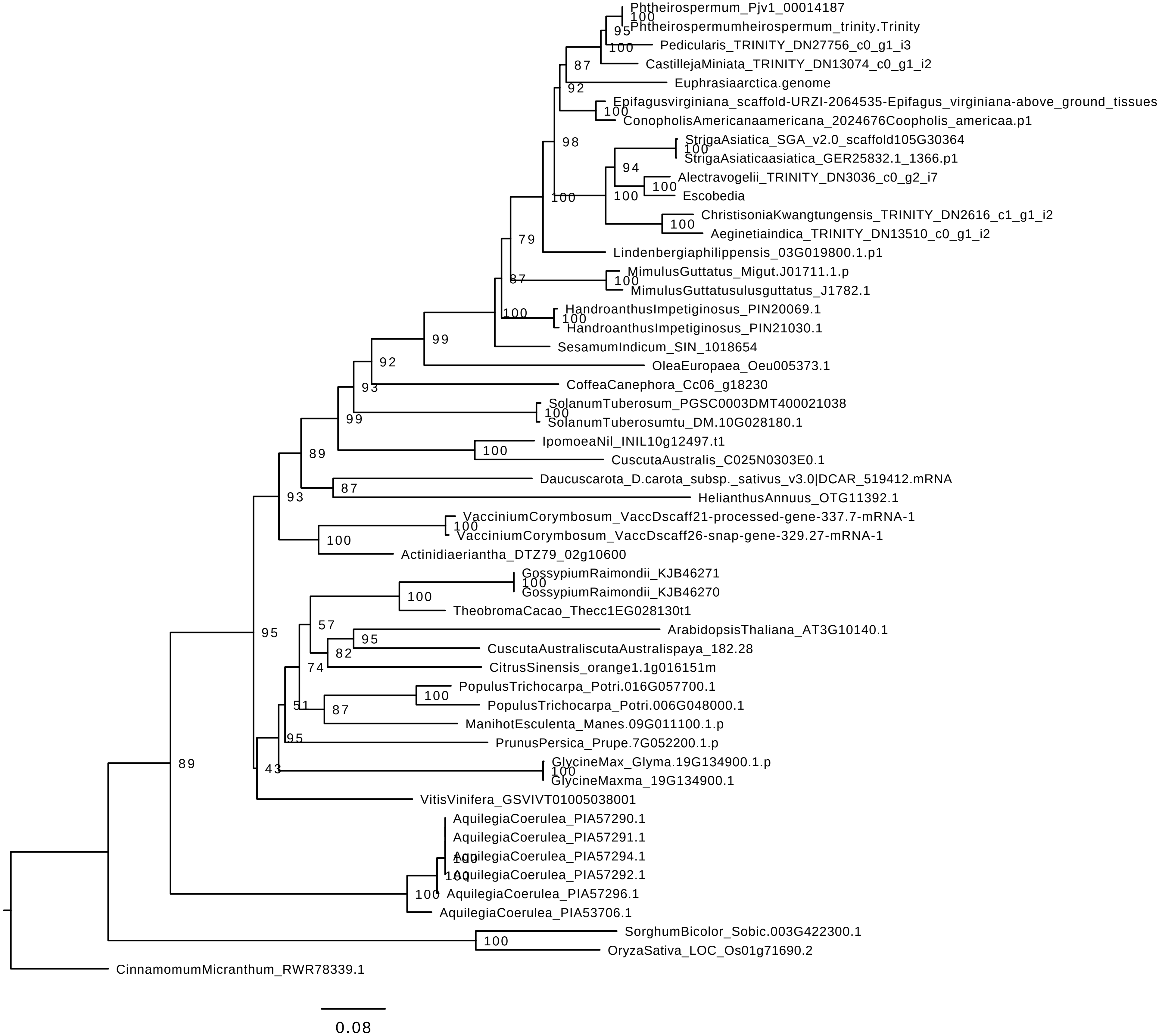
Phtheirospermum_Pjv1_00014187
Phtheirospermumheirospermum_trinity.Trinity
Pedicularis_TRINITY_DN27756_c0_g1_i3
CastillejaMiniata_TRINITY_DN13074_c0_g1_i2
Euphrasiaarctica.genome
Epifagusvirginiana_scaffold-URZI-2064535-Epifagus_virginiana-above_ground_tissues
ConopholisAmericanaamericana_2024676Coopholis_americaa.p1
StrigaAsiatica_SGA_v2.0_scaffold105G30364
StrigaAsiaticaasiatica_GER25832.1_1366.p1
Alectravogelii_TRINITY_DN3036_c0_g2_i7
Escobedia
ChristisoniaKwangtungensis_TRINITY_DN2616_c1_g1_i2
Aeginetiaindica_TRINITY_DN13510_c0_g1_i2
Lindenbergiaphilippensis_03G019800.1.p1
MimulusGuttatus_Migut.J01711.1.p
MimulusGuttatusulusguttatus_J1782.1
HandroanthusImpetiginosus_PIN20069.1
HandroanthusImpetiginosus_PIN21030.1
SesamumIndicum_SIN_1018654
OleaEuropaea_Oeu005373.1
CoffeaCanephora_Cc06_g18230
SolanumTuberosum_PGSC0003DMT400021038
SolanumTuberosumtu_DM.10G028180.1
IpomoeaNil_INIL10g12497.t1
CuscutaAustralis_C025N0303E0.1
Daucuscarota_D.carota_subsp._sativus_v3.0|DCAR_519412.mRNA
HelianthusAnnuus_OTG11392.1
VacciniumCorymbosum_VaccDscaff21-processed-gene-337.7-mRNA-1
VacciniumCorymbosum_VaccDscaff26-snap-gene-329.27-mRNA-1
Actinidiaeriantha_DTZ79_02g10600
GossypiumRaimondii_KJB46271
GossypiumRaimondii_KJB46270
TheobromaCacao_Thecc1EG028130t1
ArabidopsisThaliana_AT3G10140.1
CuscutaAustraliscutaAustralispaya_182.28
CitrusSinensis_orange1.1g016151m
PopulusTrichocarpa_Potri.016G057700.1
PopulusTrichocarpa_Potri.006G048000.1
ManihotEsculenta_Manes.09G011100.1.p
PrunusPersica_Prupe.7G052200.1.p
GlycineMax_Glyma.19G134900.1.p
GlycineMaxma_19G134900.1
VitisVinifera_GSVIVT01005038001
AquilegiaCoerulea_PIA57290.1
AquilegiaCoerulea_PIA57291.1
AquilegiaCoerulea_PIA57294.1
AquilegiaCoerulea_PIA57292.1
AquilegiaCoerulea_PIA57296.1
AquilegiaCoerulea_PIA53706.1
SorghumBicolor_Sobic.003G422300.1
OryzaSativa_LOC_Os01g71690.2
CinnamomumMicranthum_RWR78339.1
0.08
