# Supplementary material for: Altered Mitochondrial Respiration Is Associated With Loss of Nuclear‐Encoded OXPHOS Genes in Parasitic Broomrapes
Source: Ecol Evol. 2025 Jul 6;15(7):e71737. doi: 10.1002/ece3.71737 (PMC12230201; doi:10.1002/ece3.71737)
Supplement: Supplementary file 1 — Appendix S1. [file ECE3-15-e71737-s003.tgz › Orobanchaceae_O2K_Supplementary_v3/Data_S1/DataS1/RRR/OG0009767.filter.fas.treefile.pdf]

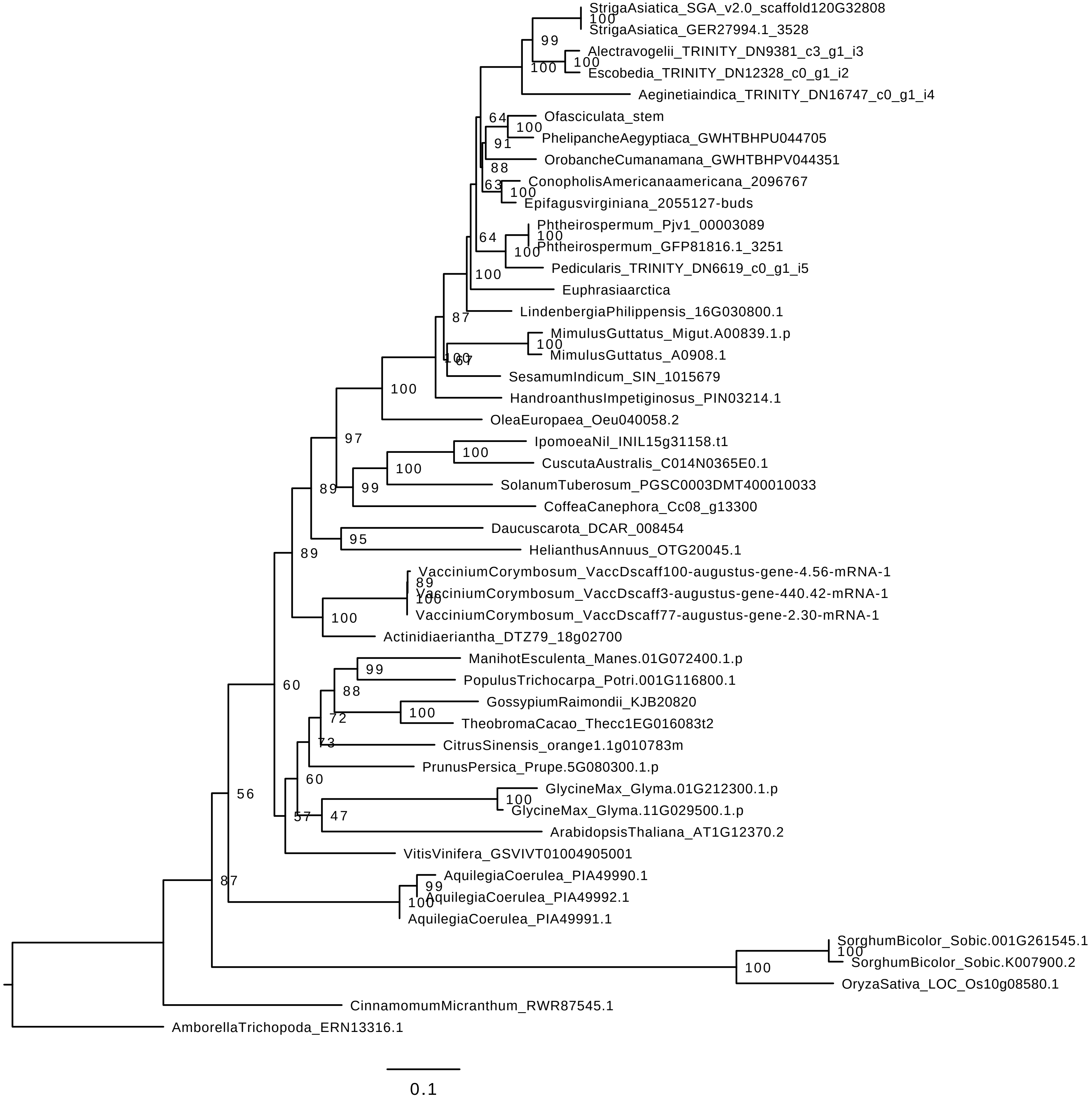
StrigaAsiatica_SGA_v2.0_scaffold120G32808
100
StrigaAsiatica_GER27994.1_3528
99
Alectravogelii_TRINITY_DN9381_c3_g1_i3
100
100
Escobedia_TRINITY_DN12328_c0_g1_i2
Aeginetiaindica_TRINITY_DN16747_c0_g1_i4
64
Ofasciculata_stem
100
PhelipancheAegyptiaca_GWHTBHPU044705
91
OrobancheCumanamana_GWHTBHPV044351
88
63
ConopholisAmericanaamericana_2096767
100
Epifagusvirginiana_2055127-buds
Phtheirospermum_Pjv1_00003089
100
64
Phtheirospermum_GFP81816.1_3251
100
Pedicularis_TRINITY_DN6619_c0_g1_i5
100
Euphrasiaarctica
LindenbergiaPhilippensis_16G030800.1
87
MimulusGuttatus_Migut.A00839.1.p
100
100
67
MimulusGuttatus_A0908.1
SesamumIndicum_SIN_1015679
HandroanthusImpetiginosus_PIN03214.1
100
OleaEuropaea_Oeu040058.2
97
IpomoeaNil_INIL15g31158.t1
100
CuscutaAustralis_C014N0365E0.1
100
89
99
SolanumTuberosum_PGSC0003DMT400010033
CoffeaCanephora_Cc08_g13300
Daucuscarota_DCAR_008454
95
89
HelianthusAnnuus_OTG20045.1
VacciniumCorymbosum_VaccDscaff100-augustus-gene-4.56-mRNA-1
89
VacciniumCorymbosum_VaccDscaff3-augustus-gene-440.42-mRNA-1
100
100
VacciniumCorymbosum_VaccDscaff77-augustus-gene-2.30-mRNA-1
Actinidiaeriantha_DTZ79_18g02700
ManihotEsculenta_Manes.01G072400.1.p
99
60
PopulusTrichocarpa_Potri.001G116800.1
88
GossypiumRaimondii_KJB20820
72
100
TheobromaCacao_Thecc1EG016083t2
73
CitrusSinensis_orange1.1g010783m
PrunusPersica_Prupe.5G080300.1.p
56
60
GlycineMax_Glyma.01G212300.1.p
100
57
47
GlycineMax_Glyma.11G029500.1.p
ArabidopsisThaliana_AT1G12370.2
VitisVinifera_GSVIVT01004905001
87
AquilegiaCoerulea_PIA49990.1
99
100
AquilegiaCoerulea_PIA49992.1
AquilegiaCoerulea_PIA49991.1
SorghumBicolor_Sobic.001G261545.1
100
100
SorghumBicolor_Sobic.K007900.2
OryzaSativa_LOC_Os10g08580.1
CinnamomumMicranthum_RWR87545.1
AmborellaTrichopoda_ERN13316.1
0.1
