# Supplementary material for: Altered Mitochondrial Respiration Is Associated With Loss of Nuclear‐Encoded OXPHOS Genes in Parasitic Broomrapes
Source: Ecol Evol. 2025 Jul 6;15(7):e71737. doi: 10.1002/ece3.71737 (PMC12230201; doi:10.1002/ece3.71737)
Supplement: Supplementary file 1 — Appendix S1. [file ECE3-15-e71737-s003.tgz › Orobanchaceae_O2K_Supplementary_v3/Data_S1/DataS1/RRR/OG0006862.filter.fas.treefile.pdf]

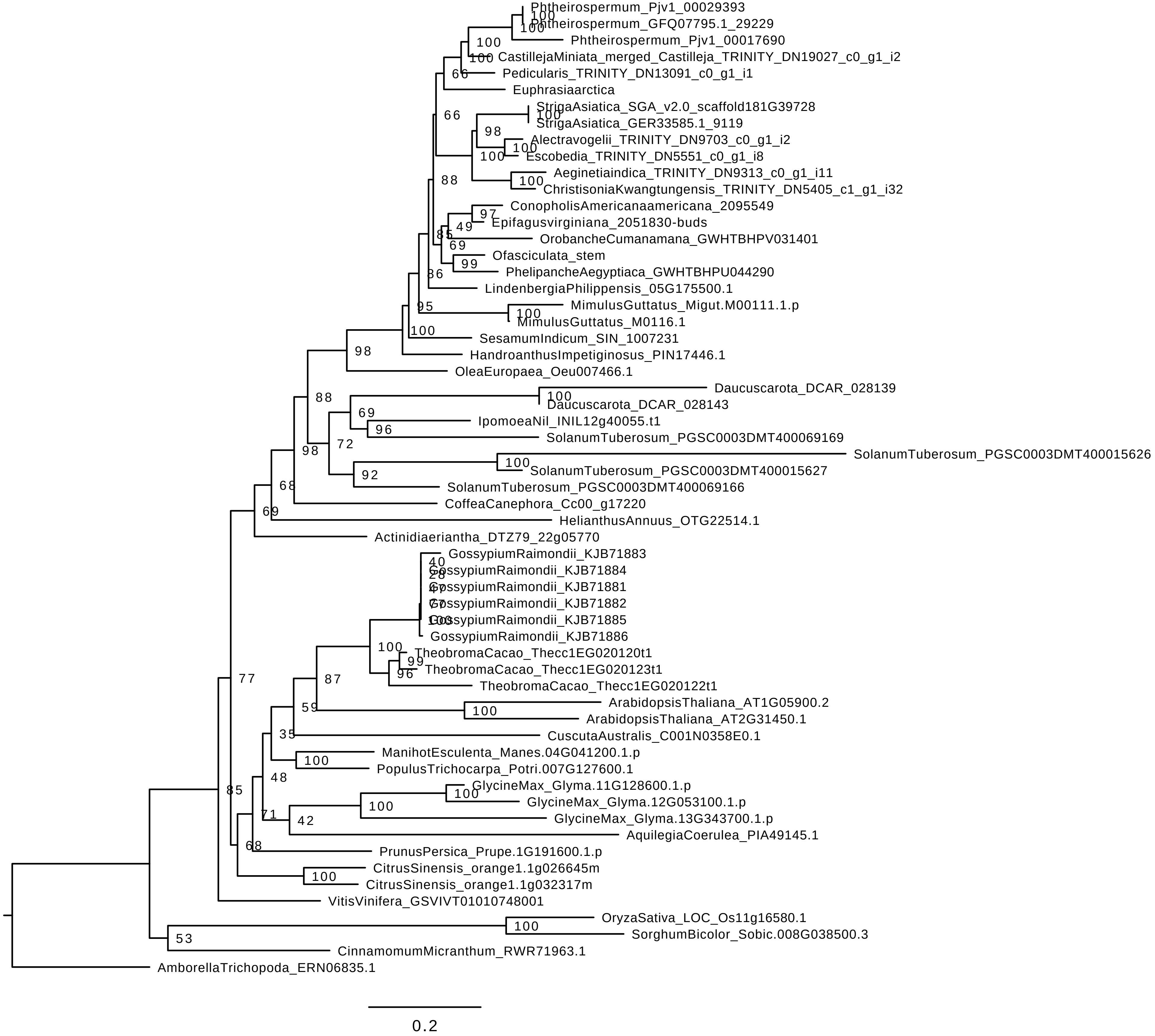

Phtheirospermum_Pjv1_00029393
Phtheirospermum_GFQ07795.1_29229
Phtheirospermum_Pjv1_00017690
CastillejaMiniata_merged_Castilleja_TRINITY_DN19027_c0_g1_i2
Pedicularis_TRINITY_DN13091_c0_g1_i1
Euphrasiaarctica
StrigaAsiatica_SGA_v2.0_scaffold181G39728
StrigaAsiatica_GER33585.1_9119
Alectravogelii_TRINITY_DN9703_c0_g1_i2
Escobedia_TRINITY_DN5551_c0_g1_i8
Aeginetiaindica_TRINITY_DN9313_c0_g1_i11
ChristisoniaKwangtungensis_TRINITY_DN5405_c1_g1_i32
ConopholisAmericanaamericana_2095549
Epifagusvirginiana_2051830-buds
OrobancheCumanamana_GWHTBHPV031401
Ofasciculata_stem
PhelipancheAegyptiaca_GWHTBHPU044290
LindenbergiaPhilippensis_05G175500.1
MimulusGuttatus_Migut.M00111.1.p
MimulusGuttatus_M0116.1
SesamumIndicum_SIN_1007231
HandroanthusImpetiginosus_PIN17446.1
OleaEuropaea_Oeu007466.1
Daucuscarota_DCAR_028139
Daucuscarota_DCAR_028143
IpomoeaNil_INIL12g40055.t1
SolanumTuberosum_PGSC0003DMT400069169
SolanumTuberosum_PGSC0003DMT400015626
SolanumTuberosum_PGSC0003DMT400015627
SolanumTuberosum_PGSC0003DMT400069166
CoffeaCanephora_Cc00_g17220
HelianthusAnnuus_OTG22514.1
Actinidiaeriantha_DTZ79_22g05770
GossypiumRaimondii_KJB71883
GossypiumRaimondii_KJB71884
GossypiumRaimondii_KJB71881
GossypiumRaimondii_KJB71882
GossypiumRaimondii_KJB71885
GossypiumRaimondii_KJB71886
TheobromaCacao_Thecc1EG020120t1
TheobromaCacao_Thecc1EG020123t1
TheobromaCacao_Thecc1EG020122t1
ArabidopsisThaliana_AT1G05900.2
ArabidopsisThaliana_AT2G31450.1
CuscutaAustralis_C001N0358E0.1
ManihotEsculenta_Manes.04G041200.1.p
PopulusTrichocarpa_Potri.007G127600.1
GlycineMax_Glyma.11G128600.1.p
GlycineMax_Glyma.12G053100.1.p
GlycineMax_Glyma.13G343700.1.p
AquilegiaCoerulea_PIA49145.1
PrunusPersica_Prupe.1G191600.1.p
CitrusSinensis_orange1.1g026645m
CitrusSinensis_orange1.1g032317m
VitisVinifera_GSVIVT01010748001
OryzaSativa_LOC_Os11g16580.1
SorghumBicolor_Sobic.008G038500.3
CinnamomumMicranthum_RWR71963.1
AmborellaTrichopoda_ERN06835.1
0.2
