# Supplementary material for: Altered Mitochondrial Respiration Is Associated With Loss of Nuclear‐Encoded OXPHOS Genes in Parasitic Broomrapes
Source: Ecol Evol. 2025 Jul 6;15(7):e71737. doi: 10.1002/ece3.71737 (PMC12230201; doi:10.1002/ece3.71737)
Supplement: Supplementary file 1 — Appendix S1. [file ECE3-15-e71737-s003.tgz › Orobanchaceae_O2K_Supplementary_v3/Data_S1/DataS1/RRR/OG0007566.filter.fas.treefile.pdf]

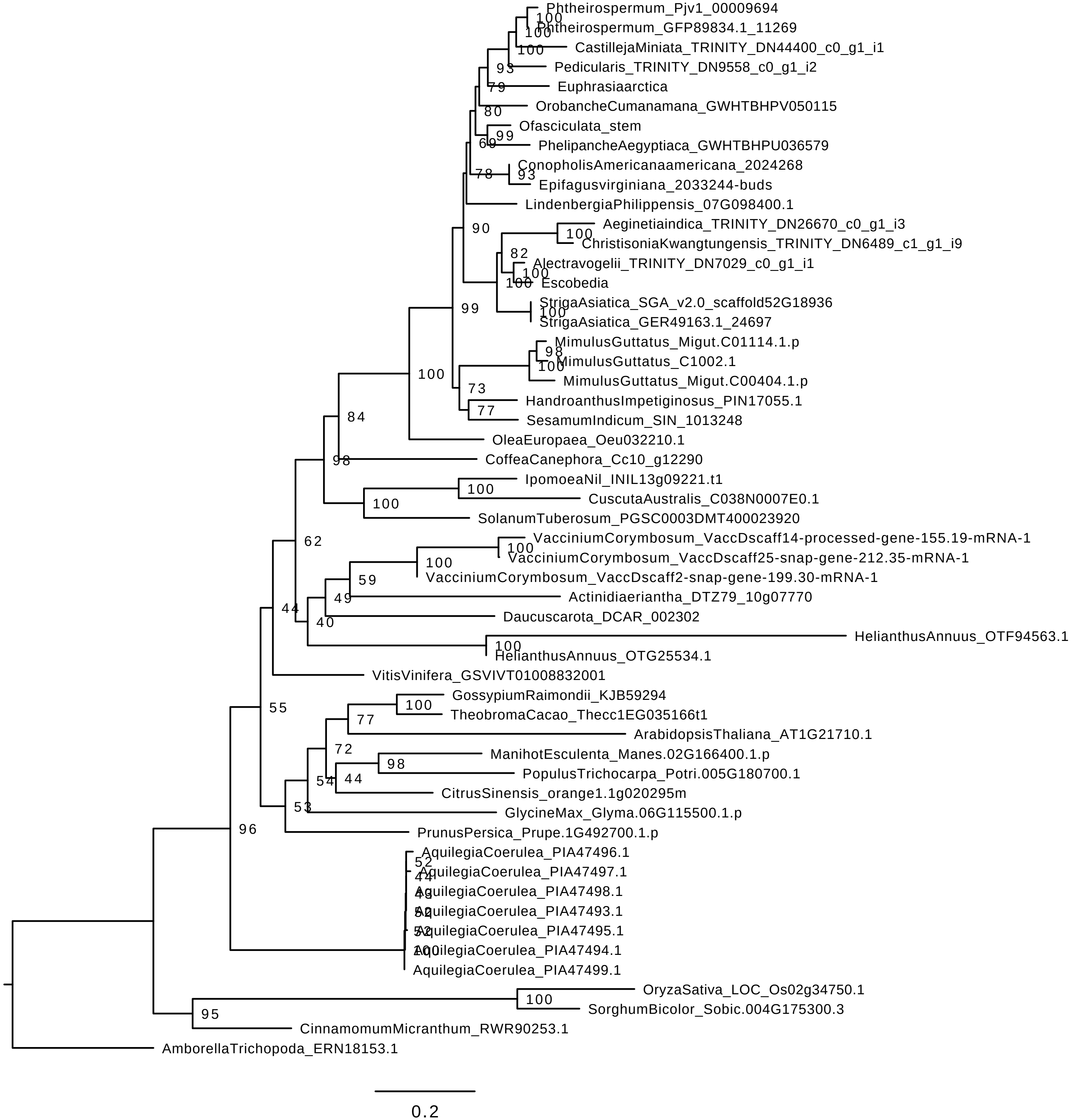
Phtheirospermum_Pjv1_00009694
Phtheirospermum_GFP89834.1_11269
CastillejaMiniata_TRINITY_DN44400_c0_g1_i1
Pedicularis_TRINITY_DN9558_c0_g1_i2
Euphrasiaarctica
OrobancheCumanamana_GWHTBHPV050115
Ofasciculata_stem
PhelipancheAegyptiaca_GWHTBHPU036579
ConopholisAmericanaamericana_2024268
Epifagusvirginiana_2033244-buds
LindenbergiaPhilippensis_07G098400.1
Aeginetiaindica_TRINITY_DN26670_c0_g1_i3
ChristisoniaKwangtungensis_TRINITY_DN6489_c1_g1_i9
Alectravogelii_TRINITY_DN7029_c0_g1_i1
Escobedia
StrigaAsiatica_SGA_v2.0_scaffold52G18936
StrigaAsiatica_GER49163.1_24697
MimulusGuttatus_Migut.C01114.1.p
MimulusGuttatus_C1002.1
MimulusGuttatus_Migut.C00404.1.p
HandroanthusImpetiginosus_PIN17055.1
SesamumIndicum_SIN_1013248
OleaEuropaea_Oeu032210.1
CoffeaCanephora_Cc10_g12290
IpomoeaNil_INIL13g09221.t1
CuscutaAustralis_C038N0007E0.1
SolanumTuberosum_PGSC0003DMT400023920
VacciniumCorymbosum_VaccDscaff14-processed-gene-155.19-mRNA-1
VacciniumCorymbosum_VaccDscaff25-snap-gene-212.35-mRNA-1
VacciniumCorymbosum_VaccDscaff2-snap-gene-199.30-mRNA-1
Actinidiaeriantha_DTZ79_10g07770
Daucuscarota_DCAR_002302
HelianthusAnnuus_OTF94563.1
HelianthusAnnuus_OTG25534.1
VitisVinifera_GSVIVT01008832001
GossypiumRaimondii_KJB59294
TheobromaCacao_Thecc1EG035166t1
ArabidopsisThaliana_AT1G21710.1
ManihotEsculenta_Manes.02G166400.1.p
PopulusTrichocarpa_Potri.005G180700.1
CitrusSinensis_orange1.1g020295m
GlycineMax_Glyma.06G115500.1.p
PrunusPersica_Prupe.1G492700.1.p
AquilegiaCoerulea_PIA47496.1
AquilegiaCoerulea_PIA47497.1
AquilegiaCoerulea_PIA47498.1
AquilegiaCoerulea_PIA47493.1
AquilegiaCoerulea_PIA47495.1
AquilegiaCoerulea_PIA47494.1
AquilegiaCoerulea_PIA47499.1
OryzaSativa_LOC_Os02g34750.1
SorghumBicolor_Sobic.004G175300.3
CinnamomumMicranthum_RWR90253.1
AmborellaTrichopoda_ERN18153.1
0.2
