# Supplementary material for: Altered Mitochondrial Respiration Is Associated With Loss of Nuclear‐Encoded OXPHOS Genes in Parasitic Broomrapes
Source: Ecol Evol. 2025 Jul 6;15(7):e71737. doi: 10.1002/ece3.71737 (PMC12230201; doi:10.1002/ece3.71737)
Supplement: Supplementary file 1 — Appendix S1. [file ECE3-15-e71737-s003.tgz › Orobanchaceae_O2K_Supplementary_v3/Data_S1/DataS1/RRR/OG0006606.filter.fas.treefile.pdf]

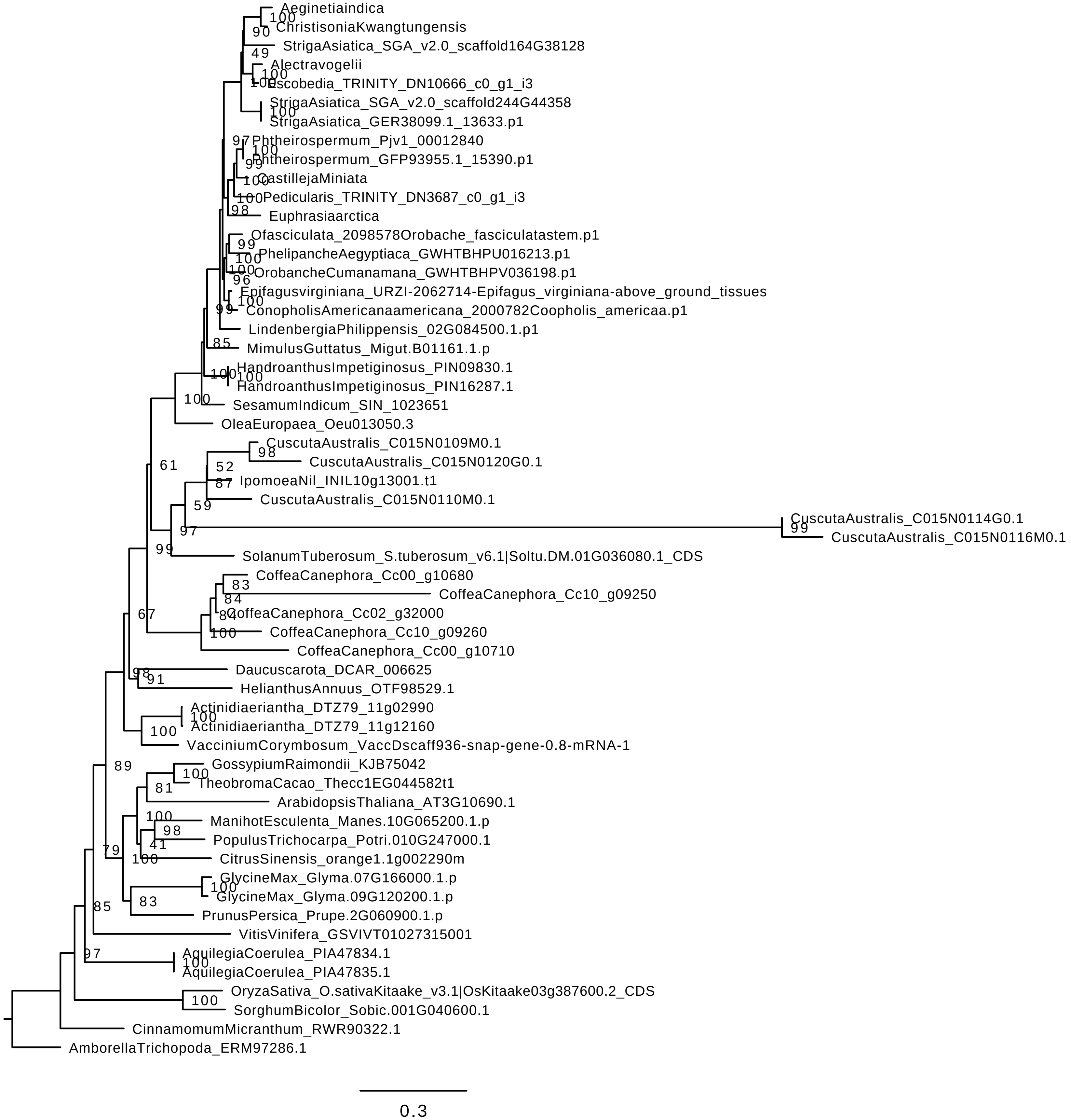

Aeginetiaindica
ChristisoniaKwangtungensis
StrigaAsiatica_SGA_v2.0_scaffold164G38128
Alectravogelii
Escobedia_TRINITY_DN10666_c0_g1_i3
StrigaAsiatica_SGA_v2.0_scaffold244G44358
StrigaAsiatica_GER38099.1_13633.p1
Phtheirospermum_Pjv1_00012840
Phtheirospermum_GFP93955.1_15390.p1
CastillejaMiniata
Pedicularis_TRINITY_DN3687_c0_g1_i3
Euphrasiaarctica
Ofasciculata_2098578Orobache_fasciculatastem.p1
PhelipancheAegyptiaca_GWHBHPU016213.p1
OrobancheCumanamana_GWHBHPV036198.p1
Epifagusvirginiana_URZI-2062714-Epifagus_virginiana-above_ground_tissues
ConopholisAmericanaamericana_2000782Coopholis_americaa.p1
LindenbergiaPhilippensis_02G084500.1.p1
MimulusGuttatus_Migut.B01161.1.p
HandroanthusImpetiginosus_PIN09830.1
HandroanthusImpetiginosus_PIN16287.1
SesamumIndicum_SIN_1023651
OleaEuropaea_Oeu013050.3
CuscutaAustralis_C015N0109M0.1
CuscutaAustralis_C015N0120G0.1
IpomoeaNil_INIL10g13001.t1
CuscutaAustralis_C015N0110M0.1
CuscutaAustralis_C015N0114G0.1
CuscutaAustralis_C015N0116M0.1
SolanumTuberosum_S.tuberosum_v6.1|Soltu.DM.01G036080.1_CDS
CoffeaCanephora_Cc00_g10680
CoffeaCanephora_Cc10_g09250
CoffeaCanephora_Cc02_g32000
CoffeaCanephora_Cc10_g09260
CoffeaCanephora_Cc00_g10710
Daucuscarota_DCAR_006625
HelianthusAnnuus_OTF98529.1
Actinidiaeriantha_DTZ79_11g02990
Actinidiaeriantha_DTZ79_11g12160
VacciniumCorymbosum_VaccDscaff936-snap-gene-0.8-mRNA-1
GossypiumRaimondii_KJB75042
TheobromaCacao_Thecc1EG044582t1
ArabidopsisThaliana_AT3G10690.1
ManihotEsculenta_Manes.10G065200.1.p
PopulusTrichocarpa_Potri.010G247000.1
CitrusSinensis_orange1.1g002290m
GlycineMax_Glyma.07G166000.1.p
GlycineMax_Glyma.09G120200.1.p
PrunusPersica_Prupe.2G060900.1.p
VitisVinifera_GSVIVT01027315001
AquilegiaCoerulea_PIA47834.1
AquilegiaCoerulea_PIA47835.1
OryzaSativa_O.sativaKitaake_v3.1|OsKitaake03g387600.2_CDS
SorghumBicolor_Sobic.001G040600.1
CinnamomumMicranthum_RWR90322.1
AmborellaTrichopoda_ERM97286.1
0.3
