# Supplementary material for: Altered Mitochondrial Respiration Is Associated With Loss of Nuclear‐Encoded OXPHOS Genes in Parasitic Broomrapes
Source: Ecol Evol. 2025 Jul 6;15(7):e71737. doi: 10.1002/ece3.71737 (PMC12230201; doi:10.1002/ece3.71737)
Supplement: Supplementary file 1 — Appendix S1. [file ECE3-15-e71737-s003.tgz › Orobanchaceae_O2K_Supplementary_v3/Data_S1/DataS1/RRR/OG0008131.filter.fas.treefile.pdf]

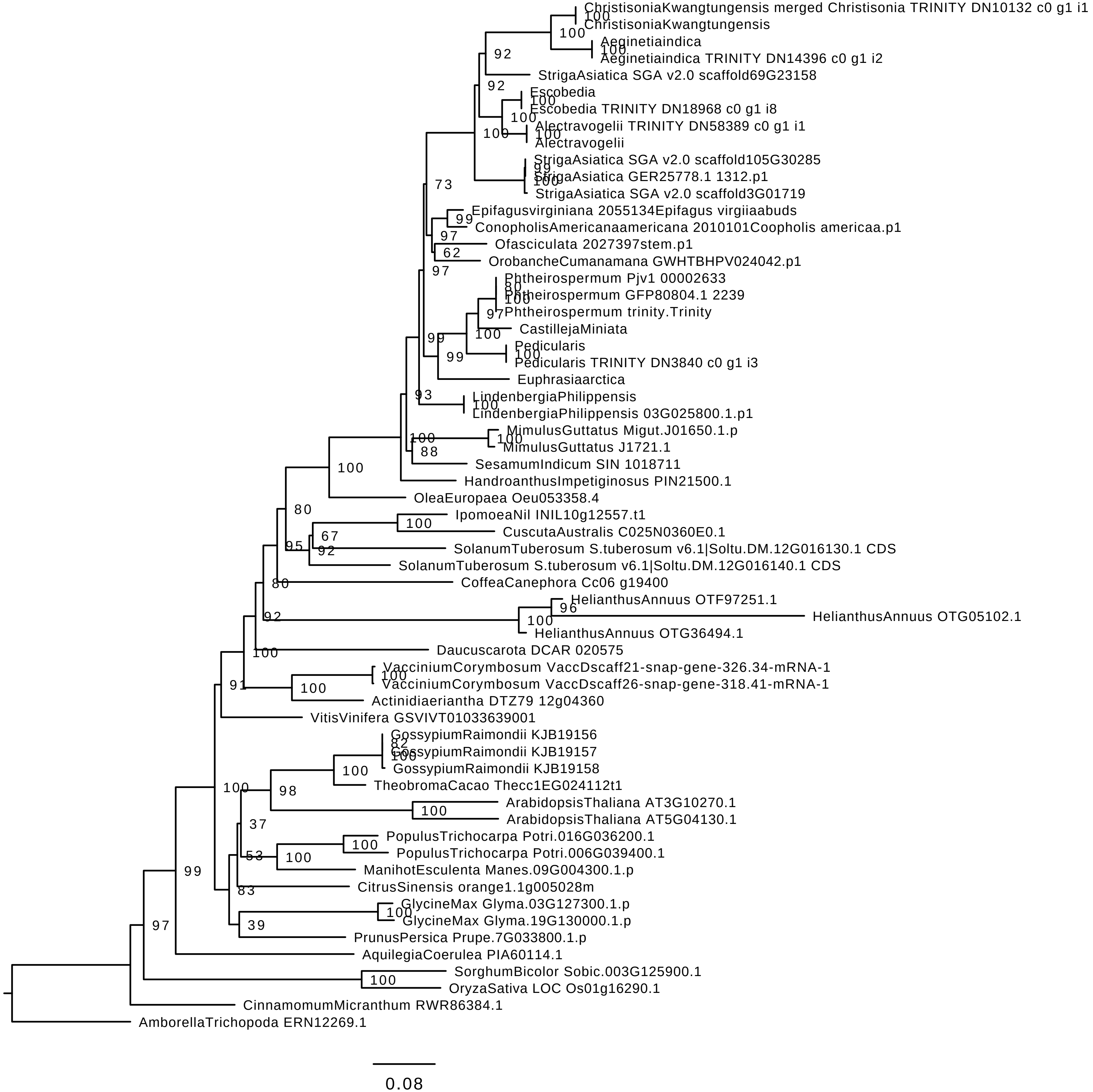
ChristisoniaKwangtungensis merged Christisonia TRINITY DN10132 c0 g1 i1
ChristisoniaKwangtungensis
Aeginetiaindica
Aeginetiaindica TRINITY DN14396 c0 g1 i2
StrigaAsiatica SGA v2.0 scaffold69G23158
Escobedia
Escobedia TRINITY DN18968 c0 g1 i8
Alectravogelii TRINITY DN58389 c0 g1 i1
Alectravogelii
StrigaAsiatica SGA v2.0 scaffold105G30285
StrigaAsiatica GER25778.1 1312.p1
StrigaAsiatica SGA v2.0 scaffold3G01719
Epifagusvirginiana 2055134Epifagus virgiiaabuds
ConopholisAmericanaamericana 2010101Coopholis americaa.p1
Ofasciculata 2027397stem.p1
OrobancheCumanamana GWHTBHPV024042.p1
Phtheirospermum Pjv1 00002633
Phtheirospermum GFP80804.1 2239
Phtheirospermum trinity.Trinity
CastillejaMiniata
Pedicularis
Pedicularis TRINITY DN3840 c0 g1 i3
Euphrasiaarctica
LindenbergiaPhilippensis
LindenbergiaPhilippensis 03G025800.1.p1
MimulusGuttatus Migut.J01650.1.p
MimulusGuttatus J1721.1
SesamumIndicum SIN 1018711
HandroanthusImpetiginosus PIN21500.1
OleaEuropaea Oeu053358.4
IpomoeaNil INIL10g12557.t1
CuscutaAustralis C025N0360E0.1
SolanumTuberosum S.tuberosum v6.1|Soltu.DM.12G016130.1 CDS
SolanumTuberosum S.tuberosum v6.1|Soltu.DM.12G016140.1 CDS
CoffeaCanephora Cc06 g19400
HelianthusAnnuus OTF97251.1
HelianthusAnnuus OTG05102.1
HelianthusAnnuus OTG36494.1
Daucuscarota DCAR 020575
VacciniumCorymbosum VaccDscaff21-snap-gene-326.34-mRNA-1
VacciniumCorymbosum VaccDscaff26-snap-gene-318.41-mRNA-1
Actinidiaeriantha DTZ79 12g04360
VitisVinifera GSVIVT01033639001
GossypiumRaimondii KJB19156
GossypiumRaimondii KJB19157
GossypiumRaimondii KJB19158
TheobromaCacao Thecc1EG024112t1
ArabidopsisThaliana AT3G10270.1
ArabidopsisThaliana AT5G04130.1
PopulusTrichocarpa Potri.016G036200.1
PopulusTrichocarpa Potri.006G039400.1
ManihotEsculenta Manes.09G004300.1.p
CitrusSinensis orange1.1g005028m
GlycineMax Glyma.03G127300.1.p
GlycineMax Glyma.19G130000.1.p
PrunusPersica Prupe.7G033800.1.p
AquilegiaCoerulea PIA60114.1
SorghumBicolor Sobic.003G125900.1
OryzaSativa LOC Os01g16290.1
CinnamomumMicranthum RWR86384.1
AmborellaTrichopoda ERN12269.1
0.08
