# Supplementary material for: Altered Mitochondrial Respiration Is Associated With Loss of Nuclear‐Encoded OXPHOS Genes in Parasitic Broomrapes
Source: Ecol Evol. 2025 Jul 6;15(7):e71737. doi: 10.1002/ece3.71737 (PMC12230201; doi:10.1002/ece3.71737)
Supplement: Supplementary file 1 — Appendix S1. [file ECE3-15-e71737-s003.tgz › Orobanchaceae_O2K_Supplementary_v3/Data_S1/DataS1/RRR/OG0005453.filter.fas.treefile.pdf]

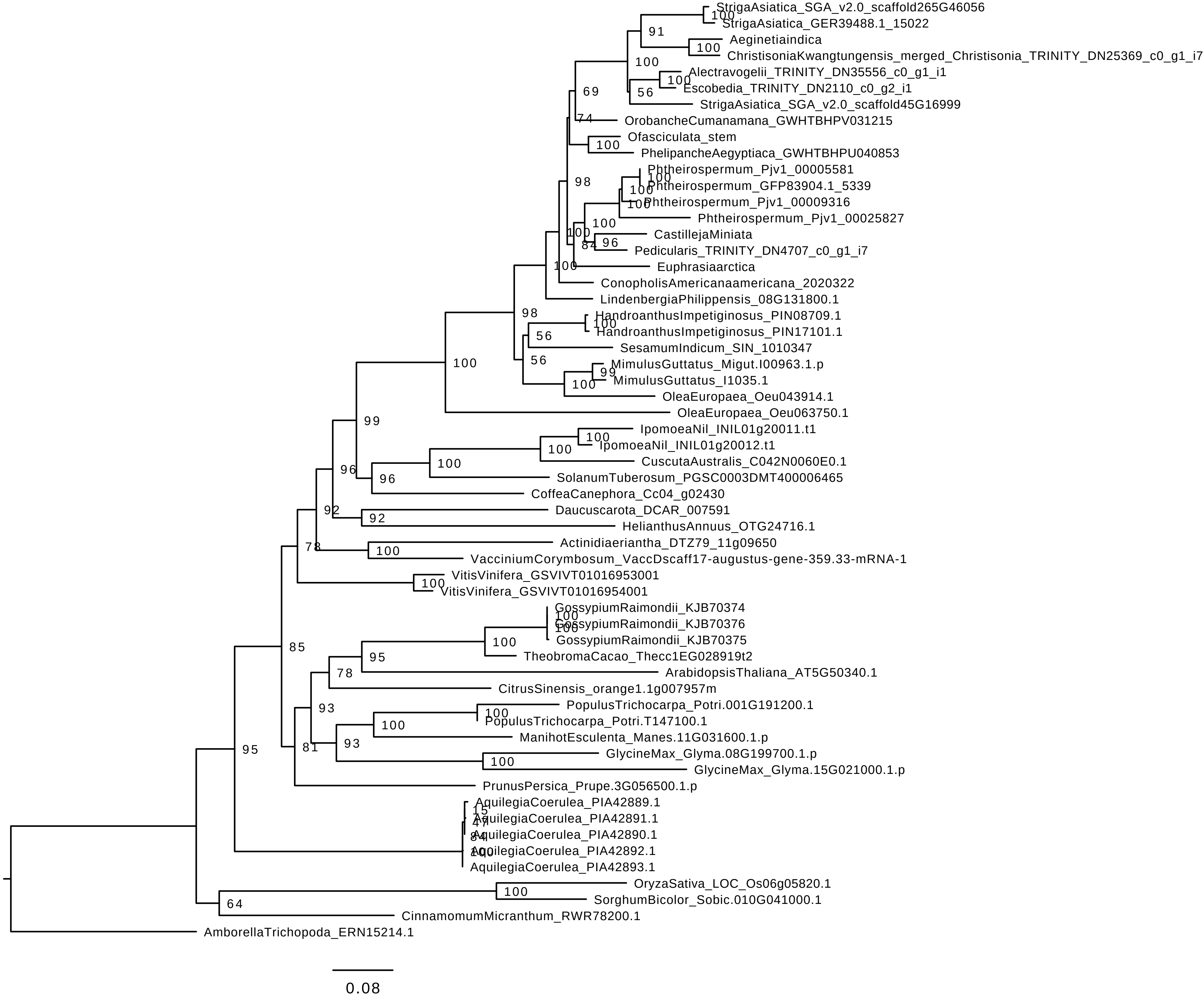
StrigaAsiatica_SGA_v2.0_scaffold265G46056
StrigaAsiatica_GER39488.1_15022
Aeginetiaindica
ChristisoniaKwangtungensis_merged_Christisonia_TRINITY_DN25369_c0_g1_i7
Alectravogelii_TRINITY_DN35556_c0_g1_i1
Escobedia_TRINITY_DN2110_c0_g2_i1
StrigaAsiatica_SGA_v2.0_scaffold45G16999
OrobancheCumanamana_GWHTBHPV031215
Ofasciculata_stem
PhelipancheAegyptiaca_GWHTBHPU040853
Phtheirospermum_Pjv1_00005581
Phtheirospermum_GFP83904.1_5339
Phtheirospermum_Pjv1_00009316
Phtheirospermum_Pjv1_00025827
CastillejaMiniata
Pedicularis_TRINITY_DN4707_c0_g1_i7
Euphrasiaarctica
ConopholisAmericanaamericana_2020322
LindenbergiaPhilippensis_08G131800.1
HandroanthusImpetiginosus_PIN08709.1
HandroanthusImpetiginosus_PIN17101.1
SesamumIndicum_SIN_1010347
MimulusGuttatus_Migut.I00963.1.p
MimulusGuttatus_I1035.1
OleaEuropaea_Oeu043914.1
OleaEuropaea_Oeu063750.1
IpomoeaNil_INIL01g20011.t1
IpomoeaNil_INIL01g20012.t1
CuscutaAustralis_C042N0060E0.1
SolanumTuberosum_PGSC0003DMT400006465
CoffeaCanephora_Cc04_g02430
Daucuscarota_DCAR_007591
HelianthusAnnuus_OTG24716.1
Actinidiaeriantha_DTZ79_11g09650
VacciniumCorymbosum_VaccDscaff17-augustus-gene-359.33-mRNA-1
VitisVinifera_GSVIVT01016953001
VitisVinifera_GSVIVT01016954001
GossypiumRaimondii_KJB70374
GossypiumRaimondii_KJB70376
GossypiumRaimondii_KJB70375
TheobromaCacao_Thecc1EG028919t2
ArabidopsisThaliana_AT5G50340.1
CitrusSinensis_orange1.1g007957m
PopulusTrichocarpa_Potri.001G191200.1
PopulusTrichocarpa_Potri.T147100.1
ManihotEsculenta_Manes.11G031600.1.p
GlycineMax_Glyma.08G199700.1.p
GlycineMax_Glyma.15G021000.1.p
PrunusPersica_Prupe.3G056500.1.p
AquilegiaCoerulea_PIA42889.1
AquilegiaCoerulea_PIA42891.1
AquilegiaCoerulea_PIA42890.1
AquilegiaCoerulea_PIA42892.1
AquilegiaCoerulea_PIA42893.1
OryzaSativa_LOC_Os06g05820.1
SorghumBicolor_Sobic.010G041000.1
CinnamomumMicranthum_RWR78200.1
AmborellaTrichopoda_ERN15214.1
0.08
