# Supplementary material for: Altered Mitochondrial Respiration Is Associated With Loss of Nuclear‐Encoded OXPHOS Genes in Parasitic Broomrapes
Source: Ecol Evol. 2025 Jul 6;15(7):e71737. doi: 10.1002/ece3.71737 (PMC12230201; doi:10.1002/ece3.71737)
Supplement: Supplementary file 1 — Appendix S1. [file ECE3-15-e71737-s003.tgz › Orobanchaceae_O2K_Supplementary_v3/Data_S1/DataS1/RRR/OG0007377.filter.fas.treefile.pdf]

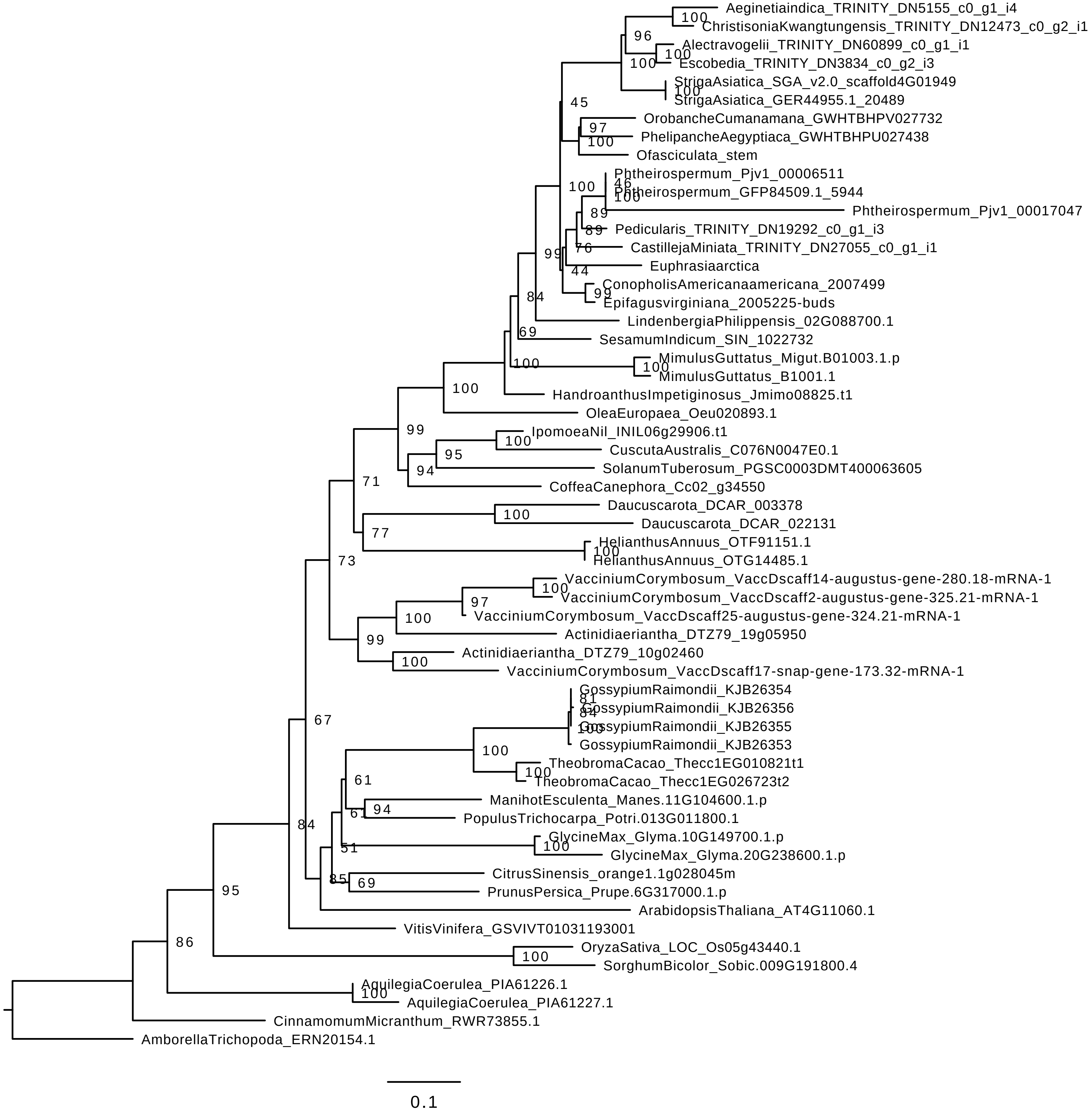
Aeginetiaindica_TRINITY_DN5155_c0_g1_i4
ChristisoniaKwangtungensis_TRINITY_DN12473_c0_g2_i1
Alectravogelii_TRINITY_DN60899_c0_g1_i1
Escobedia_TRINITY_DN3834_c0_g2_i3
StrigaAsiatica_SGA_v2.0_scaffold4G01949
StrigaAsiatica_GER44955.1_20489
OrobancheCumanamana_GWHTBHPV027732
PhelipancheAegyptiaca_GWHTBHPU027438
Ofasciculata_stem
Phtheirospermum_Pjv1_00006511
Phtheirospermum_GFP84509.1_5944
Phtheirospermum_Pjv1_00017047
Pedicularis_TRINITY_DN19292_c0_g1_i3
CastillejaMiniata_TRINITY_DN27055_c0_g1_i1
Euphrasiaarctica
ConopholisAmericanaamericana_2007499
Epifagusvirginiana_2005225-buds
LindenbergiaPhilippensis_02G088700.1
SesamumIndicum_SIN_1022732
MimulusGuttatus_Migut.B01003.1.p
MimulusGuttatus_B1001.1
HandroanthusImpetiginosus_Jmimo08825.t1
OleaEuropaea_Oeu020893.1
IpomoeaNil_INIL06g29906.t1
CuscutaAustralis_C076N0047E0.1
SolanumTuberosum_PGSC0003DMT400063605
CoffeaCanephora_Cc02_g34550
Daucuscarota_DCAR_003378
Daucuscarota_DCAR_022131
HelianthusAnnuus_OTF91151.1
HelianthusAnnuus_OTG14485.1
VacciniumCorymbosum_VaccDscaff14-augustus-gene-280.18-mRNA-1
VacciniumCorymbosum_VaccDscaff2-augustus-gene-325.21-mRNA-1
VacciniumCorymbosum_VaccDscaff25-augustus-gene-324.21-mRNA-1
Actinidiaeriantha_DTZ79_19g05950
Actinidiaeriantha_DTZ79_10g02460
VacciniumCorymbosum_VaccDscaff17-snap-gene-173.32-mRNA-1
GossypiumRaimondii_KJB26354
GossypiumRaimondii_KJB26356
GossypiumRaimondii_KJB26355
GossypiumRaimondii_KJB26353
TheobromaCacao_Thecc1EG010821t1
TheobromaCacao_Thecc1EG026723t2
ManihotEsculenta_Manes.11G104600.1.p
PopulusTrichocarpa_Potri.013G011800.1
GlycineMax_Glyma.10G149700.1.p
GlycineMax_Glyma.20G238600.1.p
CitrusSinensis_orange1.1g028045m
PrunusPersica_Prupe.6G317000.1.p
ArabidopsisThaliana_AT4G11060.1
VitisVinifera_GSVIVT01031193001
OryzaSativa_LOC_Os05g43440.1
SorghumBicolor_Sobic.009G191800.4
AquilegiaCoerulea_PIA61226.1
AquilegiaCoerulea_PIA61227.1
CinnamomumMicranthum_RWR73855.1
AmborellaTrichopoda_ERN20154.1
0.1
