# Supplementary material for: Altered Mitochondrial Respiration Is Associated With Loss of Nuclear‐Encoded OXPHOS Genes in Parasitic Broomrapes
Source: Ecol Evol. 2025 Jul 6;15(7):e71737. doi: 10.1002/ece3.71737 (PMC12230201; doi:10.1002/ece3.71737)
Supplement: Supplementary file 1 — Appendix S1. [file ECE3-15-e71737-s003.tgz › Orobanchaceae_O2K_Supplementary_v3/Data_S1/DataS1/RRR/OG0007721.filter.fas.treefile.pdf]

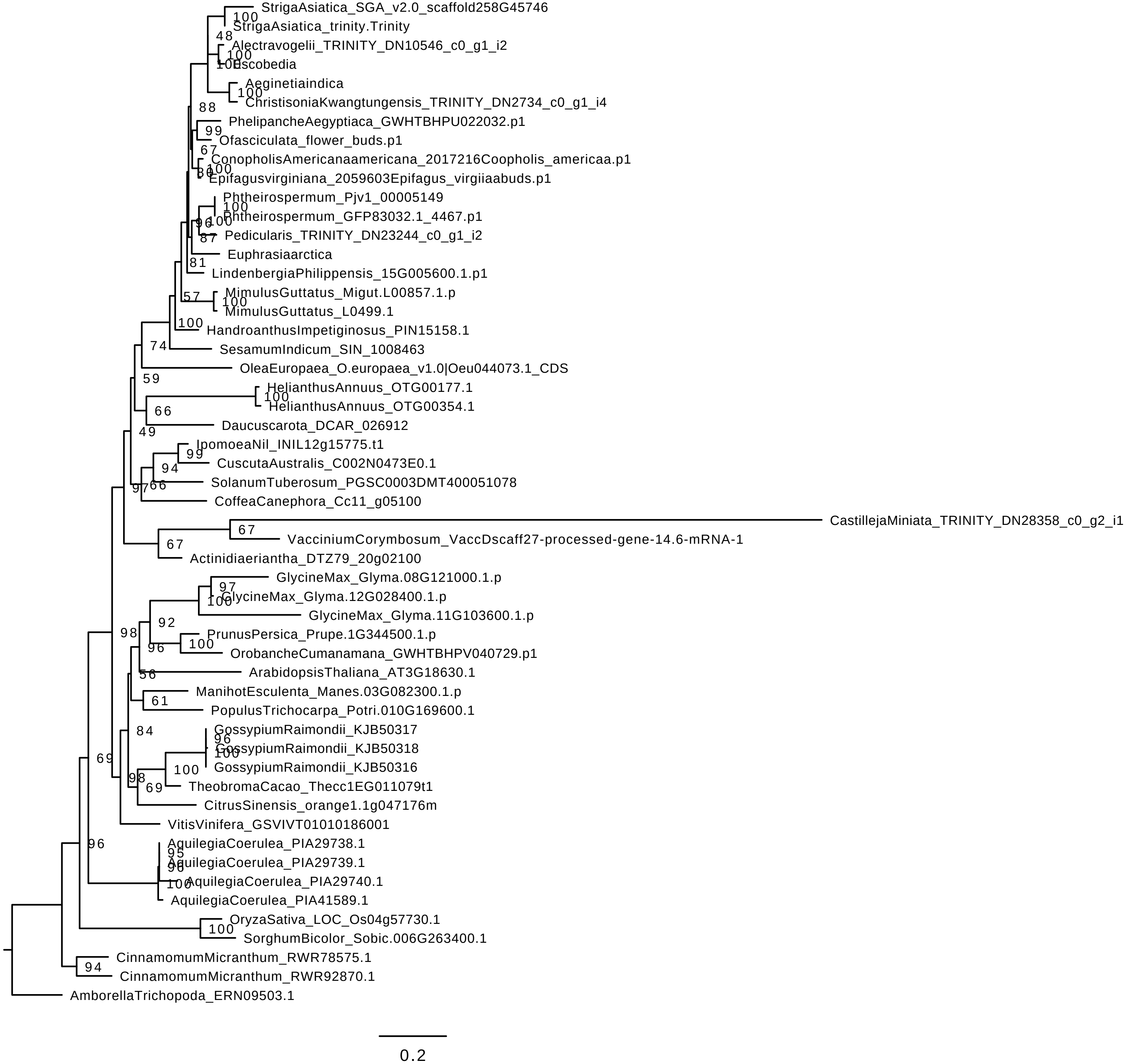
StrigaAsiatica_SGA_v2.0_scaffold258G45746
100
StrigaAsiatica_trinity.Trinity
48
Alectravogelii_TRINITY_DN10546_c0_g1_i2
100
100
Escobedia
Aeginetiaindica
100
ChristisoniaKwangtungensis_TRINITY_DN2734_c0_g1_i4
88
PhelipancheAegyptiaca_GWHTBHPU022032.p1
99
Ofasciculata_flower_buds.p1
67
ConopholisAmericanaamericana_2017216Coopholis_americaa.p1
100
Epifagusvirginiana_2059603Epifagus_virgiiaabuds.p1
Phtheirospermum_Pjv1_00005149
100
Phtheirospermum_GFP83032.1_4467.p1
96
100
Pedicularis_TRINITY_DN23244_c0_g1_i2
87
Euphrasiaarctica
81
LindenbergiaPhilippensis_15G005600.1.p1
57
MimulusGuttatus_Migut.L00857.1.p
100
MimulusGuttatus_L0499.1
100
HandroanthusImpetiginosus_PIN15158.1
74
SesamumIndicum_SIN_1008463
OleaEuropaea_O.europaea_v1.0|Oeu044073.1_CDS
59
HelianthusAnnuus_OTG00177.1
100
HelianthusAnnuus_OTG00354.1
66
Daucuscarota_DCAR_026912
49
IpomoeaNil_INIL12g15775.t1
99
CuscutaAustralis_C002N0473E0.1
94
SolanumTuberosum_PGSC0003DMT400051078
97
66
CoffeaCanephora_Cc11_g05100
CastillejaMiniata_TRINITY_DN28358_c0_g2_i1
67
VacciniumCorymbosum_VaccDscaff27-processed-gene-14.6-mRNA-1
67
Actinidiaeriantha_DTZ79_20g02100
GlycineMax_Glyma.08G121000.1.p
97
GlycineMax_Glyma.12G028400.1.p
100
GlycineMax_Glyma.11G103600.1.p
92
98
PrunusPersica_Prupe.1G344500.1.p
100
96
OrobancheCumanamana_GWHTBHPV040729.p1
56
ArabidopsisThaliana_AT3G18630.1
ManihotEsculenta_Manes.03G082300.1.p
61
PopulusTrichocarpa_Potri.010G169600.1
84
GossypiumRaimondii_KJB50317
96
GossypiumRaimondii_KJB50318
100
GossypiumRaimondii_KJB50316
100
69
98
69
TheobromaCacao_Thecc1EG011079t1
CitrusSinensis_orange1.1g047176m
VitisVinifera_GSVIVT01010186001
96
AquilegiaCoerulea_PIA29738.1
95
AquilegiaCoerulea_PIA29739.1
96
100
AquilegiaCoerulea_PIA29740.1
AquilegiaCoerulea_PIA41589.1
OryzaSativa_LOC_Os04g57730.1
100
SorghumBicolor_Sobic.006G263400.1
CinnamomumMicranthum_RWR78575.1
94
CinnamomumMicranthum_RWR92870.1
AmborellaTrichopoda_ERN09503.1
0.2
