# Supplementary material for: Altered Mitochondrial Respiration Is Associated With Loss of Nuclear‐Encoded OXPHOS Genes in Parasitic Broomrapes
Source: Ecol Evol. 2025 Jul 6;15(7):e71737. doi: 10.1002/ece3.71737 (PMC12230201; doi:10.1002/ece3.71737)
Supplement: Supplementary file 1 — Appendix S1. [file ECE3-15-e71737-s003.tgz › Orobanchaceae_O2K_Supplementary_v3/Data_S1/DataS1/RRR/OG0003488.filter.fas.treefile.pdf]

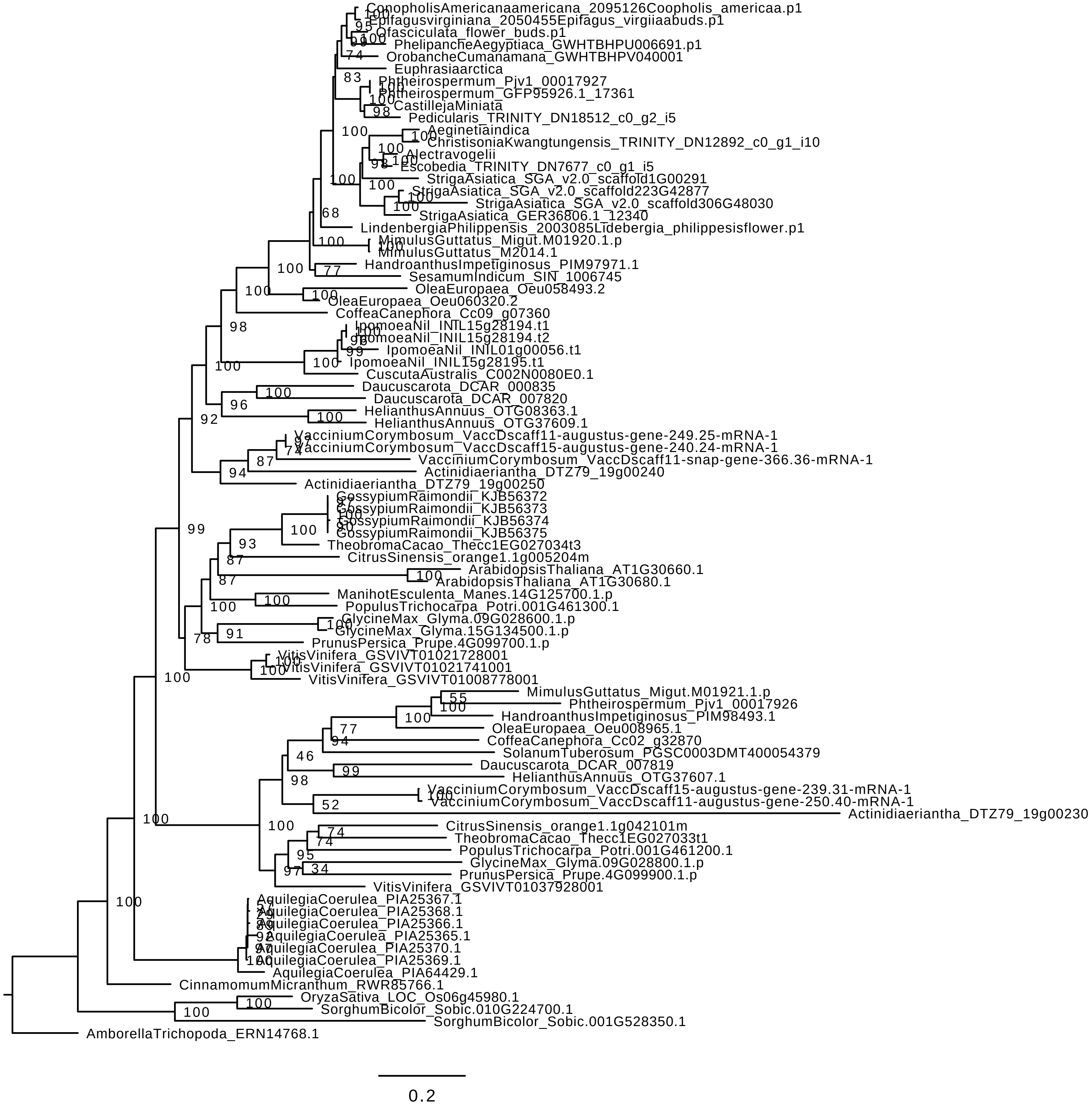

ConopholisAmericanaamericana_2095126Coopholis_americaa.p1
Epifagusvirginiana_2050455Epifagus_virgiiaabuds.p1
Ofasciculata_flower_buds.p1
PhelipancheAegyptiaca_GWHTBHPU006691.p1
OrobancheCumanamana_GWHTBHPV040001
Euphrasiaarctica
Phtheirospermum_Pjv1_00017927
Phtheirospermum_GFP95926.1_17361
CastillejaMiniata
Pedicularis_TRINITY_DN18512_c0_g2_i5
Aeginetiaindica
ChristisoniaKwangtungensis_TRINITY_DN12892_c0_g1_i10
Alectravogelii
Escobedia_TRINITY_DN7677_c0_g1_i5
StrigaAsiatica_SGA_v2.0_scaffold1G00291
StrigaAsiatica_SGA_v2.0_scaffold223G42877
StrigaAsiatica_SGA_v2.0_scaffold306G48030
StrigaAsiatica_GER36806.1_12340
LindenbergiaPhilippensis_2003085Lidebergia_philippesisflower.p1
MimulusGuttatus_Migut.M01920.1.p
MimulusGuttatus_M2014.1
HandroanthusImpetiginosus_PIM97971.1
SesamumIndicum_SIN_1006745
OleaEuropaea_Oeu058493.2
OleaEuropaea_Oeu060320.2
CoffeaCanephora_Cc09_g07360
IpomoeaNil_INIL15g28194.t1
gIpomoeaNil_INIL15g28194.t2
IpomoeaNil_INIL01g00056.t1
IpomoeaNil_INIL15g28195.t1
CuscutaAustralis_C002N0080E0.1
Daucuscarota_DCAR_000835
Daucuscarota_DCAR_007820
HelianthusAnnuus_OTG08363.1
HelianthusAnnuus_OTG37609.1
VacciniumCorymbosum_VaccDscaff11-augustus-gene-249.25-mRNA-1
VacciniumCorymbosum_VaccDscaff15-augustus-gene-240.24-mRNA-1
VacciniumCorymbosum_VaccDscaff11-snap-gene-366.36-mRNA-1
Actinidiaeriantha_DTZ79_19g00240
Actinidiaeriantha_DTZ79_19g00250
GossypiumRaimondii_KJB56372
GossypiumRaimondii_KJB56373
GossypiumRaimondii_KJB56374
GossypiumRaimondii_KJB56375
TheobromaCacao_Thecc1EG027034t3
CitrusSinensis_orange1.1g005204m
ArabidopsisThaliana_AT1G30660.1
ArabidopsisThaliana_AT1G30680.1
ManihotEsculenta_Manes.14G125700.1.p
PopulusTrichocarpa_Potri.001G461300.1
GlycineMax_Glyma.09G028600.1.p
GlycineMax_Glyma.15G134500.1.p
PrunusPersica_Prupe.4G099700.1.p
VitisVinifera_GSVIVT01021728001
VitisVinifera_GSVIVT01021741001
VitisVinifera_GSVIVT01008778001
MimulusGuttatus_Migut.M01921.1.p
Phtheirospermum_Pjv1_00017926
HandroanthusImpetiginosus_PIM98493.1
OleaEuropaea_Oeu008965.1
CoffeaCanephora_Cc02_g32870
SolanumTuberosum_PGSC0003DMT400054379
Daucuscarota_DCAR_007819
HelianthusAnnuus_OTG37607.1
VacciniumCorymbosum_VaccDscaff15-augustus-gene-239.31-mRNA-1
VacciniumCorymbosum_VaccDscaff11-augustus-gene-250.40-mRNA-1
Actinidiaeriantha_DTZ79_19g00230
CitrusSinensis_orange1.1g042101m
TheobromaCacao_Thecc1EG027033t1
PopulusTrichocarpa_Potri.001G461200.1
GlycineMax_Glyma.09G028800.1.p
PrunusPersica_Prupe.4G099900.1.p
VitisVinifera_GSVIVT01037928001
AquilegiaCoerulea_PIA25367.1
AquilegiaCoerulea_PIA25368.1
AquilegiaCoerulea_PIA25366.1
AquilegiaCoerulea_PIA25365.1
AquilegiaCoerulea_PIA25370.1
AquilegiaCoerulea_PIA25369.1
AquilegiaCoerulea_PIA64429.1
CinnamomumMicranthum_RWR85766.1
OryzaSativa_LOC_Os06g45980.1
SorghumBicolor_Sobic.010G224700.1
SorghumBicolor_Sobic.001G528350.1
AmborellaTrichopoda_ERN14768.1
0.2
