# Supplementary material for: Altered Mitochondrial Respiration Is Associated With Loss of Nuclear‐Encoded OXPHOS Genes in Parasitic Broomrapes
Source: Ecol Evol. 2025 Jul 6;15(7):e71737. doi: 10.1002/ece3.71737 (PMC12230201; doi:10.1002/ece3.71737)
Supplement: Supplementary file 1 — Appendix S1. [file ECE3-15-e71737-s003.tgz › Orobanchaceae_O2K_Supplementary_v3/Data_S1/DataS1/RRR/OG0005593.filter.fas.treefile.pdf]

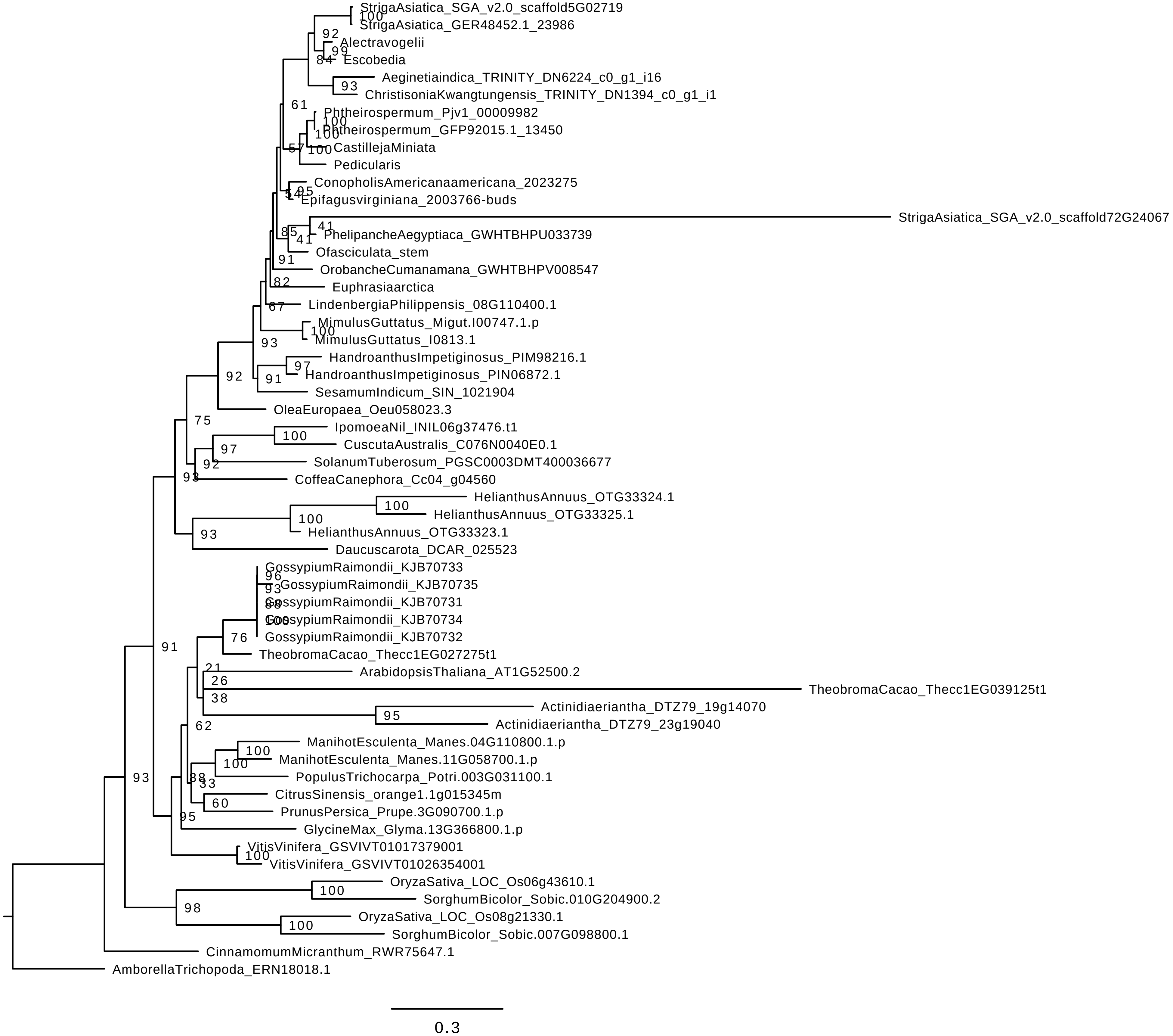
StrigaAsiatica_SGA_v2.0_scaffold5G02719
StrigaAsiatica_GER48452.1_23986
Alectravogelii
Escobedia
Aeginetiaindica_TRINITY_DN6224_c0_g1_i16
ChristisoniaKwangtungensis_TRINITY_DN1394_c0_g1_i1
Phtheirospermum_Pjv1_00009982
Phtheirospermum_GFP92015.1_13450
CastillejaMiniata
Pedicularis
ConopholisAmericanaamericana_2023275
Epifagusvirginiana_2003766-buds
StrigaAsiatica_SGA_v2.0_scaffold72G24067
PhelipancheAegyptiaca_GWHTBHPU033739
Ofasciculata_stem
OrobancheCumanamana_GWHTBHPV008547
Euphrasiaarctica
LindenbergiaPhilippensis_08G110400.1
MimulusGuttatus_Migut.I00747.1.p
MimulusGuttatus_I0813.1
HandroanthusImpetiginosus_PIM98216.1
HandroanthusImpetiginosus_PIN06872.1
SesamumIndicum_SIN_1021904
OleaEuropaea_Oeu058023.3
IpomoeaNil_INIL06g37476.t1
CuscutaAustralis_C076N0040E0.1
SolanumTuberosum_PGSC0003DMT400036677
CoffeaCanephora_Cc04_g04560
HelianthusAnnuus_OTG33324.1
HelianthusAnnuus_OTG33325.1
HelianthusAnnuus_OTG33323.1
Daucuscarota_DCAR_025523
GossypiumRaimondii_KJB70733
GossypiumRaimondii_KJB70735
GossypiumRaimondii_KJB70731
GossypiumRaimondii_KJB70734
GossypiumRaimondii_KJB70732
TheobromaCacao_Thecc1EG027275t1
ArabidopsisThaliana_AT1G52500.2
TheobromaCacao_Thecc1EG039125t1
Actinidiaeriantha_DTZ79_19g14070
Actinidiaeriantha_DTZ79_23g19040
ManihotEsculenta_Manes.04G110800.1.p
ManihotEsculenta_Manes.11G058700.1.p
PopulusTrichocarpa_Potri.003G031100.1
CitrusSinensis_orange1.1g015345m
PrunusPersica_Prupe.3G090700.1.p
GlycineMax_Glyma.13G366800.1.p
VitisVinifera_GSVIVT01017379001
VitisVinifera_GSVIVT01026354001
OryzaSativa_LOC_Os06g43610.1
SorghumBicolor_Sobic.010G204900.2
OryzaSativa_LOC_Os08g21330.1
SorghumBicolor_Sobic.007G098800.1
CinnamomumMicranthum_RWR75647.1
AmborellaTrichopoda_ERN18018.1
0.3
