# Supplementary material for: Altered Mitochondrial Respiration Is Associated With Loss of Nuclear‐Encoded OXPHOS Genes in Parasitic Broomrapes
Source: Ecol Evol. 2025 Jul 6;15(7):e71737. doi: 10.1002/ece3.71737 (PMC12230201; doi:10.1002/ece3.71737)
Supplement: Supplementary file 1 — Appendix S1. [file ECE3-15-e71737-s003.tgz › Orobanchaceae_O2K_Supplementary_v3/Data_S1/DataS1/RRR/OG0005887.filter.fas.treefile.pdf]

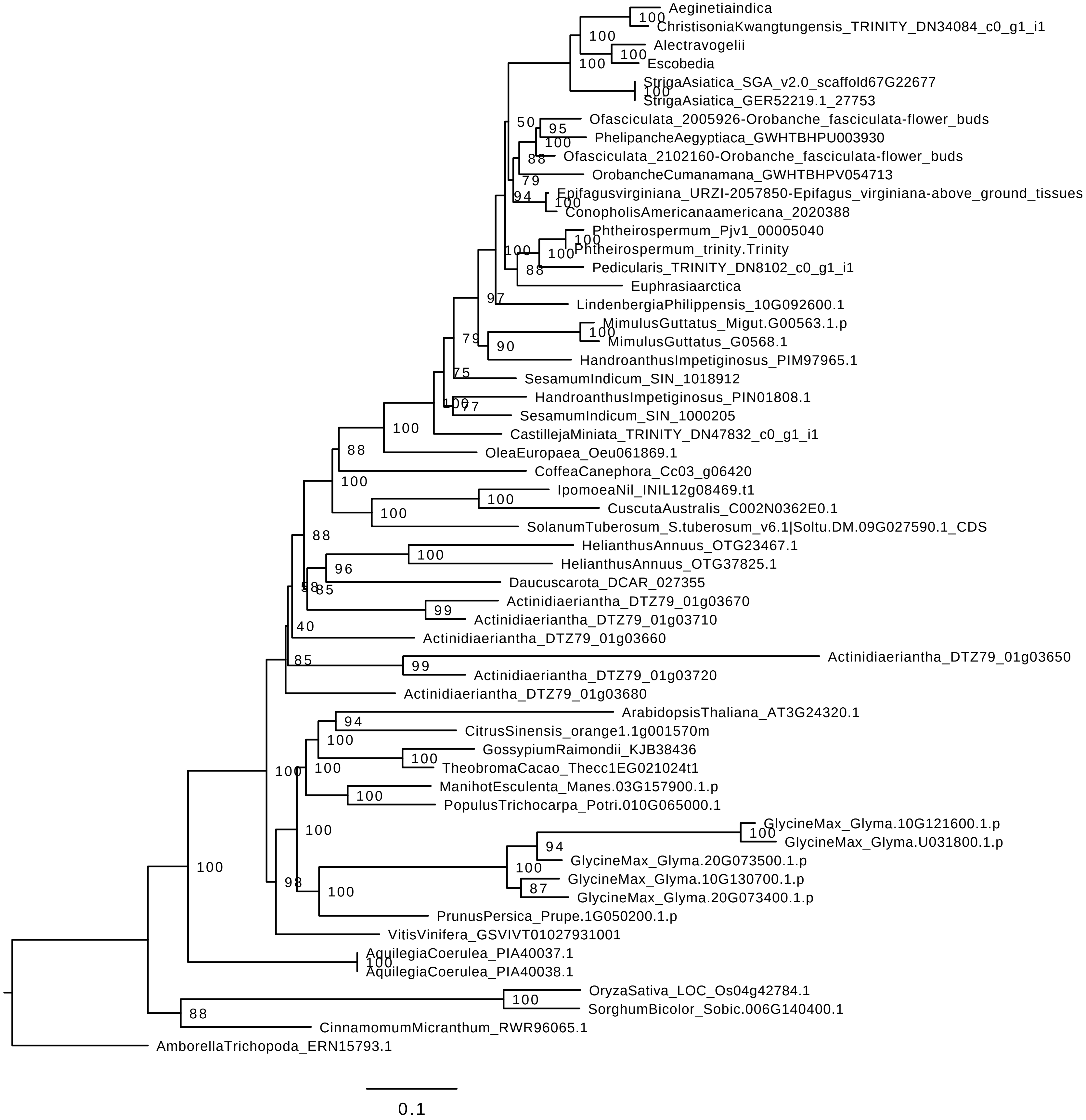
Aeginetiaindica
ChristisoniaKwangtungensis_TRINITY_DN34084_c0_g1_i1
Alectravogelii
Escobedia
StrigaAsiatica_SGA_v2.0_scaffold67G22677
StrigaAsiatica_GER52219.1_27753
Ofasciculata_2005926-Orobanche_fasciculata-flower_buds
PhelipancheAegyptiaca_GWHTBHPU003930
Ofasciculata_2102160-Orobanche_fasciculata-flower_buds
OrobancheCumanamana_GWHTBHPV054713
Epifagusvirginiana_URZI-2057850-Epifagus_virginiana-above_ground_tissues
ConopholisAmericanaamericana_2020388
Phtheirospermum_Pjv1_00005040
Phtheirospermum_trinity.Trinity
Pedicularis_TRINITY_DN8102_c0_g1_i1
Euphrasiaarctica
LindenbergiaPhilippensis_10G092600.1
MimulusGuttatus_Migut.G00563.1.p
MimulusGuttatus_G0568.1
HandroanthusImpetiginosus_PIM97965.1
SesamumIndicum_SIN_1018912
HandroanthusImpetiginosus_PIN01808.1
SesamumIndicum_SIN_1000205
CastillejaMiniata_TRINITY_DN47832_c0_g1_i1
OleaEuropaea_Oeu061869.1
CoffeaCanephora_Cc03_g06420
IpomoeaNil_INIL12g08469.t1
CuscutaAustralis_C002N0362E0.1
SolanumTuberosum_S.tuberosum_v6.1|Soltu.DM.09G027590.1_CDS
HelianthusAnnuus_OTG23467.1
HelianthusAnnuus_OTG37825.1
Daucuscarota_DCAR_027355
Actinidiaeriantha_DTZ79_01g03670
Actinidiaeriantha_DTZ79_01g03710
Actinidiaeriantha_DTZ79_01g03660
Actinidiaeriantha_DTZ79_01g03650
Actinidiaeriantha_DTZ79_01g03720
Actinidiaeriantha_DTZ79_01g03680
ArabidopsisThaliana_AT3G24320.1
CitrusSinensis_orange1.1g001570m
GossypiumRaimondii_KJB38436
TheobromaCacao_Thecc1EG021024t1
ManihotEsculenta_Manes.03G157900.1.p
PopulusTrichocarpa_Potri.010G065000.1
GlycineMax_Glyma.10G121600.1.p
GlycineMax_Glyma.U031800.1.p
GlycineMax_Glyma.20G073500.1.p
GlycineMax_Glyma.10G130700.1.p
GlycineMax_Glyma.20G073400.1.p
PrunusPersica_Prupe.1G050200.1.p
VitisVinifera_GSVIVT01027931001
AquilegiaCoerulea_PIA40037.1
AquilegiaCoerulea_PIA40038.1
OryzaSativa_LOC_Os04g42784.1
SorghumBicolor_Sobic.006G140400.1
CinnamomumMicranthum_RWR96065.1
AmborellaTrichopoda_ERN15793.1
0.1
