# Supplementary material for: Altered Mitochondrial Respiration Is Associated With Loss of Nuclear‐Encoded OXPHOS Genes in Parasitic Broomrapes
Source: Ecol Evol. 2025 Jul 6;15(7):e71737. doi: 10.1002/ece3.71737 (PMC12230201; doi:10.1002/ece3.71737)
Supplement: Supplementary file 1 — Appendix S1. [file ECE3-15-e71737-s003.tgz › Orobanchaceae_O2K_Supplementary_v3/Data_S1/DataS1/RRR/OG0008579.filter.fas.treefile.pdf]

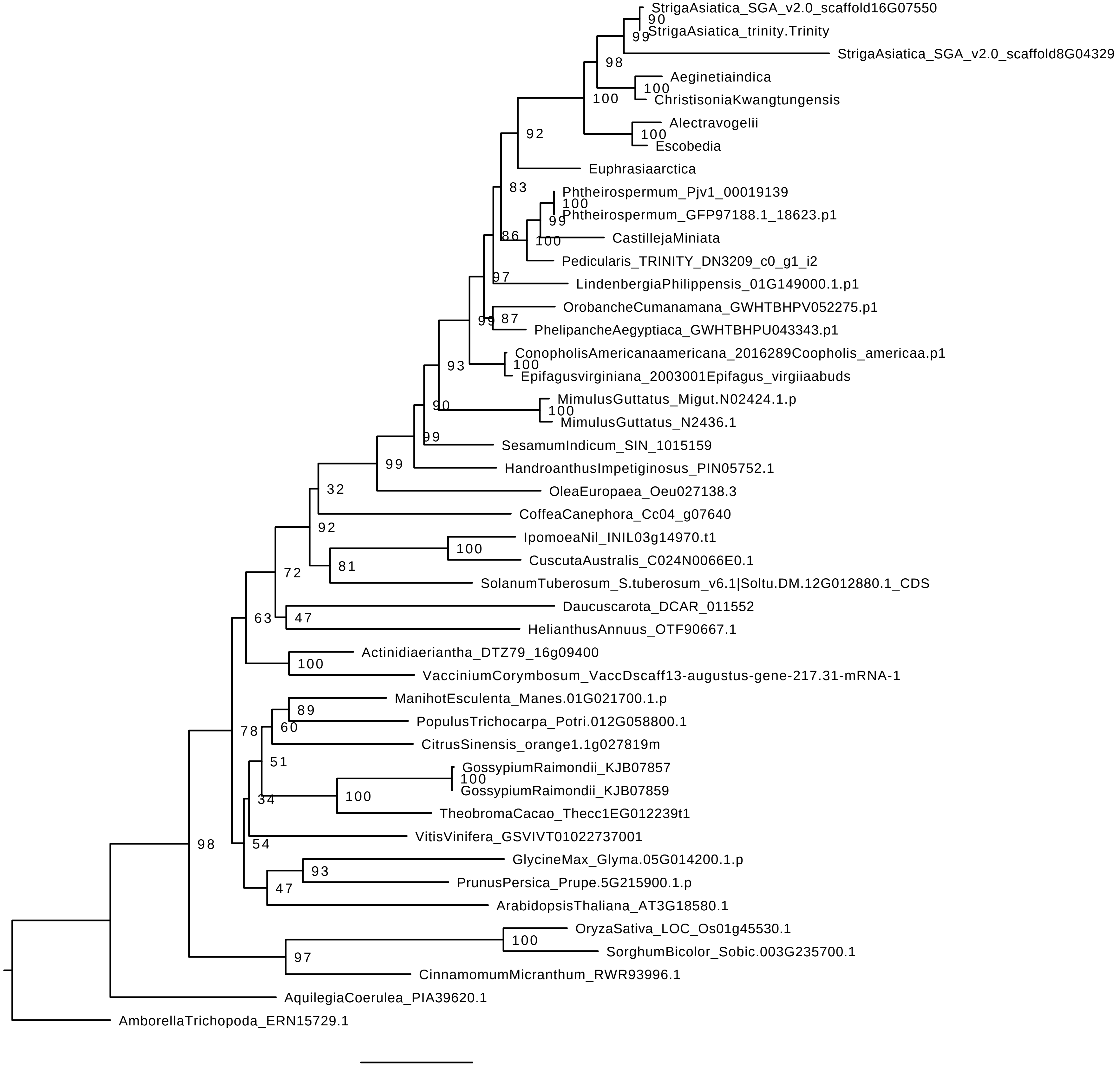
StrigaAsiatica_SGA_v2.0_scaffold16G07550
StrigaAsiatica_trinity.Trinity
StrigaAsiatica_SGA_v2.0_scaffold8G04329
Aeginetiaindica
ChristisoniaKwangtungensis
Alectravogelii
Escobedia
Euphrasiaarctica
Phtheirospermum_Pjv1_00019139
Phtheirospermum_GFP97188.1_18623.p1
CastillejaMiniata
Pedicularis_TRINITY_DN3209_c0_g1_i2
LindenbergiaPhilippensis_01G149000.1.p1
OrobancheCumanamana_GWHTBHPV052275.p1
PhelipancheAegyptiaca_GWHTBHPU043343.p1
ConopholisAmericanaamericana_2016289Coopholis_americaa.p1
Epifagusvirginiana_2003001Epifagus_virgiiaabuds
MimulusGuttatus_Migut.N02424.1.p
MimulusGuttatus_N2436.1
SesamumIndicum_SIN_1015159
HandroanthusImpetiginosus_PIN05752.1
OleaEuropaea_Oeu027138.3
CoffeaCanephora_Cc04_g07640
IpomoeaNil_INIL03g14970.t1
CuscutaAustralis_C024N0066E0.1
SolanumTuberosum_S.tuberosum_v6.1|Soltu.DM.12G012880.1_CDS
Daucuscarota_DCAR_011552
HelianthusAnnuus_OTF90667.1
Actinidiaeriantha_DTZ79_16g09400
VacciniumCorymbosum_VaccDscaff13-augustus-gene-217.31-mRNA-1
ManihotEsculenta_Manes.01G021700.1.p
PopulusTrichocarpa_Potri.012G058800.1
CitrusSinensis_orange1.1g027819m
GossypiumRaimondii_KJB07857
GossypiumRaimondii_KJB07859
TheobromaCacao_Thecc1EG012239t1
VitisVinifera_GSVIVT01022737001
GlycineMax_Glyma.05G014200.1.p
PrunusPersica_Prupe.5G215900.1.p
ArabidopsisThaliana_AT3G18580.1
OryzaSativa_LOC_Os01g45530.1
SorghumBicolor_Sobic.003G235700.1
CinnamomumMicranthum_RWR93996.1
AquilegiaCoerulea_PIA39620.1
AmborellaTrichopoda_ERN15729.1
0.2
