# Supplementary material for: Altered Mitochondrial Respiration Is Associated With Loss of Nuclear‐Encoded OXPHOS Genes in Parasitic Broomrapes
Source: Ecol Evol. 2025 Jul 6;15(7):e71737. doi: 10.1002/ece3.71737 (PMC12230201; doi:10.1002/ece3.71737)
Supplement: Supplementary file 1 — Appendix S1. [file ECE3-15-e71737-s003.tgz › Orobanchaceae_O2K_Supplementary_v3/Data_S1/DataS1/RRR/OG0004058.filter.fas.treefile.pdf]

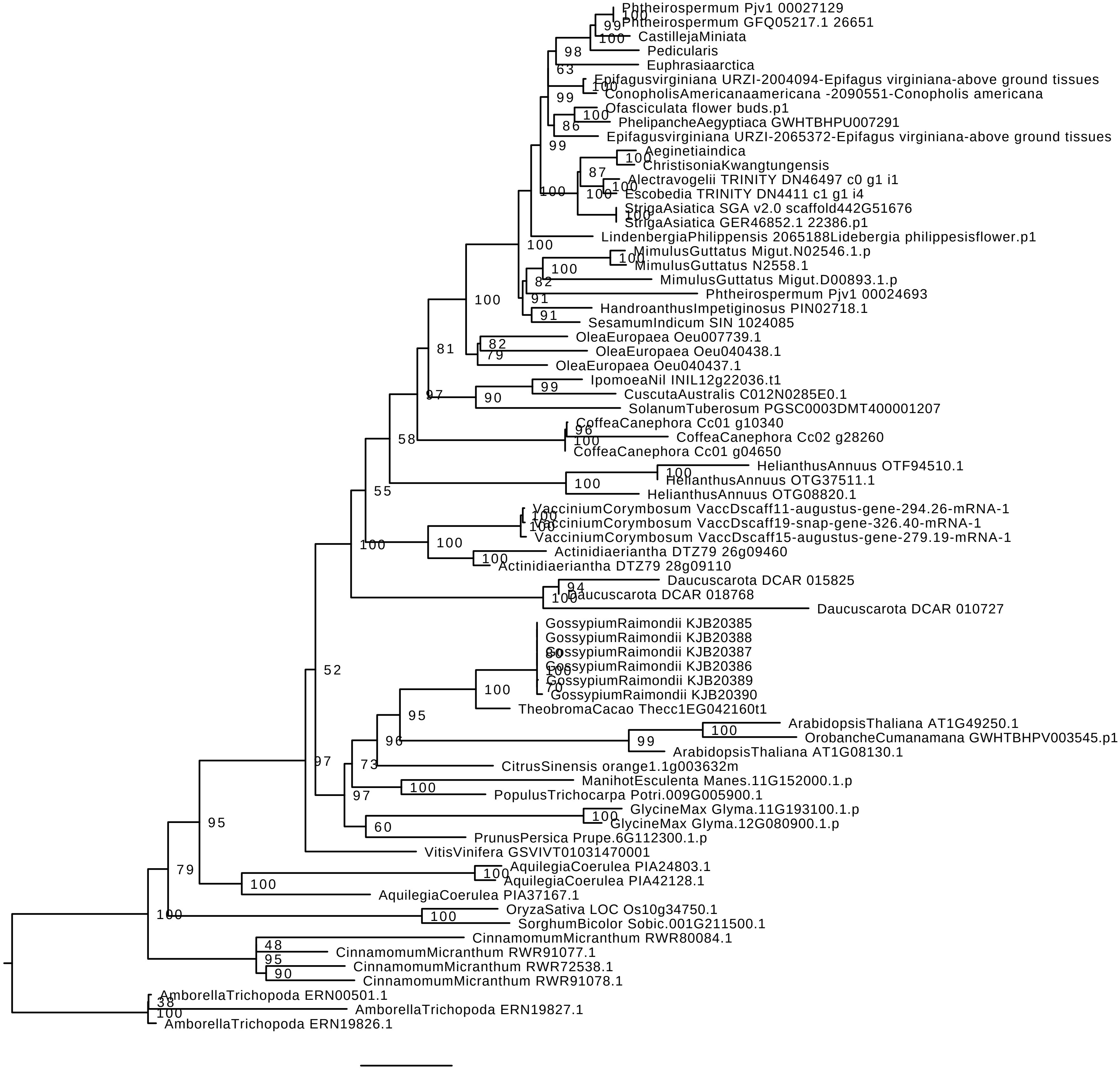
Phtheirospermum Pjv1 00027129
Phtheirospermum GFQ05217.1 26651
CastillejaMiniata
Pedicularis
Euphrasiaarctica
Epifagusvirginiana URZI-2004094-Epifagus virginiana-above ground tissues
ConopholisAmericanaamericana -2090551-Conopholis americana
Ofasciculata flower buds.p1
PhelipancheAegyptiaca GWHTBHPU007291
Epifagusvirginiana URZI-2065372-Epifagus virginiana-above ground tissues
Aeginetiaindica
ChristisoniaKwangtungensis
Alectravogelii TRINITY DN46497 c0 g1 i1
Escobedia TRINITY DN4411 c1 g1 i4
StrigaAsiatica SGA v2.0 scaffold442G51676
StrigaAsiatica GER46852.1 22386.p1
LindenbergiaPhilippensis 2065188Lidebergia philippesisflower.p1
MimulusGuttatus Migut.N02546.1.p
MimulusGuttatus N2558.1
MimulusGuttatus Migut.D00893.1.p
Phtheirospermum Pjv1 00024693
HandroanthusImpetiginosus PIN02718.1
SesamumIndicum SIN 1024085
OleaEuropaea Oeu007739.1
OleaEuropaea Oeu040438.1
OleaEuropaea Oeu040437.1
IpomoeaNil INIL12g22036.t1
CuscutaAustralis C012N0285E0.1
SolanumTuberosum PGSC0003DMT400001207
CoffeaCanephora Cc01 g10340
CoffeaCanephora Cc02 g28260
CoffeaCanephora Cc01 g04650
HelianthusAnnuus OTF94510.1
HelianthusAnnuus OTG37511.1
HelianthusAnnuus OTG08820.1
VacciniumCorymbosum VaccDscaff11-augustus-gene-294.26-mRNA-1
VacciniumCorymbosum VaccDscaff19-snap-gene-326.40-mRNA-1
VacciniumCorymbosum VaccDscaff15-augustus-gene-279.19-mRNA-1
Actinidiaeriantha DTZ79 26g09460
Actinidiaeriantha DTZ79 28g09110
Daucuscarota DCAR 015825
Daucuscarota DCAR 018768
Daucuscarota DCAR 010727
GossypiumRaimondii KJB20385
GossypiumRaimondii KJB20388
GossypiumRaimondii KJB20387
GossypiumRaimondii KJB20386
GossypiumRaimondii KJB20389
GossypiumRaimondii KJB20390
TheobromaCacao Thecc1EG042160t1
ArabidopsisThaliana AT1G49250.1
OrobancheCumanamana GWHTBHPV003545.p1
ArabidopsisThaliana AT1G08130.1
CitrusSinensis orange1.1g003632m
ManihotEsculenta Manes.11G152000.1.p
PopulusTrichocarpa Potri.009G005900.1
GlycineMax Glyma.11G193100.1.p
GlycineMax Glyma.12G080900.1.p
PrunusPersica Prupe.6G112300.1.p
VitisVinifera GSVIVT01031470001
AquilegiaCoerulea PIA24803.1
AquilegiaCoerulea PIA42128.1
AquilegiaCoerulea PIA37167.1
OryzaSativa LOC Os10g34750.1
SorghumBicolor Sobic.001G211500.1
CinnamomumMicranthum RWR80084.1
CinnamomumMicranthum RWR91077.1
CinnamomumMicranthum RWR72538.1
CinnamomumMicranthum RWR91078.1
AmborellaTrichopoda ERN00501.1
AmborellaTrichopoda ERN19827.1
AmborellaTrichopoda ERN19826.1
0.09
