# Supplementary material for: Altered Mitochondrial Respiration Is Associated With Loss of Nuclear‐Encoded OXPHOS Genes in Parasitic Broomrapes
Source: Ecol Evol. 2025 Jul 6;15(7):e71737. doi: 10.1002/ece3.71737 (PMC12230201; doi:10.1002/ece3.71737)
Supplement: Supplementary file 1 — Appendix S1. [file ECE3-15-e71737-s003.tgz › Orobanchaceae_O2K_Supplementary_v3/Data_S1/DataS1/RRR/OG0005338.filter.fas.treefile.pdf]

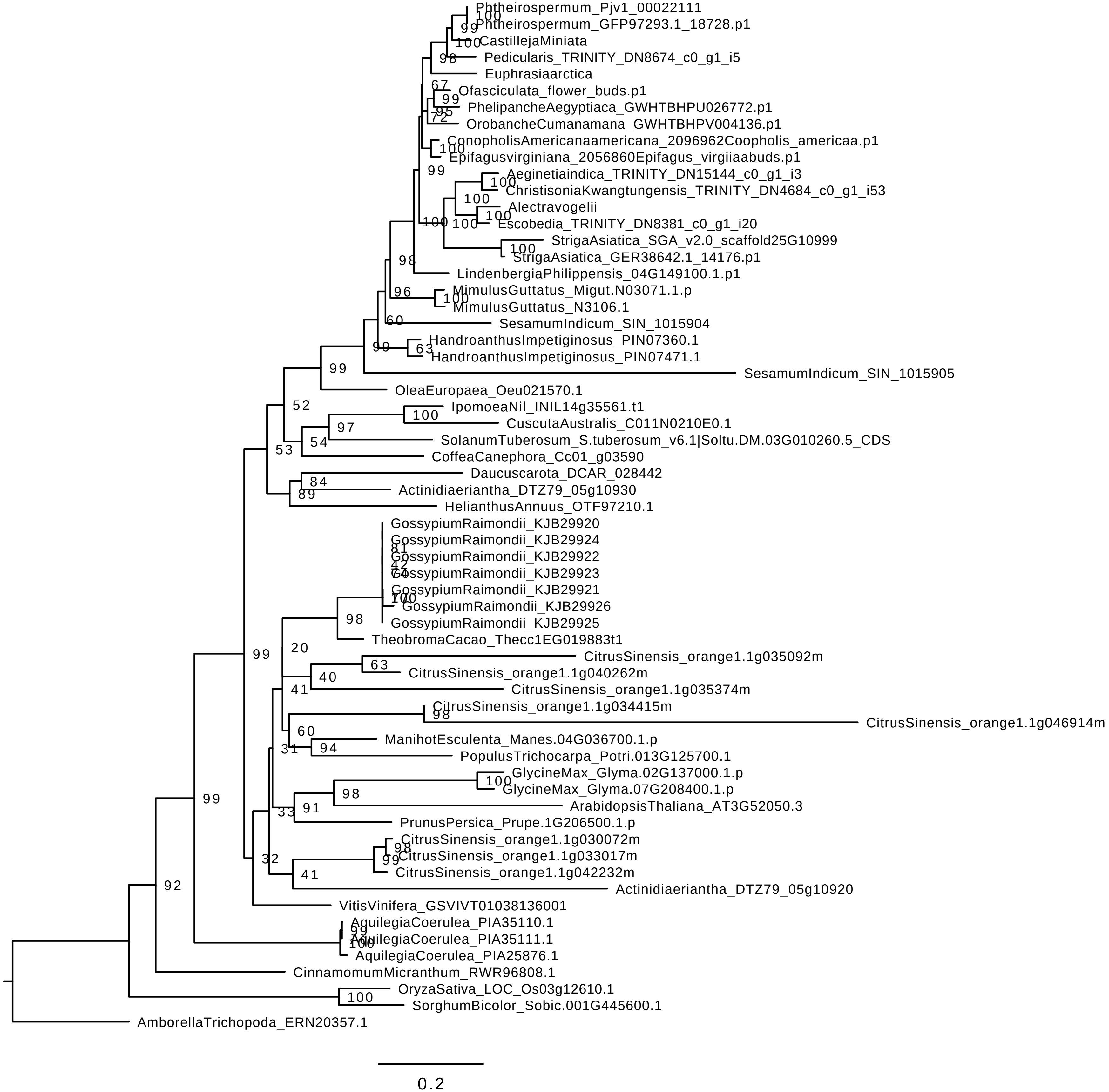

Phtheirospermum_Pjv1_00022111
Phtheirospermum_GFP97293.1_18728.p1
CastillejaMiniata
Pedicularis_TRINITY_DN8674_c0_g1_i5
Euphrasiaarctica
Ofasciculata_flower_buds.p1
PhelipancheAegyptiaca_GWHTBHPU026772.p1
OrobancheCumanamana_GWHTBHPV004136.p1
ConopholisAmericanaamericana_2096962Coopholis_americaa.p1
Epifagusvirginiana_2056860Epifagus_virgiiaabuds.p1
Aeginetiaindica_TRINITY_DN15144_c0_g1_i3
ChristisoniaKwangtungensis_TRINITY_DN4684_c0_g1_i53
Alectravogelii
Escobedia_TRINITY_DN8381_c0_g1_i20
StrigaAsiatica_SGA_v2.0_scaffold25G10999
StrigaAsiatica_GER38642.1_14176.p1
LindenbergiaPhilippensis_04G149100.1.p1
MimulusGuttatus_Migut.N03071.1.p
MimulusGuttatus_N3106.1
SesamumIndicum_SIN_1015904
HandroanthusImpetiginosus_PIN07360.1
HandroanthusImpetiginosus_PIN07471.1
SesamumIndicum_SIN_1015905
OleaEuropaea_Oeu021570.1
IpomoeaNil_INIL14g35561.t1
CuscutaAustralis_C011N0210E0.1
SolanumTuberosum_S.tuberosum_v6.1|Soltu.DM.03G010260.5_CDS
CoffeaCanephora_Cc01_g03590
Daucuscarota_DCAR_028442
Actinidiaeriantha_DTZ79_05g10930
HelianthusAnnuus_OTF97210.1
GossypiumRaimondii_KJB29920
GossypiumRaimondii_KJB29924
GossypiumRaimondii_KJB29922
GossypiumRaimondii_KJB29923
GossypiumRaimondii_KJB29921
GossypiumRaimondii_KJB29926
GossypiumRaimondii_KJB29925
TheobromaCacao_Thecc1EG019883t1
CitrusSinensis_orange1.1g035092m
CitrusSinensis_orange1.1g040262m
CitrusSinensis_orange1.1g035374m
CitrusSinensis_orange1.1g034415m
CitrusSinensis_orange1.1g046914m
ManihotEsculenta_Manes.04G036700.1.p
PopulusTrichocarpa_Potri.013G125700.1
GlycineMax_Glyma.02G137000.1.p
GlycineMax_Glyma.07G208400.1.p
ArabidopsisThaliana_AT3G52050.3
PrunusPersica_Prupe.1G206500.1.p
CitrusSinensis_orange1.1g030072m
CitrusSinensis_orange1.1g033017m
CitrusSinensis_orange1.1g042232m
Actinidiaeriantha_DTZ79_05g10920
VitisVinifera_GSVIVT01038136001
AquilegiaCoerulea_PIA35110.1
AquilegiaCoerulea_PIA35111.1
AquilegiaCoerulea_PIA25876.1
CinnamomumMicranthum_RWR96808.1
OryzaSativa_LOC_Os03g12610.1
SorghumBicolor_Sobic.001G445600.1
AmborellaTrichopoda_ERN20357.1
0.2
