# Supplementary material for: Altered Mitochondrial Respiration Is Associated With Loss of Nuclear‐Encoded OXPHOS Genes in Parasitic Broomrapes
Source: Ecol Evol. 2025 Jul 6;15(7):e71737. doi: 10.1002/ece3.71737 (PMC12230201; doi:10.1002/ece3.71737)
Supplement: Supplementary file 1 — Appendix S1. [file ECE3-15-e71737-s003.tgz › Orobanchaceae_O2K_Supplementary_v3/Data_S1/DataS1/RRR/OG0009232.filter.fas.treefile.pdf]

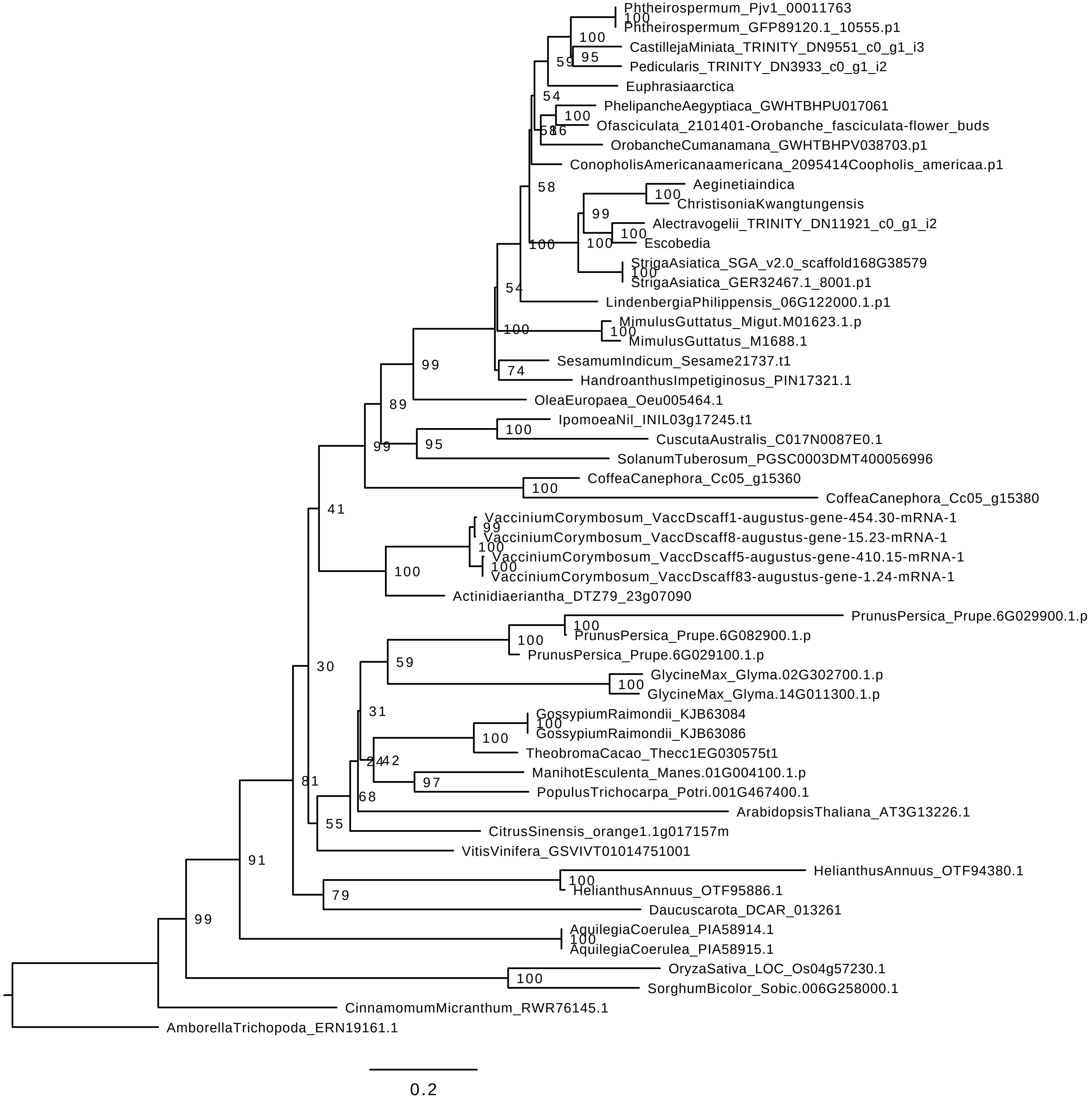
Phtheirospermum_Pjv1_00011763
Phtheirospermum_GFP89120.1_10555.p1
CastillejaMiniata_TRINITY_DN9551_c0_g1_i3
Pedicularis_TRINITY_DN3933_c0_g1_i2
Euphrasiaarctica
PhelipancheAegyptiaca_GWHTBHPU017061
Ofasciculata_2101401-Orobanche_fasciculata-flower_buds
OrobancheCumanamana_GWHTBHPV038703.p1
ConopholisAmericanaamericana_2095414Coopholis_americaa.p1
Aeginetiaindica
ChristisoniaKwangtungensis
Alectravogelii_TRINITY_DN11921_c0_g1_i2
Escobedia
StrigaAsiatica_SGA_v2.0_scaffold168G38579
StrigaAsiatica_GER32467.1_8001.p1
LindenbergiaPhilippensis_06G122000.1.p1
MimulusGuttatus_Migut.M01623.1.p
MimulusGuttatus_M1688.1
SesamumIndicum_Sesame21737.t1
HandroanthusImpetiginosus_PIN17321.1
OleaEuropaea_Oeu005464.1
IpomoeaNil_INIL03g17245.t1
CuscutaAustralis_C017N0087E0.1
SolanumTuberosum_PGSC0003DMT400056996
CoffeaCanephora_Cc05_g15360
CoffeaCanephora_Cc05_g15380
VacciniumCorymbosum_VaccDscaff1-augustus-gene-454.30-mRNA-1
VacciniumCorymbosum_VaccDscaff8-augustus-gene-15.23-mRNA-1
VacciniumCorymbosum_VaccDscaff5-augustus-gene-410.15-mRNA-1
VacciniumCorymbosum_VaccDscaff83-augustus-gene-1.24-mRNA-1
Actinidiaeriantha_DTZ79_23g07090
PrunusPersica_Prupe.6G029900.1.p
PrunusPersica_Prupe.6G082900.1.p
PrunusPersica_Prupe.6G029100.1.p
GlycineMax_Glyma.02G302700.1.p
GlycineMax_Glyma.14G011300.1.p
GossypiumRaimondii_KJB63084
GossypiumRaimondii_KJB63086
TheobromaCacao_Thecc1EG030575t1
ManihotEsculenta_Manes.01G004100.1.p
PopulusTrichocarpa_Potri.001G467400.1
ArabidopsisThaliana_AT3G13226.1
CitrusSinensis_orange1.1g017157m
VitisVinifera_GSVIVT01014751001
HelianthusAnnuus_OTF94380.1
HelianthusAnnuus_OTF95886.1
Daucuscarota_DCAR_013261
AquilegiaCoerulea_PIA58914.1
AquilegiaCoerulea_PIA58915.1
OryzaSativa_LOC_Os04g57230.1
SorghumBicolor_Sobic.006G258000.1
CinnamomumMicranthum_RWR76145.1
AmborellaTrichopoda_ERN19161.1
0.2
