# Supplementary material for: Altered Mitochondrial Respiration Is Associated With Loss of Nuclear‐Encoded OXPHOS Genes in Parasitic Broomrapes
Source: Ecol Evol. 2025 Jul 6;15(7):e71737. doi: 10.1002/ece3.71737 (PMC12230201; doi:10.1002/ece3.71737)
Supplement: Supplementary file 1 — Appendix S1. [file ECE3-15-e71737-s003.tgz › Orobanchaceae_O2K_Supplementary_v3/Data_S1/DataS1/RRR/OG0005829.filter.fas.treefile.pdf]

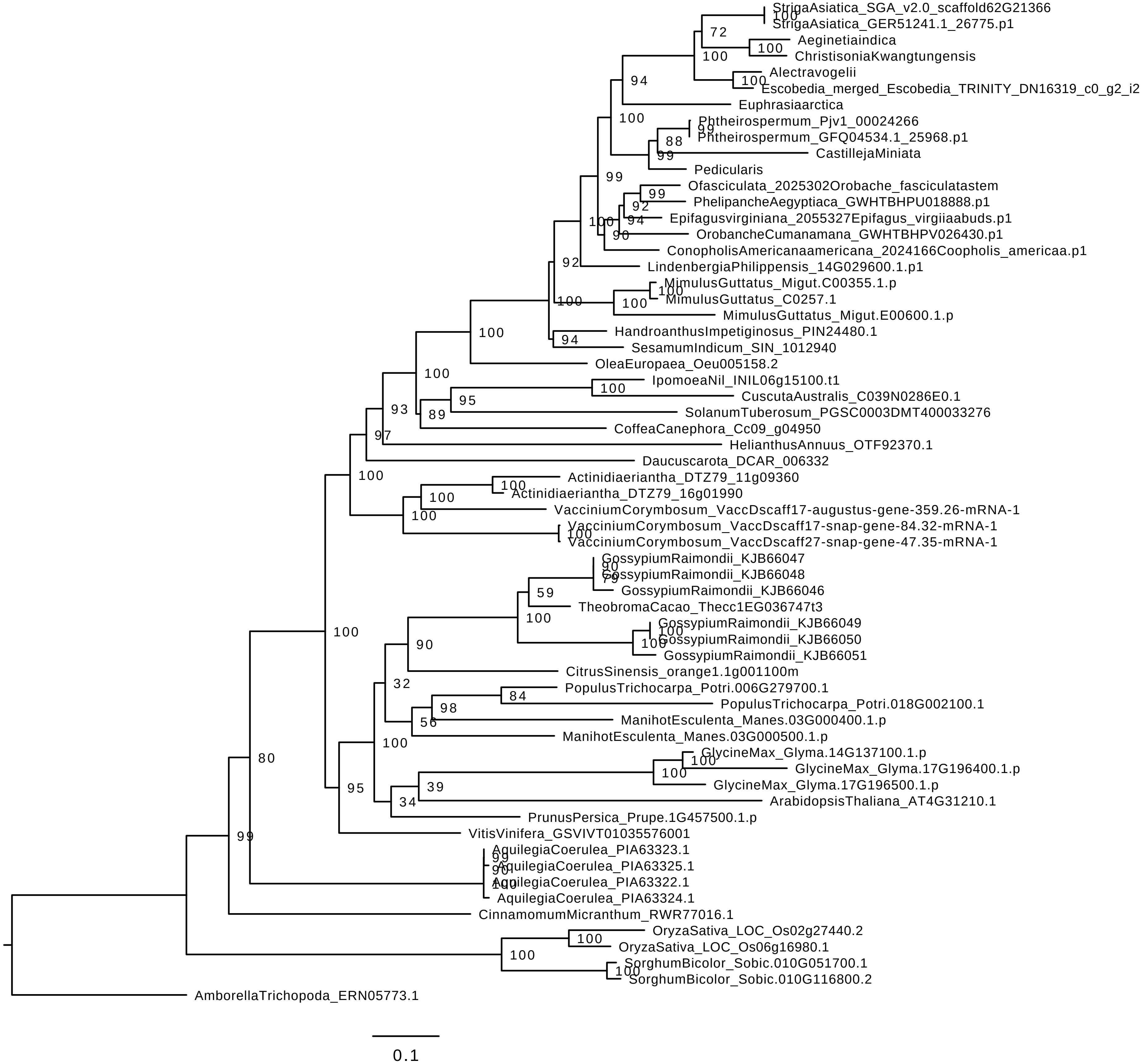
StrigaAsiatica_SGA_v2.0_scaffold62G21366
StrigaAsiatica_GER51241.1_26775.p1
Aeginetiaindica
ChristisoniaKwangtungensis
Alectravogelii
Escobedia_merged_Escobedia_TRINITY_DN16319_c0_g2_i2
Euphrasiaarctica
Phtheirospermum_Pjv1_00024266
Phtheirospermum_GFQ04534.1_25968.p1
CastillejaMiniata
Pedicularis
Ofasciculata_2025302Orobache_fasciculatastem
PhelipancheAegyptiaca_GWHTBHPU018888.p1
Epifagusvirginiana_2055327Epifagus_virgiiaabuds.p1
OrobancheCumanamana_GWHTBHPV026430.p1
ConopholisAmericanaamericana_2024166Coopholis_americaa.p1
LindenbergiaPhilippensis_14G029600.1.p1
MimulusGuttatus_Migut.C00355.1.p
MimulusGuttatus_C0257.1
MimulusGuttatus_Migut.E00600.1.p
HandroanthusImpetiginosus_PIN24480.1
SesamumIndicum_SIN_1012940
OleaEuropaea_Oeu005158.2
IpomoeaNil_INIL06g15100.t1
CuscutaAustralis_C039N0286E0.1
SolanumTuberosum_PGSC0003DMT400033276
CoffeaCanephora_Cc09_g04950
HelianthusAnnuus_OTF92370.1
Daucuscarota_DCAR_006332
Actinidiaerianthа_DTZ79_11g09360
Actinidiaeriantha_DTZ79_16g01990
VacciniumCorymbosum_VaccDscaff17-augustus-gene-359.26-mRNA-1
VacciniumCorymbosum_VaccDscaff17-snap-gene-84.32-mRNA-1
VacciniumCorymbosum_VaccDscaff27-snap-gene-47.35-mRNA-1
GossypiumRaimondii_KJB66047
GossypiumRaimondii_KJB66048
GossypiumRaimondii_KJB66046
TheobromaCacao_Thecc1EG036747t3
GossypiumRaimondii_KJB66049
GossypiumRaimondii_KJB66050
GossypiumRaimondii_KJB66051
CitrusSinensis_orange1.1g001100m
PopulusTrichocarpa_Potri.006G279700.1
PopulusTrichocarpa_Potri.018G002100.1
ManihotEsculenta_Manes.03G000400.1.p
ManihotEsculenta_Manes.03G000500.1.p
GlycineMax_Glyma.14G137100.1.p
GlycineMax_Glyma.17G196400.1.p
GlycineMax_Glyma.17G196500.1.p
ArabidopsisThaliana_AT4G31210.1
PrunusPersica_Prupe.1G457500.1.p
VitisVinifera_GSVIVT01035576001
AquilegiaCoerulea_PIA63323.1
AquilegiaCoerulea_PIA63325.1
AquilegiaCoerulea_PIA63322.1
AquilegiaCoerulea_PIA63324.1
CinnamomumMicranthum_RWR77016.1
OryzaSativa_LOC_Os02g27440.2
OryzaSativa_LOC_Os06g16980.1
SorghumBicolor_Sobic.010G051700.1
SorghumBicolor_Sobic.010G116800.2
AmborellaTrichopoda_ERN05773.1
0.1
