# Supplementary material for: Altered Mitochondrial Respiration Is Associated With Loss of Nuclear‐Encoded OXPHOS Genes in Parasitic Broomrapes
Source: Ecol Evol. 2025 Jul 6;15(7):e71737. doi: 10.1002/ece3.71737 (PMC12230201; doi:10.1002/ece3.71737)
Supplement: Supplementary file 1 — Appendix S1. [file ECE3-15-e71737-s003.tgz › Orobanchaceae_O2K_Supplementary_v3/Data_S1/DataS1/RRR/OG0009989.filter.fas.treefile.pdf]

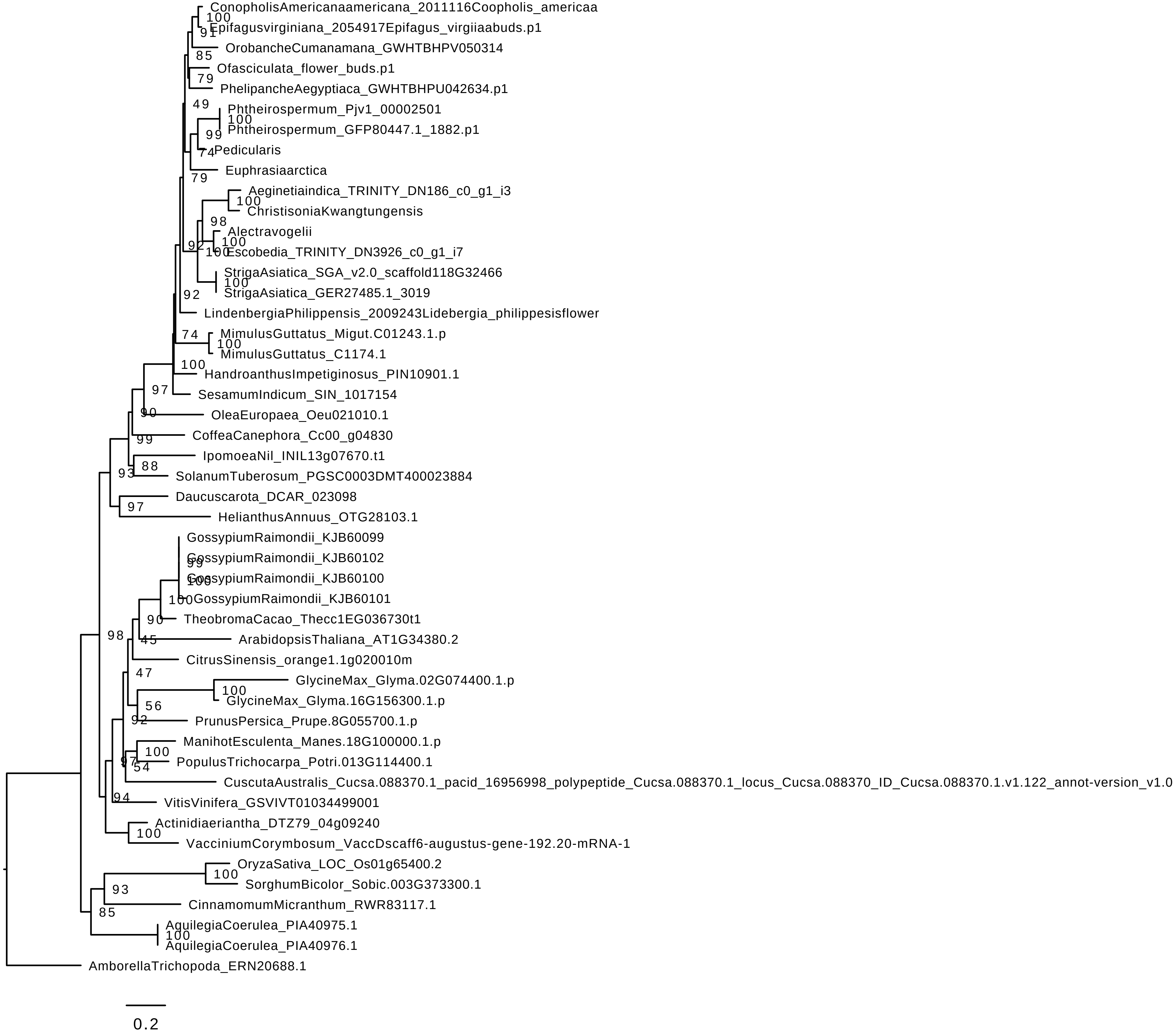
ConopholisAmericanaamericana_2011116Coopholis_americaa
Epifagusvirginiana_2054917Epifagus_virgiiaabuds.p1
OrobancheCumanamana_GWHTBHPV050314
Ofasciculata_flower_buds.p1
PhelipancheAegyptiaca_GWHTBHPU042634.p1
Phtheirospermum_Pjv1_00002501
Phtheirospermum_GFP80447.1_1882.p1
Pedicularis
Euphrasiaarctica
Aeginetiaindica_TRINITY_DN186_c0_g1_i3
ChristisoniaKwangtungensis
Alectravogelii
Escobedia_TRINITY_DN3926_c0_g1_i7
StrigaAsiatica_SGA_v2.0_scaffold118G32466
StrigaAsiatica_GER27485.1_3019
LindenbergiaPhilippensis_2009243Lidebergia_philippesisflower
MimulusGuttatus_Migut.C01243.1.p
MimulusGuttatus_C1174.1
HandroanthusImpetiginosus_PIN10901.1
SesamumIndicum_SIN_1017154
OleaEuropaea_Oeu021010.1
CoffeaCanephora_Cc00_g04830
IpomoeaNil_INIL13g07670.t1
SolanumTuberosum_PGSC0003DMT400023884
Daucuscarota_DCAR_023098
HelianthusAnnuus_OTG28103.1
GossypiumRaimondii_KJB60099
GossypiumRaimondii_KJB60102
GossypiumRaimondii_KJB60100
GossypiumRaimondii_KJB60101
TheobromaCacao_Thecc1EG036730t1
ArabidopsisThaliana_AT1G34380.2
CitrusSinensis_orange1.1g020010m
GlycineMax_Glyma.02G074400.1.p
GlycineMax_Glyma.16G156300.1.p
PrunusPersica_Prupe.8G055700.1.p
ManihotEsculenta_Manes.18G100000.1.p
PopulusTrichocarpa_Potri.013G114400.1
CuscutaAustralis_Cucsa.088370.1_pacid_16956998_polypeptide_Cucsa.088370.1_locus_Cucsa.088370_ID_Cucsa.088370.1.v1.122_annot-version_v1.0
VitisVinifera_GSVIVT01034499001
Actinidiaeriantha_DTZ79_04g09240
VacciniumCorymbosum_VaccDscaff6-augustus-gene-192.20-mRNA-1
OryzaSativa_LOC_Os01g65400.2
SorghumBicolor_Sobic.003G373300.1
CinnamomumMicranthum_RWR83117.1
AquilegiaCoerulea_PIA40975.1
AquilegiaCoerulea_PIA40976.1
AmborellaTrichopoda_ERN20688.1
0.2
