# Supplementary material for: Altered Mitochondrial Respiration Is Associated With Loss of Nuclear‐Encoded OXPHOS Genes in Parasitic Broomrapes
Source: Ecol Evol. 2025 Jul 6;15(7):e71737. doi: 10.1002/ece3.71737 (PMC12230201; doi:10.1002/ece3.71737)
Supplement: Supplementary file 1 — Appendix S1. [file ECE3-15-e71737-s003.tgz › Orobanchaceae_O2K_Supplementary_v3/Data_S1/DataS1/RRR/OG0001482_RECA2.filter.fas.treefile.pdf]

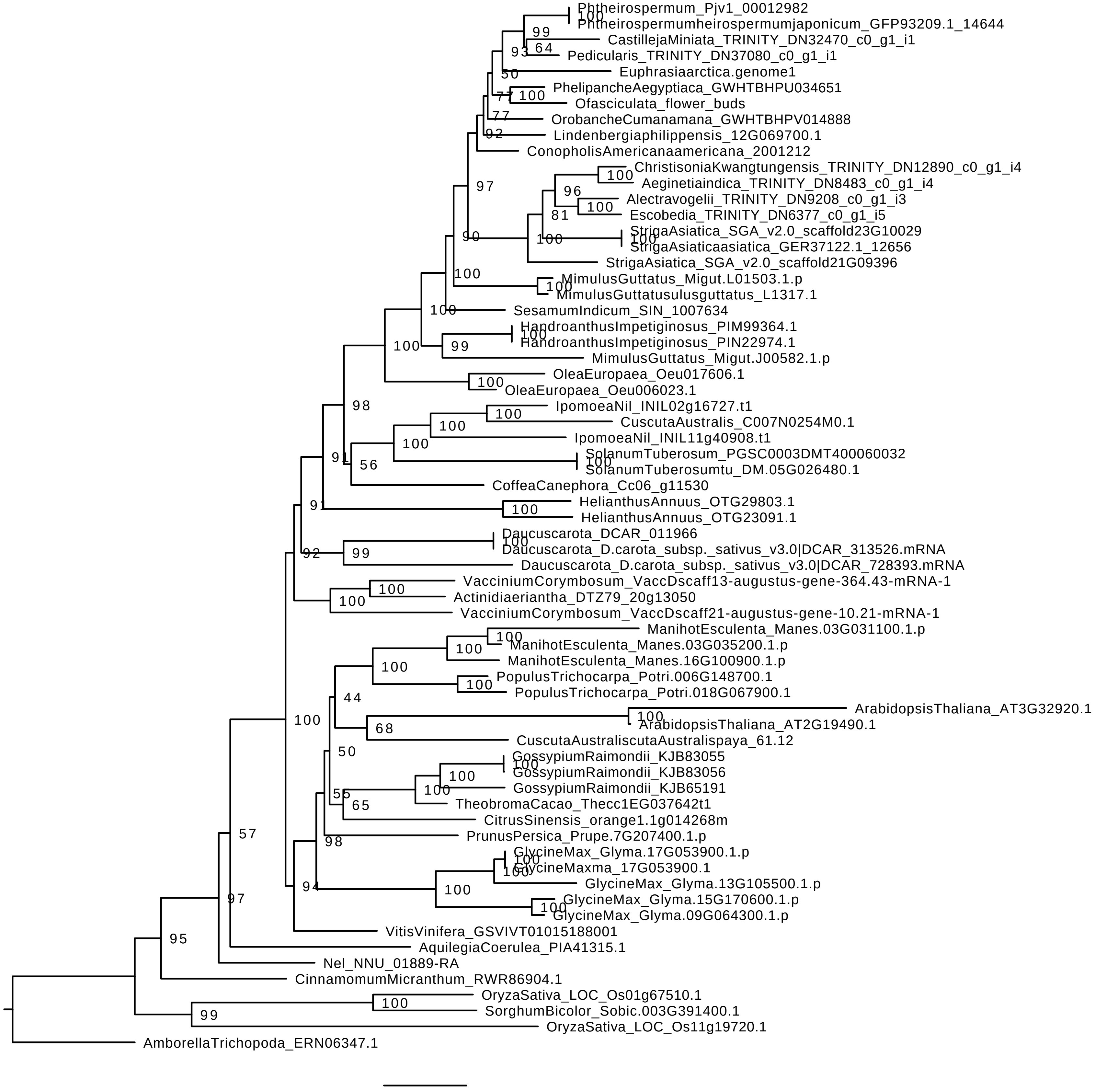
Phtheirospermum_Pjv1_00012982
Phtheirospermumheirospermumjaponicum_GFP93209.1_14644
CastillejaMiniata_TRINITY_DN32470_c0_g1_i1
Pedicularis_TRINITY_DN37080_c0_g1_i1
Euphrasiaarctica.genome1
PhelipancheAegyptiaca_GWHTBHPU034651
Ofasciculata_flower_buds
OrobancheCumanamana_GWHTBHPV014888
Lindenbergiaphilippensis_12G069700.1
ConopholisAmericanaamericana_2001212
ChristisoniaKwangtungensis_TRINITY_DN12890_c0_g1_i4
Aeginetiaindica_TRINITY_DN8483_c0_g1_i4
Alectravogelii_TRINITY_DN9208_c0_g1_i3
Escobedia_TRINITY_DN6377_c0_g1_i5
StrigaAsiatica_SGA_v2.0_scaffold23G10029
StrigaAsiaticaasiatica_GER37122.1_12656
StrigaAsiatica_SGA_v2.0_scaffold21G09396
MimulusGuttatus_Migut.L01503.1.p
MimulusGuttatusulusguttatus_L1317.1
SesamumIndicum_SIN_1007634
HandroanthusImpetiginosus_PIM99364.1
HandroanthusImpetiginosus_PIN22974.1
MimulusGuttatus_Migut.J00582.1.p
OleaEuropaea_Oeu017606.1
OleaEuropaea_Oeu006023.1
IpomoeaNil_INIL02g16727.t1
CuscutaAustralis_C007N0254M0.1
IpomoeaNil_INIL11g40908.t1
SolanumTuberosum_PGSC0003DMT400060032
SolanumTuberosumtu_DM.05G026480.1
CoffeaCanephora_Cc06_g11530
HelianthusAnnuus_OTG29803.1
HelianthusAnnuus_OTG23091.1
Daucuscarota_DCAR_011966
Daucuscarota_D.carota_subsp._sativus_v3.0|DCAR_313526.mRNA
Daucuscarota_D.carota_subsp._sativus_v3.0|DCAR_728393.mRNA
VacciniumCorymbosum_VaccDscaff13-augustus-gene-364.43-mRNA-1
Actinidiaerianthа_DTZ79_20g13050
VacciniumCorymbosum_VaccDscaff21-augustus-gene-10.21-mRNA-1
ManihotEsculenta_Manes.03G031100.1.p
ManihotEsculenta_Manes.03G035200.1.p
ManihotEsculenta_Manes.16G100900.1.p
PopulusTrichocarpa_Potri.006G148700.1
PopulusTrichocarpa_Potri.018G067900.1
ArabidopsisThaliana_AT3G32920.1
ArabidopsisThaliana_AT2G19490.1
CuscutaAustraliscutaAustralispaya_61.12
GossypiumRaimondii_KJB83055
GossypiumRaimondii_KJB83056
GossypiumRaimondii_KJB65191
TheobromaCacao_Thecc1EG037642t1
CitrusSinensis_orange1.1g014268m
PrunusPersica_Prupe.7G207400.1.p
GlycineMax_Glyma.17G053900.1.p
GlycineMaxma_17G053900.1
GlycineMax_Glyma.13G105500.1.p
GlycineMax_Glyma.15G170600.1.p
GlycineMax_Glyma.09G064300.1.p
VitisVinifera_GSVIVT01015188001
AquilegiaCoerulea_PIA41315.1
Nel_NNU_01889-RA
CinnamomumMicranthum_RWR86904.1
OryzaSativa_LOC_Os01g67510.1
SorghumBicolor_Sobic.003G391400.1
OryzaSativa_LOC_Os11g19720.1
AmborellaTrichopoda_ERN06347.1
0.1
