# Supplementary material for: Altered Mitochondrial Respiration Is Associated With Loss of Nuclear‐Encoded OXPHOS Genes in Parasitic Broomrapes
Source: Ecol Evol. 2025 Jul 6;15(7):e71737. doi: 10.1002/ece3.71737 (PMC12230201; doi:10.1002/ece3.71737)
Supplement: Supplementary file 1 — Appendix S1. [file ECE3-15-e71737-s003.tgz › Orobanchaceae_O2K_Supplementary_v3/Data_S1/DataS1/RRR/OG0001016.filter.fas.treefile.pdf]

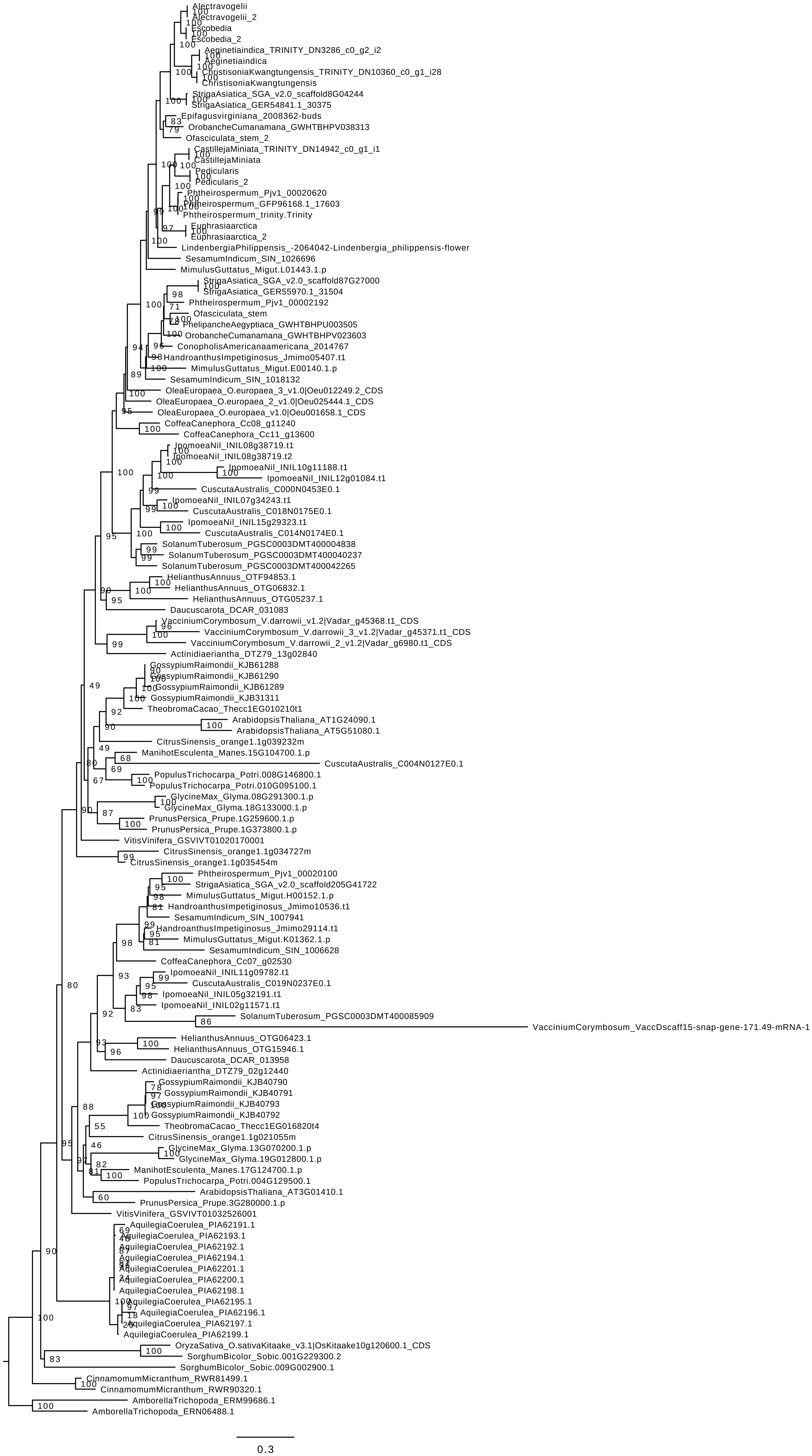
Alectravogelii
Alectravogelii_2
Escobedia
Escobedia_2
Aeginetiaindica_TRINITY_DN3286_c0_g2_i2
Aeginetiaindica
ChristisoniaKwangtungensis_TRINITY_DN10360_c0_g1_i28
ChristisoniaKwangtungensis
StrigaAsiatica_SGA_v2.0_scaffold8G04244
StrigaAsiatica_GER54841.1_30375
Epifagusvirginiana_2008362-buds
OrobancheCumanamana_GWHTBHPV038313
Ofasciculata_stem_2
CastillejaMiniata_TRINITY_DN14942_c0_g1_i1
CastillejaMiniata
Pedicularis
Pedicularis_2
Phtheirospermum_Pjv1_00020620
Phtheirospermum_GFP96168.1_17603
Phtheirospermum_trinity.Trinity
Euphrasiaarctica
Euphrasiaarctica_2
LindenbergiaPhilippensis_-2064042-Lindenbergia_philippensis-flower
SesamumIndicum_SIN_1026696
MimulusGuttatus_Migut.L01443.1.p
StrigaAsiatica_SGA_v2.0_scaffold87G27000
StrigaAsiatica_GER55970.1_31504
Phtheirospermum_Pjv1_00002192
Ofasciculata_stem
PhelipancheAegyptiaca_GWHTBHPU003505
OrobancheCumanamana_GWHTBHPV023603
ConopholisAmericanaamericana_2014767
HandroanthusImpetiginosus_Jmimo05407.t1
MimulusGuttatus_Migut.E00140.1.p
SesamumIndicum_SIN_1018132
OleaEuropaea_O.europaea_3_v1.0|Oeu012249.2_CDS
OleaEuropaea_O.europaea_2_v1.0|Oeu025444.1_CDS
OleaEuropaea_O.europaea_v1.0|Oeu001658.1_CDS
CoffeaCanephora_Cc08_g11240
CoffeaCanephora_Cc11_g13600
IpomoeaNil_INIL08g38719.t1
IpomoeaNil_INIL08g38719.t2
IpomoeaNil_INIL10g11188.t1
IpomoeaNil_INIL12g01084.t1
CuscutaAustralis_C000N0453E0.1
IpomoeaNil_INIL07g34243.t1
CuscutaAustralis_C018N0175E0.1
IpomoeaNil_INIL15g29323.t1
CuscutaAustralis_C014N0174E0.1
SolanumTuberosum_PGSC0003DMT400004838
SolanumTuberosum_PGSC0003DMT400040237
SolanumTuberosum_PGSC0003DMT400042265
HelianthusAnnuus_OTF94853.1
HelianthusAnnuus_OTG06832.1
HelianthusAnnuus_OTG05237.1
Daucuscarota_DCAR_031083
VacciniumCorymbosum_V.darrowii_v1.2|Vadar_g45368.t1_CDS
VacciniumCorymbosum_V.darrowii_3_v1.2|Vadar_g45371.t1_CDS
VacciniumCorymbosum_V.darrowii_2_v1.2|Vadar_g6980.t1_CDS
Actinidiaeriantha_DTZ79_13g02840
GossypiumRaimondii_KJB61288
GossypiumRaimondii_KJB61290
GossypiumRaimondii_KJB61289
GossypiumRaimondii_KJB31311
TheobromaCacao_Thecc1EG010210t1
ArabidopsisThaliana_AT1G24090.1
ArabidopsisThaliana_AT5G51080.1
CitrusSinensis_orange1.1g039232m
ManihotEsculenta_Manes.15G104700.1.p
CuscutaAustralis_C004N0127E0.1
PopulusTrichocarpa_Potri.008G146800.1
PopulusTrichocarpa_Potri.010G095100.1
GlycineMax_Glyma.08G291300.1.p
GlycineMax_Glyma.18G133000.1.p
PrunusPersica_Prupe.1G259600.1.p
PrunusPersica_Prupe.1G373800.1.p
VitisVinifera_GSVIVT01020170001
CitrusSinensis_orange1.1g034727m
CitrusSinensis_orange1.1g035454m
Phtheirospermum_Pjv1_00020100
StrigaAsiatica_SGA_v2.0_scaffold205G41722
MimulusGuttatus_Migut.H00152.1.p
HandroanthusImpetiginosus_Jmimo10536.t1
SesamumIndicum_SIN_1007941
HandroanthusImpetiginosus_Jmimo29114.t1
MimulusGuttatus_Migut.K01362.1.p
SesamumIndicum_SIN_1006628
CoffeaCanephora_Cc07_g02530
IpomoeaNil_INIL11g09782.t1
CuscutaAustralis_C019N0237E0.1
IpomoeaNil_INIL05g32191.t1
IpomoeaNil_INIL02g11571.t1
SolanumTuberosum_PGSC0003DMT400085909
VacciniumCorymbosum_VaccDscaff15-snap-gene-171.49-mRNA-1
HelianthusAnnuus_OTG06423.1
HelianthusAnnuus_OTG15946.1
Daucuscarota_DCAR_013958
Actinidiaeriantha_DTZ79_02g12440
GossypiumRaimondii_KJB40790
GossypiumRaimondii_KJB40791
GossypiumRaimondii_KJB40793
GossypiumRaimondii_KJB40792
TheobromaCacao_Thecc1EG016820t4
CitrusSinensis_orange1.1g021055m
GlycineMax_Glyma.13G070200.1.p
GlycineMax_Glyma.19G012800.1.p
ManihotEsculenta_Manes.17G124700.1.p
PopulusTrichocarpa_Potri.004G129500.1
ArabidopsisThaliana_AT3G01410.1
PrunusPersica_Prupe.3G280000.1.p
VitisVinifera_GSVIVT01032526001
AquilegiaCoerulea_PIA62191.1
AquilegiaCoerulea_PIA62193.1
AquilegiaCoerulea_PIA62192.1
AquilegiaCoerulea_PIA62194.1
AquilegiaCoerulea_PIA62201.1
AquilegiaCoerulea_PIA62200.1
AquilegiaCoerulea_PIA62198.1
AquilegiaCoerulea_PIA62195.1
AquilegiaCoerulea_PIA62196.1
AquilegiaCoerulea_PIA62197.1
AquilegiaCoerulea_PIA62199.1
OryzaSativa_O.sativaKitaake_v3.1|OsKitaake10g120600.1_CDS
SorghumBicolor_Sobic.001G229300.2
SorghumBicolor_Sobic.009G002900.1
CinnamomumMicranthum_RWR81499.1
CinnamomumMicranthum_RWR90320.1
AmborellaTrichopoda_ERM99686.1
AmborellaTrichopoda_ERN06488.1
0.3
