# Supplementary material for: Altered Mitochondrial Respiration Is Associated With Loss of Nuclear‐Encoded OXPHOS Genes in Parasitic Broomrapes
Source: Ecol Evol. 2025 Jul 6;15(7):e71737. doi: 10.1002/ece3.71737 (PMC12230201; doi:10.1002/ece3.71737)
Supplement: Supplementary file 1 — Appendix S1. [file ECE3-15-e71737-s003.tgz › Orobanchaceae_O2K_Supplementary_v3/Data_S1/DataS1/CIV/OG0001581.filter.fas.treefile.pdf]

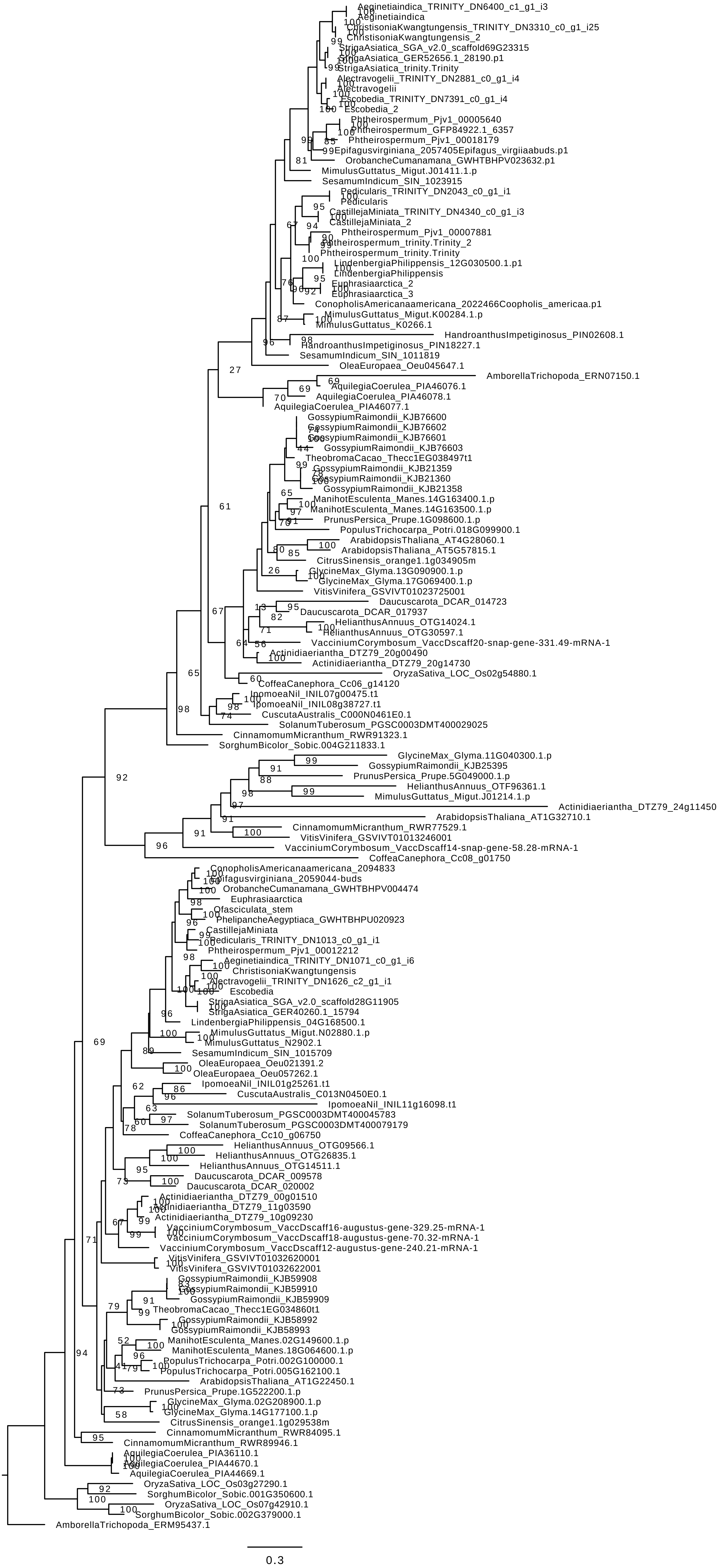

Aeginetiaindica_TRINITY_DN6400_c1_g1_i3
Aeginetiaindica
ChristisoniaKwangtungensis_TRINITY_DN3310_c0_g1_i25
ChristisoniaKwangtungensis_2
StrigaAsiatica_SGA_v2.0_scaffold69G23315
StrigaAsiatica_GER52656.1_28190.p1
StrigaAsiatica_trinity.Trinity
Alectravogelii_TRINITY_DN2881_c0_g1_i4
Alectravogelii
Escobedia_TRINITY_DN7391_c0_g1_i4
Escobedia_2
Phtheirospermum_Pjv1_00005640
Phtheirospermum_GFP84922.1_6357
Phtheirospermum_Pjv1_00018179
Epifagusvirginiana_2057405Epifagus_virgiiaabuds.p1
OrobancheCumanamana_GWHTBHPV023632.p1
MimulusGuttatus_Migut.J01411.1.p
SesamumIndicum_SIN_1023915
Pedicularis_TRINITY_DN2043_c0_g1_i1
Pedicularis
CastillejaMiniata_TRINITY_DN4340_c0_g1_i3
CastillejaMiniata_2
Phtheirospermum_Pjv1_00007881
Phtheirospermum_trinity.Trinity_2
Phtheirospermum_trinity.Trinity
LindenbergiaPhilippensis_12G030500.1.p1
LindenbergiaPhilippensis
Euphrasiaarctica_2
Euphrasiaarctica_3
ConopholisAmericanaamericana_2022466Coopholis_americaa.p1
MimulusGuttatus_Migut.K00284.1.p
MimulusGuttatus_K0266.1
HandroanthusImpetiginosus_PIN02608.1
HandroanthusImpetiginosus_PIN18227.1
SesamumIndicum_SIN_1011819
OleaEuropaea_Oeu045647.1
AmborellaTrichopoda_ERN07150.1
AquilegiaCoerulea_PIA46076.1
AquilegiaCoerulea_PIA46078.1
AquilegiaCoerulea_PIA46077.1
GossypiumRaimondii_KJB76600
GossypiumRaimondii_KJB76602
GossypiumRaimondii_KJB76601
GossypiumRaimondii_KJB76603
TheobromaCacao_Thecc1EG038497t1
GossypiumRaimondii_KJB21359
GossypiumRaimondii_KJB21360
GossypiumRaimondii_KJB21358
ManihotEsculenta_Manes.14G163400.1.p
ManihotEsculenta_Manes.14G163500.1.p
PrunusPersica_Prupe.1G098600.1.p
PopulusTrichocarpa_Potri.018G099900.1
ArabidopsisThaliana_AT4G28060.1
ArabidopsisThaliana_AT5G57815.1
CitrusSinensis_orange1.1g034905m
GlycineMax_Glyma.13G090900.1.p
GlycineMax_Glyma.17G069400.1.p
VitisVinifera_GSVIVT01023725001
Daucuscarota_DCAR_014723
Daucuscarota_DCAR_017937
HelianthusAnnuus_OTG14024.1
HelianthusAnnuus_OTG30597.1
VacciniumCorymbosum_VaccDscaff20-snap-gene-331.49-mRNA-1
Actinidiaeriantha_DTZ79_20g00490
Actinidiaeriantha_DTZ79_20g14730
OryzaSativa_LOC_Os02g54880.1
CoffeaCanephora_Cc06_g14120
IpomoeaNil_INIL07g00475.t1
IpomoeaNil_INIL08g38727.t1
CuscutaAustralis_C000N0461E0.1
SolanumTuberosum_PGSC0003DMT400029025
CinnamomumMicranthum_RWR91323.1
SorghumBicolor_Sobic.004G211833.1
GlycineMax_Glyma.11G040300.1.p
GossypiumRaimondii_KJB25395
PrunusPersica_Prupe.5G049000.1.p
HelianthusAnnuus_OTF96361.1
MimulusGuttatus_Migut.J01214.1.p
Actinidiaeriantha_DTZ79_24g11450
ArabidopsisThaliana_AT1G32710.1
CinnamomumMicranthum_RWR77529.1
VitisVinifera_GSVIVT01013246001
VacciniumCorymbosum_VaccDscaff14-snap-gene-58.28-mRNA-1
CoffeaCanephora_Cc08_g01750
ConopholisAmericanaamericana_2094833
Epifagusvirginiana_2059044-buds
OrobancheCumanamana_GWHTBHPV004474
Euphrasiaarctica
Ofasciculata_stem
PhelipancheAegyptiaca_GWHTBHPU020923
CastillejaMiniata
Pedicularis_TRINITY_DN1013_c0_g1_i1
Phtheirospermum_Pjv1_00012212
Aeginetiaindica_TRINITY_DN1071_c0_g1_i6
ChristisoniaKwangtungensis
Alectravogelii_TRINITY_DN1626_c2_g1_i1
Escobedia
StrigaAsiatica_SGA_v2.0_scaffold28G11905
StrigaAsiatica_GER40260.1_15794
LindenbergiaPhilippensis_04G168500.1
MimulusGuttatus_Migut.N02880.1.p
MimulusGuttatus_N2902.1
SesamumIndicum_SIN_1015709
OleaEuropaea_Oeu021391.2
OleaEuropaea_Oeu057262.1
IpomoeaNil_INIL01g25261.t1
CuscutaAustralis_C013N0450E0.1
IpomoeaNil_INIL11g16098.t1
SolanumTuberosum_PGSC0003DMT400045783
SolanumTuberosum_PGSC0003DMT400079179
CoffeaCanephora_Cc10_g06750
HelianthusAnnuus_OTG09566.1
HelianthusAnnuus_OTG26835.1
HelianthusAnnuus_OTG14511.1
Daucuscarota_DCAR_009578
Daucuscarota_DCAR_020002
Actinidiaeriantha_DTZ79_00g01510
Actinidiaeriantha_DTZ79_11g03590
Actinidiaeriantha_DTZ79_10g09230
VacciniumCorymbosum_VaccDscaff16-augustus-gene-329.25-mRNA-1
VacciniumCorymbosum_VaccDscaff18-augustus-gene-70.32-mRNA-1
VacciniumCorymbosum_VaccDscaff12-augustus-gene-240.21-mRNA-1
VitisVinifera_GSVIVT01032620001
VitisVinifera_GSVIVT01032622001
GossypiumRaimondii_KJB59908
GossypiumRaimondii_KJB59910
GossypiumRaimondii_KJB59909
TheobromaCacao_Thecc1EG034860t1
GossypiumRaimondii_KJB58992
GossypiumRaimondii_KJB58993
ManihotEsculenta_Manes.02G149600.1.p
ManihotEsculenta_Manes.18G064600.1.p
PopulusTrichocarpa_Potri.002G100000.1
PopulusTrichocarpa_Potri.005G162100.1
ArabidopsisThaliana_AT1G22450.1
PrunusPersica_Prupe.1G522200.1.p
GlycineMax_Glyma.02G208900.1.p
GlycineMax_Glyma.14G177100.1.p
CitrusSinensis_orange1.1g029538m
CinnamomumMicranthum_RWR84095.1
CinnamomumMicranthum_RWR89946.1
AquilegiaCoerulea_PIA36110.1
AquilegiaCoerulea_PIA44670.1
AquilegiaCoerulea_PIA44669.1
OryzaSativa_LOC_Os03g27290.1
SorghumBicolor_Sobic.001G350600.1
OryzaSativa_LOC_Os07g42910.1
SorghumBicolor_Sobic.002G379000.1
AmborellaTrichopoda_ERM95437.1
0.3
