# Supplementary material for: Altered Mitochondrial Respiration Is Associated With Loss of Nuclear‐Encoded OXPHOS Genes in Parasitic Broomrapes
Source: Ecol Evol. 2025 Jul 6;15(7):e71737. doi: 10.1002/ece3.71737 (PMC12230201; doi:10.1002/ece3.71737)
Supplement: Supplementary file 1 — Appendix S1. [file ECE3-15-e71737-s003.tgz › Orobanchaceae_O2K_Supplementary_v3/Data_S1/DataS1/CIV/OG0004221.filter.fas.treefile.pdf]

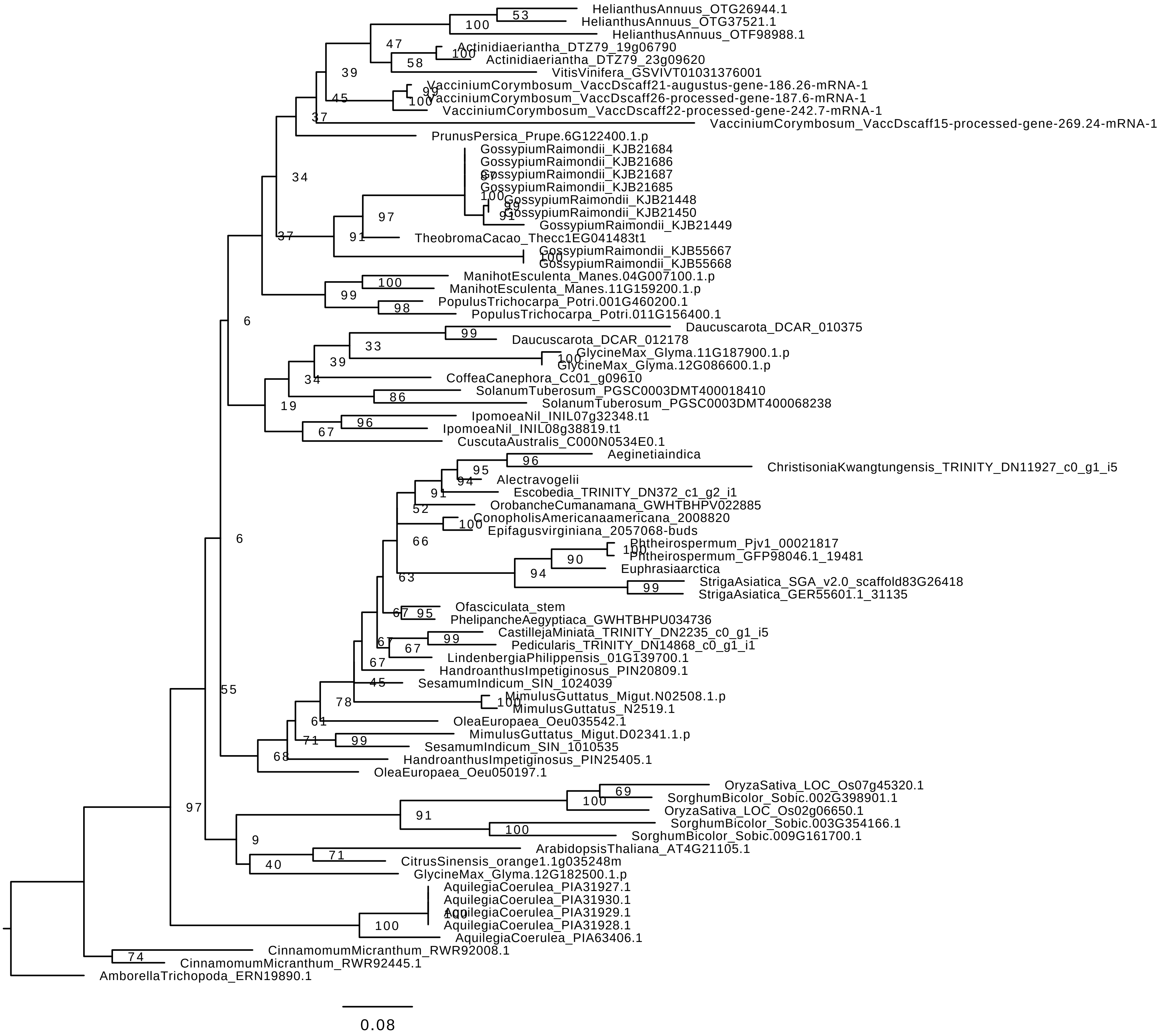
HelianthusAnnuus_OTG26944.1
HelianthusAnnuus_OTG37521.1
HelianthusAnnuus_OTF98988.1
Actinidiaeriantha_DTZ79_19g06790
Actinidiaeriantha_DTZ79_23g09620
VitisVinifera_GSVIVT01031376001
VacciniumCorymbosum_VaccDscaff21-augustus-gene-186.26-mRNA-1
VacciniumCorymbosum_VaccDscaff26-processed-gene-187.6-mRNA-1
VacciniumCorymbosum_VaccDscaff22-processed-gene-242.7-mRNA-1
VacciniumCorymbosum_VaccDscaff15-processed-gene-269.24-mRNA-1
PrunusPersica_Prupe.6G122400.1.p
GossypiumRaimondii_KJB21684
GossypiumRaimondii_KJB21686
GossypiumRaimondii_KJB21687
GossypiumRaimondii_KJB21685
GossypiumRaimondii_KJB21448
GossypiumRaimondii_KJB21450
GossypiumRaimondii_KJB21449
TheobromaCacao_Thecc1EG041483t1
GossypiumRaimondii_KJB55667
GossypiumRaimondii_KJB55668
ManihotEsculenta_Manes.04G007100.1.p
ManihotEsculenta_Manes.11G159200.1.p
PopulusTrichocarpa_Potri.001G460200.1
PopulusTrichocarpa_Potri.011G156400.1
Daucuscarota_DCAR_010375
Daucuscarota_DCAR_012178
GlycineMax_Glyma.11G187900.1.p
GlycineMax_Glyma.12G086600.1.p
CoffeaCanephora_Cc01_g09610
SolanumTuberosum_PGSC0003DMT400018410
SolanumTuberosum_PGSC0003DMT400068238
IpomoeaNil_INIL07g32348.t1
IpomoeaNil_INIL08g38819.t1
CuscutaAustralis_C000N0534E0.1
Aeginetiaindica
ChristisoniaKwangtungensis_TRINITY_DN11927_c0_g1_i5
Alectravogelii
Escobedia_TRINITY_DN372_c1_g2_i1
OrobancheCumanamana_GWHTBHPV022885
ConopholisAmericanaamericana_2008820
Epifagusvirginiana_2057068-buds
Phtheirospermum_Pjv1_00021817
Phtheirospermum_GFP98046.1_19481
Euphrasiaarctica
StrigaAsiatica_SGA_v2.0_scaffold83G26418
StrigaAsiatica_GER55601.1_31135
Ofasciculata_stem
PhelipancheAegyptiaca_GWHTBHPU034736
CastillejaMiniata_TRINITY_DN2235_c0_g1_i5
Pedicularis_TRINITY_DN14868_c0_g1_i1
LindenbergiaPhilippensis_01G139700.1
HandroanthusImpetiginosus_PIN20809.1
SesamumIndicum_SIN_1024039
MimulusGuttatus_Migut.N02508.1.p
MimulusGuttatus_N2519.1
OleaEuropaea_Oeu035542.1
MimulusGuttatus_Migut.D02341.1.p
SesamumIndicum_SIN_1010535
HandroanthusImpetiginosus_PIN25405.1
OleaEuropaea_Oeu050197.1
OryzaSativa_LOC_Os07g45320.1
SorghumBicolor_Sobic.002G398901.1
OryzaSativa_LOC_Os02g06650.1
SorghumBicolor_Sobic.003G354166.1
SorghumBicolor_Sobic.009G161700.1
ArabidopsisThaliana_AT4G21105.1
CitrusSinensis_orange1.1g035248m
GlycineMax_Glyma.12G182500.1.p
AquilegiaCoerulea_PIA31927.1
AquilegiaCoerulea_PIA31930.1
AquilegiaCoerulea_PIA31929.1
AquilegiaCoerulea_PIA31928.1
AquilegiaCoerulea_PIA63406.1
CinnamomumMicranthum_RWR92008.1
CinnamomumMicranthum_RWR92445.1
AmborellaTrichopoda_ERN19890.1
0.08
