# Supplementary material for: Altered Mitochondrial Respiration Is Associated With Loss of Nuclear‐Encoded OXPHOS Genes in Parasitic Broomrapes
Source: Ecol Evol. 2025 Jul 6;15(7):e71737. doi: 10.1002/ece3.71737 (PMC12230201; doi:10.1002/ece3.71737)
Supplement: Supplementary file 1 — Appendix S1. [file ECE3-15-e71737-s003.tgz › Orobanchaceae_O2K_Supplementary_v3/Data_S1/DataS1/CIV/OG0006041.filter.fas.treefile.pdf]

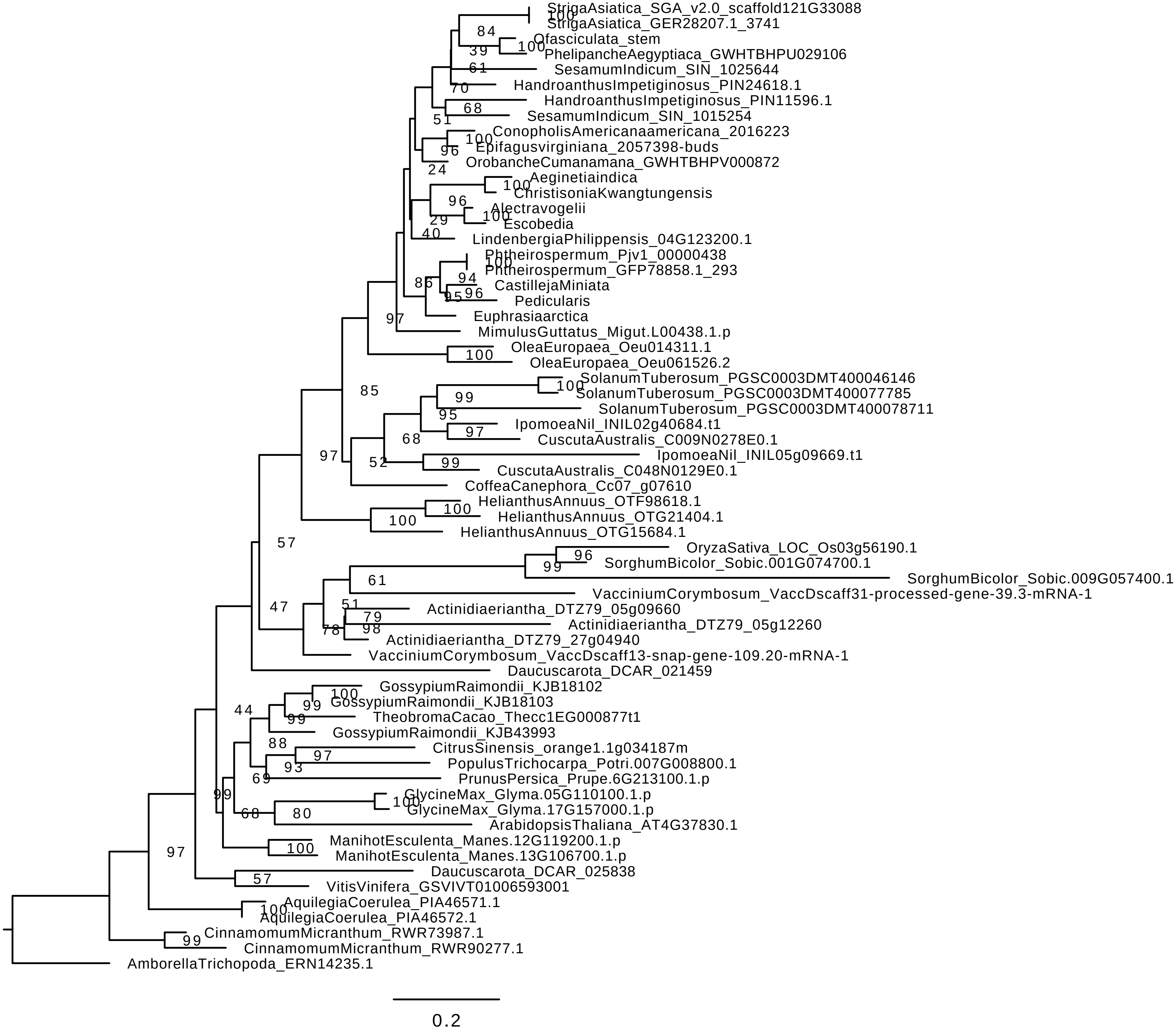
StrigaAsiatica_SGA_v2.0_scaffold121G33088
StrigaAsiatica_GER28207.1_3741
Ofasciculata_stem
PhelipancheAegyptiaca_GWHTBHPU029106
SesamumIndicum_SIN_1025644
HandroanthusImpetiginosus_PIN24618.1
HandroanthusImpetiginosus_PIN11596.1
SesamumIndicum_SIN_1015254
ConopholisAmericanaamericana_2016223
Epifagusvirginiana_2057398-buds
OrobancheCumanamana_GWHTBHPV000872
Aeginetiaindica
ChristisoniaKwangtungensis
Alectravogelii
Escobedia
LindenbergiaPhilippensis_04G123200.1
Phtheirospermum_Pjv1_00000438
Phtheirospermum_GFP78858.1_293
CastillejaMiniata
Pedicularis
Euphrasiaarctica
MimulusGuttatus_Migut.L00438.1.p
OleaEuropaea_Oeu014311.1
OleaEuropaea_Oeu061526.2
SolanumTuberosum_PGSC0003DMT400046146
SolanumTuberosum_PGSC0003DMT400077785
SolanumTuberosum_PGSC0003DMT400078711
IpomoeaNil_INIL02g40684.t1
CuscutaAustralis_C009N0278E0.1
IpomoeaNil_INIL05g09669.t1
CuscutaAustralis_C048N0129E0.1
CoffeaCanephora_Cc07_g07610
HelianthusAnnuus_OTF98618.1
HelianthusAnnuus_OTG21404.1
HelianthusAnnuus_OTG15684.1
OryzaSativa_LOC_Os03g56190.1
SorghumBicolor_Sobic.001G074700.1
SorghumBicolor_Sobic.009G057400.1
VacciniumCorymbosum_VaccDscaff31-processed-gene-39.3-mRNA-1
Actinidiaeriantha_DTZ79_05g09660
Actinidiaeriantha_DTZ79_05g12260
Actinidiaeriantha_DTZ79_27g04940
VacciniumCorymbosum_VaccDscaff13-snap-gene-109.20-mRNA-1
Daucuscarota_DCAR_021459
GossypiumRaimondii_KJB18102
GossypiumRaimondii_KJB18103
TheobromaCacao_Thecc1EG000877t1
GossypiumRaimondii_KJB43993
CitrusSinensis_orange1.1g034187m
PopulusTrichocarpa_Potri.007G008800.1
PrunusPersica_Prupe.6G213100.1.p
GlycineMax_Glyma.05G110100.1.p
GlycineMax_Glyma.17G157000.1.p
ArabidopsisThaliana_AT4G37830.1
ManihotEsculenta_Manes.12G119200.1.p
ManihotEsculenta_Manes.13G106700.1.p
Daucuscarota_DCAR_025838
VitisVinifera_GSVIVT01006593001
AquilegiaCoerulea_PIA46571.1
AquilegiaCoerulea_PIA46572.1
CinnamomumMicranthum_RWR73987.1
CinnamomumMicranthum_RWR90277.1
AmborellaTrichopoda_ERN14235.1
0.2
