# Supplementary material for: Altered Mitochondrial Respiration Is Associated With Loss of Nuclear‐Encoded OXPHOS Genes in Parasitic Broomrapes
Source: Ecol Evol. 2025 Jul 6;15(7):e71737. doi: 10.1002/ece3.71737 (PMC12230201; doi:10.1002/ece3.71737)
Supplement: Supplementary file 1 — Appendix S1. [file ECE3-15-e71737-s003.tgz › Orobanchaceae_O2K_Supplementary_v3/Data_S1/DataS1/AOX/OG0002538.filter.fas.treefile.pdf]

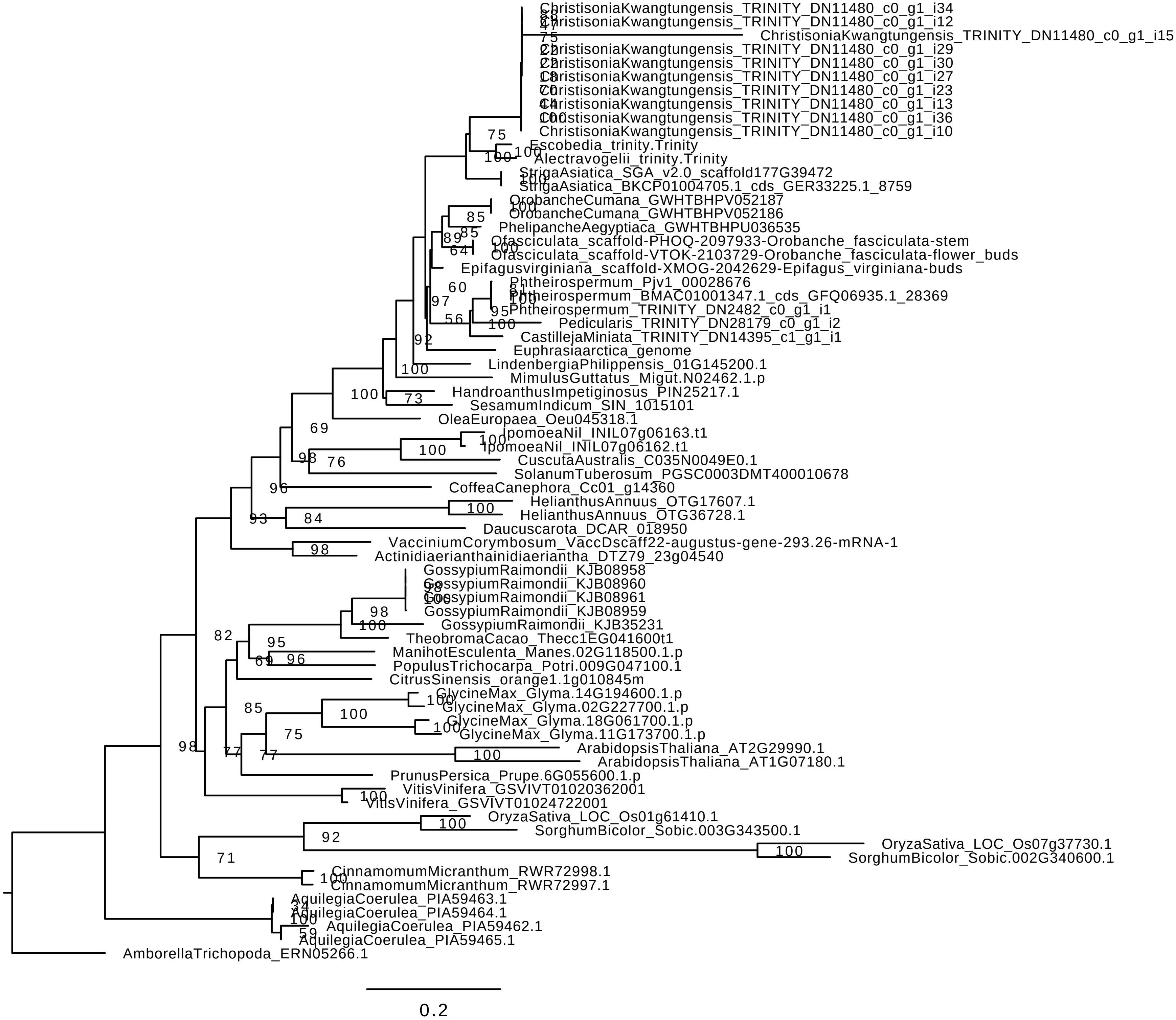
ChristisoniaKwangtungensis_TRINITY_DN11480_c0_g1_i34
ChristisoniaKwangtungensis_TRINITY_DN11480_c0_g1_i12
ChristisoniaKwangtungensis_TRINITY_DN11480_c0_g1_i15
ChristisoniaKwangtungensis_TRINITY_DN11480_c0_g1_i29
ChristisoniaKwangtungensis_TRINITY_DN11480_c0_g1_i30
ChristisoniaKwangtungensis_TRINITY_DN11480_c0_g1_i27
ChristisoniaKwangtungensis_TRINITY_DN11480_c0_g1_i23
ChristisoniaKwangtungensis_TRINITY_DN11480_c0_g1_i13
ChristisoniaKwangtungensis_TRINITY_DN11480_c0_g1_i36
ChristisoniaKwangtungensis_TRINITY_DN11480_c0_g1_i10
Escobedia_trinity.Trinity
Alectravogelii_trinity.Trinity
StrigaAsiatica_SGA_v2.0_scaffold177G39472
StrigaAsiatica_BKCP01004705.1_cds_GER33225.1_8759
OrobancheCumana_GWHTBHPV052187
OrobancheCumana_GWHTBHPV052186
PhelipancheAegyptiaca_GWHTBHPU036535
Ofasciculata_scaffold-PHOQ-2097933-Orobanche_fasciculata-stem
Ofasciculata_scaffold-VTOK-2103729-Orobanche_fasciculata-flower_buds
Epifagusvirginiana_scaffold-XMOG-2042629-Epifagus_virginiana-buds
Phtheirospermum_Pjv1_00028676
Phtheirospermum_BMAC01001347.1_cds_GFQ06935.1_28369
Phtheirospermum_TRINITY_DN2482_c0_g1_i1
Pedicularis_TRINITY_DN28179_c0_g1_i2
CastillejaMiniata_TRINITY_DN14395_c1_g1_i1
Euphrasiaarctica_genome
LindenbergiaPhilippensis_01G145200.1
MimulusGuttatus_Migut.N02462.1.p
HandroanthusImpetiginosus_PIN25217.1
SesamumIndicum_SIN_1015101
OleaEuropaea_Oeu045318.1
IpomoeaNil_INIL07g06163.t1
IpomoeaNil_INIL07g06162.t1
CuscutaAustralis_C035N0049E0.1
SolanumTuberosum_PGSC0003DMT400010678
CoffeaCanephora_Cc01_g14360
HelianthusAnnuus_OTG17607.1
HelianthusAnnuus_OTG36728.1
Daucuscarota_DCAR_018950
VacciniumCorymbosum_VaccDscaff22-augustus-gene-293.26-mRNA-1
Actinidiaerianthainidiaeriantha_DTZ79_23g04540
GossypiumRaimondii_KJB08958
GossypiumRaimondii_KJB08960
GossypiumRaimondii_KJB08961
GossypiumRaimondii_KJB08959
GossypiumRaimondii_KJB35231
TheobromaCacao_Thecc1EG041600t1
ManihotEsculenta_Manes.02G118500.1.p
PopulusTrichocarpa_Potri.009G047100.1
CitrusSinensis_orange1.1g010845m
GlycineMax_Glyma.14G194600.1.p
GlycineMax_Glyma.02G227700.1.p
GlycineMax_Glyma.18G061700.1.p
GlycineMax_Glyma.11G173700.1.p
ArabidopsisThaliana_AT2G29990.1
ArabidopsisThaliana_AT1G07180.1
PrunusPersica_Prupe.6G055600.1.p
VitisVinifera_GSVIVT01020362001
VitisVinifera_GSVIVT01024722001
OryzaSativa_LOC_Os01g61410.1
SorghumBicolor_Sobic.003G343500.1
OryzaSativa_LOC_Os07g37730.1
SorghumBicolor_Sobic.002G340600.1
CinnamomumMicranthum_RWR72998.1
CinnamomumMicranthum_RWR72997.1
AquilegiaCoerulea_PIA59463.1
AquilegiaCoerulea_PIA59464.1
AquilegiaCoerulea_PIA59462.1
AquilegiaCoerulea_PIA59465.1
AmborellaTrichopoda_ERN05266.1
0.2
