# Supplementary material for: Altered Mitochondrial Respiration Is Associated With Loss of Nuclear‐Encoded OXPHOS Genes in Parasitic Broomrapes
Source: Ecol Evol. 2025 Jul 6;15(7):e71737. doi: 10.1002/ece3.71737 (PMC12230201; doi:10.1002/ece3.71737)
Supplement: Supplementary file 1 — Appendix S1. [file ECE3-15-e71737-s003.tgz › Orobanchaceae_O2K_Supplementary_v3/Data_S1/DataS1/AOX/OG0001609.filter.fas.treefile.pdf]

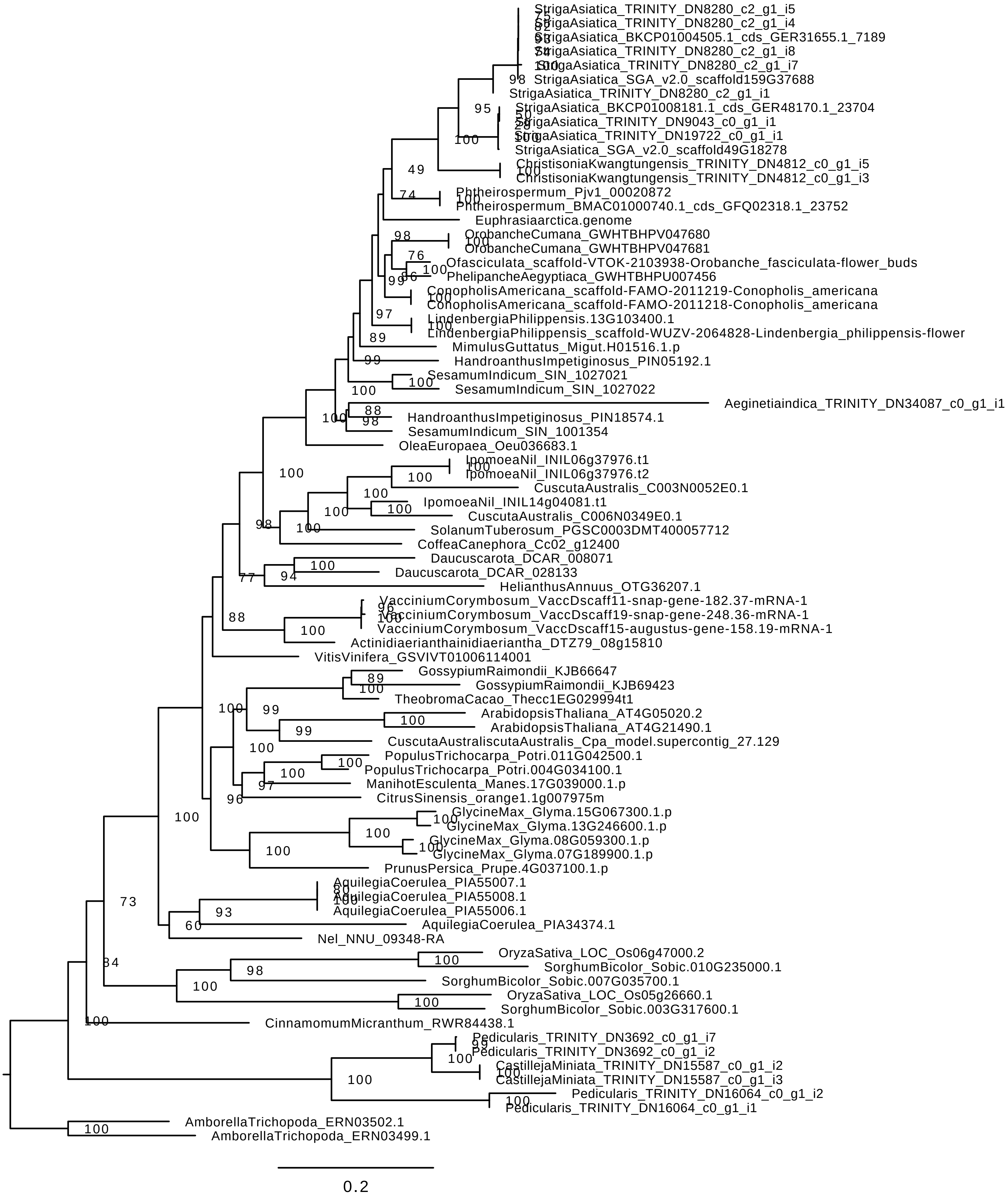
StrigaAsiatica_TRINITY_DN8280_c2_g1_i5
StrigaAsiatica_TRINITY_DN8280_c2_g1_i4
StrigaAsiatica_BKCP01004505.1_cds_GER31655.1_7189
StrigaAsiatica_TRINITY_DN8280_c2_g1_i8
StrigaAsiatica_TRINITY_DN8280_c2_g1_i7
StrigaAsiatica_SGA_v2.0_scaffold159G37688
StrigaAsiatica_TRINITY_DN8280_c2_g1_i1
StrigaAsiatica_BKCP01008181.1_cds_GER48170.1_23704
StrigaAsiatica_TRINITY_DN9043_c0_g1_i1
StrigaAsiatica_TRINITY_DN19722_c0_g1_i1
StrigaAsiatica_SGA_v2.0_scaffold49G18278
ChristisoniaKwangtungensis_TRINITY_DN4812_c0_g1_i5
ChristisoniaKwangtungensis_TRINITY_DN4812_c0_g1_i3
Phtheirospermum_Pjv1_00020872
Phtheirospermum_BMAC01000740.1_cds_GFQ02318.1_23752
Euphrasiaarctica.genome
OrobancheCumana_GWHTBHPV047680
OrobancheCumana_GWHTBHPV047681
Ofasciculata_scaffold-VTOK-2103938-Orobanche_fasciculata-flower_buds
PhelipancheAegyptiaca_GWHTBHPU007456
ConopholisAmericana_scaffold-FAMO-2011219-Conopholis_americana
ConopholisAmericana_scaffold-FAMO-2011218-Conopholis_americana
LindenbergiaPhilippensis.13G103400.1
LindenbergiaPhilippensis_scaffold-WUZV-2064828-Lindenbergia_philippensis-flower
MimulusGuttatus_Migut.H01516.1.p
HandroanthusImpetiginosus_PIN05192.1
SesamumIndicum_SIN_1027021
SesamumIndicum_SIN_1027022
Aeginetiaindica_TRINITY_DN34087_c0_g1_i1
HandroanthusImpetiginosus_PIN18574.1
SesamumIndicum_SIN_1001354
OleaEuropaea_Oeu036683.1
IpomoeaNil_INIL06g37976.t1
IpomoeaNil_INIL06g37976.t2
CuscutaAustralis_C003N0052E0.1
IpomoeaNil_INIL14g04081.t1
CuscutaAustralis_C006N0349E0.1
SolanumTuberosum_PGSC0003DMT400057712
CoffeaCanephora_Cc02_g12400
Daucuscarota_DCAR_008071
Daucuscarota_DCAR_028133
HelianthusAnnuus_OTG36207.1
VacciniumCorymbosum_VaccDscaff11-snap-gene-182.37-mRNA-1
VacciniumCorymbosum_VaccDscaff19-snap-gene-248.36-mRNA-1
VacciniumCorymbosum_VaccDscaff15-augustus-gene-158.19-mRNA-1
Actinidiaerianthainidiaeriantha_DTZ79_08g15810
VitisVinifera_GSVIVT01006114001
GossypiumRaimondii_KJB66647
GossypiumRaimondii_KJB69423
TheobromaCacao_Thecc1EG029994t1
ArabidopsisThaliana_AT4G05020.2
ArabidopsisThaliana_AT4G21490.1
CuscutaAustraliscutaAustralis_Cpa_model.supercontig_27.129
PopulusTrichocarpa_Potri.011G042500.1
PopulusTrichocarpa_Potri.004G034100.1
ManihotEsculenta_Manes.17G039000.1.p
CitrusSinensis_orange1.1g007975m
GlycineMax_Glyma.15G067300.1.p
GlycineMax_Glyma.13G246600.1.p
GlycineMax_Glyma.08G059300.1.p
GlycineMax_Glyma.07G189900.1.p
PrunusPersica_Prupe.4G037100.1.p
AquilegiaCoerulea_PIA55007.1
AquilegiaCoerulea_PIA55008.1
AquilegiaCoerulea_PIA55006.1
AquilegiaCoerulea_PIA34374.1
Nel_NNU_09348-RA
OryzaSativa_LOC_Os06g47000.2
SorghumBicolor_Sobic.010G235000.1
SorghumBicolor_Sobic.007G035700.1
OryzaSativa_LOC_Os05g26660.1
SorghumBicolor_Sobic.003G317600.1
CinnamomumMicranthum_RWR84438.1
Pedicularis_TRINITY_DN3692_c0_g1_i7
Pedicularis_TRINITY_DN3692_c0_g1_i2
CastillejaMiniata_TRINITY_DN15587_c0_g1_i2
CastillejaMiniata_TRINITY_DN15587_c0_g1_i3
Pedicularis_TRINITY_DN16064_c0_g1_i2
Pedicularis_TRINITY_DN16064_c0_g1_i1
AmborellaTrichopoda_ERN03502.1
AmborellaTrichopoda_ERN03499.1
0.2
