# Supplementary material for: Altered Mitochondrial Respiration Is Associated With Loss of Nuclear‐Encoded OXPHOS Genes in Parasitic Broomrapes
Source: Ecol Evol. 2025 Jul 6;15(7):e71737. doi: 10.1002/ece3.71737 (PMC12230201; doi:10.1002/ece3.71737)
Supplement: Supplementary file 1 — Appendix S1. [file ECE3-15-e71737-s003.tgz › Orobanchaceae_O2K_Supplementary_v3/Data_S1/DataS1/AOX/OG0001616_AOX2.filter.fas.treefile.pdf]

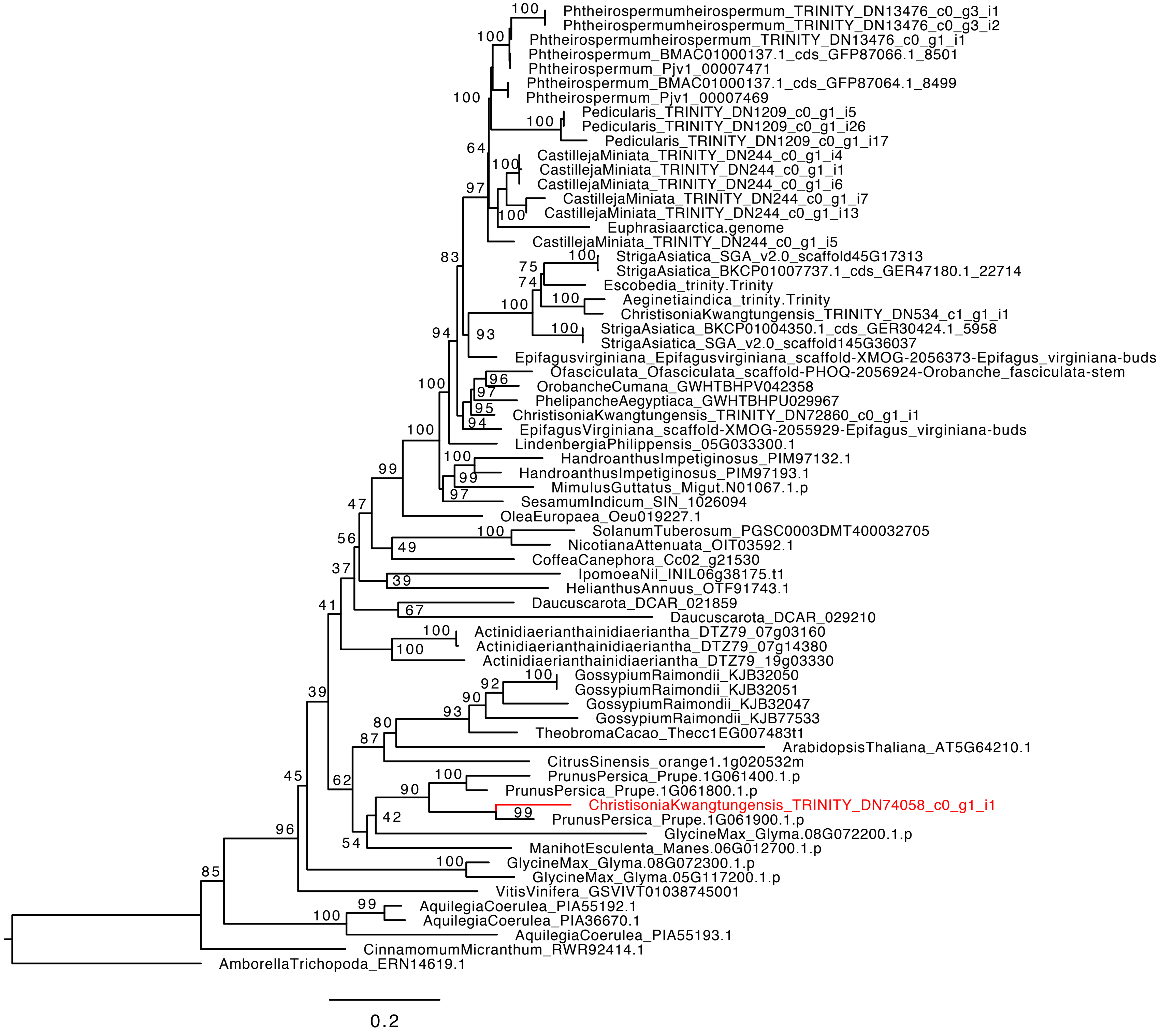
Phtheirospermumheirospermum_TRINITY_DN13476_c0_g3_i1
Phtheirospermumheirospermum_TRINITY_DN13476_c0_g3_i2
Phtheirospermumheirospermum_TRINITY_DN13476_c0_g1_i1
Phtheirospermum_BMAC01000137.1_cds_GFP87066.1_8501
Phtheirospermum_Pjv1_00007471
Phtheirospermum_BMAC01000137.1_cds_GFP87064.1_8499
Phtheirospermum_Pjv1_00007469
Pedicularis_TRINITY_DN1209_c0_g1_i5
Pedicularis_TRINITY_DN1209_c0_g1_i26
Pedicularis_TRINITY_DN1209_c0_g1_i17
CastillejaMiniata_TRINITY_DN244_c0_g1_i4
CastillejaMiniata_TRINITY_DN244_c0_g1_i1
CastillejaMiniata_TRINITY_DN244_c0_g1_i6
CastillejaMiniata_TRINITY_DN244_c0_g1_i7
CastillejaMiniata_TRINITY_DN244_c0_g1_i13
Euphrasiaarctica.genome
CastillejaMiniata_TRINITY_DN244_c0_g1_i5
StrigaAsiatica_SGA_v2.0_scaffold45G17313
StrigaAsiatica_BKCP01007737.1_cds_GER47180.1_22714
Escobedia_trinity.Trinity
Aeginetiaindica_trinity.Trinity
ChristisoniaKwangtungensis_TRINITY_DN534_c1_g1_i1
StrigaAsiatica_BKCP01004350.1_cds_GER30424.1_5958
StrigaAsiatica_SGA_v2.0_scaffold145G36037
Epifagusvirginiana_Epifagusvirginiana_scaffold-XMOG-2056373-Epifagus_virginiana-buds
Ofasciculata_Ofasciculata_scaffold-PHOQ-2056924-Orobanche_fasciculata-stem
OrobancheCumana_GWHTBHPV042358
PhelipancheAegyptiaca_GWHTBHPU029967
ChristisoniaKwangtungensis_TRINITY_DN72860_c0_g1_i1
EpifagusVirginiana_scaffold-XMOG-2055929-Epifagus_virginiana-buds
LindenbergiaPhilippensis_05G033300.1
HandroanthusImpetiginosus_PIM97132.1
HandroanthusImpetiginosus_PIM97193.1
MimulusGuttatus_Migut.N01067.1.p
SesamumIndicum_SIN_1026094
OleaEuropaea_Oeu019227.1
SolanumTuberosum_PGSC0003DMT400032705
NicotianaAttenuata_OIT03592.1
CoffeaCanephora_Cc02_g21530
IpomoeaNil_INIL06g38175.t1
HelianthusAnnuus_OTF91743.1
Daucuscarota_DCAR_021859
Daucuscarota_DCAR_029210
Actinidiaerianthainidiaeriantha_DTZ79_07g03160
Actinidiaerianthainidiaeriantha_DTZ79_07g14380
Actinidiaerianthainidiaeriantha_DTZ79_19g03330
GossypiumRaimondii_KJB32050
GossypiumRaimondii_KJB32051
GossypiumRaimondii_KJB32047
GossypiumRaimondii_KJB77533
TheobromaCacao_Thecc1EG007483t1
ArabidopsisThaliana_AT5G64210.1
CitrusSinensis_orange1.1g020532m
PrunusPersica_Prupe.1G061400.1.p
PrunusPersica_Prupe.1G061800.1.p
ChristisoniaKwangtungensis_TRINITY_DN74058_c0_g1_i1
PrunusPersica_Prupe.1G061900.1.p
GlycineMax_Glyma.08G072200.1.p
ManihotEsculenta_Manes.06G012700.1.p
GlycineMax_Glyma.08G072300.1.p
GlycineMax_Glyma.05G117200.1.p
VitisVinifera_GSVIVT01038745001
AquilegiaCoerulea_PIA55192.1
AquilegiaCoerulea_PIA36670.1
AquilegiaCoerulea_PIA55193.1
CinnamomumMicranthum_RWR92414.1
AmborellaTrichopoda_ERN14619.1
0.2
