# Supplementary material for: Altered Mitochondrial Respiration Is Associated With Loss of Nuclear‐Encoded OXPHOS Genes in Parasitic Broomrapes
Source: Ecol Evol. 2025 Jul 6;15(7):e71737. doi: 10.1002/ece3.71737 (PMC12230201; doi:10.1002/ece3.71737)
Supplement: Supplementary file 1 — Appendix S1. [file ECE3-15-e71737-s003.tgz › Orobanchaceae_O2K_Supplementary_v3/Data_S1/DataS1/AOX/OG0001616_AOX1.filter.fas.treefile.pdf]

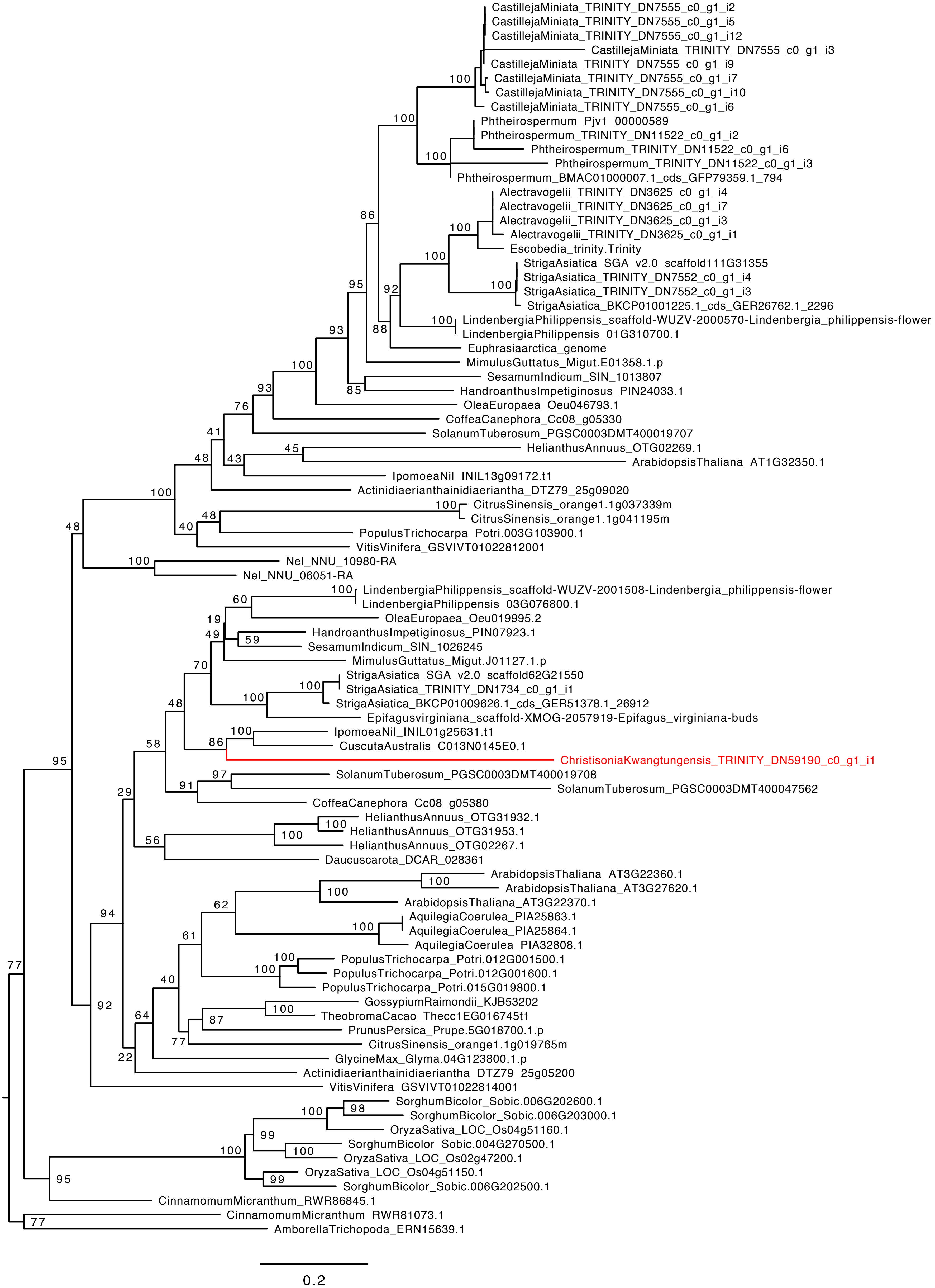
CastillejaMiniata_TRINITY_DN7555_c0_g1_i2
CastillejaMiniata_TRINITY_DN7555_c0_g1_i5
CastillejaMiniata_TRINITY_DN7555_c0_g1_i12
CastillejaMiniata_TRINITY_DN7555_c0_g1_i3
CastillejaMiniata_TRINITY_DN7555_c0_g1_i9
CastillejaMiniata_TRINITY_DN7555_c0_g1_i7
CastillejaMiniata_TRINITY_DN7555_c0_g1_i10
CastillejaMiniata_TRINITY_DN7555_c0_g1_i6
Phtheirospermum_Pjv1_00000589
Phtheirospermum_TRINITY_DN11522_c0_g1_i2
Phtheirospermum_TRINITY_DN11522_c0_g1_i6
Phtheirospermum_TRINITY_DN11522_c0_g1_i3
Phtheirospermum_BMAC01000007.1_cds_GFP79359.1_794
Alectravogelii_TRINITY_DN3625_c0_g1_i4
Alectravogelii_TRINITY_DN3625_c0_g1_i7
Alectravogelii_TRINITY_DN3625_c0_g1_i3
Alectravogelii_TRINITY_DN3625_c0_g1_i1
Escobedia_trinity.Trinity
StrigaAsiatica_SGA_v2.0_scaffold111G31355
StrigaAsiatica_TRINITY_DN7552_c0_g1_i4
StrigaAsiatica_TRINITY_DN7552_c0_g1_i3
StrigaAsiatica_BKCP01001225.1_cds_GER26762.1_2296
LindenbergiaPhilippensis_scaffold-WUZV-2000570-Lindenbergia_philippensis-flower
LindenbergiaPhilippensis_01G310700.1
Euphrasiaarctica_genome
MimulusGuttatus_Migut.E01358.1.p
SesamumIndicum_SIN_1013807
HandroanthusImpetiginosus_PIN24033.1
OleaEuropaea_Oeu046793.1
CoffeaCanephora_Cc08_g05330
SolanumTuberosum_PGSC0003DMT400019707
HelianthusAnnuus_OTG02269.1
ArabidopsisThaliana_AT1G32350.1
IpomoeaNil_INIL13g09172.t1
Actinidiaerianthainidiaeriantha_DTZ79_25g09020
CitrusSinensis_orange1.1g037339m
CitrusSinensis_orange1.1g041195m
PopulusTrichocarpa_Potri.003G103900.1
VitisVinifera_GSVIVT01022812001
Nel_NNU_10980-RA
Nel_NNU_06051-RA
LindenbergiaPhilippensis_scaffold-WUZV-2001508-Lindenbergia_philippensis-flower
LindenbergiaPhilippensis_03G076800.1
OleaEuropaea_Oeu019995.2
HandroanthusImpetiginosus_PIN07923.1
SesamumIndicum_SIN_1026245
MimulusGuttatus_Migut.J01127.1.p
StrigaAsiatica_SGA_v2.0_scaffold62G21550
StrigaAsiatica_TRINITY_DN1734_c0_g1_i1
StrigaAsiatica_BKCP01009626.1_cds_GER51378.1_26912
Epifagusvirginiana_scaffold-XMOG-2057919-Epifagus_virginiana-buds
IpomoeaNil_INIL01g25631.t1
CuscutaAustralis_C013N0145E0.1
ChristisoniaKwangtungensis_TRINITY_DN59190_c0_g1_i1
SolanumTuberosum_PGSC0003DMT400019708
SolanumTuberosum_PGSC0003DMT400047562
CoffeaCanephora_Cc08_g05380
HelianthusAnnuus_OTG31932.1
HelianthusAnnuus_OTG31953.1
HelianthusAnnuus_OTG02267.1
Daucuscarota_DCAR_028361
ArabidopsisThaliana_AT3G22360.1
ArabidopsisThaliana_AT3G27620.1
ArabidopsisThaliana_AT3G22370.1
AquilegiaCoerulea_PIA25863.1
AquilegiaCoerulea_PIA25864.1
AquilegiaCoerulea_PIA32808.1
PopulusTrichocarpa_Potri.012G001500.1
PopulusTrichocarpa_Potri.012G001600.1
PopulusTrichocarpa_Potri.015G019800.1
GossypiumRaimondii_KJB53202
TheobromaCacao_Thecc1EG016745t1
PrunusPersica_Prupe.5G018700.1.p
CitrusSinensis_orange1.1g019765m
GlycineMax_Glyma.04G123800.1.p
Actinidiaerianthainidiaeriantha_DTZ79_25g05200
VitisVinifera_GSVIVT01022814001
SorghumBicolor_Sobic.006G202600.1
SorghumBicolor_Sobic.006G203000.1
OryzaSativa_LOC_Os04g51160.1
SorghumBicolor_Sobic.004G270500.1
OryzaSativa_LOC_Os02g47200.1
OryzaSativa_LOC_Os04g51150.1
SorghumBicolor_Sobic.006G202500.1
CinnamomumMicranthum_RWR86845.1
CinnamomumMicranthum_RWR81073.1
AmborellaTrichopoda_ERN15639.1
0.2
