# Supplementary material for: Altered Mitochondrial Respiration Is Associated With Loss of Nuclear‐Encoded OXPHOS Genes in Parasitic Broomrapes
Source: Ecol Evol. 2025 Jul 6;15(7):e71737. doi: 10.1002/ece3.71737 (PMC12230201; doi:10.1002/ece3.71737)
Supplement: Supplementary file 1 — Appendix S1. [file ECE3-15-e71737-s003.tgz › Orobanchaceae_O2K_Supplementary_v3/Data_S1/DataS1/ribosomal/OG0008136.filter.fas.treefile.pdf]

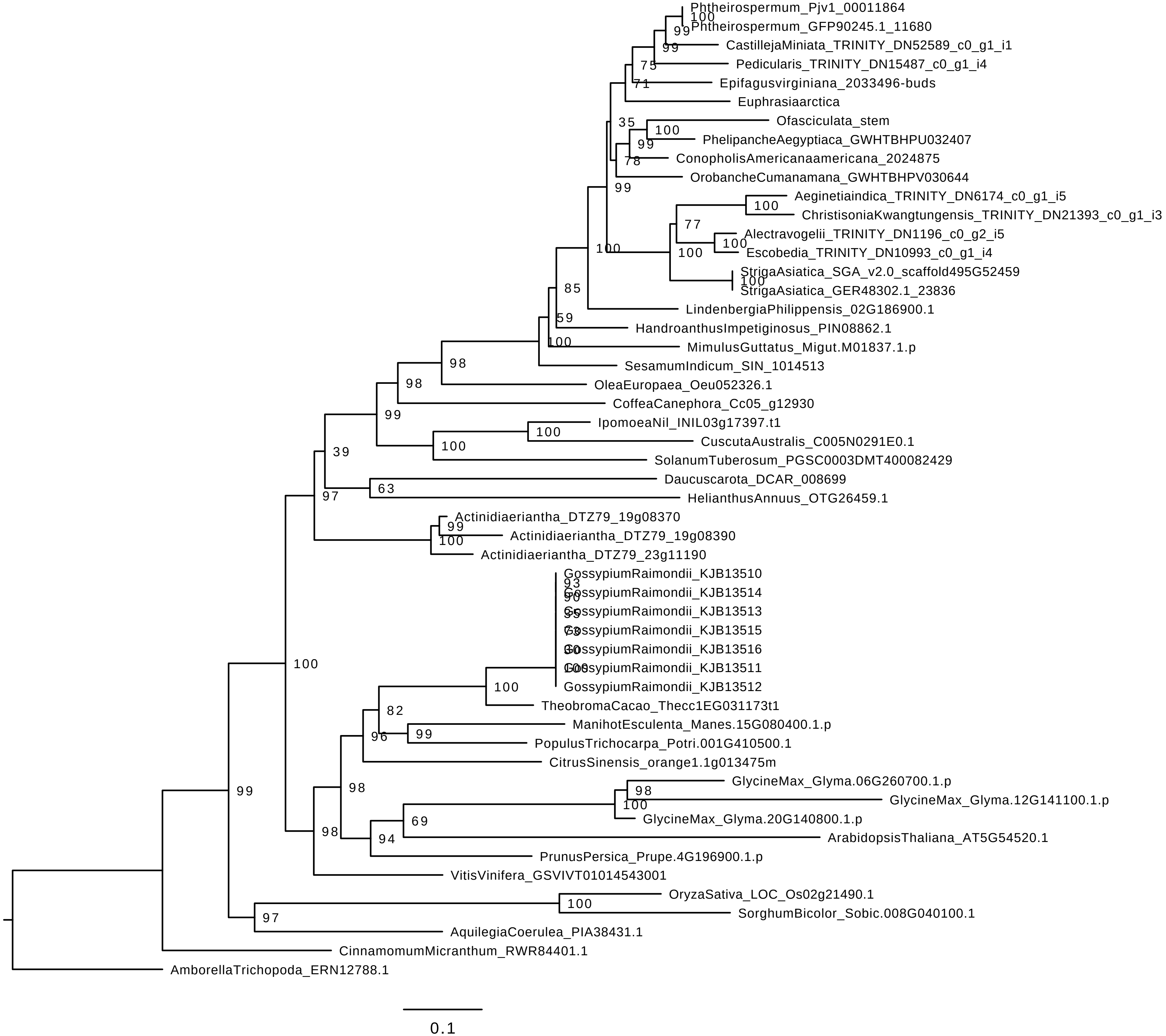
Phtheirospermum_Pjv1_00011864
100
Phtheirospermum_GFP90245.1_11680
99
CastillejaMiniata_TRINITY_DN52589_c0_g1_i1
99
Pedicularis_TRINITY_DN15487_c0_g1_i4
75
Epifagusvirginiana_2033496-buds
71
Euphrasiaarctica
35
Ofasciculata_stem
100
PhelipancheAegyptiaca_GWHTBHPU032407
99
ConopholisAmericanaamericana_2024875
78
OrobancheCumanamana_GWHTBHPV030644
99
Aeginetiaindica_TRINITY_DN6174_c0_g1_i5
100
ChristisoniaKwangtungensis_TRINITY_DN21393_c0_g1_i3
77
Alectravogelii_TRINITY_DN1196_c0_g2_i5
100
Escobedia_TRINITY_DN10993_c0_g1_i4
100
100
StrigaAsiatica_SGA_v2.0_scaffold495G52459
100
StrigaAsiatica_GER48302.1_23836
85
LindenbergiaPhilippensis_02G186900.1
59
HandroanthusImpetiginosus_PIN08862.1
100
MimulusGuttatus_Migut.M01837.1.p
SesamumIndicum_SIN_1014513
98
OleaEuropaea_Oeu052326.1
98
CoffeaCanephora_Cc05_g12930
99
IpomoeaNil_INIL03g17397.t1
100
CuscutaAustralis_C005N0291E0.1
100
SolanumTuberosum_PGSC0003DMT400082429
39
Daucuscarota_DCAR_008699
63
HelianthusAnnuus_OTG26459.1
97
Actinidiaeriantha_DTZ79_19g08370
99
Actinidiaeriantha_DTZ79_19g08390
100
Actinidiaeriantha_DTZ79_23g11190
GossypiumRaimondii_KJB13510
93
GossypiumRaimondii_KJB13514
GossypiumRaimondii_KJB13513
GossypiumRaimondii_KJB13515
GossypiumRaimondii_KJB13516
GossypiumRaimondii_KJB13511
GossypiumRaimondii_KJB13512
100
100
TheobromaCacao_Thecc1EG031173t1
82
ManihotEsculenta_Manes.15G080400.1.p
99
96
PopulusTrichocarpa_Potri.001G410500.1
CitrusSinensis_orange1.1g013475m
98
GlycineMax_Glyma.06G260700.1.p
98
GlycineMax_Glyma.12G141100.1.p
100
GlycineMax_Glyma.20G140800.1.p
69
ArabidopsisThaliana_AT5G54520.1
94
98
PrunusPersica_Prupe.4G196900.1.p
99
VitisVinifera_GSVIVT01014543001
OryzaSativa_LOC_Os02g21490.1
100
SorghumBicolor_Sobic.008G040100.1
97
AquilegiaCoerulea_PIA38431.1
CinnamomumMicranthum_RWR84401.1
AmborellaTrichopoda_ERN12788.1
0.1
