# Supplementary material for: Altered Mitochondrial Respiration Is Associated With Loss of Nuclear‐Encoded OXPHOS Genes in Parasitic Broomrapes
Source: Ecol Evol. 2025 Jul 6;15(7):e71737. doi: 10.1002/ece3.71737 (PMC12230201; doi:10.1002/ece3.71737)
Supplement: Supplementary file 1 — Appendix S1. [file ECE3-15-e71737-s003.tgz › Orobanchaceae_O2K_Supplementary_v3/Data_S1/DataS1/ribosomal/OG0007013.filter.fas.treefile.pdf]

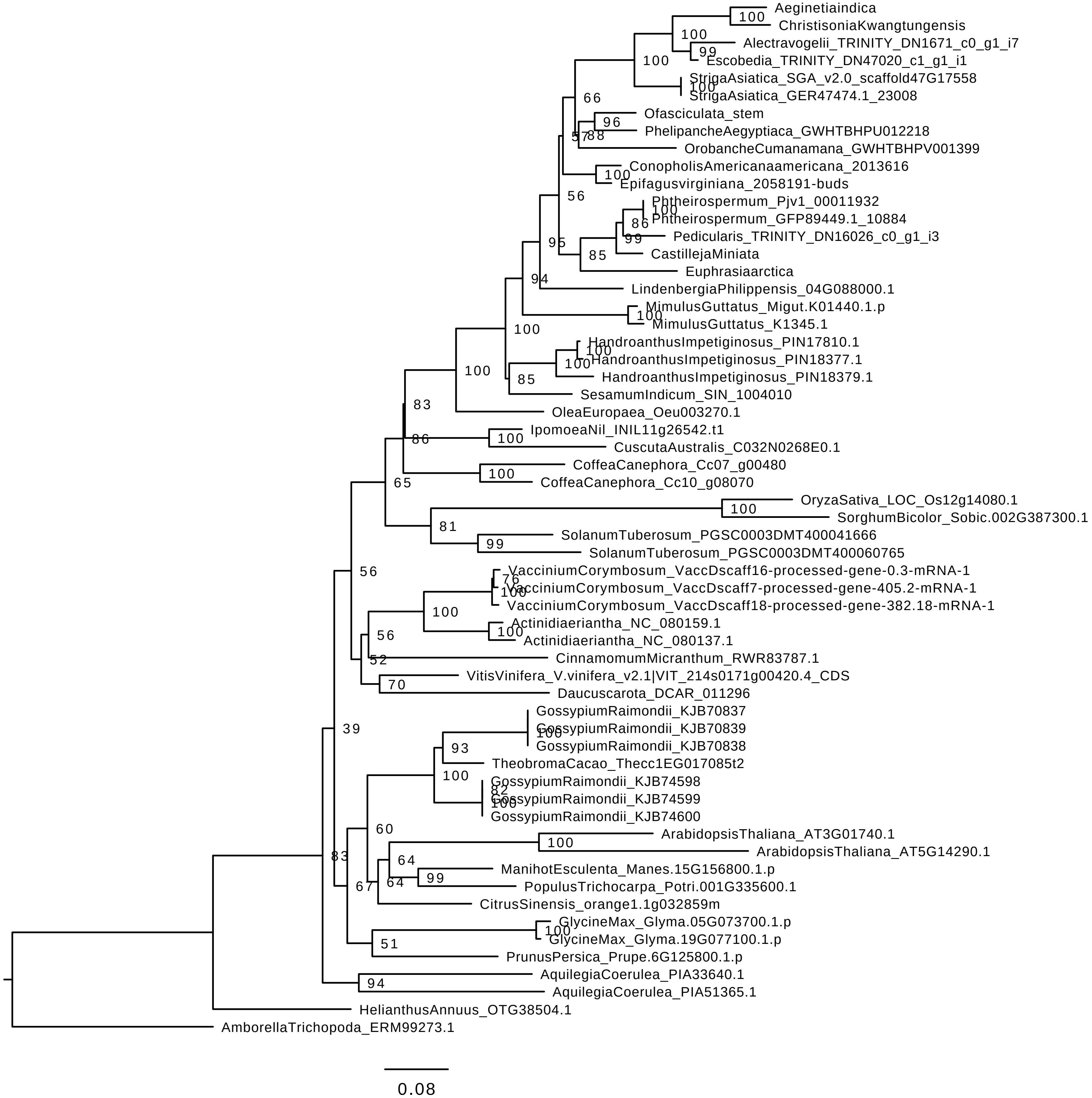
Aeginetiaindica
ChristisoniaKwangtungensis
Alectravogelii_TRINITY_DN1671_c0_g1_i7
Escobedia_TRINITY_DN47020_c1_g1_i1
StrigaAsiatica_SGA_v2.0_scaffold47G17558
StrigaAsiatica_GER47474.1_23008
Ofasciculata_stem
PhelipancheAegyptiaca_GWHTBHPU012218
OrobancheCumanamana_GWHTBHPV001399
ConopholisAmericanaamericana_2013616
Epifagusvirginiana_2058191-buds
Phtheirospermum_Pjv1_00011932
Phtheirospermum_GFP89449.1_10884
Pedicularis_TRINITY_DN16026_c0_g1_i3
CastillejaMiniata
Euphrasiaarctica
LindenbergiaPhilippensis_04G088000.1
MimulusGuttatus_Migut.K01440.1.p
MimulusGuttatus_K1345.1
HandroanthusImpetiginosus_PIN17810.1
HandroanthusImpetiginosus_PIN18377.1
HandroanthusImpetiginosus_PIN18379.1
SesamumIndicum_SIN_1004010
OleaEuropaea_Oeu003270.1
IpomoeaNil_INIL11g26542.t1
CuscutaAustralis_C032N0268E0.1
CoffeaCanephora_Cc07_g00480
CoffeaCanephora_Cc10_g08070
OryzaSativa_LOC_Os12g14080.1
SorghumBicolor_Sobic.002G387300.1
SolanumTuberosum_PGSC0003DMT400041666
SolanumTuberosum_PGSC0003DMT400060765
VacciniumCorymbosum_VaccDscaff16-processed-gene-0.3-mRNA-1
VacciniumCorymbosum_VaccDscaff7-processed-gene-405.2-mRNA-1
VacciniumCorymbosum_VaccDscaff18-processed-gene-382.18-mRNA-1
Actinidiaeriantha_NC_080159.1
Actinidiaeriantha_NC_080137.1
CinnamomumMicranthum_RWR83787.1
VitisVinifera_V.vinifera_v2.1|VIT_214s0171g00420.4_CDS
Daucuscarota_DCAR_011296
GossypiumRaimondii_KJB70837
GossypiumRaimondii_KJB70839
GossypiumRaimondii_KJB70838
TheobromaCacao_Thecc1EG017085t2
GossypiumRaimondii_KJB74598
GossypiumRaimondii_KJB74599
GossypiumRaimondii_KJB74600
ArabidopsisThaliana_AT3G01740.1
ArabidopsisThaliana_AT5G14290.1
ManihotEsculenta_Manes.15G156800.1.p
PopulusTrichocarpa_Potri.001G335600.1
CitrusSinensis_orange1.1g032859m
GlycineMax_Glyma.05G073700.1.p
GlycineMax_Glyma.19G077100.1.p
PrunusPersica_Prupe.6G125800.1.p
AquilegiaCoerulea_PIA33640.1
AquilegiaCoerulea_PIA51365.1
HelianthusAnnuus_OTG38504.1
AmborellaTrichopoda_ERM99273.1
0.08
