# Supplementary material for: Altered Mitochondrial Respiration Is Associated With Loss of Nuclear‐Encoded OXPHOS Genes in Parasitic Broomrapes
Source: Ecol Evol. 2025 Jul 6;15(7):e71737. doi: 10.1002/ece3.71737 (PMC12230201; doi:10.1002/ece3.71737)
Supplement: Supplementary file 1 — Appendix S1. [file ECE3-15-e71737-s003.tgz › Orobanchaceae_O2K_Supplementary_v3/Data_S1/DataS1/ribosomal/OG0006722.filter.fas.treefile.pdf]

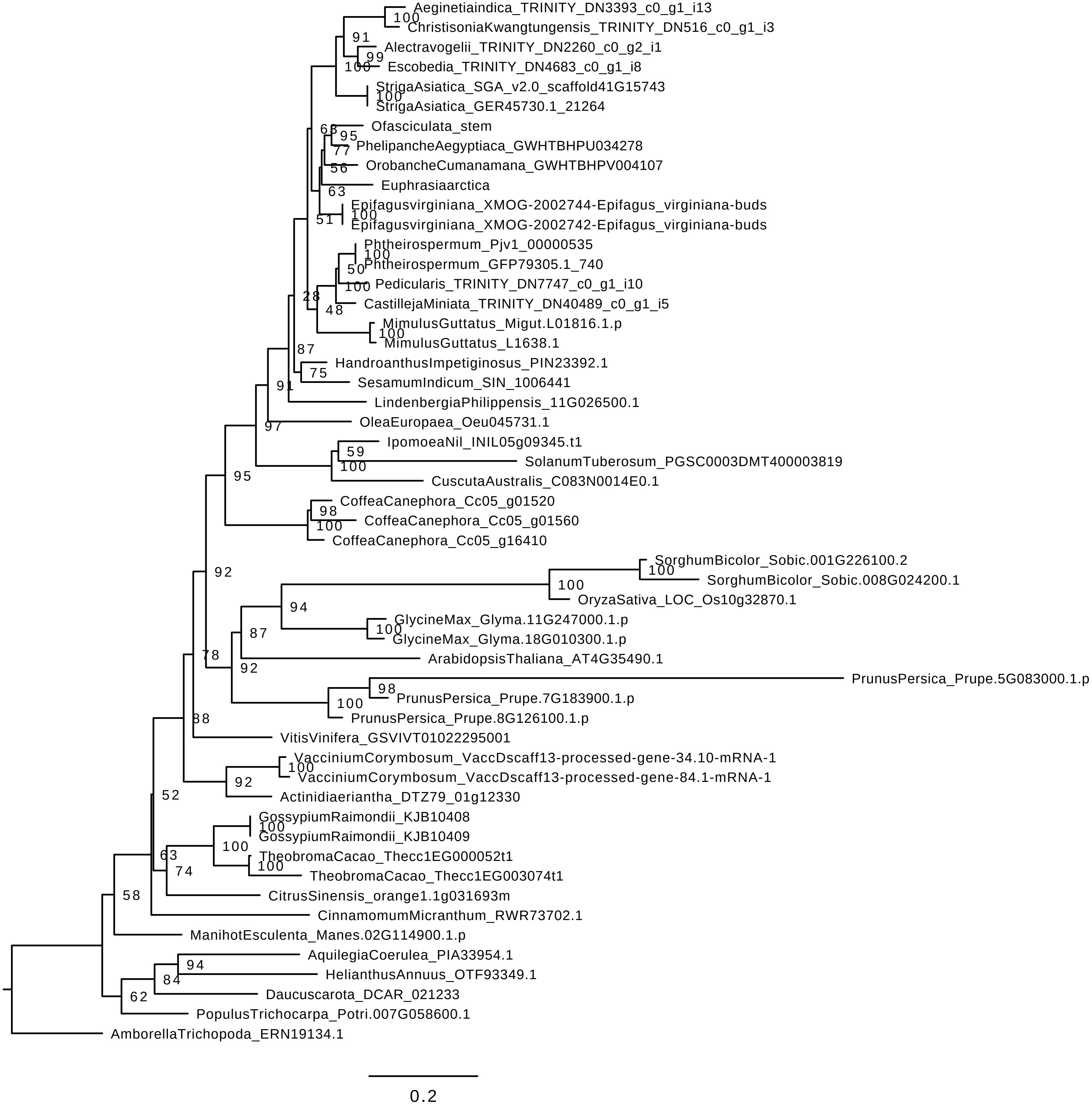
Aeginetiaindica_TRINITY_DN3393_c0_g1_i13
100
ChristisoniaKwangtungensis_TRINITY_DN516_c0_g1_i3
91
Alectravogelii_TRINITY_DN2260_c0_g2_i1
99
100
Escobedia_TRINITY_DN4683_c0_g1_i8
StrigaAsiatica_SGA_v2.0_scaffold41G15743
100
StrigaAsiatica_GER45730.1_21264
63
Ofasciculata_stem
95
77
PhelipancheAegyptiaca_GWHTBHPU034278
56
OrobancheCumanamana_GWHTBHPV004107
Euphrasiaarctica
63
Epifagusvirginiana_XMOG-2002744-Epifagus_virginiana-buds
51
100
Epifagusvirginiana_XMOG-2002742-Epifagus_virginiana-buds
Phtheirospermum_Pjv1_00000535
100
50
Phtheirospermum_GFP79305.1_740
100
Pedicularis_TRINITY_DN7747_c0_g1_i10
28
CastillejaMiniata_TRINITY_DN40489_c0_g1_i5
48
MimulusGuttatus_Migut.L01816.1.p
100
MimulusGuttatus_L1638.1
87
HandroanthusImpetiginosus_PIN23392.1
75
SesamumIndicum_SIN_1006441
91
LindenbergiaPhilippensis_11G026500.1
OleaEuropaea_Oeu045731.1
97
IpomoeaNil_INIL05g09345.t1
59
SolanumTuberosum_PGSC0003DMT400003819
100
CuscutaAustralis_C083N0014E0.1
95
CoffeaCanephora_Cc05_g01520
98
CoffeaCanephora_Cc05_g01560
100
CoffeaCanephora_Cc05_g16410
SorghumBicolor_Sobic.001G226100.2
92
100
SorghumBicolor_Sobic.008G024200.1
100
OryzaSativa_LOC_Os10g32870.1
94
GlycineMax_Glyma.11G247000.1.p
100
87
GlycineMax_Glyma.18G010300.1.p
78
ArabidopsisThaliana_AT4G35490.1
92
PrunusPersica_Prupe.5G083000.1.p
98
PrunusPersica_Prupe.7G183900.1.p
100
PrunusPersica_Prupe.8G126100.1.p
88
VitisVinifera_GSVIVT01022295001
VacciniumCorymbosum_VaccDscaff13-processed-gene-34.10-mRNA-1
100
92
VacciniumCorymbosum_VaccDscaff13-processed-gene-84.1-mRNA-1
52
Actinidiaeriantha_DTZ79_01g12330
GossypiumRaimondii_KJB10408
100
GossypiumRaimondii_KJB10409
100
63
TheobromaCacao_Thecc1EG000052t1
74
100
TheobromaCacao_Thecc1EG003074t1
CitrusSinensis_orange1.1g031693m
58
CinnamomumMicranthum_RWR73702.1
ManihotEsculenta_Manes.02G114900.1.p
AquilegiaCoerulea_PIA33954.1
94
84
HelianthusAnnuus_OTF93349.1
Daucuscarota_DCAR_021233
62
PopulusTrichocarpa_Potri.007G058600.1
AmborellaTrichopoda_ERN19134.1
0.2
