# Supplementary material for: Altered Mitochondrial Respiration Is Associated With Loss of Nuclear‐Encoded OXPHOS Genes in Parasitic Broomrapes
Source: Ecol Evol. 2025 Jul 6;15(7):e71737. doi: 10.1002/ece3.71737 (PMC12230201; doi:10.1002/ece3.71737)
Supplement: Supplementary file 1 — Appendix S1. [file ECE3-15-e71737-s003.tgz › Orobanchaceae_O2K_Supplementary_v3/Data_S1/DataS1/ribosomal/OG0010884.filter.fas.treefile.pdf]

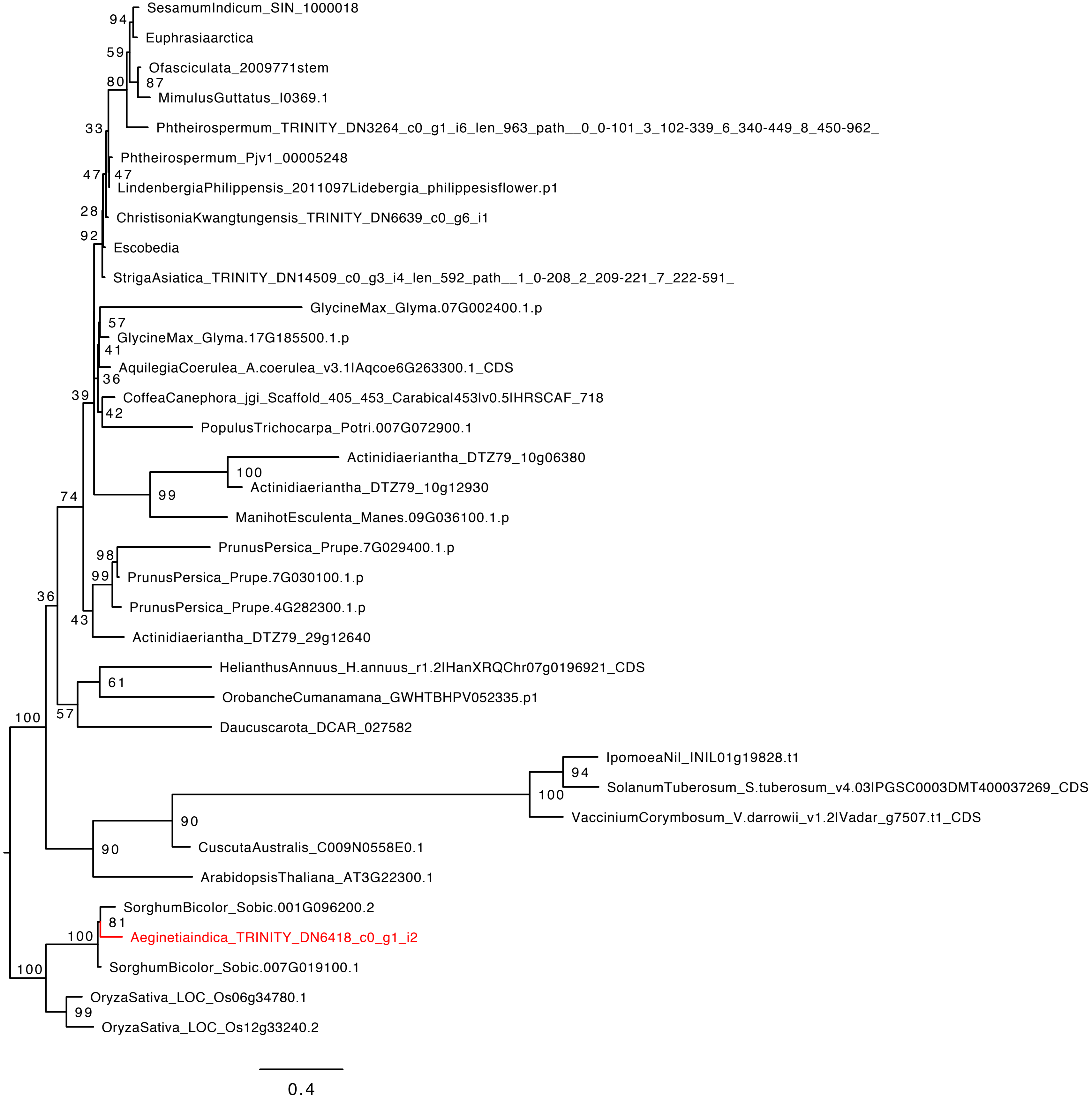

SesamumIndicum_SIN_1000018
Euphrasiaarctica
Ofasciculata_2009771stem
MimulusGuttatus_I0369.1
Phtheirospermum_TRINITY_DN3264_c0_g1_i6_len_963_path__0_0-101_3_102-339_6_340-449_8_450-962_
Phtheirospermum_Pjv1_00005248
LindenbergiaPhilippensis_2011097Lidebergia_philippesisflower.p1
ChristisoniaKwangtungensis_TRINITY_DN6639_c0_g6_i1
Escobedia
StrigaAsiatica_TRINITY_DN14509_c0_g3_i4_len_592_path__1_0-208_2_209-221_7_222-591_
GlycineMax_Glyma.07G002400.1.p
GlycineMax_Glyma.17G185500.1.p
AquilegiaCoerulea_A.coerulea_v3.1lAqcoe6G263300.1_CDS
CoffeaCanephora_jgi_Scaffold_405_453_Carabical453lv0.5lHRSCAF_718
PopulusTrichocarpa_Potri.007G072900.1
Actinidiaeriantha_DTZ79_10g06380
Actinidiaeriantha_DTZ79_10g12930
ManihotEsculenta_Manes.09G036100.1.p
PrunusPersica_Prupe.7G029400.1.p
PrunusPersica_Prupe.7G030100.1.p
PrunusPersica_Prupe.4G282300.1.p
Actinidiaeriantha_DTZ79_29g12640
HelianthusAnnuus_H.annuus_r1.2lHanXRQChr07g0196921_CDS
OrobancheCumanamana_GWHTBHPV052335.p1
Daucuscarota_DCAR_027582
IpomoeaNil_INIL01g19828.t1
SolanumTuberosum_S.tuberosum_v4.03lPGSC0003DMT400037269_CDS
VacciniumCorymbosum_V.darrowii_v1.2lVadar_g7507.t1_CDS
CuscutaAustralis_C009N0558E0.1
ArabidopsisThaliana_AT3G22300.1
SorghumBicolor_Sobic.001G096200.2
Aeginetiaindica_TRINITY_DN6418_c0_g1_i2
SorghumBicolor_Sobic.007G019100.1
OryzaSativa_LOC_Os06g34780.1
OryzaSativa_LOC_Os12g33240.2
94
59
87
80
33
47
47
28
92
57
41
36
42
39
100
99
74
98
99
43
36
61
57
100
94
100
90
90
81
100
100
99
0.4
