# Supplementary material for: Altered Mitochondrial Respiration Is Associated With Loss of Nuclear‐Encoded OXPHOS Genes in Parasitic Broomrapes
Source: Ecol Evol. 2025 Jul 6;15(7):e71737. doi: 10.1002/ece3.71737 (PMC12230201; doi:10.1002/ece3.71737)
Supplement: Supplementary file 1 — Appendix S1. [file ECE3-15-e71737-s003.tgz › Orobanchaceae_O2K_Supplementary_v3/Data_S1/DataS1/ribosomal/OG0006973.filter.fas.treefile.pdf]

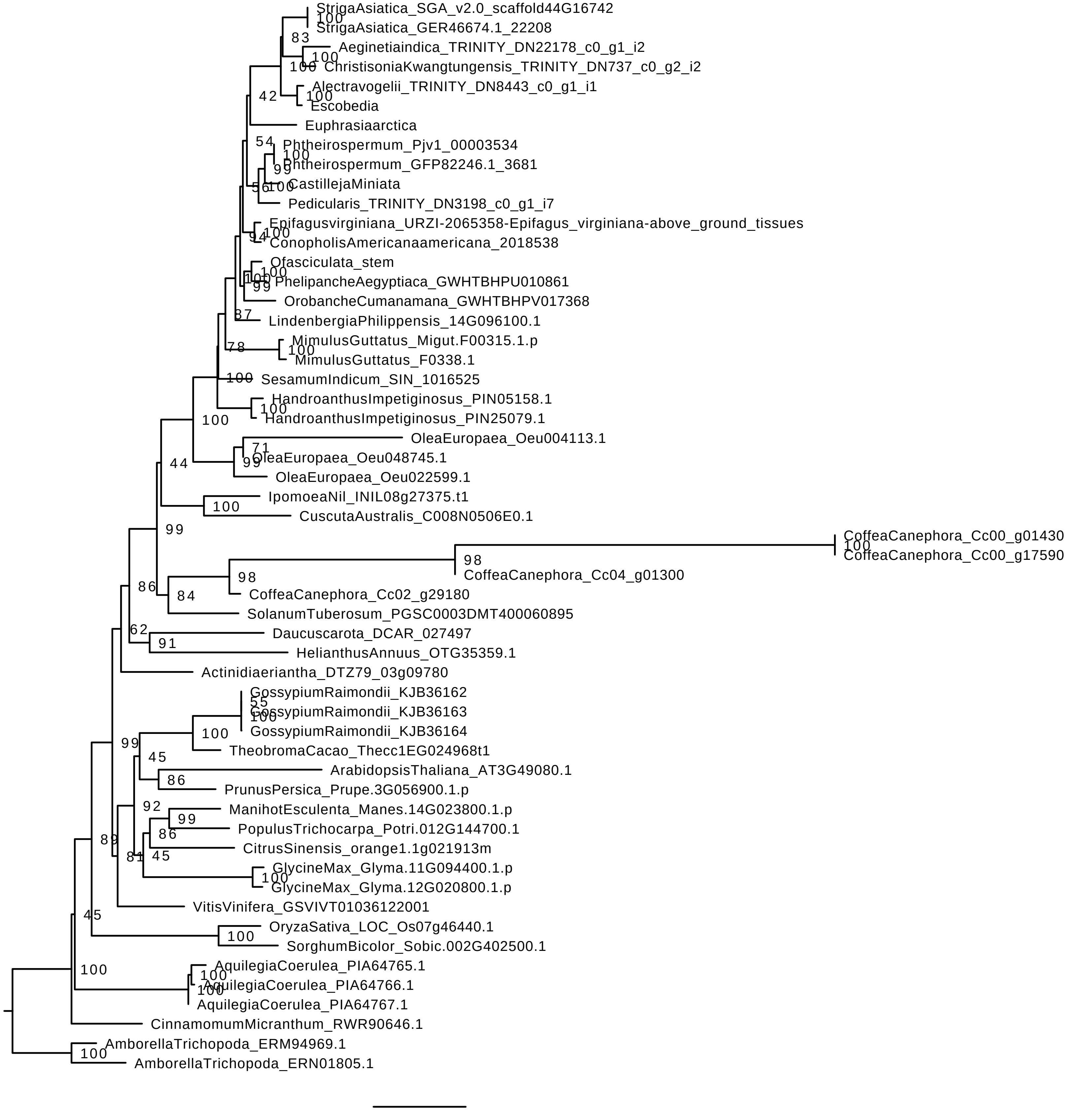
StrigaAsiatica_SGA_v2.0_scaffold44G16742
StrigaAsiatica_GER46674.1_22208
Aeginetiaindica_TRINITY_DN22178_c0_g1_i2
ChristisoniaKwangtungensis_TRINITY_DN737_c0_g2_i2
Alectravogelii_TRINITY_DN8443_c0_g1_i1
Escobedia
Euphrasiaarctica
Phtheirospermum_Pjv1_00003534
Phtheirospermum_GFP82246.1_3681
CastillejaMiniata
Pedicularis_TRINITY_DN3198_c0_g1_i7
Epifagusvirginiana_URZI-2065358-Epifagus_virginiana-above_ground_tissues
ConopholisAmericanaamericana_2018538
Ofasciculata_stem
PhelipancheAegyptiaca_GWHTBHPU010861
OrobancheCumanamana_GWHTBHPV017368
LindenbergiaPhilippensis_14G096100.1
MimulusGuttatus_Migut.F00315.1.p
MimulusGuttatus_F0338.1
SesamumIndicum_SIN_1016525
HandroanthusImpetiginosus_PIN05158.1
HandroanthusImpetiginosus_PIN25079.1
OleaEuropaea_Oeu004113.1
OleaEuropaea_Oeu048745.1
OleaEuropaea_Oeu022599.1
IpomoeaNil_INIL08g27375.t1
CuscutaAustralis_C008N0506E0.1
CoffeaCanephora_Cc00_g01430
CoffeaCanephora_Cc00_g17590
CoffeaCanephora_Cc04_g01300
CoffeaCanephora_Cc02_g29180
SolanumTuberosum_PGSC0003DMT400060895
Daucuscarota_DCAR_027497
HelianthusAnnuus_OTG35359.1
Actinidiaeriantha_DTZ79_03g09780
GossypiumRaimondii_KJB36162
GossypiumRaimondii_KJB36163
GossypiumRaimondii_KJB36164
TheobromaCacao_Thecc1EG024968t1
ArabidopsisThaliana_AT3G49080.1
PrunusPersica_Prupe.3G056900.1.p
ManihotEsculenta_Manes.14G023800.1.p
PopulusTrichocarpa_Potri.012G144700.1
CitrusSinensis_orange1.1g021913m
GlycineMax_Glyma.11G094400.1.p
GlycineMax_Glyma.12G020800.1.p
VitisVinifera_GSVIVT01036122001
OryzaSativa_LOC_Os07g46440.1
SorghumBicolor_Sobic.002G402500.1
AquilegiaCoerulea_PIA64765.1
AquilegiaCoerulea_PIA64766.1
AquilegiaCoerulea_PIA64767.1
CinnamomumMicranthum_RWR90646.1
AmborellaTrichopoda_ERM94969.1
AmborellaTrichopoda_ERN01805.1
0.3
