# Supplementary material for: Altered Mitochondrial Respiration Is Associated With Loss of Nuclear‐Encoded OXPHOS Genes in Parasitic Broomrapes
Source: Ecol Evol. 2025 Jul 6;15(7):e71737. doi: 10.1002/ece3.71737 (PMC12230201; doi:10.1002/ece3.71737)
Supplement: Supplementary file 1 — Appendix S1. [file ECE3-15-e71737-s003.tgz › Orobanchaceae_O2K_Supplementary_v3/Data_S1/DataS1/ribosomal/OG0006560.filter.fas.treefile.pdf]

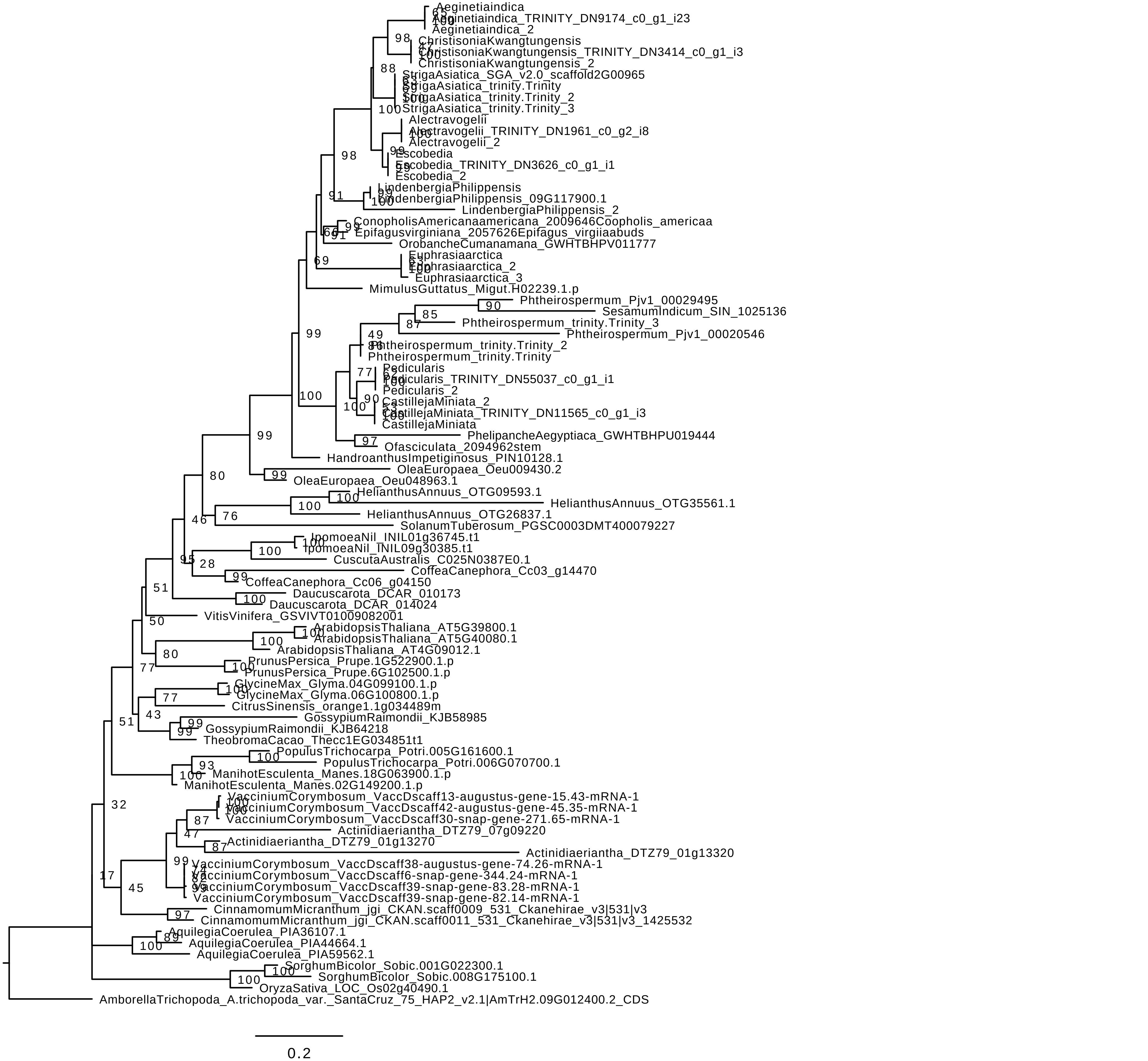
Aeginetiaindica
Aeginetiaindica_TRINITY_DN9174_c0_g1_i23
Aeginetiaindica_2
ChristisoniaKwangtungensis
ChristisoniaKwangtungensis_TRINITY_DN3414_c0_g1_i3
ChristisoniaKwangtungensis_2
StrigaAsiatica_SGA_v2.0_scaffold2G00965
StrigaAsiatica_trinity.Trinity
StrigaAsiatica_trinity.Trinity_2
StrigaAsiatica_trinity.Trinity_3
Alectravogelii
Alectravogelii_TRINITY_DN1961_c0_g2_i8
Alectravogelii_2
Escobedia
Escobedia_TRINITY_DN3626_c0_g1_i1
Escobedia_2
LindenbergiaPhilippensis
LindenbergiaPhilippensis_09G117900.1
LindenbergiaPhilippensis_2
ConopholisAmericanaamericana_2009646Coopholis_americaa
Epifagusvirginiana_2057626Epifagus_virgiiaabuds
OrobancheCumanamana_GWHTBHPV011777
Euphrasiaarctica
Euphrasiaarctica_2
Euphrasiaarctica_3
MimulusGuttatus_Migut.H02239.1.p
Phtheirospermum_Pjv1_00029495
SesamumIndicum_SIN_1025136
Phtheirospermum_trinity.Trinity_3
Phtheirospermum_Pjv1_00020546
Phtheirospermum_trinity.Trinity_2
Phtheirospermum_trinity.Trinity
Pedicularis
Pedicularis_TRINITY_DN55037_c0_g1_i1
Pedicularis_2
CastillejaMiniata_2
CastillejaMiniata_TRINITY_DN11565_c0_g1_i3
CastillejaMiniata
PhelipancheAegyptiaca_GWHTBHPU019444
Ofasciculata_2094962stem
HandroanthusImpetiginosus_PIN10128.1
OleaEuropaea_Oeu009430.2
OleaEuropaea_Oeu048963.1
HelianthusAnnuus_OTG09593.1
HelianthusAnnuus_OTG35561.1
HelianthusAnnuus_OTG26837.1
SolanumTuberosum_PGSC0003DMT400079227
IpomoeaNil_INIL01g36745.t1
IpomoeaNil_INIL09g30385.t1
CuscutaAustralis_C025N0387E0.1
CoffeaCanephora_Cc03_g14470
CoffeaCanephora_Cc06_g04150
Daucuscarota_DCAR_010173
Daucuscarota_DCAR_014024
VitisVinifera_GSVIVT01009082001
ArabidopsisThaliana_AT5G39800.1
ArabidopsisThaliana_AT5G40080.1
ArabidopsisThaliana_AT4G09012.1
PrunusPersica_Prupe.1G522900.1.p
PrunusPersica_Prupe.6G102500.1.p
GlycineMax_Glyma.04G099100.1.p
GlycineMax_Glyma.06G100800.1.p
CitrusSinensis_orange1.1g034489m
GossypiumRaimondii_KJB58985
GossypiumRaimondii_KJB64218
TheobromaCacao_Thecc1EG034851t1
PopulusTrichocarpa_Potri.005G161600.1
PopulusTrichocarpa_Potri.006G070700.1
ManihotEsculenta_Manes.18G063900.1.p
ManihotEsculenta_Manes.02G149200.1.p
VacciniumCorymbosum_VaccDscaff13-augustus-gene-15.43-mRNA-1
VacciniumCorymbosum_VaccDscaff42-augustus-gene-45.35-mRNA-1
VacciniumCorymbosum_VaccDscaff30-snap-gene-271.65-mRNA-1
Actinidiaeriantha_DTZ79_07g09220
Actinidiaeriantha_DTZ79_01g13270
Actinidiaeriantha_DTZ79_01g13320
VacciniumCorymbosum_VaccDscaff38-augustus-gene-74.26-mRNA-1
VacciniumCorymbosum_VaccDscaff6-snap-gene-344.24-mRNA-1
VacciniumCorymbosum_VaccDscaff39-snap-gene-83.28-mRNA-1
VacciniumCorymbosum_VaccDscaff39-snap-gene-82.14-mRNA-1
CinnamomumMicranthum_jgi_CKAN.scaff0009_531_Ckanehirae_v3|531|v3
CinnamomumMicranthum_jgi_CKAN.scaff0011_531_Ckanehirae_v3|531|v3_1425532
AquilegiaCoerulea_PIA36107.1
AquilegiaCoerulea_PIA44664.1
AquilegiaCoerulea_PIA59562.1
SorghumBicolor_Sobic.001G022300.1
SorghumBicolor_Sobic.008G175100.1
OryzaSativa_LOC_Os02g40490.1
AmborellaTrichopoda_A.trichopoda_var._SantaCruz_75_HAP2_v2.1|AmTrH2.09G012400.2_CDS
0.2
