# Supplementary material for: Altered Mitochondrial Respiration Is Associated With Loss of Nuclear‐Encoded OXPHOS Genes in Parasitic Broomrapes
Source: Ecol Evol. 2025 Jul 6;15(7):e71737. doi: 10.1002/ece3.71737 (PMC12230201; doi:10.1002/ece3.71737)
Supplement: Supplementary file 1 — Appendix S1. [file ECE3-15-e71737-s003.tgz › Orobanchaceae_O2K_Supplementary_v3/Data_S1/DataS1/ribosomal/OG0004571.filter.fas.treefile.pdf]

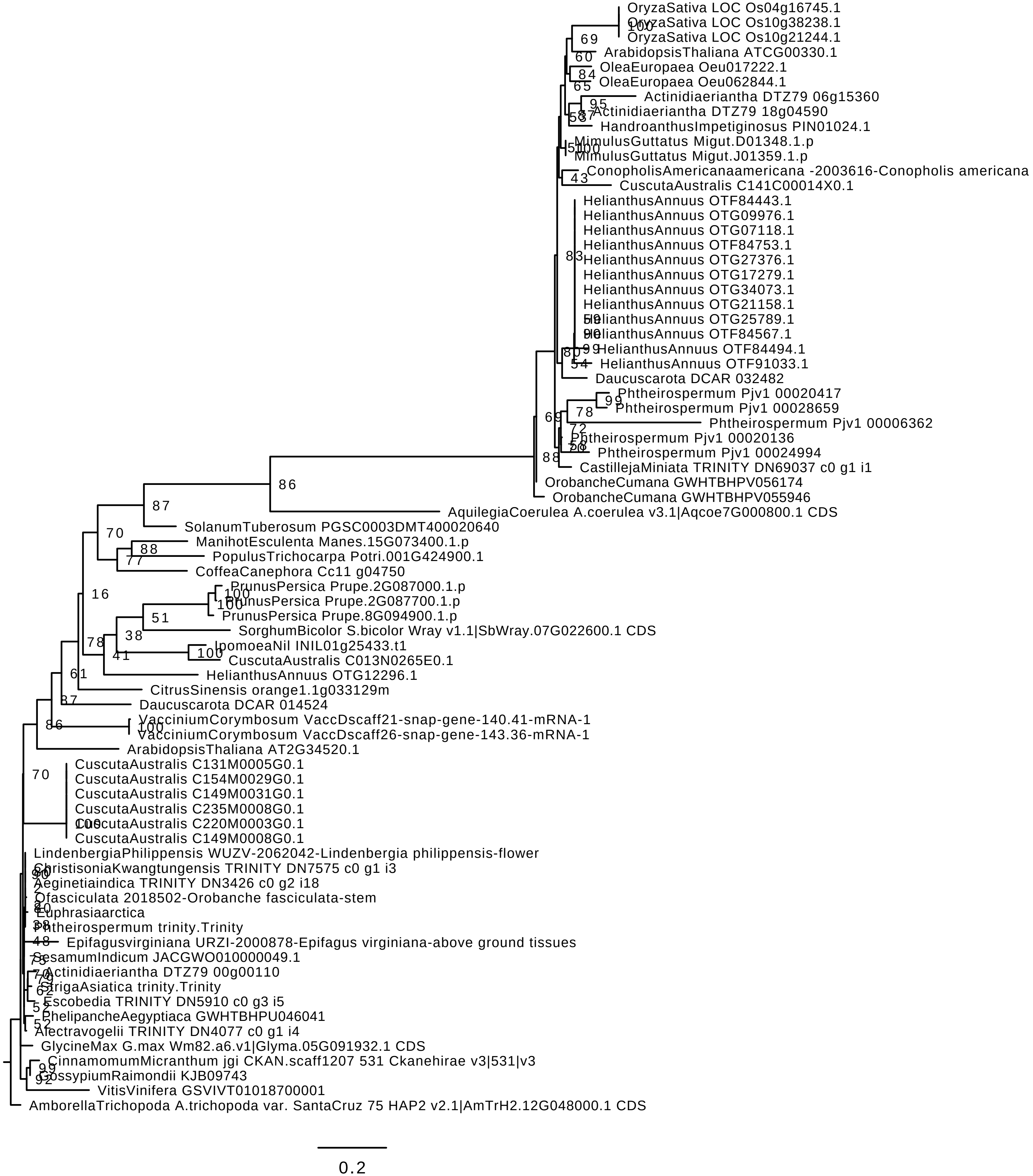
OryzaSativa LOC Os04g16745.1
OryzaSativa LOC Os10g38238.1
OryzaSativa LOC Os10g21244.1
ArabidopsisThaliana ATCG00330.1
OleaEuropaea Oeu017222.1
OleaEuropaea Oeu062844.1
Actinidiaeriantha DTZ79 06g15360
Actinidiaeriantha DTZ79 18g04590
HandroanthusImpetiginosus PIN01024.1
MimulusGuttatus Migut.D01348.1.p
MimulusGuttatus Migut.J01359.1.p
ConopholisAmericanaamericana -2003616-Conopholis americana
CuscutaAustralis C141C00014X0.1
HelianthusAnnuus OTF84443.1
HelianthusAnnuus OTG09976.1
HelianthusAnnuus OTG07118.1
HelianthusAnnuus OTF84753.1
HelianthusAnnuus OTG27376.1
HelianthusAnnuus OTG17279.1
HelianthusAnnuus OTG34073.1
HelianthusAnnuus OTG21158.1
HelianthusAnnuus OTG25789.1
HelianthusAnnuus OTF84567.1
HelianthusAnnuus OTF84494.1
HelianthusAnnuus OTF91033.1
Daucuscarota DCAR 032482
Phtheirospermum Pjv1 00020417
Phtheirospermum Pjv1 00028659
Phtheirospermum Pjv1 00006362
Phtheirospermum Pjv1 00020136
Phtheirospermum Pjv1 00024994
CastillejaMiniata TRINITY DN69037 c0 g1 i1
OrobancheCumana GWHTBHPV056174
OrobancheCumana GWHTBHPV055946
AquilegiaCoerulea A.coerulea v3.1|Aqcoe7G000800.1 CDS
SolanumTuberosum PGSC0003DMT400020640
ManihotEsculenta Manes.15G073400.1.p
PopulusTrichocarpa Potri.001G424900.1
CoffeaCanephora Cc11 g04750
PrunusPersica Prupe.2G087000.1.p
PrunusPersica Prupe.2G087700.1.p
PrunusPersica Prupe.8G094900.1.p
SorghumBicolor S.bicolor Wray v1.1|SbWray.07G022600.1 CDS
IpomoeaNil INIL01g25433.t1
CuscutaAustralis C013N0265E0.1
HelianthusAnnuus OTG12296.1
CitrusSinensis orange1.1g033129m
Daucuscarota DCAR 014524
VacciniumCorymbosum VaccDscaff21-snap-gene-140.41-mRNA-1
VacciniumCorymbosum VaccDscaff26-snap-gene-143.36-mRNA-1
ArabidopsisThaliana AT2G34520.1
CuscutaAustralis C131M0005G0.1
CuscutaAustralis C154M0029G0.1
CuscutaAustralis C149M0031G0.1
CuscutaAustralis C235M0008G0.1
CuscutaAustralis C220M0003G0.1
CuscutaAustralis C149M0008G0.1
LindenbergiaPhilippensis WUZV-2062042-Lindenbergia philippensis-flower
ChristisoniaKwangtungensis TRINITY DN7575 c0 g1 i3
Aeginetiaindica TRINITY DN3426 c0 g2 i18
Ofasciculata 2018502-Orobanche fasciculata-stem
Euphrasiaarctica
Phtheirospermum trinity.Trinity
Epifagusvirginiana URZI-2000878-Epifagus virginiana-above ground tissues
SesamumIndicum JACGWO010000049.1
Actinidiaeriantha DTZ79 00g00110
StrigaAsiatica trinity.Trinity
Escobedia TRINITY DN5910 c0 g3 i5
PhelipancheAegyptiaca GWHTBHPU046041
Alectravogelii TRINITY DN4077 c0 g1 i4
GlycineMax G.max Wm82.a6.v1|Glyma.05G091932.1 CDS
CinnamomumMicranthum jgi CKAN.scaff1207 531 Ckanehirae v3|531|v3
GossypiumRaimondii KJB09743
VitisVinifera GSVIVT01018700001
AmborellaTrichopoda A.trichopoda var. SantaCruz 75 HAP2 v2.1|AmTrH2.12G048000.1 CDS
0.2
