# Supplementary material for: Altered Mitochondrial Respiration Is Associated With Loss of Nuclear‐Encoded OXPHOS Genes in Parasitic Broomrapes
Source: Ecol Evol. 2025 Jul 6;15(7):e71737. doi: 10.1002/ece3.71737 (PMC12230201; doi:10.1002/ece3.71737)
Supplement: Supplementary file 1 — Appendix S1. [file ECE3-15-e71737-s003.tgz › Orobanchaceae_O2K_Supplementary_v3/Data_S1/DataS1/CI/OG0008027.filter.fas.treefile.pdf]

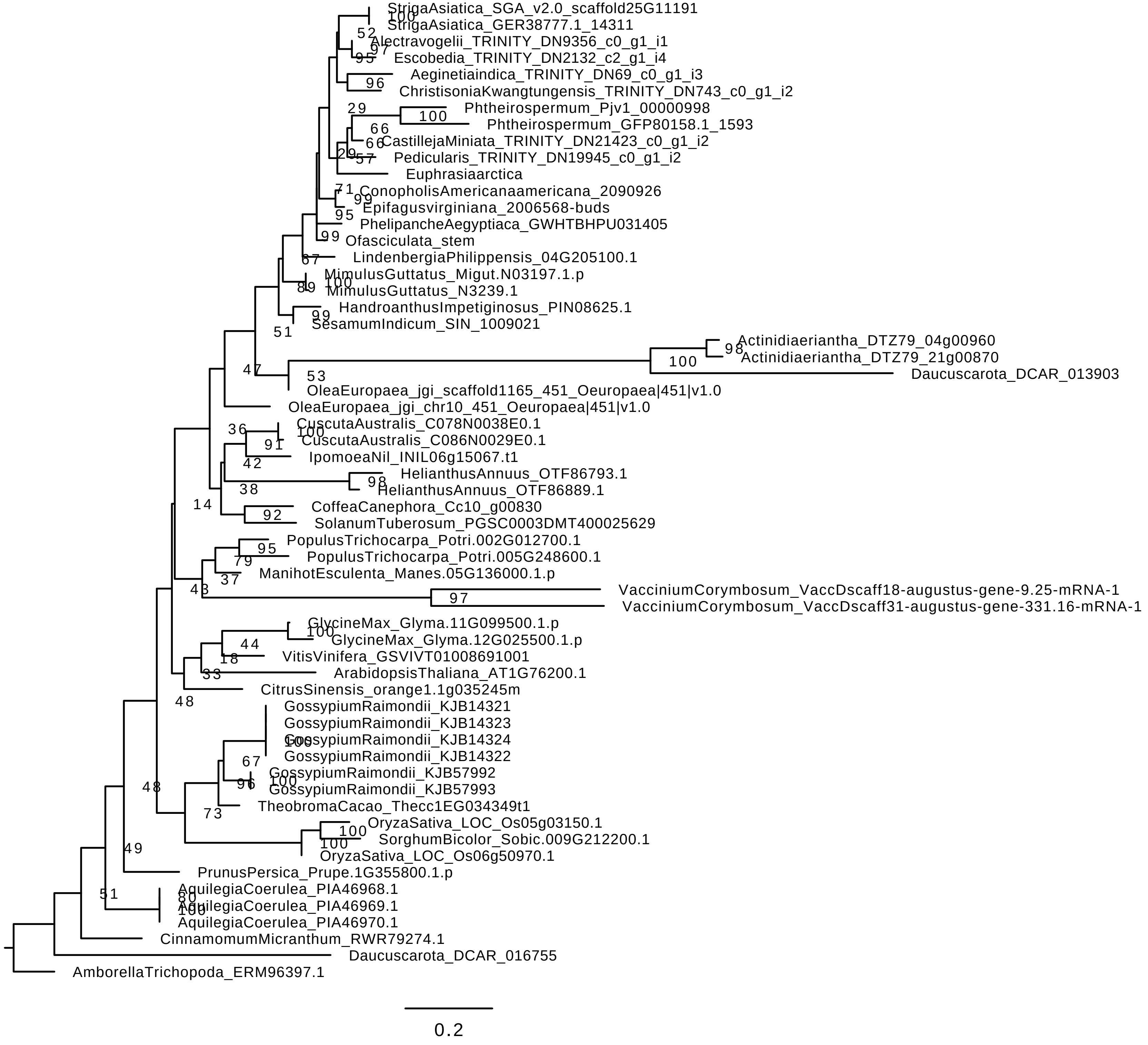
StrigaAsiatica_SGA_v2.0_scaffold25G11191
StrigaAsiatica_GER38777.1_14311
Alectravogelii_TRINITY_DN9356_c0_g1_i1
Escobedia_TRINITY_DN2132_c2_g1_i4
Aeginetiaindica_TRINITY_DN69_c0_g1_i3
ChristisoniaKwangtungensis_TRINITY_DN743_c0_g1_i2
Phtheirospermum_Pjv1_00000998
Phtheirospermum_GFP80158.1_1593
CastillejaMiniata_TRINITY_DN21423_c0_g1_i2
Pedicularis_TRINITY_DN19945_c0_g1_i2
Euphrasiaarctica
ConopholisAmericanaamericana_2090926
Epifagusvirginiana_2006568-buds
PhelipancheAegyptiaca_GWHTBHPU031405
Ofasciculata_stem
LindenbergiaPhilippensis_04G205100.1
MimulusGuttatus_Migut.N03197.1.p
MimulusGuttatus_N3239.1
HandroanthusImpetiginosus_PIN08625.1
SesamumIndicum_SIN_1009021
Actinidiaeriantha_DTZ79_04g00960
Actinidiaeriantha_DTZ79_21g00870
Daucuscarota_DCAR_013903
OleaEuropaea_jgi_scaffold1165_451_Oeuropaea|451|v1.0
OleaEuropaea_jgi_chr10_451_Oeuropaea|451|v1.0
CuscutaAustralis_C078N0038E0.1
CuscutaAustralis_C086N0029E0.1
IpomoeaNil_INIL06g15067.t1
HelianthusAnnuus_OTF86793.1
HelianthusAnnuus_OTF86889.1
CoffeaCanephora_Cc10_g00830
SolanumTuberosum_PGSC0003DMT400025629
PopulusTrichocarpa_Potri.002G012700.1
PopulusTrichocarpa_Potri.005G248600.1
ManihotEsculenta_Manes.05G136000.1.p
VacciniumCorymbosum_VaccDscaff18-augustus-gene-9.25-mRNA-1
VacciniumCorymbosum_VaccDscaff31-augustus-gene-331.16-mRNA-1
GlycineMax_Glyma.11G099500.1.p
GlycineMax_Glyma.12G025500.1.p
VitisVinifera_GSVIVT01008691001
ArabidopsisThaliana_AT1G76200.1
CitrusSinensis_orange1.1g035245m
GossypiumRaimondii_KJB14321
GossypiumRaimondii_KJB14323
GossypiumRaimondii_KJB14324
GossypiumRaimondii_KJB14322
GossypiumRaimondii_KJB57992
GossypiumRaimondii_KJB57993
TheobromaCacao_Thecc1EG034349t1
OryzaSativa_LOC_Os05g03150.1
SorghumBicolor_Sobic.009G212200.1
OryzaSativa_LOC_Os06g50970.1
PrunusPersica_Prupe.1G355800.1.p
AquilegiaCoerulea_PIA46968.1
AquilegiaCoerulea_PIA46969.1
AquilegiaCoerulea_PIA46970.1
CinnamomumMicranthum_RWR79274.1
Daucuscarota_DCAR_016755
AmborellaTrichopoda_ERM96397.1
0.2
