# Supplementary material for: Altered Mitochondrial Respiration Is Associated With Loss of Nuclear‐Encoded OXPHOS Genes in Parasitic Broomrapes
Source: Ecol Evol. 2025 Jul 6;15(7):e71737. doi: 10.1002/ece3.71737 (PMC12230201; doi:10.1002/ece3.71737)
Supplement: Supplementary file 1 — Appendix S1. [file ECE3-15-e71737-s003.tgz › Orobanchaceae_O2K_Supplementary_v3/Data_S1/DataS1/CI/OG0007695.filter.fas.treefile.pdf]

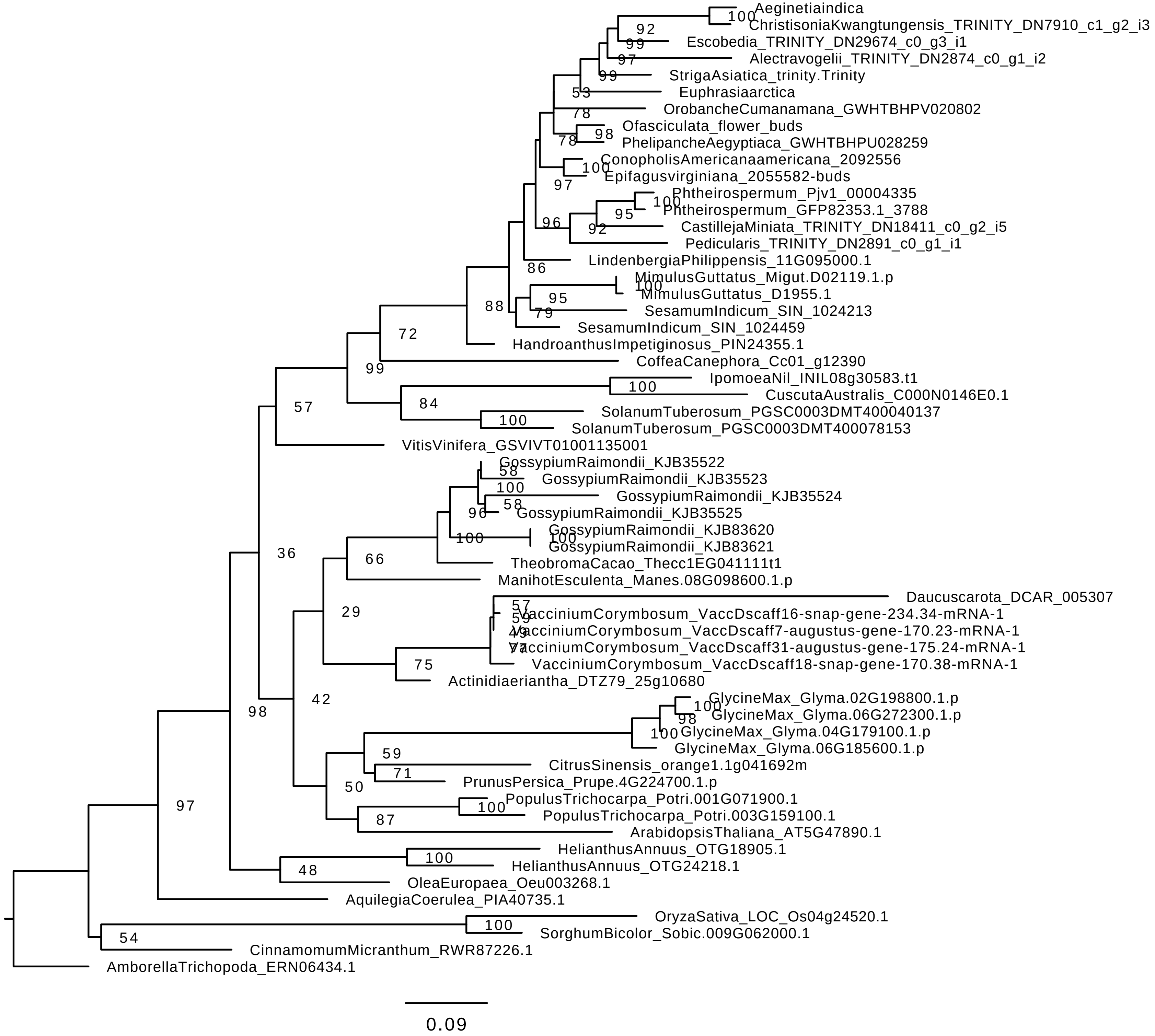
Aeginetiaindica
ChristisoniaKwangtungensis_TRINITY_DN7910_c1_g2_i3
Escobedia_TRINITY_DN29674_c0_g3_i1
Alectravogelii_TRINITY_DN2874_c0_g1_i2
StrigaAsiatica_trinity.Trinity
Euphrasiaarctica
OrobancheCumanamana_GWHTBHPV020802
Ofasciculata_flower_buds
PhelipancheAegyptiaca_GWHTBHPU028259
ConopholisAmericanaamericana_2092556
Epifagusvirginiana_2055582-buds
Phtheirospermum_Pjv1_00004335
Phtheirospermum_GFP82353.1_3788
CastillejaMiniata_TRINITY_DN18411_c0_g2_i5
Pedicularis_TRINITY_DN2891_c0_g1_i1
LindenbergiaPhilippensis_11G095000.1
MimulusGuttatus_Migut.D02119.1.p
MimulusGuttatus_D1955.1
SesamumIndicum_SIN_1024213
SesamumIndicum_SIN_1024459
HandroanthusImpetiginosus_PIN24355.1
CoffeaCanephora_Cc01_g12390
IpomoeaNil_INIL08g30583.t1
CuscutaAustralis_C000N0146E0.1
SolanumTuberosum_PGSC0003DMT400040137
SolanumTuberosum_PGSC0003DMT400078153
VitisVinifera_GSVIVT01001135001
GossypiumRaimondii_KJB35522
GossypiumRaimondii_KJB35523
GossypiumRaimondii_KJB35524
GossypiumRaimondii_KJB35525
GossypiumRaimondii_KJB83620
GossypiumRaimondii_KJB83621
TheobromaCacao_Thecc1EG041111t1
ManihotEsculenta_Manes.08G098600.1.p
Daucuscarota_DCAR_005307
VacciniumCorymbosum_VaccDscaff16-snap-gene-234.34-mRNA-1
VacciniumCorymbosum_VaccDscaff7-augustus-gene-170.23-mRNA-1
VacciniumCorymbosum_VaccDscaff31-augustus-gene-175.24-mRNA-1
VacciniumCorymbosum_VaccDscaff18-snap-gene-170.38-mRNA-1
Actinidiaeriantha_DTZ79_25g10680
GlycineMax_Glyma.02G198800.1.p
GlycineMax_Glyma.06G272300.1.p
GlycineMax_Glyma.04G179100.1.p
GlycineMax_Glyma.06G185600.1.p
CitrusSinensis_orange1.1g041692m
PrunusPersica_Prupe.4G224700.1.p
PopulusTrichocarpa_Potri.001G071900.1
PopulusTrichocarpa_Potri.003G159100.1
ArabidopsisThaliana_AT5G47890.1
HelianthusAnnuus_OTG18905.1
HelianthusAnnuus_OTG24218.1
OleaEuropaea_Oeu003268.1
AquilegiaCoerulea_PIA40735.1
OryzaSativa_LOC_Os04g24520.1
SorghumBicolor_Sobic.009G062000.1
CinnamomumMicranthum_RWR87226.1
AmborellaTrichopoda_ERN06434.1
0.09
