# Supplementary material for: Altered Mitochondrial Respiration Is Associated With Loss of Nuclear‐Encoded OXPHOS Genes in Parasitic Broomrapes
Source: Ecol Evol. 2025 Jul 6;15(7):e71737. doi: 10.1002/ece3.71737 (PMC12230201; doi:10.1002/ece3.71737)
Supplement: Supplementary file 1 — Appendix S1. [file ECE3-15-e71737-s003.tgz › Orobanchaceae_O2K_Supplementary_v3/Data_S1/DataS1/CI/OG0009252.filter.fas.treefile.pdf]

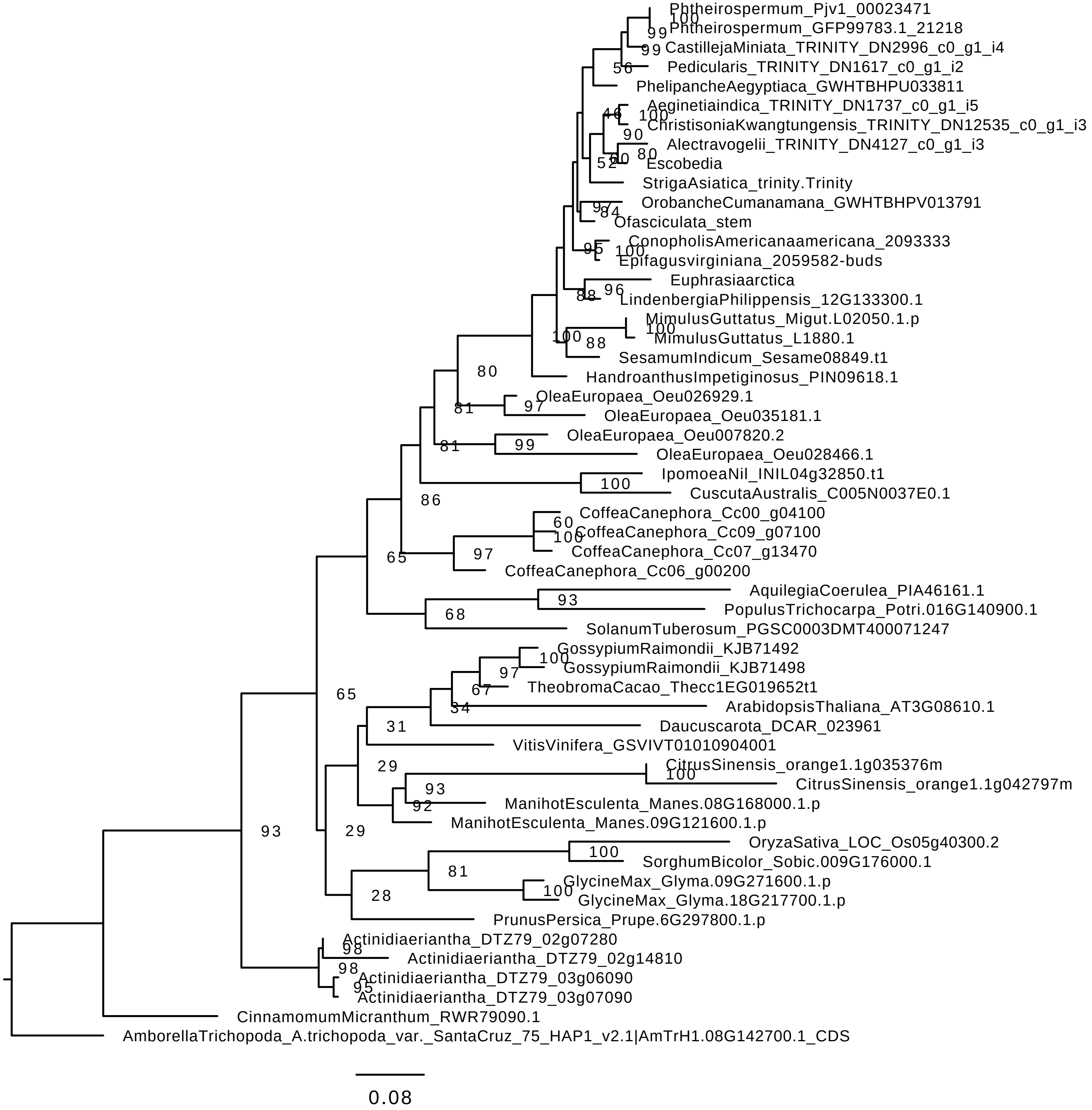

Phtheirospermum_Pjv1_00023471
100
Phtheirospermum_GFP99783.1_21218
99
CastillejaMiniata_TRINITY_DN2996_c0_g1_i4
99
Pedicularis_TRINITY_DN1617_c0_g1_i2
56
PhelipancheAegyptiaca_GWHTBHPU033811
Aeginetiaindica_TRINITY_DN1737_c0_g1_i5
46
100
ChristisoniaKwangtungensis_TRINITY_DN12535_c0_g1_i3
90
Alectravogelii_TRINITY_DN4127_c0_g1_i3
80
Escobedia
52
StrigaAsiatica_trinity.Trinity
OrobancheCumanamana_GWHTBHPV013791
84
Ofasciculata_stem
ConopholisAmericanaamericana_2093333
95
100
Epifagusvirginiana_2059582-buds
Euphrasiaarctica
96
88
LindenbergiaPhilippensis_12G133300.1
MimulusGuttatus_Migut.L02050.1.p
100
100
88
MimulusGuttatus_L1880.1
SesamumIndicum_Sesame08849.t1
80
HandroanthusImpetiginosus_PIN09618.1
OleaEuropaea_Oeu026929.1
81
97
OleaEuropaea_Oeu035181.1
OleaEuropaea_Oeu007820.2
81
99
OleaEuropaea_Oeu028466.1
IpomoeaNil_INIL04g32850.t1
100
CuscutaAustralis_C005N0037E0.1
86
CoffeaCanephora_Cc00_g04100
60
CoffeaCanephora_Cc09_g07100
100
65
97
CoffeaCanephora_Cc07_g13470
CoffeaCanephora_Cc06_g00200
AquilegiaCoerulea_PIA46161.1
93
PopulusTrichocarpa_Potri.016G140900.1
68
SolanumTuberosum_PGSC0003DMT400071247
GossypiumRaimondii_KJB71492
100
97
GossypiumRaimondii_KJB71498
65
67
TheobromaCacao_Thecc1EG019652t1
34
ArabidopsisThaliana_AT3G08610.1
31
Daucuscarota_DCAR_023961
VitisVinifera_GSVIVT01010904001
29
CitrusSinensis_orange1.1g035376m
100
CitrusSinensis_orange1.1g042797m
93
92
ManihotEsculenta_Manes.08G168000.1.p
93
29
ManihotEsculenta_Manes.09G121600.1.p
OryzaSativa_LOC_Os05g40300.2
100
SorghumBicolor_Sobic.009G176000.1
81
GlycineMax_Glyma.09G271600.1.p
100
28
GlycineMax_Glyma.18G217700.1.p
PrunusPersica_Prupe.6G297800.1.p
Actinidiaeriantha_DTZ79_02g07280
98
Actinidiaeriantha_DTZ79_02g14810
98
Actinidiaeriantha_DTZ79_03g06090
95
Actinidiaeriantha_DTZ79_03g07090
CinnamomumMicranthum_RWR79090.1
AmborellaTrichopoda_A.trichopoda_var._SantaCruz_75_HAP1_v2.1|AmTrH1.08G142700.1_CDS
0.08
