# Supplementary material for: Altered Mitochondrial Respiration Is Associated With Loss of Nuclear‐Encoded OXPHOS Genes in Parasitic Broomrapes
Source: Ecol Evol. 2025 Jul 6;15(7):e71737. doi: 10.1002/ece3.71737 (PMC12230201; doi:10.1002/ece3.71737)
Supplement: Supplementary file 1 — Appendix S1. [file ECE3-15-e71737-s003.tgz › Orobanchaceae_O2K_Supplementary_v3/Data_S1/DataS1/CI/OG0008366.filter.fas.treefile.pdf]

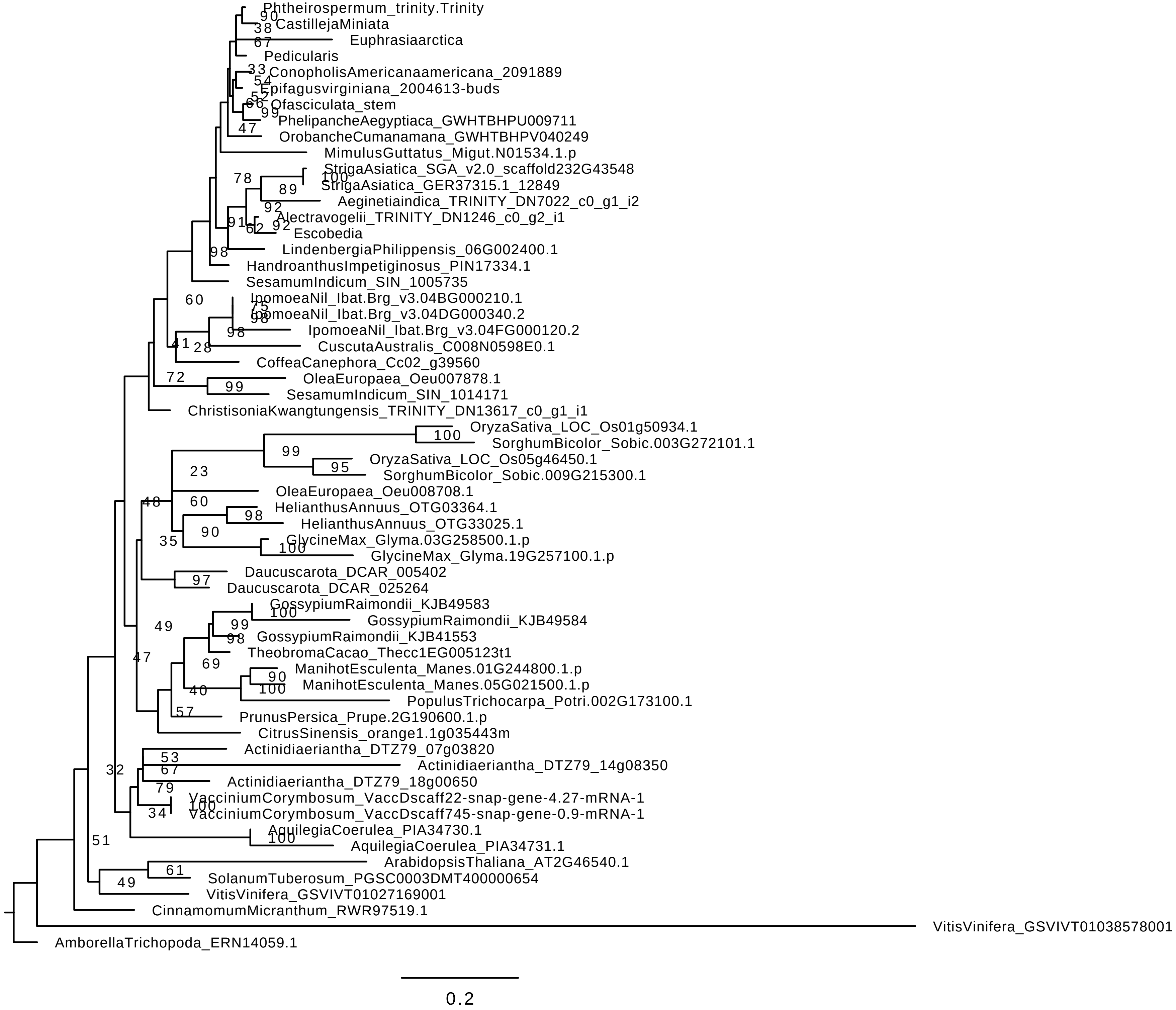

Phtheirospermum_trinity.Trinity
CastillejaMiniata
Euphrasiaarctica
Pedicularis
ConopholisAmericanaamericana_2091889
Epifagusvirginiana_2004613-buds
Ofasciculata_stem
PhelipancheAegyptiaca_GWHTBHPU009711
OrobancheCumanamana_GWHTBHPV040249
MimulusGuttatus_Migut.N01534.1.p
StrigaAsiatica_SGA_v2.0_scaffold232G43548
StrigaAsiatica_GER37315.1_12849
Aeginetiaindica_TRINITY_DN7022_c0_g1_i2
Alectravogelii_TRINITY_DN1246_c0_g2_i1
Escobedia
LindenbergiaPhilippensis_06G002400.1
HandroanthusImpetiginosus_PIN17334.1
SesamumIndicum_SIN_1005735
IpomoeaNil_Ibat.Brg_v3.04BG000210.1
IpomoeaNil_Ibat.Brg_v3.04DG000340.2
IpomoeaNil_Ibat.Brg_v3.04FG000120.2
CuscutaAustralis_C008N0598E0.1
CoffeaCanephora_Cc02_g39560
OleaEuropaea_Oeu007878.1
SesamumIndicum_SIN_1014171
ChristisoniaKwangtungensis_TRINITY_DN13617_c0_g1_i1
OryzaSativa_LOC_Os01g50934.1
SorghumBicolor_Sobic.003G272101.1
OryzaSativa_LOC_Os05g46450.1
SorghumBicolor_Sobic.009G215300.1
OleaEuropaea_Oeu008708.1
HelianthusAnnuus_OTG03364.1
HelianthusAnnuus_OTG33025.1
GlycineMax_Glyma.03G258500.1.p
GlycineMax_Glyma.19G257100.1.p
Daucuscarota_DCAR_005402
Daucuscarota_DCAR_025264
GossypiumRaimondii_KJB49583
GossypiumRaimondii_KJB49584
GossypiumRaimondii_KJB41553
TheobromaCacao_Thecc1EG005123t1
ManihotEsculenta_Manes.01G244800.1.p
ManihotEsculenta_Manes.05G021500.1.p
PopulusTrichocarpa_Potri.002G173100.1
PrunusPersica_Prupe.2G190600.1.p
CitrusSinensis_orange1.1g035443m
Actinidiaeriantha_DTZ79_07g03820
Actinidiaeriantha_DTZ79_14g08350
Actinidiaeriantha_DTZ79_18g00650
VacciniumCorymbosum_VaccDscaff22-snap-gene-4.27-mRNA-1
VacciniumCorymbosum_VaccDscaff745-snap-gene-0.9-mRNA-1
AquilegiaCoerulea_PIA34730.1
AquilegiaCoerulea_PIA34731.1
ArabidopsisThaliana_AT2G46540.1
SolanumTuberosum_PGSC0003DMT400000654
VitisVinifera_GSVIVT01027169001
CinnamomumMicranthum_RWR97519.1
VitisVinifera_GSVIVT01038578001
AmborellaTrichopoda_ERN14059.1
0.2
