# Supplementary material for: Altered Mitochondrial Respiration Is Associated With Loss of Nuclear‐Encoded OXPHOS Genes in Parasitic Broomrapes
Source: Ecol Evol. 2025 Jul 6;15(7):e71737. doi: 10.1002/ece3.71737 (PMC12230201; doi:10.1002/ece3.71737)
Supplement: Supplementary file 1 — Appendix S1. [file ECE3-15-e71737-s003.tgz › Orobanchaceae_O2K_Supplementary_v3/Data_S1/DataS1/CI/OG0008154.filter.fas.treefile.pdf]

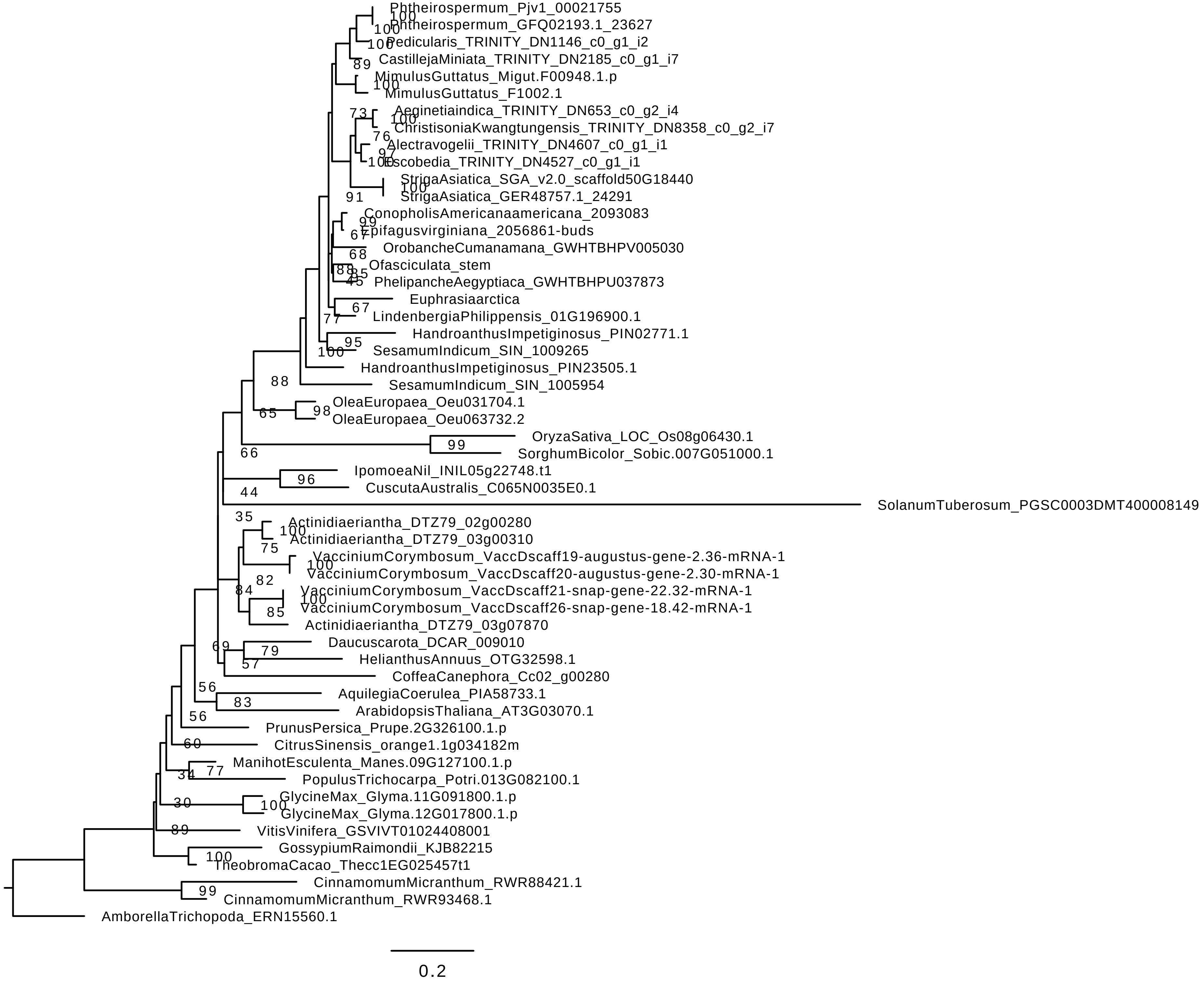
Phtheirospermum_Pjv1_00021755
Phtheirospermum_GFQ02193.1_23627
Pedicularis_TRINITY_DN1146_c0_g1_i2
CastillejaMiniata_TRINITY_DN2185_c0_g1_i7
MimulusGuttatus_Migut.F00948.1.p
MimulusGuttatus_F1002.1
Aeginetiaindica_TRINITY_DN653_c0_g2_i4
ChristisoniaKwangtungensis_TRINITY_DN8358_c0_g2_i7
Alectravogelii_TRINITY_DN4607_c0_g1_i1
Escobedia_TRINITY_DN4527_c0_g1_i1
StrigaAsiatica_SGA_v2.0_scaffold50G18440
StrigaAsiatica_GER48757.1_24291
ConopholisAmericanaamericana_2093083
Epifagusvirginiana_2056861-buds
OrobancheCumanamana_GWHTBHPV005030
Ofasciculata_stem
PhelipancheAegyptiaca_GWHTBHPU037873
Euphrasiaarctica
LindenbergiaPhilippensis_01G196900.1
HandroanthusImpetiginosus_PIN02771.1
SesamumIndicum_SIN_1009265
HandroanthusImpetiginosus_PIN23505.1
SesamumIndicum_SIN_1005954
OleaEuropaea_Oeu031704.1
OleaEuropaea_Oeu063732.2
OryzaSativa_LOC_Os08g06430.1
SorghumBicolor_Sobic.007G051000.1
IpomoeaNil_INIL05g22748.t1
CuscutaAustralis_C065N0035E0.1
SolanumTuberosum_PGSC0003DMT400008149
Actinidiaeriantha_DTZ79_02g00280
Actinidiaeriantha_DTZ79_03g00310
VacciniumCorymbosum_VaccDscaff19-augustus-gene-2.36-mRNA-1
VacciniumCorymbosum_VaccDscaff20-augustus-gene-2.30-mRNA-1
VacciniumCorymbosum_VaccDscaff21-snap-gene-22.32-mRNA-1
VacciniumCorymbosum_VaccDscaff26-snap-gene-18.42-mRNA-1
Actinidiaeriantha_DTZ79_03g07870
Daucuscarota_DCAR_009010
HelianthusAnnuus_OTG32598.1
CoffeaCanephora_Cc02_g00280
AquilegiaCoerulea_PIA58733.1
ArabidopsisThaliana_AT3G03070.1
PrunusPersica_Prupe.2G326100.1.p
CitrusSinensis_orange1.1g034182m
ManihotEsculenta_Manes.09G127100.1.p
PopulusTrichocarpa_Potri.013G082100.1
GlycineMax_Glyma.11G091800.1.p
GlycineMax_Glyma.12G017800.1.p
VitisVinifera_GSVIVT01024408001
GossypiumRaimondii_KJB82215
TheobromaCacao_Thecc1EG025457t1
CinnamomumMicranthum_RWR88421.1
CinnamomumMicranthum_RWR93468.1
AmborellaTrichopoda_ERN15560.1
0.2
