# Supplementary material for: Altered Mitochondrial Respiration Is Associated With Loss of Nuclear‐Encoded OXPHOS Genes in Parasitic Broomrapes
Source: Ecol Evol. 2025 Jul 6;15(7):e71737. doi: 10.1002/ece3.71737 (PMC12230201; doi:10.1002/ece3.71737)
Supplement: Supplementary file 1 — Appendix S1. [file ECE3-15-e71737-s003.tgz › Orobanchaceae_O2K_Supplementary_v3/Data_S1/DataS1/CI/OG0001887.filter.fas.treefile.pdf]

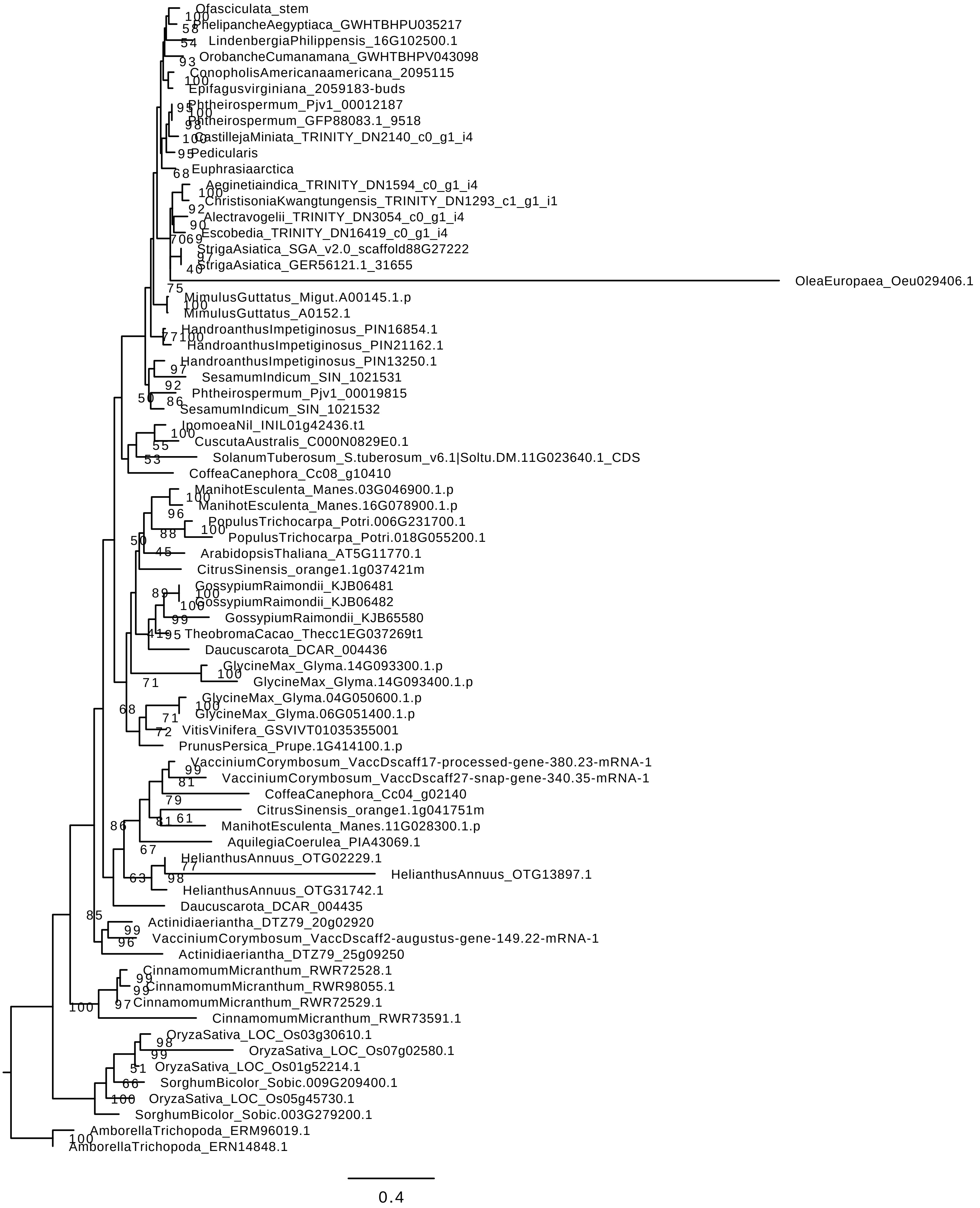

Ofasciculata_stem
PhelipancheAegyptiaca_GWHTBHPU035217
LindenbergiaPhilippensis_16G102500.1
OrobancheCumanamana_GWHTBHPV043098
ConopholisAmericanaamericana_2095115
Epifagusvirginiana_2059183-buds
Phtheirospermum_Pjv1_00012187
Phtheirospermum_GFP88083.1_9518
CastillejaMiniata_TRINITY_DN2140_c0_g1_i4
Pedicularis
Euphrasiaarctica
Aeginetiaindica_TRINITY_DN1594_c0_g1_i4
ChristisoniaKwangtungensis_TRINITY_DN1293_c1_g1_i1
Alectravogelii_TRINITY_DN3054_c0_g1_i4
Escobedia_TRINITY_DN16419_c0_g1_i4
StrigaAsiatica_SGA_v2.0_scaffold88G27222
StrigaAsiatica_GER56121.1_31655
OleaEuropaea_Oeu029406.1
MimulusGuttatus_Migut.A00145.1.p
MimulusGuttatus_A0152.1
HandroanthusImpetiginosus_PIN16854.1
HandroanthusImpetiginosus_PIN21162.1
HandroanthusImpetiginosus_PIN13250.1
SesamumIndicum_SIN_1021531
Phtheirospermum_Pjv1_00019815
SesamumIndicum_SIN_1021532
IpomoeaNil_INIL01g42436.t1
CuscutaAustralis_C000N0829E0.1
SolanumTuberosum_S.tuberosum_v6.1|Soltu.DM.11G023640.1_CDS
CoffeaCanephora_Cc08_g10410
ManihotEsculenta_Manes.03G046900.1.p
ManihotEsculenta_Manes.16G078900.1.p
PopulusTrichocarpa_Potri.006G231700.1
PopulusTrichocarpa_Potri.018G055200.1
ArabidopsisThaliana_AT5G11770.1
CitrusSinensis_orange1.1g037421m
GossypiumRaimondii_KJB06481
GossypiumRaimondii_KJB06482
GossypiumRaimondii_KJB65580
TheobromaCacao_Thecc1EG037269t1
Daucuscarota_DCAR_004436
GlycineMax_Glyma.14G093300.1.p
GlycineMax_Glyma.14G093400.1.p
GlycineMax_Glyma.04G050600.1.p
GlycineMax_Glyma.06G051400.1.p
VitisVinifera_GSVIVT01035355001
PrunusPersica_Prupe.1G414100.1.p
VacciniumCorymbosum_VaccDscaff17-processed-gene-380.23-mRNA-1
VacciniumCorymbosum_VaccDscaff27-snap-gene-340.35-mRNA-1
CoffeaCanephora_Cc04_g02140
CitrusSinensis_orange1.1g041751m
ManihotEsculenta_Manes.11G028300.1.p
AquilegiaCoerulea_PIA43069.1
HelianthusAnnuus_OTG02229.1
HelianthusAnnuus_OTG13897.1
HelianthusAnnuus_OTG31742.1
Daucuscarota_DCAR_004435
Actinidiaeriantha_DTZ79_20g02920
VacciniumCorymbosum_VaccDscaff2-augustus-gene-149.22-mRNA-1
Actinidiaeriantha_DTZ79_25g09250
CinnamomumMicranthum_RWR72528.1
CinnamomumMicranthum_RWR98055.1
CinnamomumMicranthum_RWR72529.1
CinnamomumMicranthum_RWR73591.1
OryzaSativa_LOC_Os03g30610.1
OryzaSativa_LOC_Os07g02580.1
OryzaSativa_LOC_Os01g52214.1
SorghumBicolor_Sobic.009G209400.1
OryzaSativa_LOC_Os05g45730.1
SorghumBicolor_Sobic.003G279200.1
AmborellaTrichopoda_ERM96019.1
AmborellaTrichopoda_ERN14848.1
0.4
