# Supplementary material for: Altered Mitochondrial Respiration Is Associated With Loss of Nuclear‐Encoded OXPHOS Genes in Parasitic Broomrapes
Source: Ecol Evol. 2025 Jul 6;15(7):e71737. doi: 10.1002/ece3.71737 (PMC12230201; doi:10.1002/ece3.71737)
Supplement: Supplementary file 1 — Appendix S1. [file ECE3-15-e71737-s003.tgz › Orobanchaceae_O2K_Supplementary_v3/Data_S1/DataS1/CI/OG0007195.filter.fas.treefile.pdf]

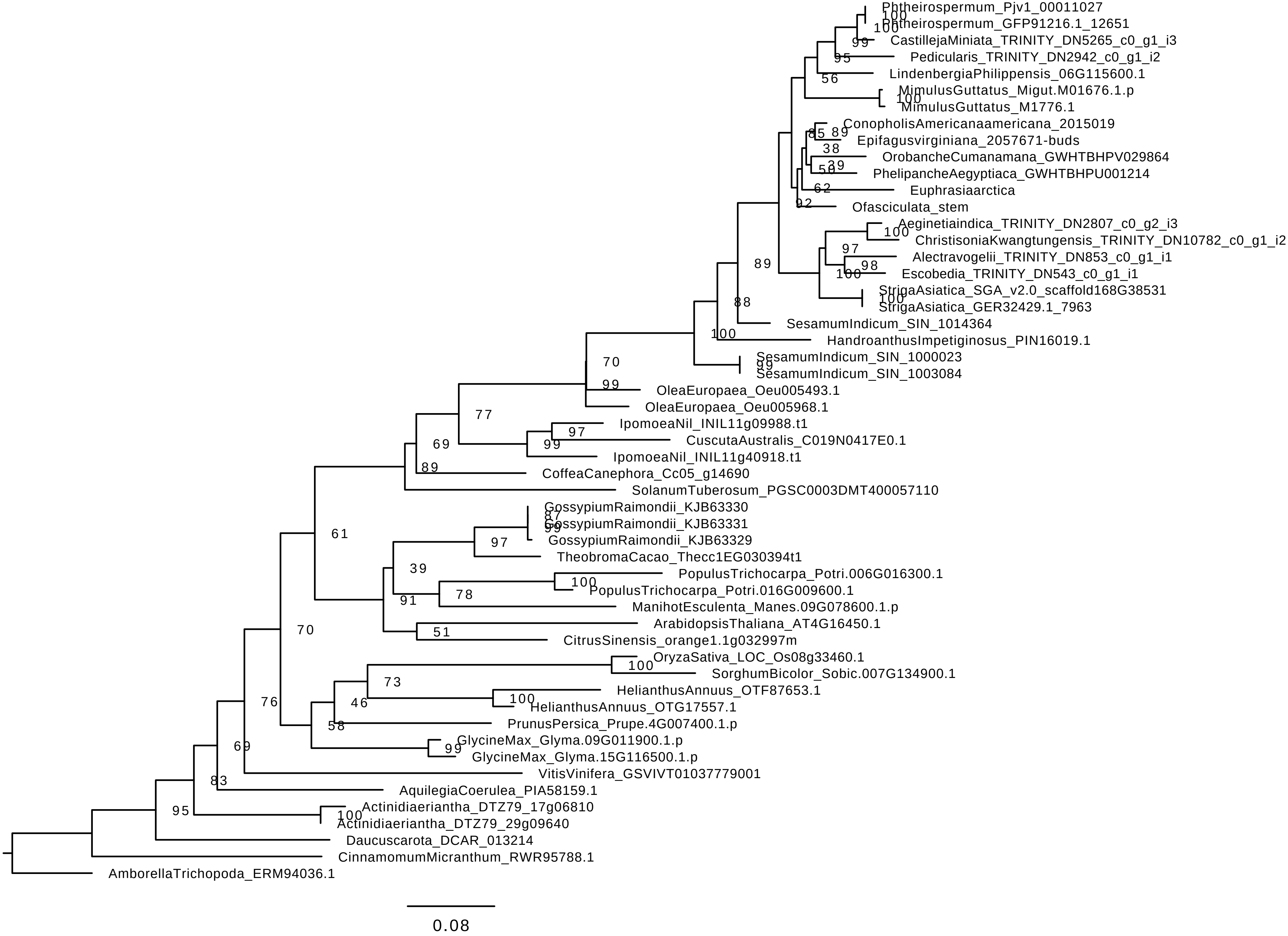
Phtheirospermum_Pjv1_00011027
Phtheirospermum_GFP91216.1_12651
CastillejaMiniata_TRINITY_DN5265_c0_g1_i3
Pedicularis_TRINITY_DN2942_c0_g1_i2
LindenbergiaPhilippensis_06G115600.1
MimulusGuttatus_Migut.M01676.1.p
MimulusGuttatus_M1776.1
ConopholisAmericanaamericana_2015019
Epifagusvirginiana_2057671-buds
OrobancheCumanamana_GWHTBHPV029864
PhelipancheAegyptiaca_GWHTBHPU001214
Euphrasiaarctica
Ofasciculata_stem
Aeginetiaindica_TRINITY_DN2807_c0_g2_i3
ChristisoniaKwangtungensis_TRINITY_DN10782_c0_g1_i2
Alectravogelii_TRINITY_DN853_c0_g1_i1
Escobedia_TRINITY_DN543_c0_g1_i1
StrigaAsiatica_SGA_v2.0_scaffold168G38531
StrigaAsiatica_GER32429.1_7963
SesamumIndicum_SIN_1014364
HandroanthusImpetiginosus_PIN16019.1
SesamumIndicum_SIN_1000023
SesamumIndicum_SIN_1003084
OleaEuropaea_Oeu005493.1
OleaEuropaea_Oeu005968.1
IpomoeaNil_INIL11g09988.t1
CuscutaAustralis_C019N0417E0.1
IpomoeaNil_INIL11g40918.t1
CoffeaCanephora_Cc05_g14690
SolanumTuberosum_PGSC0003DMT400057110
GossypiumRaimondii_KJB63330
GossypiumRaimondii_KJB63331
GossypiumRaimondii_KJB63329
TheobromaCacao_Thecc1EG030394t1
PopulusTrichocarpa_Potri.006G016300.1
PopulusTrichocarpa_Potri.016G009600.1
ManihotEsculenta_Manes.09G078600.1.p
ArabidopsisThaliana_AT4G16450.1
CitrusSinensis_orange1.1g032997m
OryzaSativa_LOC_Os08g33460.1
SorghumBicolor_Sobic.007G134900.1
HelianthusAnnuus_OTF87653.1
HelianthusAnnuus_OTG17557.1
PrunusPersica_Prupe.4G007400.1.p
GlycineMax_Glyma.09G011900.1.p
GlycineMax_Glyma.15G116500.1.p
VitisVinifera_GSVIVT01037779001
AquilegiaCoerulea_PIA58159.1
Actinidiaeriantha_DTZ79_17g06810
Actinidiaeriantha_DTZ79_29g09640
Daucuscarota_DCAR_013214
CinnamomumMicranthum_RWR95788.1
AmborellaTrichopoda_ERM94036.1
0.08
