# Supplementary material for: Altered Mitochondrial Respiration Is Associated With Loss of Nuclear‐Encoded OXPHOS Genes in Parasitic Broomrapes
Source: Ecol Evol. 2025 Jul 6;15(7):e71737. doi: 10.1002/ece3.71737 (PMC12230201; doi:10.1002/ece3.71737)
Supplement: Supplementary file 1 — Appendix S1. [file ECE3-15-e71737-s003.tgz › Orobanchaceae_O2K_Supplementary_v3/Data_S1/DataS1/CI/OG0007352.filter.fas.treefile.pdf]

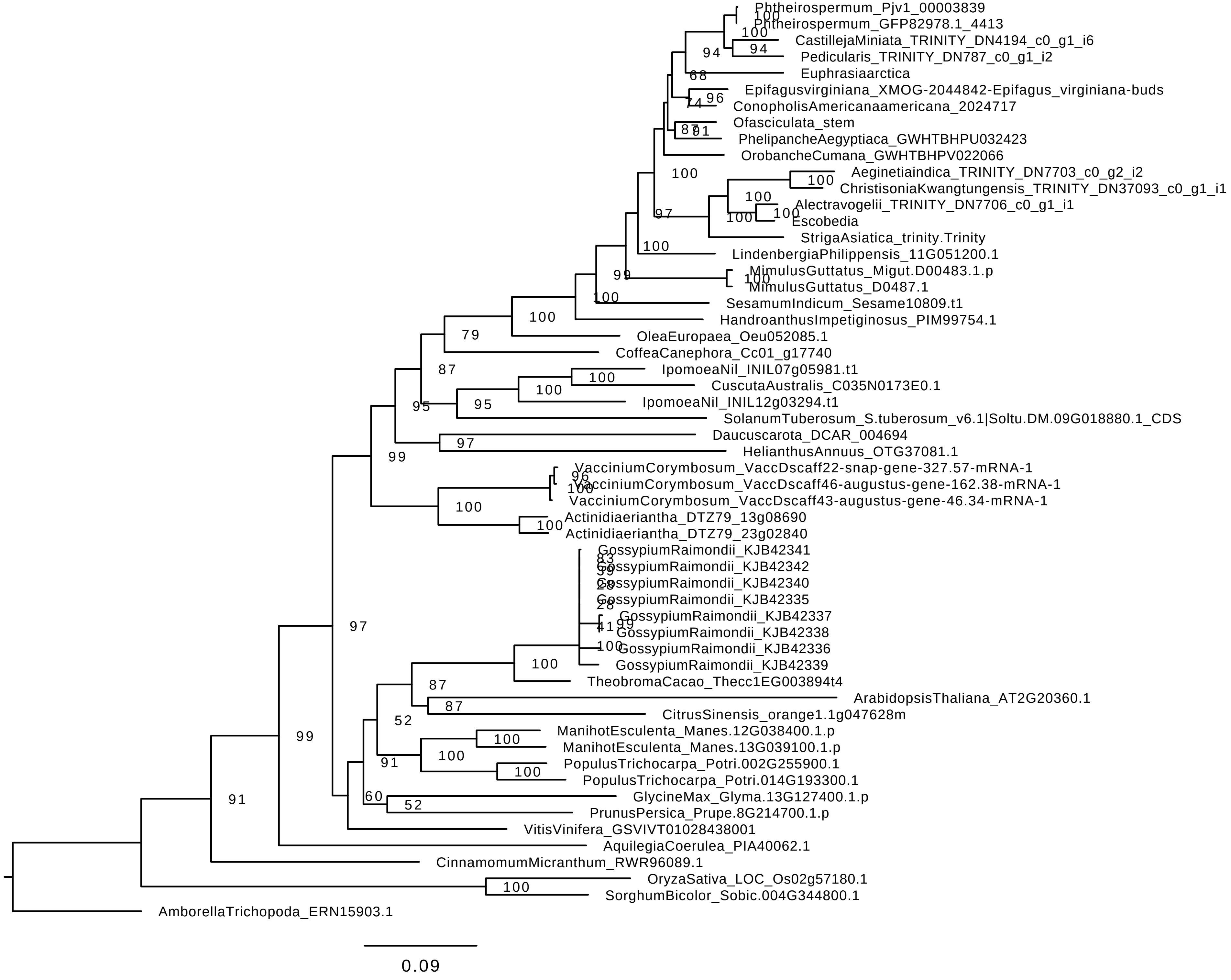
Phtheirospermum_Pjv1_00003839
Phtheirospermum_GFP82978.1_4413
CastillejaMiniata_TRINITY_DN4194_c0_g1_i6
Pedicularis_TRINITY_DN787_c0_g1_i2
Euphrasiaarctica
Epifagusvirginiana_XMOG-2044842-Epifagus_virginiana-buds
ConopholisAmericanaamericana_2024717
Ofasciculata_stem
PhelipancheAegyptiaca_GWHTBHPU032423
OrobancheCumana_GWHTBHPV022066
Aeginetiaindica_TRINITY_DN7703_c0_g2_i2
ChristisoniaKwangtungensis_TRINITY_DN37093_c0_g1_i1
Alectravogelii_TRINITY_DN7706_c0_g1_i1
Escobedia
StrigaAsiatica_trinity.Trinity
LindenbergiaPhilippensis_11G051200.1
MimulusGuttatus_Migut.D00483.1.p
MimulusGuttatus_D0487.1
SesamumIndicum_Sesame10809.t1
HandroanthusImpetiginosus_PIM99754.1
OleaEuropaea_Oeu052085.1
CoffeaCanephora_Cc01_g17740
IpomoeaNil_INIL07g05981.t1
CuscutaAustralis_C035N0173E0.1
IpomoeaNil_INIL12g03294.t1
SolanumTuberosum_S.tuberosum_v6.1|Soltu.DM.09G018880.1_CDS
Daucuscarota_DCAR_004694
HelianthusAnnuus_OTG37081.1
VacciniumCorymbosum_VaccDscaff22-snap-gene-327.57-mRNA-1
VacciniumCorymbosum_VaccDscaff46-augustus-gene-162.38-mRNA-1
VacciniumCorymbosum_VaccDscaff43-augustus-gene-46.34-mRNA-1
Actinidiaeriantha_DTZ79_13g08690
Actinidiaeriantha_DTZ79_23g02840
GossypiumRaimondii_KJB42341
GossypiumRaimondii_KJB42342
GossypiumRaimondii_KJB42340
GossypiumRaimondii_KJB42335
GossypiumRaimondii_KJB42337
GossypiumRaimondii_KJB42338
GossypiumRaimondii_KJB42336
GossypiumRaimondii_KJB42339
TheobromaCacao_Thecc1EG003894t4
ArabidopsisThaliana_AT2G20360.1
CitrusSinensis_orange1.1g047628m
ManihotEsculenta_Manes.12G038400.1.p
ManihotEsculenta_Manes.13G039100.1.p
PopulusTrichocarpa_Potri.002G255900.1
PopulusTrichocarpa_Potri.014G193300.1
GlycineMax_Glyma.13G127400.1.p
PrunusPersica_Prupe.8G214700.1.p
VitisVinifera_GSVIVT01028438001
AquilegiaCoerulea_PIA40062.1
CinnamomumMicranthum_RWR96089.1
OryzaSativa_LOC_Os02g57180.1
SorghumBicolor_Sobic.004G344800.1
AmborellaTrichopoda_ERN15903.1
0.09
