# Supplementary material for: Altered Mitochondrial Respiration Is Associated With Loss of Nuclear‐Encoded OXPHOS Genes in Parasitic Broomrapes
Source: Ecol Evol. 2025 Jul 6;15(7):e71737. doi: 10.1002/ece3.71737 (PMC12230201; doi:10.1002/ece3.71737)
Supplement: Supplementary file 1 — Appendix S1. [file ECE3-15-e71737-s003.tgz › Orobanchaceae_O2K_Supplementary_v3/Data_S1/DataS1/CI/OG0009020.filter.fas.treefile.pdf]

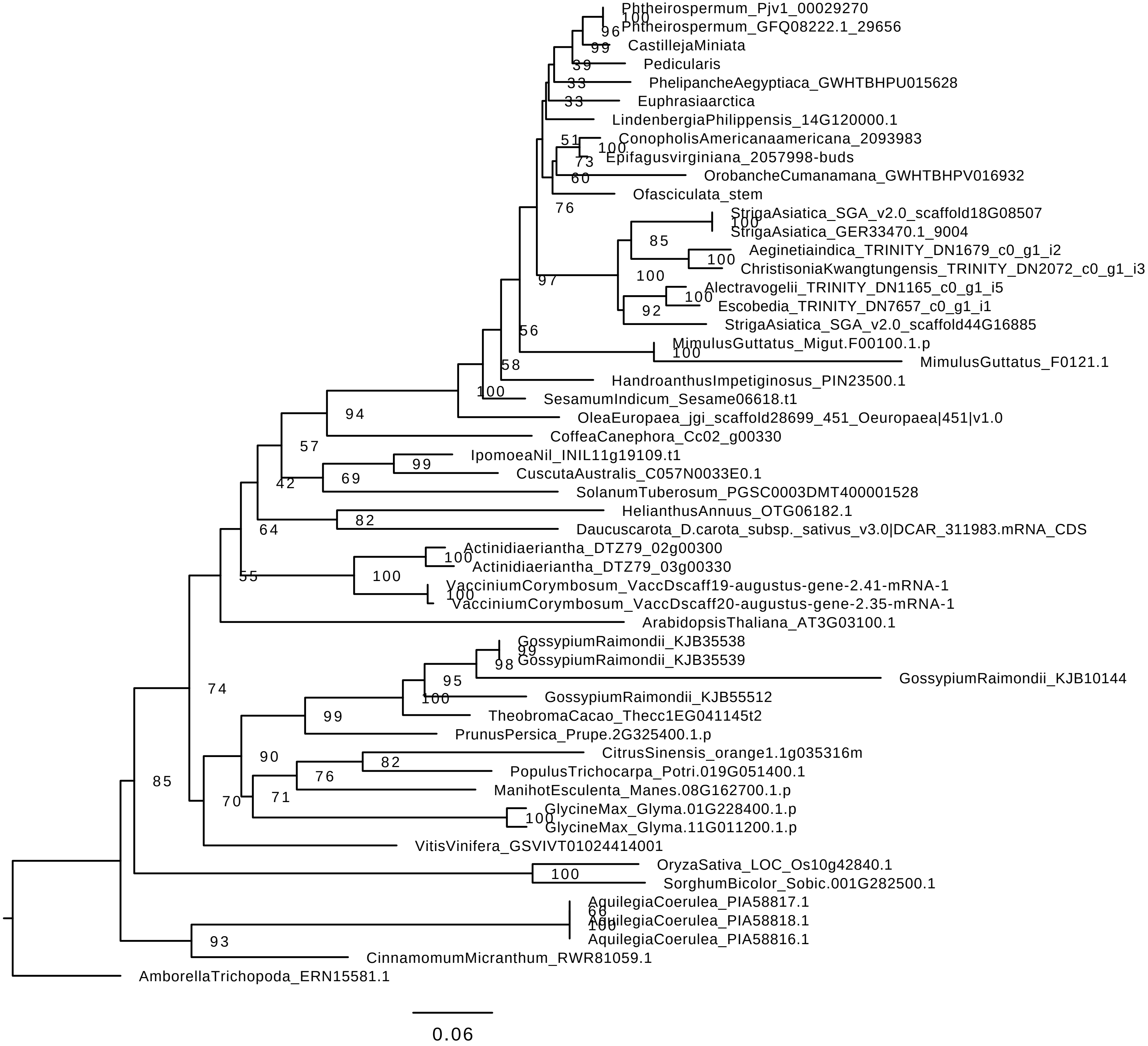
Phtheirospermum_Pjv1_00029270
Phtheirospermum_GFQ08222.1_29656
CastillejaMiniata
Pedicularis
PhelipancheAegyptiaca_GWHTBHPU015628
Euphrasiaarctica
LindenbergiaPhilippensis_14G120000.1
ConopholisAmericanaamericana_2093983
Epifagusvirginiana_2057998-buds
OrobancheCumanamana_GWHTBHPV016932
Ofasciculata_stem
StrigaAsiatica_SGA_v2.0_scaffold18G08507
StrigaAsiatica_GER33470.1_9004
Aeginetiaindica_TRINITY_DN1679_c0_g1_i2
ChristisoniaKwangtungensis_TRINITY_DN2072_c0_g1_i3
Alectravogelii_TRINITY_DN1165_c0_g1_i5
Escobedia_TRINITY_DN7657_c0_g1_i1
StrigaAsiatica_SGA_v2.0_scaffold44G16885
MimulusGuttatus_Migut.F00100.1.p
MimulusGuttatus_F0121.1
HandroanthusImpetiginosus_PIN23500.1
SesamumIndicum_Sesame06618.t1
OleaEuropaea_jgi_scaffold28699_451_Oeuropaea|451|v1.0
CoffeaCanephora_Cc02_g00330
IpomoeaNil_INIL11g19109.t1
CuscutaAustralis_C057N0033E0.1
SolanumTuberosum_PGSC0003DMT400001528
HelianthusAnnuus_OTG06182.1
Daucuscarota_D.carota_subsp._sativus_v3.0|DCAR_311983.mRNA_CDS
Actinidiaeriantha_DTZ79_02g00300
Actinidiaeriantha_DTZ79_03g00330
VacciniumCorymbosum_VaccDscaff19-augustus-gene-2.41-mRNA-1
VacciniumCorymbosum_VaccDscaff20-augustus-gene-2.35-mRNA-1
ArabidopsisThaliana_AT3G03100.1
GossypiumRaimondii_KJB35538
GossypiumRaimondii_KJB35539
GossypiumRaimondii_KJB10144
GossypiumRaimondii_KJB55512
TheobromaCacao_Thecc1EG041145t2
PrunusPersica_Prupe.2G325400.1.p
CitrusSinensis_orange1.1g035316m
PopulusTrichocarpa_Potri.019G051400.1
ManihotEsculenta_Manes.08G162700.1.p
GlycineMax_Glyma.01G228400.1.p
GlycineMax_Glyma.11G011200.1.p
VitisVinifera_GSVIVT01024414001
OryzaSativa_LOC_Os10g42840.1
SorghumBicolor_Sobic.001G282500.1
AquilegiaCoerulea_PIA58817.1
AquilegiaCoerulea_PIA58818.1
AquilegiaCoerulea_PIA58816.1
CinnamomumMicranthum_RWR81059.1
AmborellaTrichopoda_ERN15581.1
0.06
