# Supplementary material for: Altered Mitochondrial Respiration Is Associated With Loss of Nuclear‐Encoded OXPHOS Genes in Parasitic Broomrapes
Source: Ecol Evol. 2025 Jul 6;15(7):e71737. doi: 10.1002/ece3.71737 (PMC12230201; doi:10.1002/ece3.71737)
Supplement: Supplementary file 1 — Appendix S1. [file ECE3-15-e71737-s003.tgz › Orobanchaceae_O2K_Supplementary_v3/Data_S1/DataS1/CI/OG0007747.filter.fas.treefile.pdf]

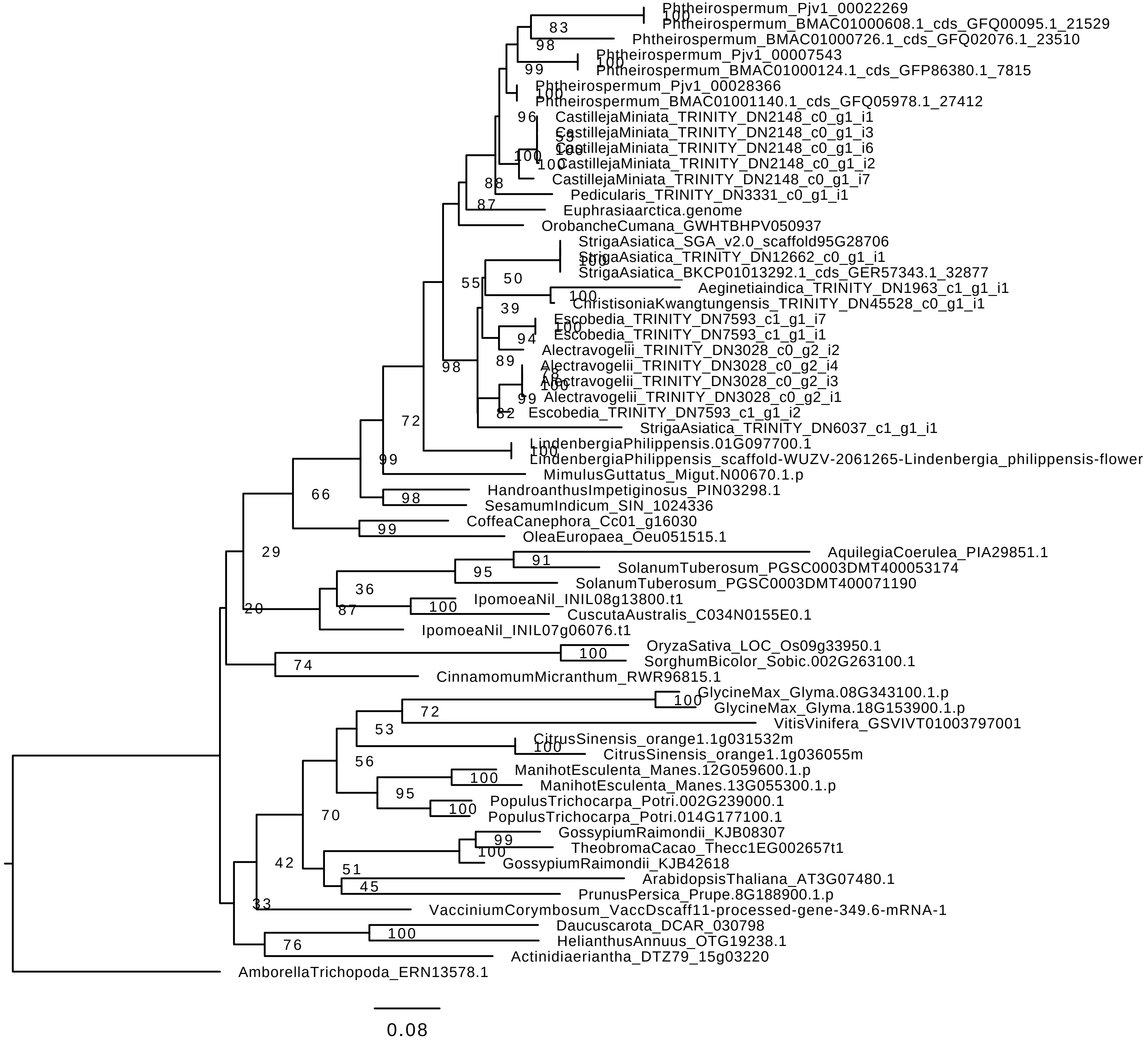

Phtheirospermum_Pjv1_00022269
Phtheirospermum_BMAC01000608.1_cds_GFQ00095.1_21529
Phtheirospermum_BMAC01000726.1_cds_GFQ02076.1_23510
Phtheirospermum_Pjv1_00007543
Phtheirospermum_BMAC01000124.1_cds_GFP86380.1_7815
Phtheirospermum_Pjv1_00028366
Phtheirospermum_BMAC01001140.1_cds_GFQ05978.1_27412
CastillejaMiniata_TRINITY_DN2148_c0_g1_i1
CastillejaMiniata_TRINITY_DN2148_c0_g1_i3
CastillejaMiniata_TRINITY_DN2148_c0_g1_i6
CastillejaMiniata_TRINITY_DN2148_c0_g1_i2
CastillejaMiniata_TRINITY_DN2148_c0_g1_i7
Pedicularis_TRINITY_DN3331_c0_g1_i1
Euphrasiaarctica.genome
OrobancheCumana_GWHTBHPV050937
StrigaAsiatica_SGA_v2.0_scaffold95G28706
StrigaAsiatica_TRINITY_DN12662_c0_g1_i1
StrigaAsiatica_BKCP01013292.1_cds_GER57343.1_32877
Aeginetiaindica_TRINITY_DN1963_c1_g1_i1
ChristisoniaKwangtungensis_TRINITY_DN45528_c0_g1_i1
Escobedia_TRINITY_DN7593_c1_g1_i7
Escobedia_TRINITY_DN7593_c1_g1_i1
Alectravogelii_TRINITY_DN3028_c0_g2_i2
Alectravogelii_TRINITY_DN3028_c0_g2_i4
Alectravogelii_TRINITY_DN3028_c0_g2_i3
Alectravogelii_TRINITY_DN3028_c0_g2_i1
Escobedia_TRINITY_DN7593_c1_g1_i2
StrigaAsiatica_TRINITY_DN6037_c1_g1_i1
LindenbergiaPhilippensis.01G097700.1
LindenbergiaPhilippensis_scaffold-WUZV-2061265-Lindenbergia_philippensis-flower
MimulusGuttatus_Migut.N00670.1.p
HandroanthusImpetiginosus_PIN03298.1
SesamumIndicum_SIN_1024336
CoffeaCanephora_Cc01_g16030
OleaEuropaea_Oeu051515.1
AquilegiaCoerulea_PIA29851.1
SolanumTuberosum_PGSC0003DMT400053174
SolanumTuberosum_PGSC0003DMT400071190
IpomoeaNil_INIL08g13800.t1
CuscutaAustralis_C034N0155E0.1
IpomoeaNil_INIL07g06076.t1
OryzaSativa_LOC_Os09g33950.1
SorghumBicolor_Sobic.002G263100.1
CinnamomumMicranthum_RWR96815.1
GlycineMax_Glyma.08G343100.1.p
GlycineMax_Glyma.18G153900.1.p
VitisVinifera_GSVIVT01003797001
CitrusSinensis_orange1.1g031532m
CitrusSinensis_orange1.1g036055m
ManihotEsculenta_Manes.12G059600.1.p
ManihotEsculenta_Manes.13G055300.1.p
PopulusTrichocarpa_Potri.002G239000.1
PopulusTrichocarpa_Potri.014G177100.1
GossypiumRaimondii_KJB08307
TheobromaCacao_Thecc1EG002657t1
GossypiumRaimondii_KJB42618
ArabidopsisThaliana_AT3G07480.1
PrunusPersica_Prupe.8G188900.1.p
VacciniumCorymbosum_VaccDscaff11-processed-gene-349.6-mRNA-1
Daucuscarota_DCAR_030798
HelianthusAnnuus_OTG19238.1
Actinidiaeriantha_DTZ79_15g03220
AmborellaTrichopoda_ERN13578.1
0.08
