# Supplementary material for: Altered Mitochondrial Respiration Is Associated With Loss of Nuclear‐Encoded OXPHOS Genes in Parasitic Broomrapes
Source: Ecol Evol. 2025 Jul 6;15(7):e71737. doi: 10.1002/ece3.71737 (PMC12230201; doi:10.1002/ece3.71737)
Supplement: Supplementary file 1 — Appendix S1. [file ECE3-15-e71737-s003.tgz › Orobanchaceae_O2K_Supplementary_v3/Data_S1/DataS1/CI/OG0005154.filter.fas.treefile.pdf]

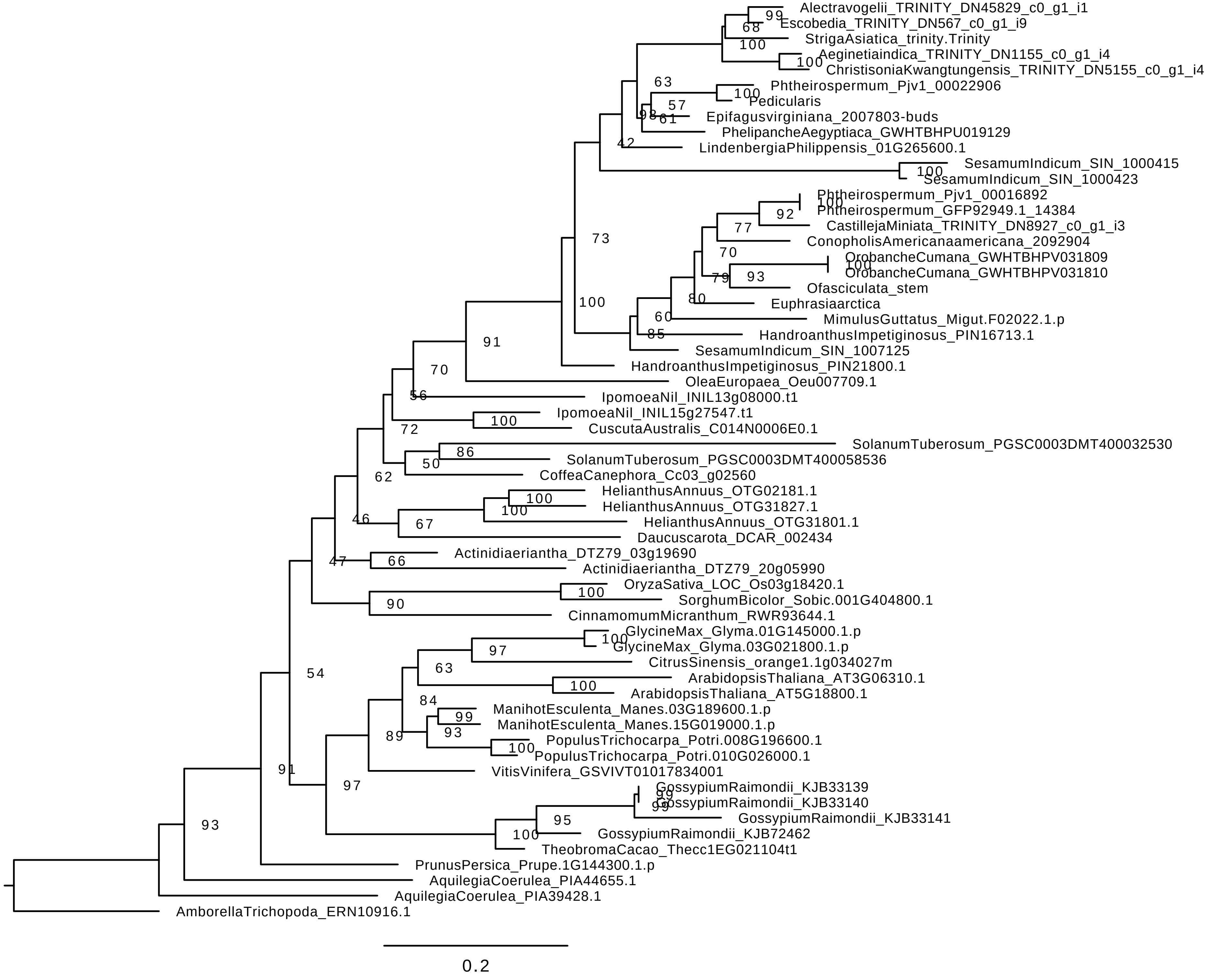
Alectravogelii_TRINITY_DN45829_c0_g1_i1
Escobedia_TRINITY_DN567_c0_g1_i9
StrigaAsiatica_trinity.Trinity
Aeginetiaindica_TRINITY_DN1155_c0_g1_i4
ChristisoniaKwangtungensis_TRINITY_DN5155_c0_g1_i4
Phtheirospermum_Pjv1_00022906
Pedicularis
Epifagusvirginiana_2007803-buds
PhelipancheAegyptiaca_GWHTBHPU019129
LindenbergiaPhilippensis_01G265600.1
SesamumIndicum_SIN_1000415
SesamumIndicum_SIN_1000423
Phtheirospermum_Pjv1_00016892
Phtheirospermum_GFP92949.1_14384
CastillejaMiniata_TRINITY_DN8927_c0_g1_i3
ConopholisAmericanaamericana_2092904
OrobancheCumana_GWHTBHPV031809
OrobancheCumana_GWHTBHPV031810
Ofasciculata_stem
Euphrasiaarctica
MimulusGuttatus_Migut.F02022.1.p
HandroanthusImpetiginosus_PIN16713.1
SesamumIndicum_SIN_1007125
HandroanthusImpetiginosus_PIN21800.1
OleaEuropaea_Oeu007709.1
IpomoeaNil_INIL13g08000.t1
IpomoeaNil_INIL15g27547.t1
CuscutaAustralis_C014N0006E0.1
SolanumTuberosum_PGSC0003DMT400032530
SolanumTuberosum_PGSC0003DMT400058536
CoffeaCanephora_Cc03_g02560
HelianthusAnnuus_OTG02181.1
HelianthusAnnuus_OTG31827.1
HelianthusAnnuus_OTG31801.1
Daucuscarota_DCAR_002434
Actinidiaeriantha_DTZ79_03g19690
Actinidiaeriantha_DTZ79_20g05990
OryzaSativa_LOC_Os03g18420.1
SorghumBicolor_Sobic.001G404800.1
CinnamomumMicranthum_RWR93644.1
GlycineMax_Glyma.01G145000.1.p
GlycineMax_Glyma.03G021800.1.p
CitrusSinensis_orange1.1g034027m
ArabidopsisThaliana_AT3G06310.1
ArabidopsisThaliana_AT5G18800.1
ManihotEsculenta_Manes.03G189600.1.p
ManihotEsculenta_Manes.15G019000.1.p
PopulusTrichocarpa_Potri.008G196600.1
PopulusTrichocarpa_Potri.010G026000.1
VitisVinifera_GSVIVT01017834001
GossypiumRaimondii_KJB33139
GossypiumRaimondii_KJB33140
GossypiumRaimondii_KJB33141
GossypiumRaimondii_KJB72462
TheobromaCacao_Thecc1EG021104t1
PrunusPersica_Prupe.1G144300.1.p
AquilegiaCoerulea_PIA44655.1
AquilegiaCoerulea_PIA39428.1
AmborellaTrichopoda_ERN10916.1
0.2
