# Supplementary material for: Altered Mitochondrial Respiration Is Associated With Loss of Nuclear‐Encoded OXPHOS Genes in Parasitic Broomrapes
Source: Ecol Evol. 2025 Jul 6;15(7):e71737. doi: 10.1002/ece3.71737 (PMC12230201; doi:10.1002/ece3.71737)
Supplement: Supplementary file 1 — Appendix S1. [file ECE3-15-e71737-s003.tgz › Orobanchaceae_O2K_Supplementary_v3/Data_S1/DataS1/CI/OG0007003.filter.fas.treefile.pdf]

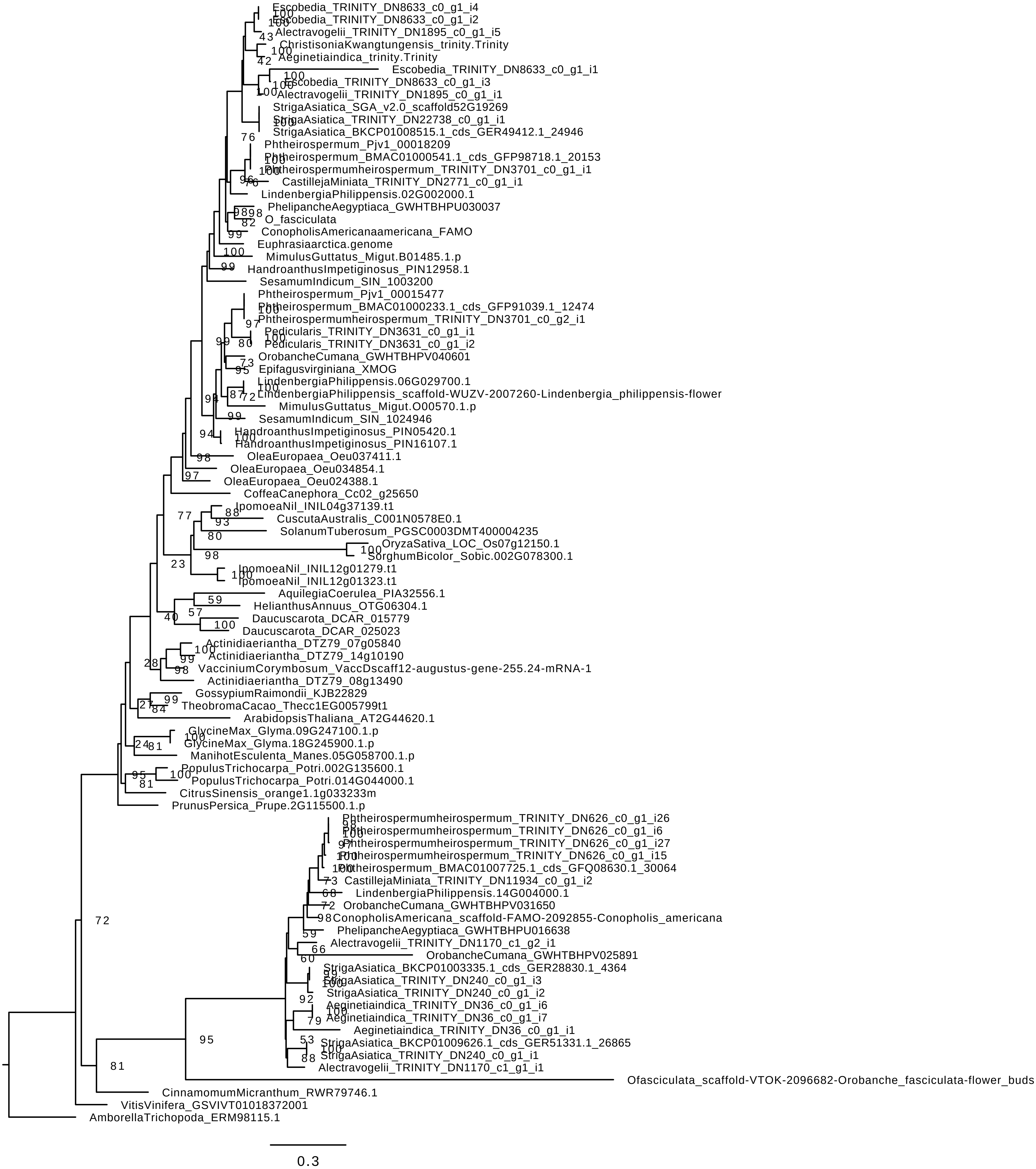
Escobedia_TRINITY_DN8633_c0_g1_i4
Escobedia_TRINITY_DN8633_c0_g1_i2
Alectravogelii_TRINITY_DN1895_c0_g1_i5
ChristisoniaKwangtungensis_trinity.Trinity
Aeginetiaindica_trinity.Trinity
Escobedia_TRINITY_DN8633_c0_g1_i1
Escobedia_TRINITY_DN8633_c0_g1_i3
Alectravogelii_TRINITY_DN1895_c0_g1_i1
StrigaAsiatica_SGA_v2.0_scaffold52G19269
StrigaAsiatica_TRINITY_DN22738_c0_g1_i1
StrigaAsiatica_BKCP01008515.1_cds_GER49412.1_24946
Phtheirospermum_Pjv1_00018209
Phtheirospermum_BMAC01000541.1_cds_GFP98718.1_20153
Phtheirospermumheirospermum_TRINITY_DN3701_c0_g1_i1
CastillejaMiniata_TRINITY_DN2771_c0_g1_i1
LindenbergiaPhilippensis.02G002000.1
PhelipancheAegyptiaca_GWHTBHPU030037
O_fasciculata
ConopholisAmericanaamericana_FAMO
Euphrasiaarctica.genome
MimulusGuttatus_Migut.B01485.1.p
HandroanthusImpetiginosus_PIN12958.1
SesamumIndicum_SIN_1003200
Phtheirospermum_Pjv1_00015477
Phtheirospermum_BMAC01000233.1_cds_GFP91039.1_12474
Phtheirospermumheirospermum_TRINITY_DN3701_c0_g2_i1
Pedicularis_TRINITY_DN3631_c0_g1_i1
Pedicularis_TRINITY_DN3631_c0_g1_i2
OrobancheCumana_GWHTBHPV040601
Epifagusvirginiana_XMOG
LindenbergiaPhilippensis.06G029700.1
LindenbergiaPhilippensis_scaffold-WUZV-2007260-Lindenbergia_philippensis-flower
MimulusGuttatus_Migut.O00570.1.p
SesamumIndicum_SIN_1024946
HandroanthusImpetiginosus_PIN05420.1
HandroanthusImpetiginosus_PIN16107.1
OleaEuropaea_Oeu037411.1
OleaEuropaea_Oeu034854.1
OleaEuropaea_Oeu024388.1
CoffeaCanephora_Cc02_g25650
IpomoeaNil_INIL04g37139.t1
CuscutaAustralis_C001N0578E0.1
SolanumTuberosum_PGSC0003DMT400004235
OryzaSativa_LOC_Os07g12150.1
SorghumBicolor_Sobic.002G078300.1
IpomoeaNil_INIL12g01279.t1
IpomoeaNil_INIL12g01323.t1
AquilegiaCoerulea_PIA32556.1
HelianthusAnnuus_OTG06304.1
Daucuscarota_DCAR_015779
Daucuscarota_DCAR_025023
Actinidiaeriantha_DTZ79_07g05840
Actinidiaeriantha_DTZ79_14g10190
VacciniumCorymbosum_VaccDscaff12-augustus-gene-255.24-mRNA-1
Actinidiaeriantha_DTZ79_08g13490
GossypiumRaimondii_KJB22829
TheobromaCacao_Thecc1EG005799t1
ArabidopsisThaliana_AT2G44620.1
GlycineMax_Glyma.09G247100.1.p
GlycineMax_Glyma.18G245900.1.p
ManihotEsculenta_Manes.05G058700.1.p
PopulusTrichocarpa_Potri.002G135600.1
PopulusTrichocarpa_Potri.014G044000.1
CitrusSinensis_orange1.1g033233m
PrunusPersica_Prupe.2G115500.1.p
Phtheirospermumheirospermum_TRINITY_DN626_c0_g1_i26
Phtheirospermumheirospermum_TRINITY_DN626_c0_g1_i6
Phtheirospermumheirospermum_TRINITY_DN626_c0_g1_i27
Phtheirospermumheirospermum_TRINITY_DN626_c0_g1_i15
Phtheirospermum_BMAC01007725.1_cds_GFQ08630.1_30064
CastillejaMiniata_TRINITY_DN11934_c0_g1_i2
LindenbergiaPhilippensis.14G004000.1
OrobancheCumana_GWHTBHPV031650
ConopholisAmericana_scaffold-FAMO-2092855-Conopholis_americana
PhelipancheAegyptiaca_GWHTBHPU016638
Alectravogelii_TRINITY_DN1170_c1_g2_i1
OrobancheCumana_GWHTBHPV025891
StrigaAsiatica_BKCP01003335.1_cds_GER28830.1_4364
StrigaAsiatica_TRINITY_DN240_c0_g1_i3
StrigaAsiatica_TRINITY_DN240_c0_g1_i2
Aeginetiaindica_TRINITY_DN36_c0_g1_i6
Aeginetiaindica_TRINITY_DN36_c0_g1_i7
Aeginetiaindica_TRINITY_DN36_c0_g1_i1
StrigaAsiatica_BKCP01009626.1_cds_GER51331.1_26865
StrigaAsiatica_TRINITY_DN240_c0_g1_i1
Alectravogelii_TRINITY_DN1170_c1_g1_i1
Ofasciculata_scaffold-VTOK-2096682-Orobanche_fasciculata-flower_buds
CinnamomumMicranthum_RWR79746.1
VitisVinifera_GSVIVT01018372001
AmborellaTrichopoda_ERM98115.1
0.3
