# Supplementary material for: Altered Mitochondrial Respiration Is Associated With Loss of Nuclear‐Encoded OXPHOS Genes in Parasitic Broomrapes
Source: Ecol Evol. 2025 Jul 6;15(7):e71737. doi: 10.1002/ece3.71737 (PMC12230201; doi:10.1002/ece3.71737)
Supplement: Supplementary file 1 — Appendix S1. [file ECE3-15-e71737-s003.tgz › Orobanchaceae_O2K_Supplementary_v3/Data_S1/DataS1/CI/OG0005589.filter.fas.treefile.pdf]

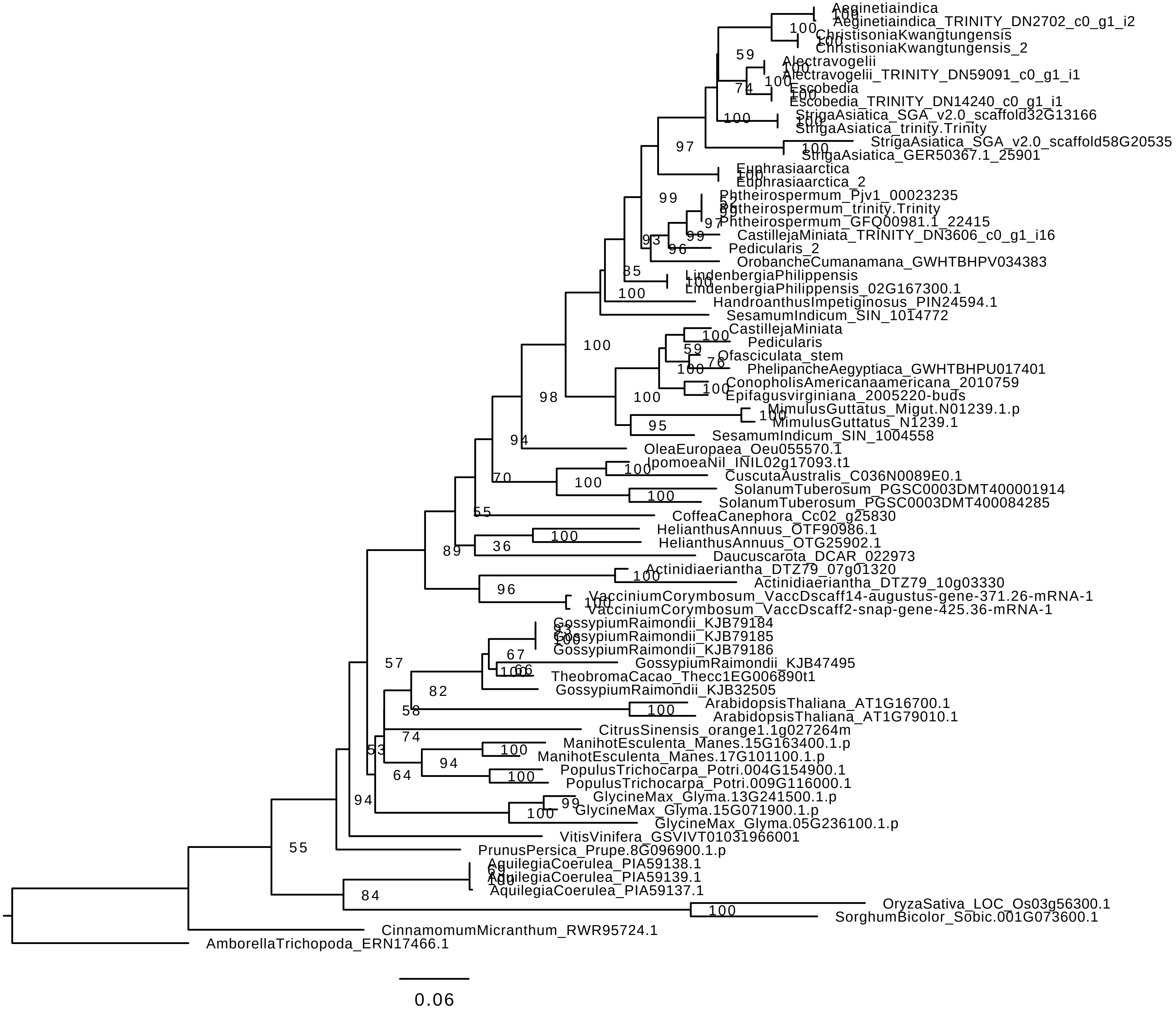
Aeginetiaindica
Aeginetiaindica_TRINITY_DN2702_c0_g1_i2
ChristisoniaKwangtungensis
ChristisoniaKwangtungensis_2
Alectravogelii
Alectravogelii_TRINITY_DN59091_c0_g1_i1
Escobedia
Escobedia_TRINITY_DN14240_c0_g1_i1
StrigaAsiatica_SGA_v2.0_scaffold32G13166
StrigaAsiatica_trinity.Trinity
StrigaAsiatica_SGA_v2.0_scaffold58G20535
StrigaAsiatica_GER50367.1_25901
Euphrasiaarctica
Euphrasiaarctica_2
Phtheirospermum_Pjv1_00023235
Phtheirospermum_trinity.Trinity
Phtheirospermum_GFQ00981.1_22415
CastillejaMiniata_TRINITY_DN3606_c0_g1_i16
Pedicularis_2
OrobancheCumanamana_GWHTBHPV034383
LindenbergiaPhilippensis
LindenbergiaPhilippensis_02G167300.1
HandroanthusImpetiginosus_PIN24594.1
SesamumIndicum_SIN_1014772
CastillejaMiniata
Pedicularis
Ofasciculata_stem
PhelipancheAegyptiaca_GWHTBHPU017401
ConopholisAmericanaamericana_2010759
Epifagusvirginiana_2005220-buds
MimulusGuttatus_Migut.N01239.1.p
MimulusGuttatus_N1239.1
SesamumIndicum_SIN_1004558
OleaEuropaea_Oeu055570.1
IpomoeaNil_INIL02g17093.t1
CuscutaAustralis_C036N0089E0.1
SolanumTuberosum_PGSC0003DMT400001914
SolanumTuberosum_PGSC0003DMT400084285
CoffeaCanephora_Cc02_g25830
HelianthusAnnuus_OTF90986.1
HelianthusAnnuus_OTG25902.1
Daucuscarota_DCAR_022973
Actinidiaeriantha_DTZ79_07g01320
Actinidiaeriantha_DTZ79_10g03330
VacciniumCorymbosum_VaccDscaff14-augustus-gene-371.26-mRNA-1
VacciniumCorymbosum_VaccDscaff2-snap-gene-425.36-mRNA-1
GossypiumRaimondii_KJB79184
GossypiumRaimondii_KJB79185
GossypiumRaimondii_KJB79186
GossypiumRaimondii_KJB47495
TheobromaCacao_Thecc1EG006890t1
GossypiumRaimondii_KJB32505
ArabidopsisThaliana_AT1G16700.1
ArabidopsisThaliana_AT1G79010.1
CitrusSinensis_orange1.1g027264m
ManihotEsculenta_Manes.15G163400.1.p
ManihotEsculenta_Manes.17G101100.1.p
PopulusTrichocarpa_Potri.004G154900.1
PopulusTrichocarpa_Potri.009G116000.1
GlycineMax_Glyma.13G241500.1.p
GlycineMax_Glyma.15G071900.1.p
GlycineMax_Glyma.05G236100.1.p
VitisVinifera_GSVIVT01031966001
PrunusPersica_Prupe.8G096900.1.p
AquilegiaCoerulea_PIA59138.1
AquilegiaCoerulea_PIA59139.1
AquilegiaCoerulea_PIA59137.1
OryzaSativa_LOC_Os03g56300.1
SorghumBicolor_Sobic.001G073600.1
CinnamomumMicranthum_RWR95724.1
AmborellaTrichopoda_ERN17466.1
0.06
