# Supplementary material for: Altered Mitochondrial Respiration Is Associated With Loss of Nuclear‐Encoded OXPHOS Genes in Parasitic Broomrapes
Source: Ecol Evol. 2025 Jul 6;15(7):e71737. doi: 10.1002/ece3.71737 (PMC12230201; doi:10.1002/ece3.71737)
Supplement: Supplementary file 1 — Appendix S1. [file ECE3-15-e71737-s003.tgz › Orobanchaceae_O2K_Supplementary_v3/Data_S1/DataS1/CI/OG0010102.filter.fas.treefile.pdf]

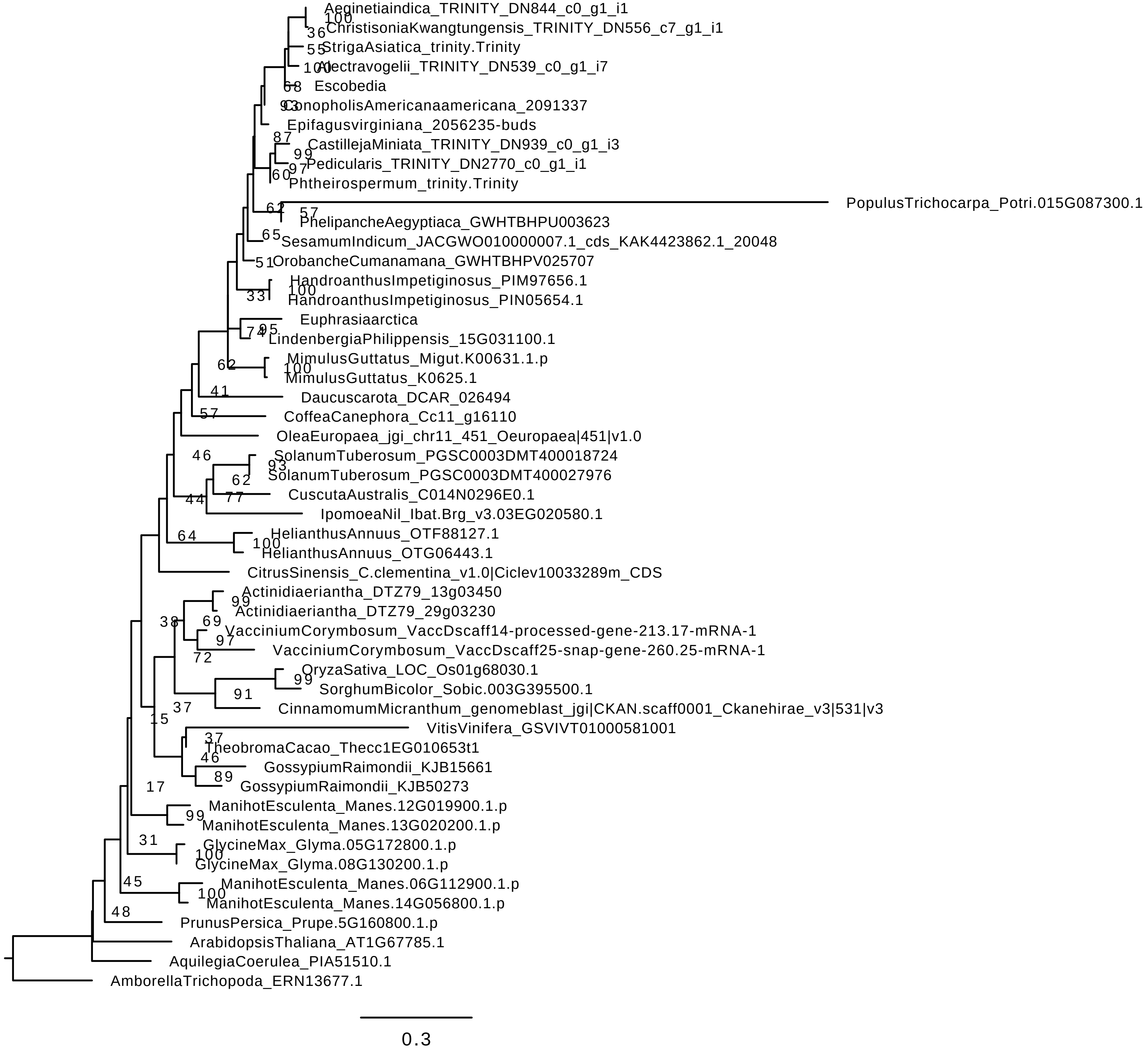
Aeginetiaindica_TRINITY_DN844_c0_g1_i1
ChristisoniaKwangtungensis_TRINITY_DN556_c7_g1_i1
StrigaAsiatica_trinity.Trinity
Alectravogelii_TRINITY_DN539_c0_g1_i7
Escobedia
ConopholisAmericanaamericana_2091337
Epifagusvirginiana_2056235-buds
CastillejaMiniata_TRINITY_DN939_c0_g1_i3
Pedicularis_TRINITY_DN2770_c0_g1_i1
Phtheirospermum_trinity.Trinity
PopulusTrichocarpa_Potri.015G087300.1
PhelipancheAegyptiaca_GWHTBHPU003623
SesamumIndicum_JACGWO010000007.1_cds_KAK4423862.1_20048
OrobancheCumanamana_GWHTBHPV025707
HandroanthusImpetiginosus_PIM97656.1
HandroanthusImpetiginosus_PIN05654.1
Euphrasiaarctica
LindenbergiaPhilippensis_15G031100.1
MimulusGuttatus_Migut.K00631.1.p
MimulusGuttatus_K0625.1
Daucuscarota_DCAR_026494
CoffeaCanephora_Cc11_g16110
OleaEuropaea_jgi_chr11_451_Oeuropaea|451|v1.0
SolanumTuberosum_PGSC0003DMT400018724
SolanumTuberosum_PGSC0003DMT400027976
CuscutaAustralis_C014N0296E0.1
IpomoeaNil_Ibat.Brg_v3.03EG020580.1
HelianthusAnnuus_OTF88127.1
HelianthusAnnuus_OTG06443.1
CitrusSinensis_C.clementina_v1.0|Ciclev10033289m_CDS
Actinidiaeriantha_DTZ79_13g03450
Actinidiaeriantha_DTZ79_29g03230
VacciniumCorymbosum_VaccDscaff14-processed-gene-213.17-mRNA-1
VacciniumCorymbosum_VaccDscaff25-snap-gene-260.25-mRNA-1
OryzaSativa_LOC_Os01g68030.1
SorghumBicolor_Sobic.003G395500.1
CinnamomumMicranthum_genomeblast_jgi|CKAN.scaff0001_Ckanehirae_v3|531|v3
VitisVinifera_GSVIVT01000581001
TheobromaCacao_Thecc1EG010653t1
GossypiumRaimondii_KJB15661
GossypiumRaimondii_KJB50273
ManihotEsculenta_Manes.12G019900.1.p
ManihotEsculenta_Manes.13G020200.1.p
GlycineMax_Glyma.05G172800.1.p
GlycineMax_Glyma.08G130200.1.p
ManihotEsculenta_Manes.06G112900.1.p
ManihotEsculenta_Manes.14G056800.1.p
PrunusPersica_Prupe.5G160800.1.p
ArabidopsisThaliana_AT1G67785.1
AquilegiaCoerulea_PIA51510.1
AmborellaTrichopoda_ERN13677.1
0.3
