# Supplementary material for: Altered Mitochondrial Respiration Is Associated With Loss of Nuclear‐Encoded OXPHOS Genes in Parasitic Broomrapes
Source: Ecol Evol. 2025 Jul 6;15(7):e71737. doi: 10.1002/ece3.71737 (PMC12230201; doi:10.1002/ece3.71737)
Supplement: Supplementary file 1 — Appendix S1. [file ECE3-15-e71737-s003.tgz › Orobanchaceae_O2K_Supplementary_v3/Data_S1/DataS1/CI/OG0008443.filter.fas.treefile.pdf]

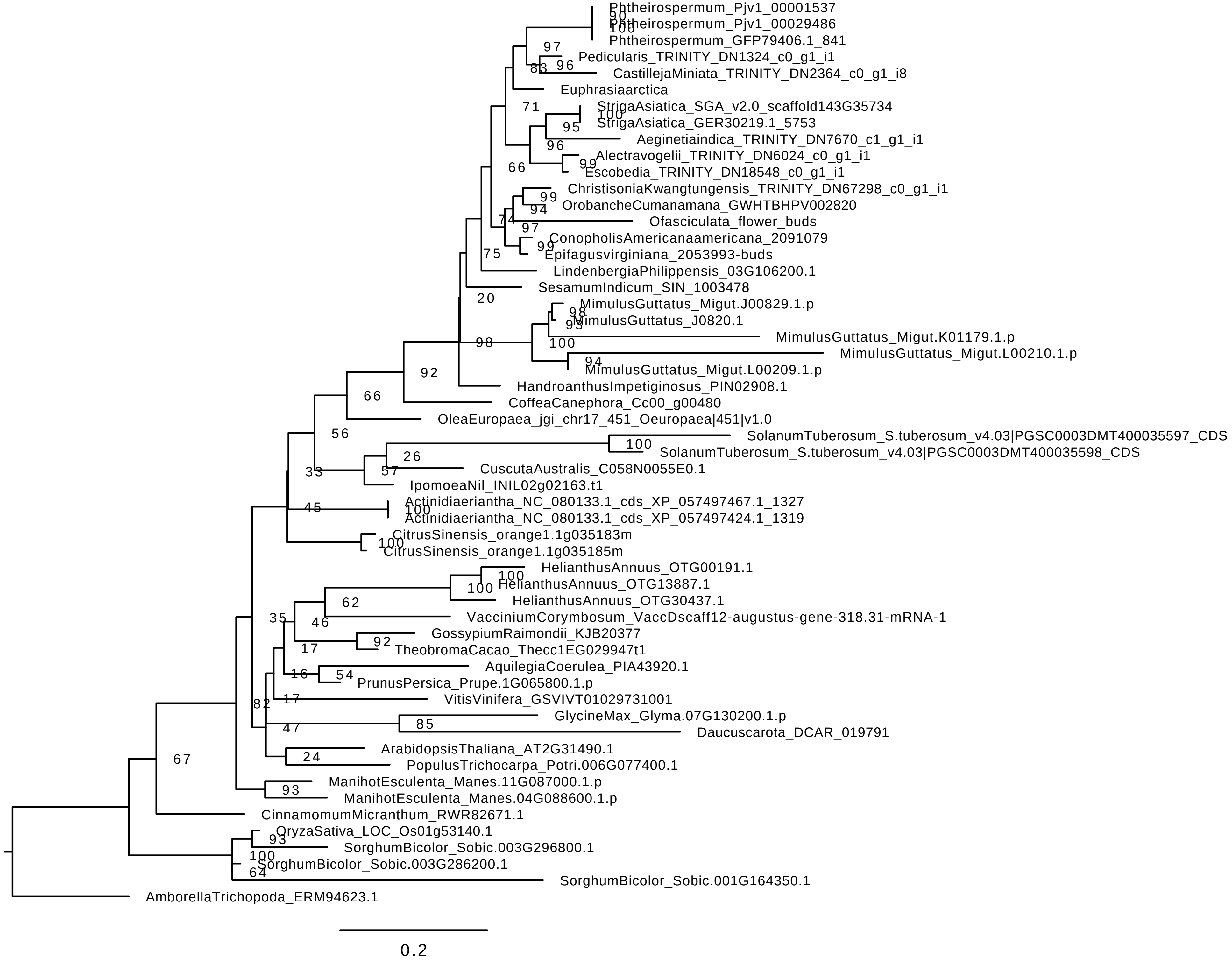

Phtheirospermum_Pjv1_00001537
Phtheirospermum_Pjv1_00029486
Phtheirospermum_GFP79406.1_841
Pedicularis_TRINITY_DN1324_c0_g1_i1
CastillejaMiniata_TRINITY_DN2364_c0_g1_i8
Euphrasiaarctica
StrigaAsiatica_SGA_v2.0_scaffold143G35734
StrigaAsiatica_GER30219.1_5753
Aeginetiaindica_TRINITY_DN7670_c1_g1_i1
Alectravogelii_TRINITY_DN6024_c0_g1_i1
Escobedia_TRINITY_DN18548_c0_g1_i1
ChristisoniaKwangtungensis_TRINITY_DN67298_c0_g1_i1
OrobancheCumanamana_GWHTBHPV002820
Ofasciculata_flower_buds
ConopholisAmericanaamericana_2091079
Epifagusvirginiana_2053993-buds
LindenbergiaPhilippensis_03G106200.1
SesamumIndicum_SIN_1003478
MimulusGuttatus_Migut.J00829.1.p
MimulusGuttatus_J0820.1
MimulusGuttatus_Migut.K01179.1.p
MimulusGuttatus_Migut.L00210.1.p
MimulusGuttatus_Migut.L00209.1.p
HandroanthusImpetiginosus_PIN02908.1
CoffeaCanephora_Cc00_g00480
OleaEuropaea_jgi_chr17_451_Oeuropaea|451|v1.0
SolanumTuberosum_S.tuberosum_v4.03|PGSC0003DMT400035597_CDS
SolanumTuberosum_S.tuberosum_v4.03|PGSC0003DMT400035598_CDS
CuscutaAustralis_C058N0055E0.1
IpomoeaNil_INIL02g02163.t1
Actinidiaeriantha_NC_080133.1_cds_XP_057497467.1_1327
Actinidiaeriantha_NC_080133.1_cds_XP_057497424.1_1319
CitrusSinensis_orange1.1g035183m
CitrusSinensis_orange1.1g035185m
HelianthusAnnuus_OTG00191.1
HelianthusAnnuus_OTG13887.1
HelianthusAnnuus_OTG30437.1
VacciniumCorymbosum_VaccDscaff12-augustus-gene-318.31-mRNA-1
GossypiumRaimondii_KJB20377
TheobromaCacao_Thecc1EG029947t1
AquilegiaCoerulea_PIA43920.1
PrunusPersica_Prupe.1G065800.1.p
VitisVinifera_GSVIVT01029731001
GlycineMax_Glyma.07G130200.1.p
Daucuscarota_DCAR_019791
ArabidopsisThaliana_AT2G31490.1
PopulusTrichocarpa_Potri.006G077400.1
ManihotEsculenta_Manes.11G087000.1.p
ManihotEsculenta_Manes.04G088600.1.p
CinnamomumMicranthum_RWR82671.1
OryzaSativa_LOC_Os01g53140.1
SorghumBicolor_Sobic.003G296800.1
SorghumBicolor_Sobic.003G286200.1
SorghumBicolor_Sobic.001G164350.1
AmborellaTrichopoda_ERM94623.1
0.2
