# Supplementary material for: Altered Mitochondrial Respiration Is Associated With Loss of Nuclear‐Encoded OXPHOS Genes in Parasitic Broomrapes
Source: Ecol Evol. 2025 Jul 6;15(7):e71737. doi: 10.1002/ece3.71737 (PMC12230201; doi:10.1002/ece3.71737)
Supplement: Supplementary file 1 — Appendix S1. [file ECE3-15-e71737-s003.tgz › Orobanchaceae_O2K_Supplementary_v3/Data_S1/DataS1/CI/OG0005513.filter.fas.treefile.pdf]

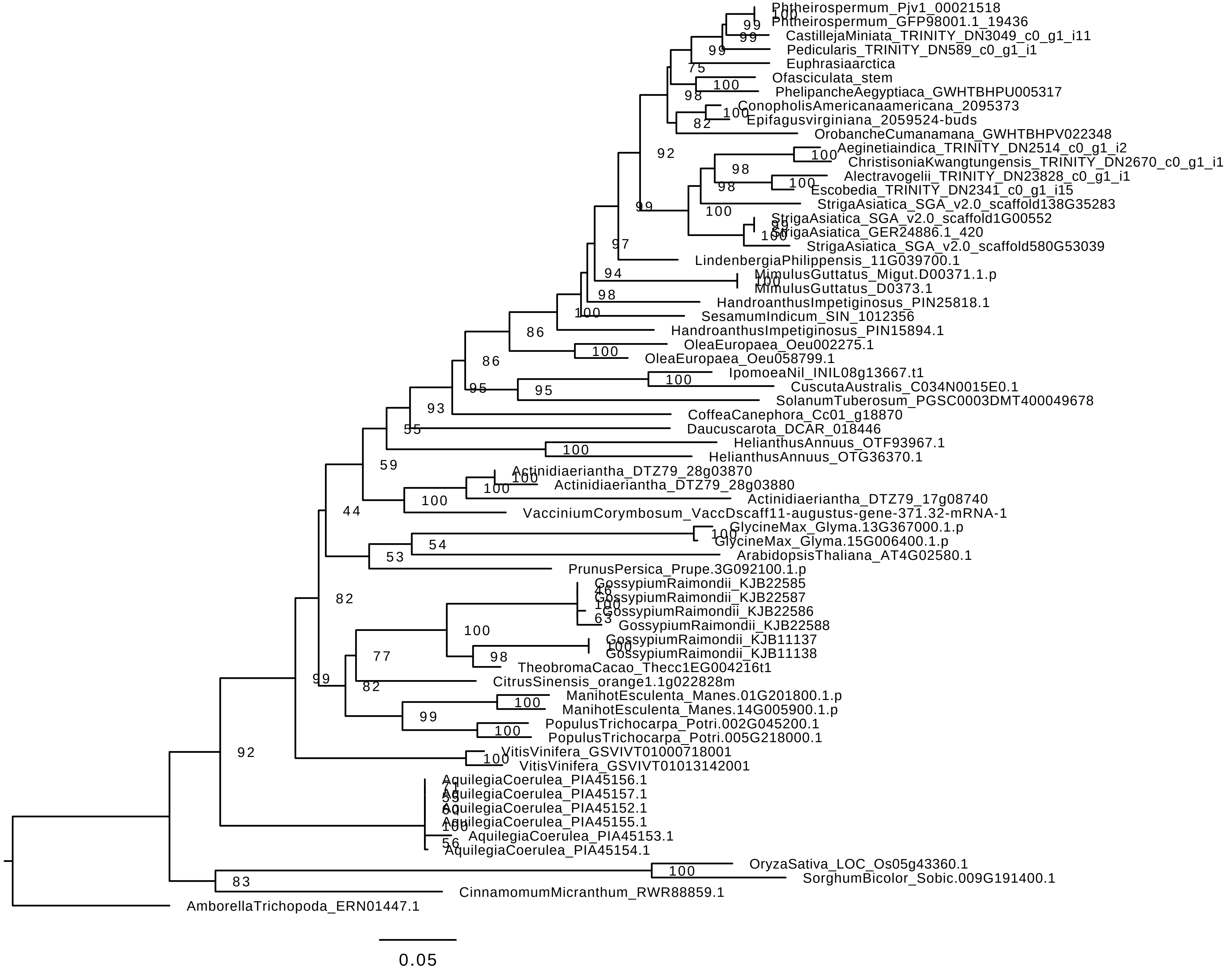

Phtheirospermum_Pjv1_00021518
Phtheirospermum_GFP98001.1_19436
CastillejaMiniata_TRINITY_DN3049_c0_g1_i11
Pedicularis_TRINITY_DN589_c0_g1_i1
Euphrasiaarctica
Ofasciculata_stem
PhelipancheAegyptiaca_GWHTBHPU005317
ConopholisAmericanaamericana_2095373
Epifagusvirginiana_2059524-buds
OrobancheCumanamana_GWHTBHPV022348
Aeginetiaindica_TRINITY_DN2514_c0_g1_i2
ChristisoniaKwangtungensis_TRINITY_DN2670_c0_g1_i1
Alectravogelii_TRINITY_DN23828_c0_g1_i1
Escobedia_TRINITY_DN2341_c0_g1_i15
StrigaAsiatica_SGA_v2.0_scaffold138G35283
StrigaAsiatica_SGA_v2.0_scaffold1G00552
StrigaAsiatica_GER24886.1_420
StrigaAsiatica_SGA_v2.0_scaffold580G53039
LindenbergiaPhilippensis_11G039700.1
MimulusGuttatus_Migut.D00371.1.p
MimulusGuttatus_D0373.1
HandroanthusImpetiginosus_PIN25818.1
SesamumIndicum_SIN_1012356
HandroanthusImpetiginosus_PIN15894.1
OleaEuropaea_Oeu002275.1
OleaEuropaea_Oeu058799.1
IpomoeaNil_INIL08g13667.t1
CuscutaAustralis_C034N0015E0.1
SolanumTuberosum_PGSC0003DMT400049678
CoffeaCanephora_Cc01_g18870
Daucuscarota_DCAR_018446
HelianthusAnnuus_OTF93967.1
HelianthusAnnuus_OTG36370.1
Actinidiaeriantha_DTZ79_28g03870
Actinidiaeriantha_DTZ79_28g03880
Actinidiaeriantha_DTZ79_17g08740
VacciniumCorymbosum_VaccDscaff11-augustus-gene-371.32-mRNA-1
GlycineMax_Glyma.13G367000.1.p
GlycineMax_Glyma.15G006400.1.p
ArabidopsisThaliana_AT4G02580.1
PrunusPersica_Prupe.3G092100.1.p
GossypiumRaimondii_KJB22585
GossypiumRaimondii_KJB22587
GossypiumRaimondii_KJB22586
GossypiumRaimondii_KJB22588
GossypiumRaimondii_KJB11137
GossypiumRaimondii_KJB11138
TheobromaCacao_Thecc1EG004216t1
CitrusSinensis_orange1.1g022828m
ManihotEsculenta_Manes.01G201800.1.p
ManihotEsculenta_Manes.14G005900.1.p
PopulusTrichocarpa_Potri.002G045200.1
PopulusTrichocarpa_Potri.005G218000.1
VitisVinifera_GSVIVT01000718001
VitisVinifera_GSVIVT01013142001
AquilegiaCoerulea_PIA45156.1
AquilegiaCoerulea_PIA45157.1
AquilegiaCoerulea_PIA45152.1
AquilegiaCoerulea_PIA45155.1
AquilegiaCoerulea_PIA45153.1
AquilegiaCoerulea_PIA45154.1
OryzaSativa_LOC_Os05g43360.1
SorghumBicolor_Sobic.009G191400.1
CinnamomumMicranthum_RWR88859.1
AmborellaTrichopoda_ERN01447.1
0.05
