# Supplementary material for: Altered Mitochondrial Respiration Is Associated With Loss of Nuclear‐Encoded OXPHOS Genes in Parasitic Broomrapes
Source: Ecol Evol. 2025 Jul 6;15(7):e71737. doi: 10.1002/ece3.71737 (PMC12230201; doi:10.1002/ece3.71737)
Supplement: Supplementary file 1 — Appendix S1. [file ECE3-15-e71737-s003.tgz › Orobanchaceae_O2K_Supplementary_v3/Data_S1/DataS1/CI/OG0007108.filter.fas.treefile.pdf]

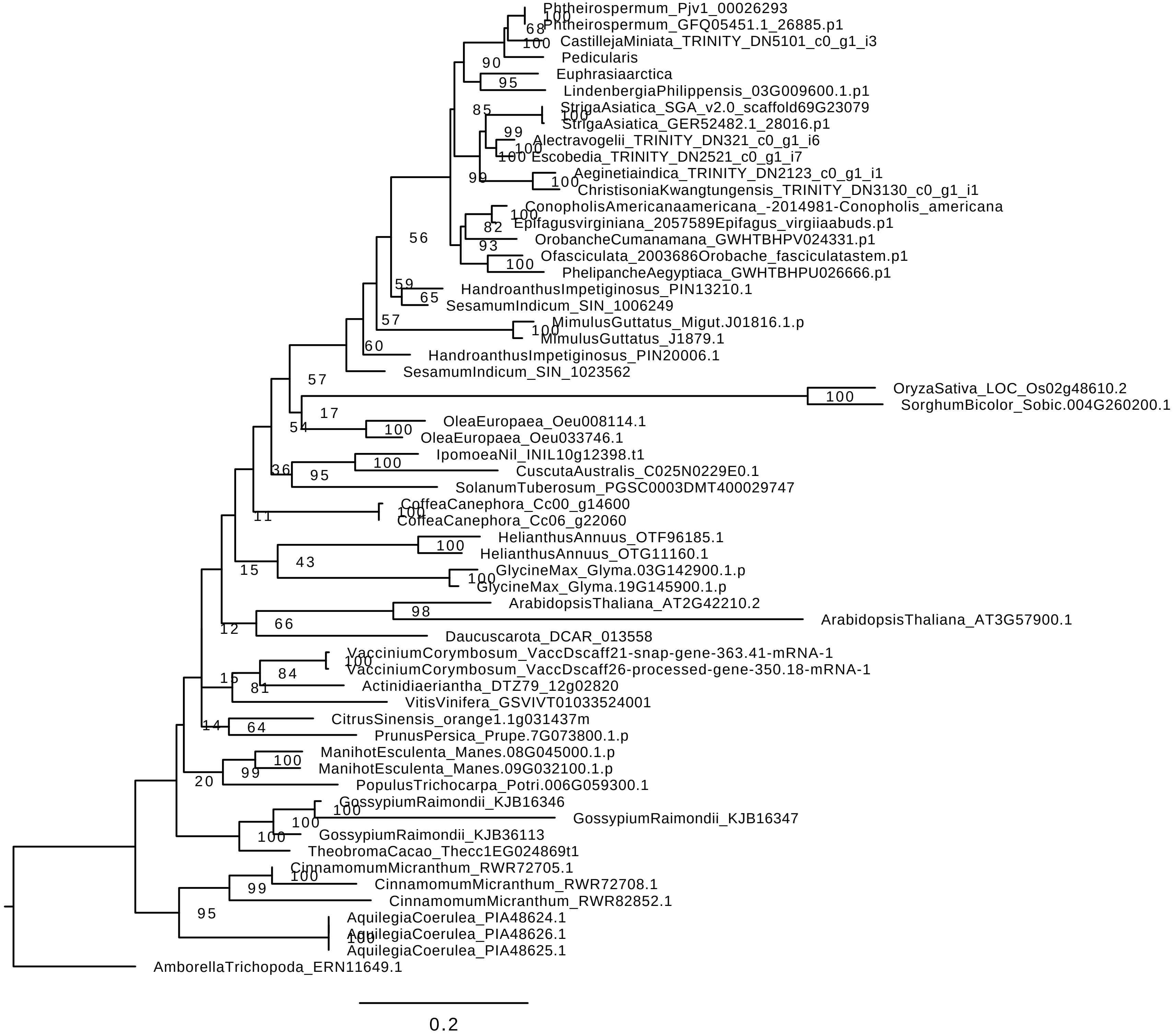
Phtheirospermum_Pjv1_00026293
Phtheirospermum_GFQ05451.1_26885.p1
CastillejaMiniata_TRINITY_DN5101_c0_g1_i3
Pedicularis
Euphrasiaarctica
LindenbergiaPhilippensis_03G009600.1.p1
StrigaAsiatica_SGA_v2.0_scaffold69G23079
StrigaAsiatica_GER52482.1_28016.p1
Alectravogelii_TRINITY_DN321_c0_g1_i6
Escobedia_TRINITY_DN2521_c0_g1_i7
Aeginetiaindica_TRINITY_DN2123_c0_g1_i1
ChristisoniaKwangtungensis_TRINITY_DN3130_c0_g1_i1
ConopholisAmericanaamericana_-2014981-Conopholis_americana
Epifagusvirginiana_2057589Epifagus_virgiiaabuds.p1
OrobancheCumanamana_GWHTBHPV024331.p1
Ofasciculata_2003686Orobache_fasciculatastem.p1
PhelipancheAegyptiaca_GWHTBHPU026666.p1
HandroanthusImpetiginosus_PIN13210.1
SesamumIndicum_SIN_1006249
MimulusGuttatus_Migut.J01816.1.p
MimulusGuttatus_J1879.1
HandroanthusImpetiginosus_PIN20006.1
SesamumIndicum_SIN_1023562
OryzaSativa_LOC_Os02g48610.2
SorghumBicolor_Sobic.004G260200.1
OleaEuropaea_Oeu008114.1
OleaEuropaea_Oeu033746.1
IpomoeaNil_INIL10g12398.t1
CuscutaAustralis_C025N0229E0.1
SolanumTuberosum_PGSC0003DMT400029747
CoffeaCanephora_Cc00_g14600
CoffeaCanephora_Cc06_g22060
HelianthusAnnuus_OTF96185.1
HelianthusAnnuus_OTG11160.1
GlycineMax_Glyma.03G142900.1.p
GlycineMax_Glyma.19G145900.1.p
ArabidopsisThaliana_AT2G42210.2
ArabidopsisThaliana_AT3G57900.1
Daucuscarota_DCAR_013558
VacciniumCorymbosum_VaccDscaff21-snap-gene-363.41-mRNA-1
VacciniumCorymbosum_VaccDscaff26-processed-gene-350.18-mRNA-1
Actinidiaeriantha_DTZ79_12g02820
VitisVinifera_GSVIVT01033524001
CitrusSinensis_orange1.1g031437m
PrunusPersica_Prupe.7G073800.1.p
ManihotEsculenta_Manes.08G045000.1.p
ManihotEsculenta_Manes.09G032100.1.p
PopulusTrichocarpa_Potri.006G059300.1
GossypiumRaimondii_KJB16346
GossypiumRaimondii_KJB16347
GossypiumRaimondii_KJB36113
TheobromaCacao_Thecc1EG024869t1
CinnamomumMicranthum_RWR72705.1
CinnamomumMicranthum_RWR72708.1
CinnamomumMicranthum_RWR82852.1
AquilegiaCoerulea_PIA48624.1
AquilegiaCoerulea_PIA48626.1
AquilegiaCoerulea_PIA48625.1
AmborellaTrichopoda_ERN11649.1
0.2
