# Supplementary material for: Altered Mitochondrial Respiration Is Associated With Loss of Nuclear‐Encoded OXPHOS Genes in Parasitic Broomrapes
Source: Ecol Evol. 2025 Jul 6;15(7):e71737. doi: 10.1002/ece3.71737 (PMC12230201; doi:10.1002/ece3.71737)
Supplement: Supplementary file 1 — Appendix S1. [file ECE3-15-e71737-s003.tgz › Orobanchaceae_O2K_Supplementary_v3/Data_S1/DataS1/CI/OG0004769.filter.fas.treefile.pdf]

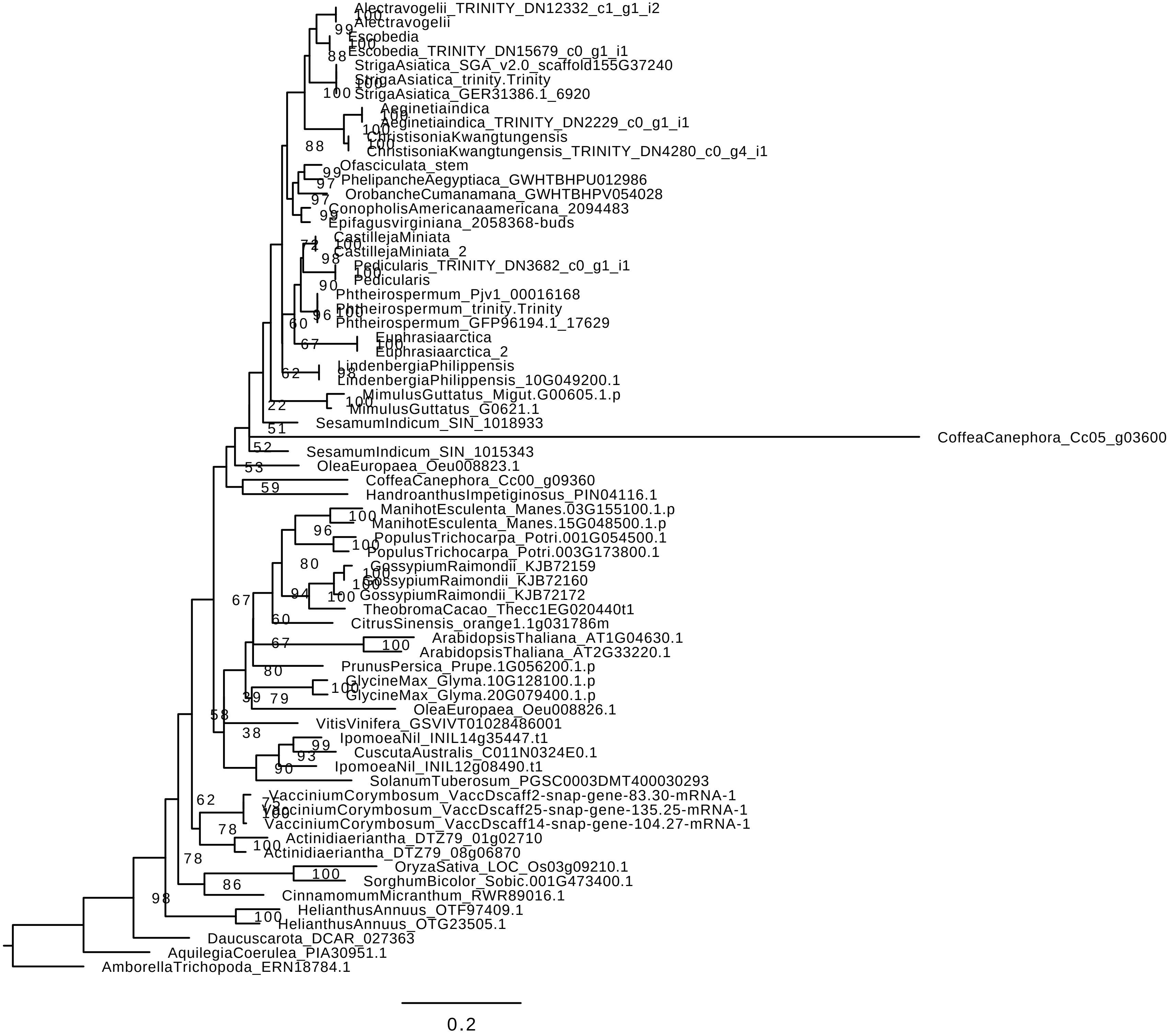

Alectravogelii_TRINITY_DN12332_c1_g1_i2
Alectravogelii
Escobedia
Escobedia_TRINITY_DN15679_c0_g1_i1
StrigaAsiatica_SGA_v2.0_scaffold155G37240
StrigaAsiatica_trinity.Trinity
StrigaAsiatica_GER31386.1_6920
Aeginetiaindica
Aeginetiaindica_TRINITY_DN2229_c0_g1_i1
ChristisoniaKwangtungensis
ChristisoniaKwangtungensis_TRINITY_DN4280_c0_g4_i1
Ofasciculata_stem
PhelipancheAegyptiaca_GWHTBHPU012986
OrobancheCumanamana_GWHTBHPV054028
ConopholisAmericanaamericana_2094483
Epifagusvirginiana_2058368-buds
CastillejaMiniata
CastillejaMiniata_2
Pedicularis_TRINITY_DN3682_c0_g1_i1
Pedicularis
Phtheirospermum_Pjv1_00016168
Phtheirospermum_trinity.Trinity
Phtheirospermum_GFP96194.1_17629
Euphrasiaarctica
Euphrasiaarctica_2
LindenbergiaPhilippensis
LindenbergiaPhilippensis_10G049200.1
MimulusGuttatus_Migut.G00605.1.p
MimulusGuttatus_G0621.1
SesamumIndicum_SIN_1018933
CoffeaCanephora_Cc05_g03600
SesamumIndicum_SIN_1015343
OleaEuropaea_Oeu008823.1
CoffeaCanephora_Cc00_g09360
HandroanthusImpetiginosus_PIN04116.1
ManihotEsculenta_Manes.03G155100.1.p
ManihotEsculenta_Manes.15G048500.1.p
PopulusTrichocarpa_Potri.001G054500.1
PopulusTrichocarpa_Potri.003G173800.1
GossypiumRaimondii_KJB72159
GossypiumRaimondii_KJB72160
GossypiumRaimondii_KJB72172
TheobromaCacao_Thecc1EG020440t1
CitrusSinensis_orange1.1g031786m
ArabidopsisThaliana_AT1G04630.1
ArabidopsisThaliana_AT2G33220.1
PrunusPersica_Prupe.1G056200.1.p
GlycineMax_Glyma.10G128100.1.p
GlycineMax_Glyma.20G079400.1.p
OleaEuropaea_Oeu008826.1
VitisVinifera_GSVIVT01028486001
IpomoeaNil_INIL14g35447.t1
CuscutaAustralis_C011N0324E0.1
IpomoeaNil_INIL12g08490.t1
SolanumTuberosum_PGSC0003DMT400030293
VacciniumCorymbosum_VaccDscaff2-snap-gene-83.30-mRNA-1
VacciniumCorymbosum_VaccDscaff25-snap-gene-135.25-mRNA-1
VacciniumCorymbosum_VaccDscaff14-snap-gene-104.27-mRNA-1
Actinidiaeriantha_DTZ79_01g02710
Actinidiaeriantha_DTZ79_08g06870
OryzaSativa_LOC_Os03g09210.1
SorghumBicolor_Sobic.001G473400.1
CinnamomumMicranthum_RWR89016.1
HelianthusAnnuus_OTF97409.1
HelianthusAnnuus_OTG23505.1
Daucuscarota_DCAR_027363
AquilegiaCoerulea_PIA30951.1
AmborellaTrichopoda_ERN18784.1
0.2
