# Supplementary material for: Altered Mitochondrial Respiration Is Associated With Loss of Nuclear‐Encoded OXPHOS Genes in Parasitic Broomrapes
Source: Ecol Evol. 2025 Jul 6;15(7):e71737. doi: 10.1002/ece3.71737 (PMC12230201; doi:10.1002/ece3.71737)
Supplement: Supplementary file 1 — Appendix S1. [file ECE3-15-e71737-s003.tgz › Orobanchaceae_O2K_Supplementary_v3/Data_S1/DataS1/CI/OG0003138.filter.fas.treefile.pdf]

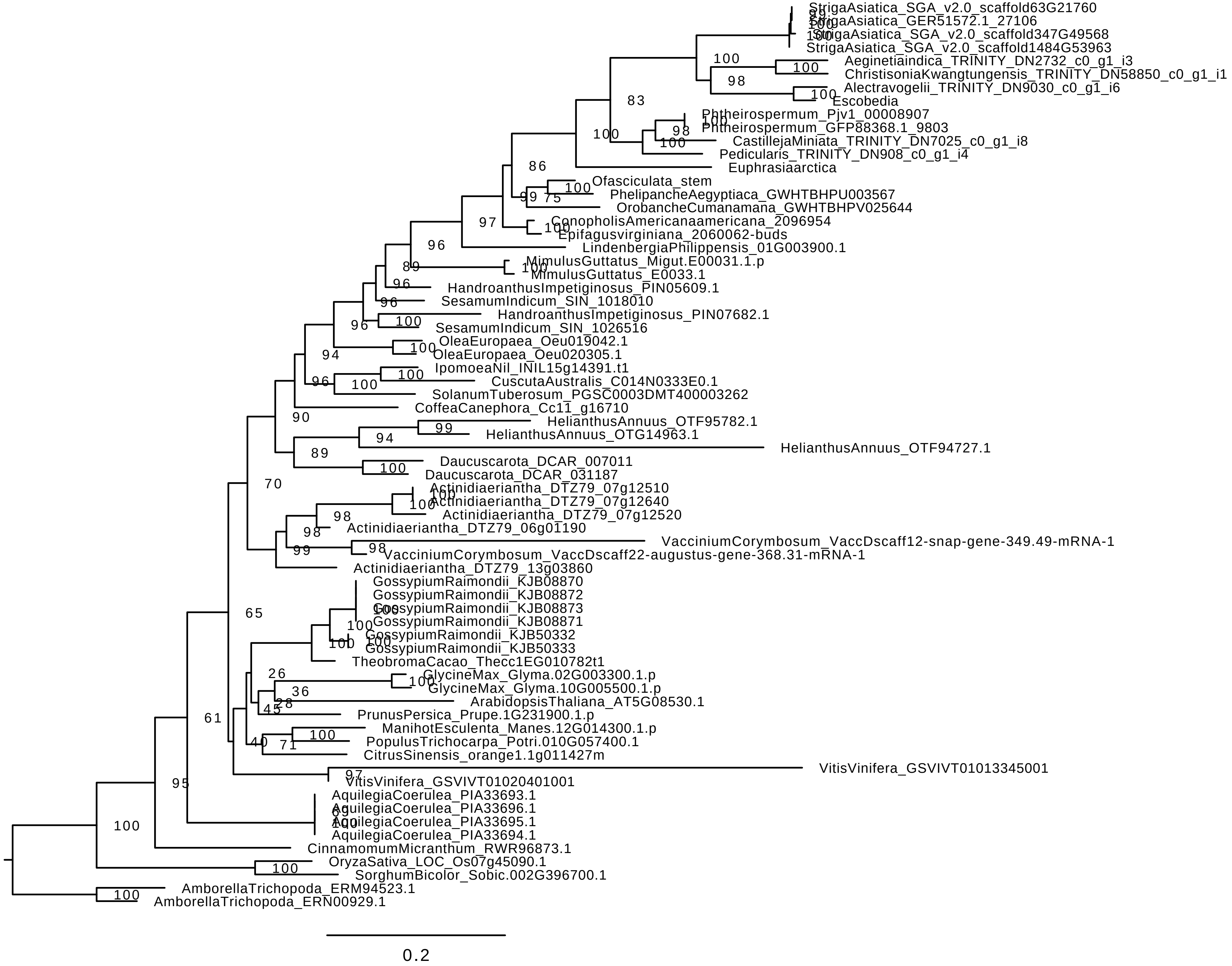
StrigaAsiatica_SGA_v2.0_scaffold63G21760
StrigaAsiatica_GER51572.1_27106
StrigaAsiatica_SGA_v2.0_scaffold347G49568
StrigaAsiatica_SGA_v2.0_scaffold1484G53963
Aeginetiaindica_TRINITY_DN2732_c0_g1_i3
ChristisoniaKwangtungensis_TRINITY_DN58850_c0_g1_i1
Alectravogelii_TRINITY_DN9030_c0_g1_i6
Escobedia
Phtheirospermum_Pjv1_00008907
Phtheirospermum_GFP88368.1_9803
CastillejaMiniata_TRINITY_DN7025_c0_g1_i8
Pedicularis_TRINITY_DN908_c0_g1_i4
Euphrasiaarctica
Ofasciculata_stem
PhelipancheAegyptiaca_GWHTBHPU003567
OrobancheCumanamana_GWHTBHPV025644
ConopholisAmericanaamericana_2096954
Epifagusvirginiana_2060062-buds
LindenbergiaPhilippensis_01G003900.1
MimulusGuttatus_Migut.E00031.1.p
MimulusGuttatus_E0033.1
HandroanthusImpetiginosus_PIN05609.1
SesamumIndicum_SIN_1018010
HandroanthusImpetiginosus_PIN07682.1
SesamumIndicum_SIN_1026516
OleaEuropaea_Oeu019042.1
OleaEuropaea_Oeu020305.1
IpomoeaNil_INIL15g14391.t1
CuscutaAustralis_C014N0333E0.1
SolanumTuberosum_PGSC0003DMT400003262
CoffeaCanephora_Cc11_g16710
HelianthusAnnuus_OTF95782.1
HelianthusAnnuus_OTG14963.1
HelianthusAnnuus_OTF94727.1
Daucuscarota_DCAR_007011
Daucuscarota_DCAR_031187
Actinidiaeriantha_DTZ79_07g12510
Actinidiaeriantha_DTZ79_07g12640
Actinidiaeriantha_DTZ79_07g12520
Actinidiaeriantha_DTZ79_06g01190
VacciniumCorymbosum_VaccDscaff12-snap-gene-349.49-mRNA-1
VacciniumCorymbosum_VaccDscaff22-augustus-gene-368.31-mRNA-1
Actinidiaeriantha_DTZ79_13g03860
GossypiumRaimondii_KJB08870
GossypiumRaimondii_KJB08872
GossypiumRaimondii_KJB08873
GossypiumRaimondii_KJB08871
GossypiumRaimondii_KJB50332
GossypiumRaimondii_KJB50333
TheobromaCacao_Thecc1EG010782t1
GlycineMax_Glyma.02G003300.1.p
GlycineMax_Glyma.10G005500.1.p
ArabidopsisThaliana_AT5G08530.1
PrunusPersica_Prupe.1G231900.1.p
ManihotEsculenta_Manes.12G014300.1.p
PopulusTrichocarpa_Potri.010G057400.1
CitrusSinensis_orange1.1g011427m
VitisVinifera_GSVIVT01013345001
VitisVinifera_GSVIVT01020401001
AquilegiaCoerulea_PIA33693.1
AquilegiaCoerulea_PIA33696.1
AquilegiaCoerulea_PIA33695.1
AquilegiaCoerulea_PIA33694.1
CinnamomumMicranthum_RWR96873.1
OryzaSativa_LOC_Os07g45090.1
SorghumBicolor_Sobic.002G396700.1
AmborellaTrichopoda_ERM94523.1
AmborellaTrichopoda_ERN00929.1
0.2
