# Supplementary material for: Altered Mitochondrial Respiration Is Associated With Loss of Nuclear‐Encoded OXPHOS Genes in Parasitic Broomrapes
Source: Ecol Evol. 2025 Jul 6;15(7):e71737. doi: 10.1002/ece3.71737 (PMC12230201; doi:10.1002/ece3.71737)
Supplement: Supplementary file 1 — Appendix S1. [file ECE3-15-e71737-s003.tgz › Orobanchaceae_O2K_Supplementary_v3/Data_S1/DataS1/CI/OG0005865.filter.fas.treefile.pdf]

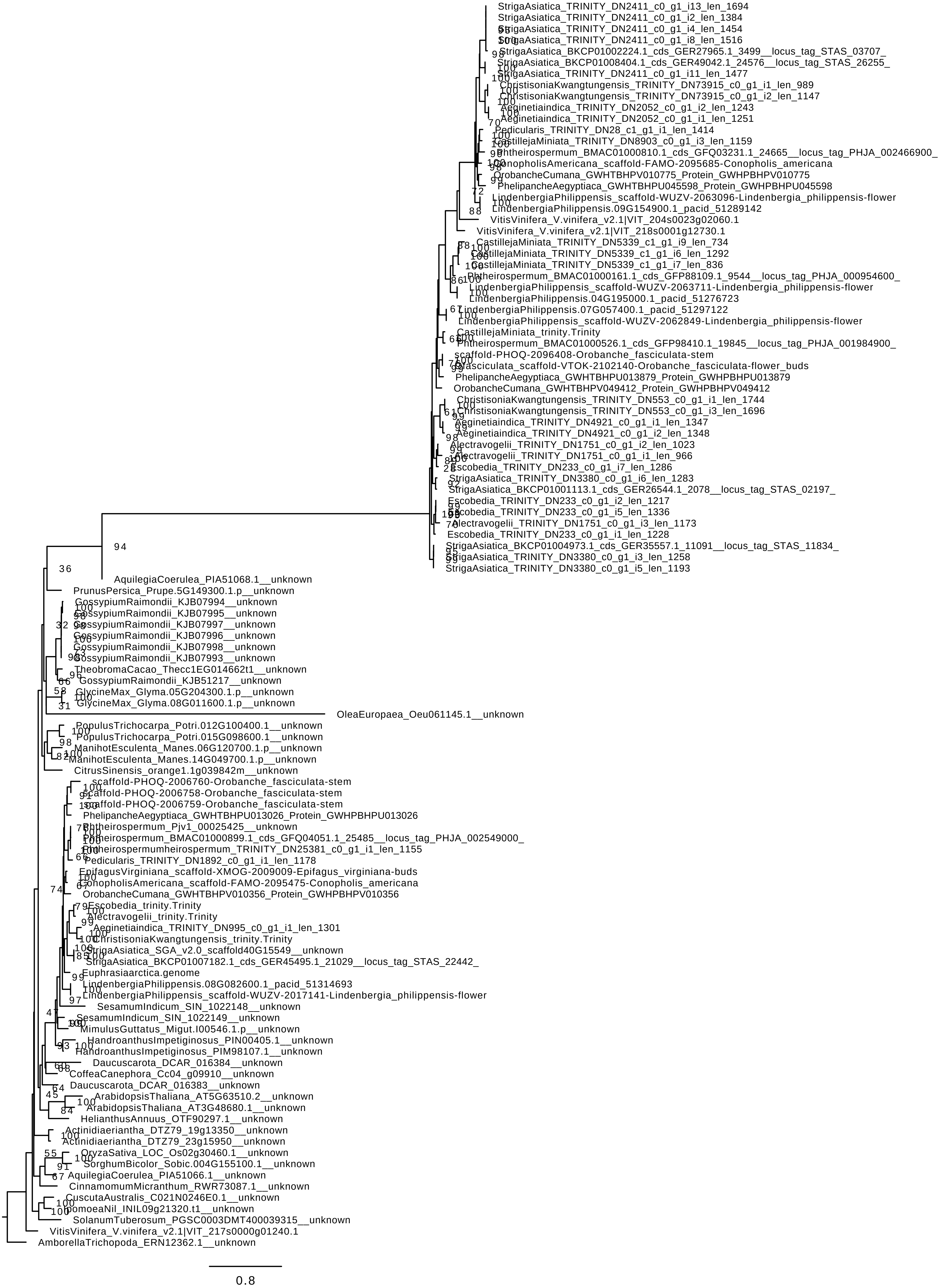
StrigaAsiatica_TRINITY_DN2411_c0_g1_i13_len_1694
StrigaAsiatica_TRINITY_DN2411_c0_g1_i2_len_1384
StrigaAsiatica_TRINITY_DN2411_c0_g1_i4_len_1454
StrigaAsiatica_TRINITY_DN2411_c0_g1_i8_len_1516
StrigaAsiatica_BKCP01002224.1_cds_GER27965.1_3499__locus_tag_STAS_03707_
StrigaAsiatica_BKCP01008404.1_cds_GER49042.1_24576__locus_tag_STAS_26255_
StrigaAsiatica_TRINITY_DN2411_c0_g1_i11_len_1477
ChristisoniaKwangtungensis_TRINITY_DN73915_c0_g1_i1_len_989
ChristisoniaKwangtungensis_TRINITY_DN73915_c0_g1_i2_len_1147
Aeginetiaindica_TRINITY_DN2052_c0_g1_i2_len_1243
Aeginetiaindica_TRINITY_DN2052_c0_g1_i1_len_1251
Pedicularis_TRINITY_DN28_c1_g1_i1_len_1414
CastillejaMiniata_TRINITY_DN8903_c0_g1_i3_len_1159
Phtheirospermum_BMAC01000810.1_cds_GFQ03231.1_24665__locus_tag_PHJA_002466900_
ConopholisAmericana_scaffold-FAMO-2095685-Conopholis_americana
OrobancheCumana_GWHTBHPV010775_Protein_GWHPBHPV010775
PhelipancheAegyptiaca_GWHTBHPU045598_Protein_GWHPBHPU045598
LindenbergiaPhilippensis_scaffold-WUZV-2063096-Lindenbergia_philippensis-flower
LindenbergiaPhilippensis.09G154900.1_pacid_51289142
VitisVinifera_V.vinifera_v2.1|VIT_204s0023g02060.1
VitisVinifera_V.vinifera_v2.1|VIT_218s0001g12730.1
CastillejaMiniata_TRINITY_DN5339_c1_g1_i9_len_734
CastillejaMiniata_TRINITY_DN5339_c1_g1_i6_len_1292
CastillejaMiniata_TRINITY_DN5339_c1_g1_i7_len_836
Phtheirospermum_BMAC01000161.1_cds_GFP88109.1_9544__locus_tag_PHJA_000954600_
LindenbergiaPhilippensis_scaffold-WUZV-2063711-Lindenbergia_philippensis-flower
LindenbergiaPhilippensis.04G195000.1_pacid_51276723
LindenbergiaPhilippensis.07G057400.1_pacid_51297122
LindenbergiaPhilippensis_scaffold-WUZV-2062849-Lindenbergia_philippensis-flower
CastillejaMiniata_trinity.Trinity
Phtheirospermum_BMAC01000526.1_cds_GFP98410.1_19845__locus_tag_PHJA_001984900_
scaffold-PHOQ-2096408-Orobanche_fasciculata-stem
Ofasciculata_scaffold-VTOK-2102140-Orobanche_fasciculata-flower_buds
PhelipancheAegyptiaca_GWHTBHPU013879_Protein_GWHPBHPU013879
OrobancheCumana_GWHTBHPV049412_Protein_GWHPBHPV049412
ChristisoniaKwangtungensis_TRINITY_DN553_c0_g1_i1_len_1744
ChristisoniaKwangtungensis_TRINITY_DN553_c0_g1_i3_len_1696
Aeginetiaindica_TRINITY_DN4921_c0_g1_i1_len_1347
Aeginetiaindica_TRINITY_DN4921_c0_g1_i2_len_1348
Alectravogelii_TRINITY_DN1751_c0_g1_i2_len_1023
Alectravogelii_TRINITY_DN1751_c0_g1_i1_len_966
Escobedia_TRINITY_DN233_c0_g1_i7_len_1286
StrigaAsiatica_TRINITY_DN3380_c0_g1_i6_len_1283
StrigaAsiatica_BKCP01001113.1_cds_GER26544.1_2078__locus_tag_STAS_02197_
Escobedia_TRINITY_DN233_c0_g1_i2_len_1217
Escobedia_TRINITY_DN233_c0_g1_i5_len_1336
Alectravogelii_TRINITY_DN1751_c0_g1_i3_len_1173
Escobedia_TRINITY_DN233_c0_g1_i1_len_1228
StrigaAsiatica_BKCP01004973.1_cds_GER35557.1_11091__locus_tag_STAS_11834_
StrigaAsiatica_TRINITY_DN3380_c0_g1_i3_len_1258
StrigaAsiatica_TRINITY_DN3380_c0_g1_i5_len_1193
94
36
AquilegiaCoerulea_PIA51068.1__unknown
PrunusPersica_Prupe.5G149300.1.p__unknown
GossypiumRaimondii_KJB07994__unknown
GossypiumRaimondii_KJB07995__unknown
GossypiumRaimondii_KJB07997__unknown
GossypiumRaimondii_KJB07996__unknown
GossypiumRaimondii_KJB07998__unknown
GossypiumRaimondii_KJB07993__unknown
TheobromaCacao_Thecc1EG014662t1__unknown
GossypiumRaimondii_KJB51217__unknown
GlycineMax_Glyma.05G204300.1.p__unknown
GlycineMax_Glyma.08G011600.1.p__unknown
OleaEuropaea_Oeu061145.1__unknown
PopulusTrichocarpa_Potri.012G100400.1__unknown
PopulusTrichocarpa_Potri.015G098600.1__unknown
ManihotEsculenta_Manes.06G120700.1.p__unknown
ManihotEsculenta_Manes.14G049700.1.p__unknown
CitrusSinensis_orange1.1g039842m__unknown
scaffold-PHOQ-2006760-Orobanche_fasciculata-stem
scaffold-PHOQ-2006758-Orobanche_fasciculata-stem
scaffold-PHOQ-2006759-Orobanche_fasciculata-stem
PhelipancheAegyptiaca_GWHTBHPU013026_Protein_GWHPBHPU013026
Phtheirospermum_Pjv1_00025425__unknown
Phtheirospermum_BMAC01000899.1_cds_GFQ04051.1_25485__locus_tag_PHJA_002549000_
Phtheirospermumheirospermum_TRINITY_DN25381_c0_g1_i1_len_1155
Pedicularis_TRINITY_DN1892_c0_g1_i1_len_1178
EpifagusVirginiana_scaffold-XMOG-2009009-Epifagus_virginiana-buds
ConopholisAmericana_scaffold-FAMO-2095475-Conopholis_americana
OrobancheCumana_GWHTBHPV010356_Protein_GWHPBHPV010356
Escobedia_trinity.Trinity
Alectravogelii_trinity.Trinity
Aeginetiaindica_TRINITY_DN995_c0_g1_i1_len_1301
ChristisoniaKwangtungensis_trinity.Trinity
StrigaAsiatica_SGA_v2.0_scaffold40G15549__unknown
StrigaAsiatica_BKCP01007182.1_cds_GER45495.1_21029__locus_tag_STAS_22442_
Euphrasiaarctica.genome
LindenbergiaPhilippensis.08G082600.1_pacid_51314693
LindenbergiaPhilippensis_scaffold-WUZV-2017141-Lindenbergia_philippensis-flower
SesamumIndicum_SIN_1022148__unknown
SesamumIndicum_SIN_1022149__unknown
MimulusGuttatus_Migut.I00546.1.p__unknown
HandroanthusImpetiginosus_PIN00405.1__unknown
HandroanthusImpetiginosus_PIM98107.1__unknown
Daucuscarota_DCAR_016384__unknown
CoffeaCanephora_Cc04_g09910__unknown
Daucuscarota_DCAR_016383__unknown
ArabidopsisThaliana_AT5G63510.2__unknown
ArabidopsisThaliana_AT3G48680.1__unknown
HelianthusAnnuus_OTF90297.1__unknown
Actinidiaeriantha_DTZ79_19g13350__unknown
Actinidiaeriantha_DTZ79_23g15950__unknown
OryzaSativa_LOC_Os02g30460.1__unknown
SorghumBicolor_Sobic.004G155100.1__unknown
AquilegiaCoerulea_PIA51066.1__unknown
CinnamomumMicranthum_RWR73087.1__unknown
CuscutaAustralis_C021N0246E0.1__unknown
IpomoeaNil_INIL09g21320.t1__unknown
SolanumTuberosum_PGSC0003DMT400039315__unknown
VitisVinifera_V.vinifera_v2.1|VIT_217s0000g01240.1
AmborellaTrichopoda_ERN12362.1__unknown
0.8
