# Supplementary material for: Altered Mitochondrial Respiration Is Associated With Loss of Nuclear‐Encoded OXPHOS Genes in Parasitic Broomrapes
Source: Ecol Evol. 2025 Jul 6;15(7):e71737. doi: 10.1002/ece3.71737 (PMC12230201; doi:10.1002/ece3.71737)
Supplement: Supplementary file 1 — Appendix S1. [file ECE3-15-e71737-s003.tgz › Orobanchaceae_O2K_Supplementary_v3/Data_S1/DataS1/CI/OG0005402.filter.fas.treefile.pdf]

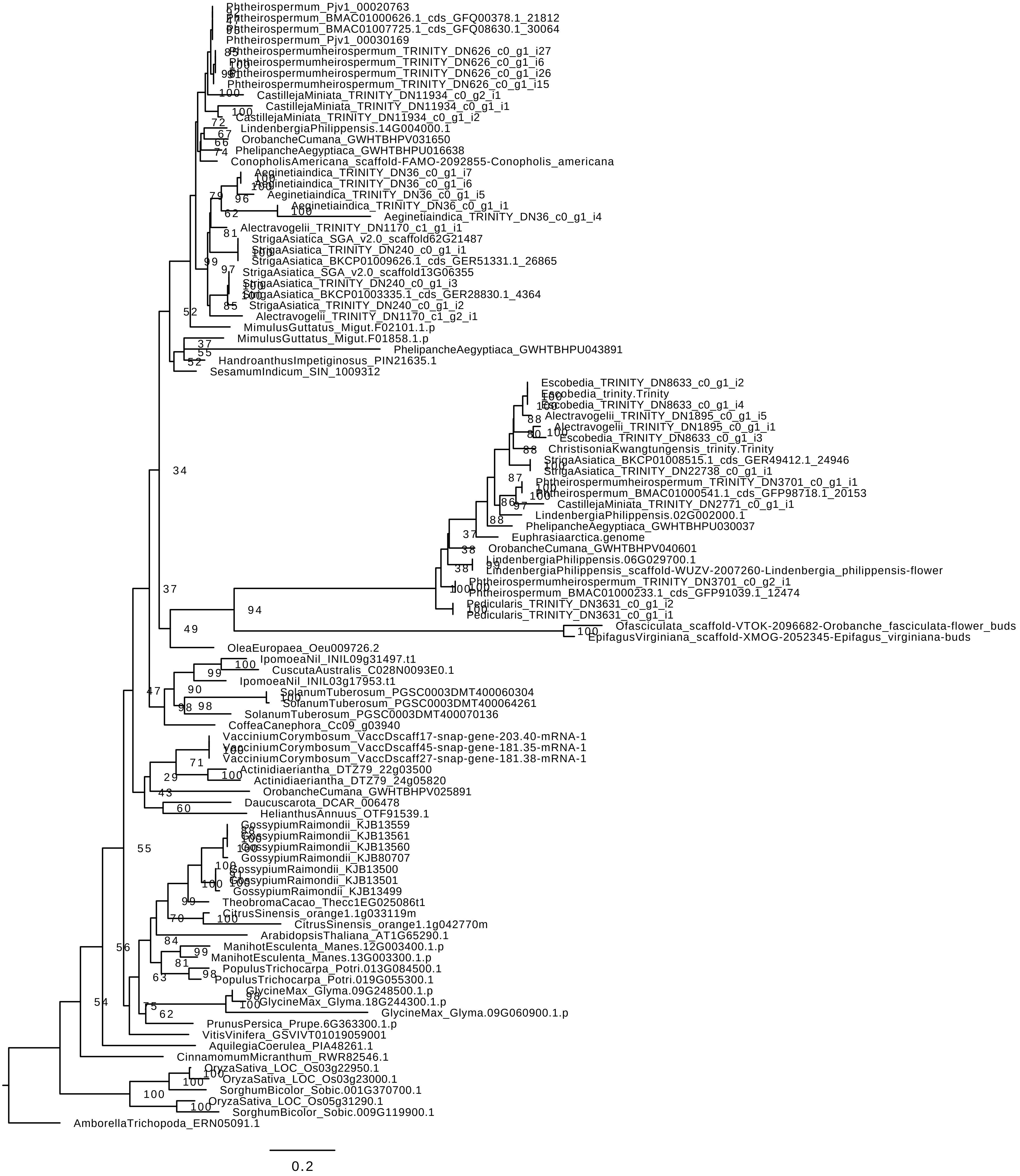

Phtheirospermum_Pjv1_00020763
Phtheirospermum_BMAC01000626.1_cds_GFQ00378.1_21812
Phtheirospermum_BMAC01007725.1_cds_GFQ08630.1_30064
Phtheirospermum_Pjv1_00030169
Phtheirospermumheirospermum_TRINITY_DN626_c0_g1_i27
Phtheirospermumheirospermum_TRINITY_DN626_c0_g1_i6
Phtheirospermumheirospermum_TRINITY_DN626_c0_g1_i26
Phtheirospermumheirospermum_TRINITY_DN626_c0_g1_i15
CastillejaMiniata_TRINITY_DN11934_c0_g2_i1
CastillejaMiniata_TRINITY_DN11934_c0_g1_i1
CastillejaMiniata_TRINITY_DN11934_c0_g1_i2
LindenbergiaPhilippensis.14G004000.1
OrobancheCumana_GWHTBHPV031650
PhelipancheAegyptiaca_GWHTBHPU016638
ConopholisAmericana_scaffold-FAMO-2092855-Conopholis_americana
Aeginetiaindica_TRINITY_DN36_c0_g1_i7
Aeginetiaindica_TRINITY_DN36_c0_g1_i6
Aeginetiaindica_TRINITY_DN36_c0_g1_i5
Aeginetiaindica_TRINITY_DN36_c0_g1_i1
Aeginetiaindica_TRINITY_DN36_c0_g1_i4
Alectravogelii_TRINITY_DN1170_c1_g1_i1
StrigaAsiatica_SGA_v2.0_scaffold62G21487
StrigaAsiatica_TRINITY_DN240_c0_g1_i1
StrigaAsiatica_BKCP01009626.1_cds_GER51331.1_26865
StrigaAsiatica_SGA_v2.0_scaffold13G06355
StrigaAsiatica_TRINITY_DN240_c0_g1_i3
StrigaAsiatica_BKCP01003335.1_cds_GER28830.1_4364
StrigaAsiatica_TRINITY_DN240_c0_g1_i2
Alectravogelii_TRINITY_DN1170_c1_g2_i1
MimulusGuttatus_Migut.F02101.1.p
MimulusGuttatus_Migut.F01858.1.p
PhelipancheAegyptiaca_GWHTBHPU043891
HandroanthusImpetiginosus_PIN21635.1
SesamumIndicum_SIN_1009312
Escobedia_TRINITY_DN8633_c0_g1_i2
Escobedia_trinity.Trinity
Escobedia_TRINITY_DN8633_c0_g1_i4
Alectravogelii_TRINITY_DN1895_c0_g1_i5
Alectravogelii_TRINITY_DN1895_c0_g1_i1
Escobedia_TRINITY_DN8633_c0_g1_i3
ChristisoniaKwangtungensis_trinity.Trinity
StrigaAsiatica_BKCP01008515.1_cds_GER49412.1_24946
StrigaAsiatica_TRINITY_DN22738_c0_g1_i1
Phtheirospermumheirospermum_TRINITY_DN3701_c0_g1_i1
Phtheirospermum_BMAC01000541.1_cds_GFP98718.1_20153
CastillejaMiniata_TRINITY_DN2771_c0_g1_i1
LindenbergiaPhilippensis.02G002000.1
PhelipancheAegyptiaca_GWHTBHPU030037
Euphrasiaarctica.genome
OrobancheCumana_GWHTBHPV040601
LindenbergiaPhilippensis.06G029700.1
LindenbergiaPhilippensis_scaffold-WUZV-2007260-Lindenbergia_philippensis-flower
Phtheirospermumheirospermum_TRINITY_DN3701_c0_g2_i1
Phtheirospermum_BMAC01000233.1_cds_GFP91039.1_12474
Pedicularis_TRINITY_DN3631_c0_g1_i2
Pedicularis_TRINITY_DN3631_c0_g1_i1
Ofasciculata_scaffold-VTOK-2096682-Orobanche_fasciculata-flower_buds
EpifagusVirginiana_scaffold-XMOG-2052345-Epifagus_virginiana-buds
OleaEuropaea_Oeu009726.2
IpomoeaNil_INIL09g31497.t1
CuscutaAustralis_C028N0093E0.1
IpomoeaNil_INIL03g17953.t1
SolanumTuberosum_PGSC0003DMT400060304
SolanumTuberosum_PGSC0003DMT400064261
SolanumTuberosum_PGSC0003DMT400070136
CoffeaCanephora_Cc09_g03940
VacciniumCorymbosum_VaccDscaff17-snap-gene-203.40-mRNA-1
VacciniumCorymbosum_VaccDscaff45-snap-gene-181.35-mRNA-1
VacciniumCorymbosum_VaccDscaff27-snap-gene-181.38-mRNA-1
Actinidiaeriantha_DTZ79_22g03500
Actinidiaeriantha_DTZ79_24g05820
OrobancheCumana_GWHTBHPV025891
Daucuscarota_DCAR_006478
HelianthusAnnuus_OTF91539.1
GossypiumRaimondii_KJB13559
GossypiumRaimondii_KJB13561
GossypiumRaimondii_KJB13560
GossypiumRaimondii_KJB80707
GossypiumRaimondii_KJB13500
GossypiumRaimondii_KJB13501
GossypiumRaimondii_KJB13499
TheobromaCacao_Thecc1EG025086t1
CitrusSinensis_orange1.1g033119m
CitrusSinensis_orange1.1g042770m
ArabidopsisThaliana_AT1G65290.1
ManihotEsculenta_Manes.12G003400.1.p
ManihotEsculenta_Manes.13G003300.1.p
PopulusTrichocarpa_Potri.013G084500.1
PopulusTrichocarpa_Potri.019G055300.1
GlycineMax_Glyma.09G248500.1.p
GlycineMax_Glyma.18G244300.1.p
GlycineMax_Glyma.09G060900.1.p
PrunusPersica_Prupe.6G363300.1.p
VitisVinifera_GSVIVT01019059001
AquilegiaCoerulea_PIA48261.1
CinnamomumMicranthum_RWR82546.1
OryzaSativa_LOC_Os03g22950.1
OryzaSativa_LOC_Os03g23000.1
SorghumBicolor_Sobic.001G370700.1
OryzaSativa_LOC_Os05g31290.1
SorghumBicolor_Sobic.009G119900.1
AmborellaTrichopoda_ERN05091.1
0.2
