# Supplementary material for: Altered Mitochondrial Respiration Is Associated With Loss of Nuclear‐Encoded OXPHOS Genes in Parasitic Broomrapes
Source: Ecol Evol. 2025 Jul 6;15(7):e71737. doi: 10.1002/ece3.71737 (PMC12230201; doi:10.1002/ece3.71737)
Supplement: Supplementary file 1 — Appendix S1. [file ECE3-15-e71737-s003.tgz › Orobanchaceae_O2K_Supplementary_v3/Data_S1/DataS1/CI/OG0003871.filter.fas.treefile.pdf]

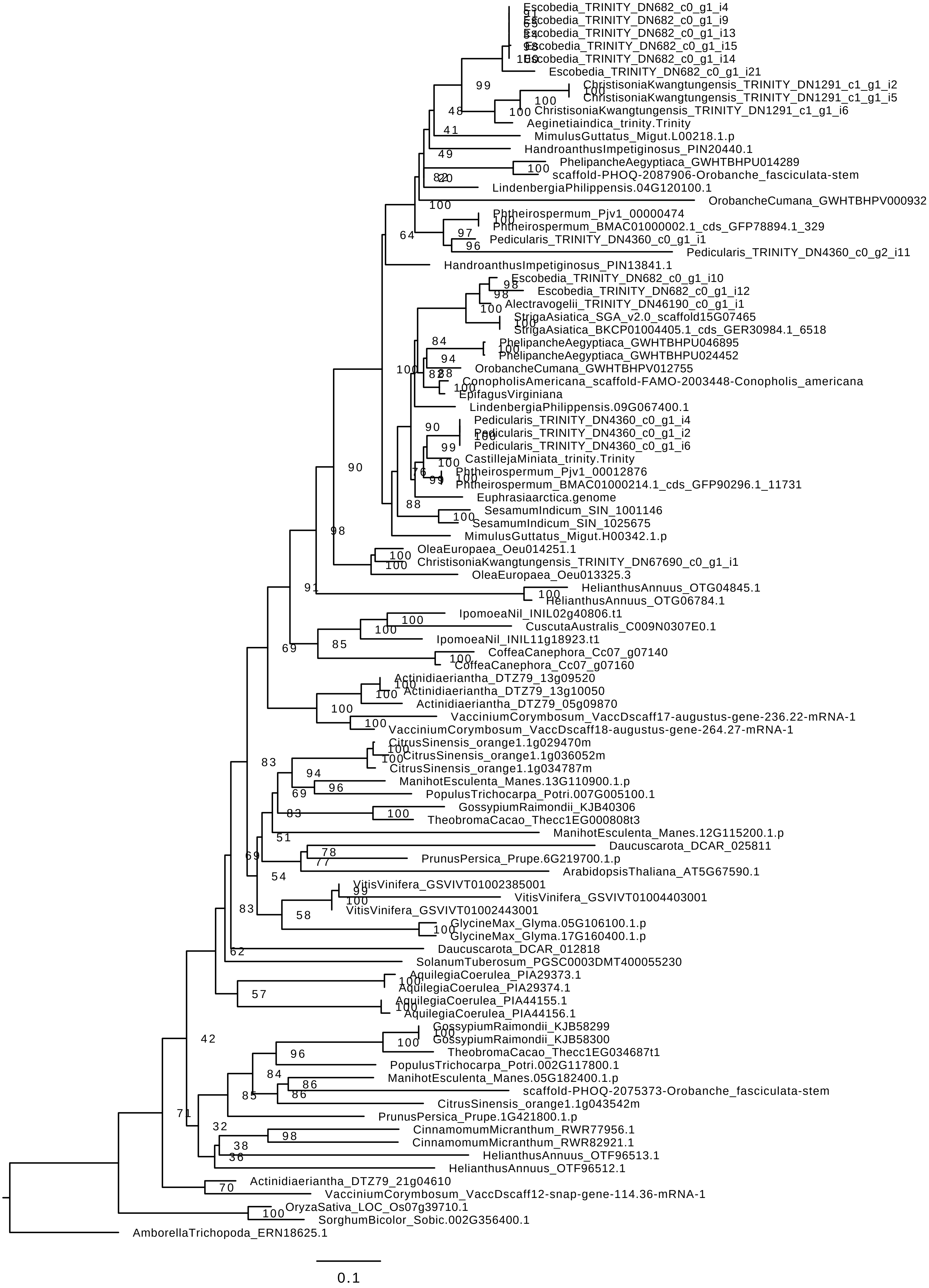
Escobedia_TRINITY_DN682_c0_g1_i4
Escobedia_TRINITY_DN682_c0_g1_i9
Escobedia_TRINITY_DN682_c0_g1_i13
Escobedia_TRINITY_DN682_c0_g1_i15
Escobedia_TRINITY_DN682_c0_g1_i14
Escobedia_TRINITY_DN682_c0_g1_i21
ChristisoniaKwangtungensis_TRINITY_DN1291_c1_g1_i2
ChristisoniaKwangtungensis_TRINITY_DN1291_c1_g1_i5
ChristisoniaKwangtungensis_TRINITY_DN1291_c1_g1_i6
Aeginetiaindica_trinity.Trinity
MimulusGuttatus_Migut.L00218.1.p
HandroanthusImpetiginosus_PIN20440.1
PhelipancheAegyptiaca_GWHTBHPU014289
scaffold-PHOQ-2087906-Orobanche_fasciculata-stem
LindenbergiaPhilippensis.04G120100.1
OrobancheCumana_GWHTBHPV000932
Phtheirospermum_Pjv1_00000474
Phtheirospermum_BMAC01000002.1_cds_GFP78894.1_329
Pedicularis_TRINITY_DN4360_c0_g1_i1
Pedicularis_TRINITY_DN4360_c0_g2_i11
HandroanthusImpetiginosus_PIN13841.1
Escobedia_TRINITY_DN682_c0_g1_i10
Escobedia_TRINITY_DN682_c0_g1_i12
Alectravogelii_TRINITY_DN46190_c0_g1_i1
StrigaAsiatica_SGA_v2.0_scaffold15G07465
StrigaAsiatica_BKCP01004405.1_cds_GER30984.1_6518
PhelipancheAegyptiaca_GWHTBHPU046895
PhelipancheAegyptiaca_GWHTBHPU024452
OrobancheCumana_GWHTBHPV012755
ConopholisAmericana_scaffold-FAMO-2003448-Conopholis_americana
EpifagusVirginiana
LindenbergiaPhilippensis.09G067400.1
Pedicularis_TRINITY_DN4360_c0_g1_i4
Pedicularis_TRINITY_DN4360_c0_g1_i2
Pedicularis_TRINITY_DN4360_c0_g1_i6
CastillejaMiniata_trinity.Trinity
Phtheirospermum_Pjv1_00012876
Phtheirospermum_BMAC01000214.1_cds_GFP90296.1_11731
Euphrasiaarctica.genome
SesamumIndicum_SIN_1001146
SesamumIndicum_SIN_1025675
MimulusGuttatus_Migut.H00342.1.p
OleaEuropaea_Oeu014251.1
ChristisoniaKwangtungensis_TRINITY_DN67690_c0_g1_i1
OleaEuropaea_Oeu013325.3
HelianthusAnnuus_OTG04845.1
HelianthusAnnuus_OTG06784.1
IpomoeaNil_INIL02g40806.t1
CuscutaAustralis_C009N0307E0.1
IpomoeaNil_INIL11g18923.t1
CoffeaCanephora_Cc07_g07140
CoffeaCanephora_Cc07_g07160
Actinidiaeriantha_DTZ79_13g09520
Actinidiaeriantha_DTZ79_13g10050
Actinidiaeriantha_DTZ79_05g09870
VacciniumCorymbosum_VaccDscaff17-augustus-gene-236.22-mRNA-1
VacciniumCorymbosum_VaccDscaff18-augustus-gene-264.27-mRNA-1
CitrusSinensis_orange1.1g029470m
CitrusSinensis_orange1.1g036052m
CitrusSinensis_orange1.1g034787m
ManihotEsculenta_Manes.13G110900.1.p
PopulusTrichocarpa_Potri.007G005100.1
GossypiumRaimondii_KJB40306
TheobromaCacao_Thecc1EG000808t3
ManihotEsculenta_Manes.12G115200.1.p
Daucuscarota_DCAR_025811
PrunusPersica_Prupe.6G219700.1.p
ArabidopsisThaliana_AT5G67590.1
VitisVinifera_GSVIVT01002385001
VitisVinifera_GSVIVT01004403001
VitisVinifera_GSVIVT01002443001
GlycineMax_Glyma.05G106100.1.p
GlycineMax_Glyma.17G160400.1.p
Daucuscarota_DCAR_012818
SolanumTuberosum_PGSC0003DMT400055230
AquilegiaCoerulea_PIA29373.1
AquilegiaCoerulea_PIA29374.1
AquilegiaCoerulea_PIA44155.1
AquilegiaCoerulea_PIA44156.1
GossypiumRaimondii_KJB58299
GossypiumRaimondii_KJB58300
TheobromaCacao_Thecc1EG034687t1
PopulusTrichocarpa_Potri.002G117800.1
ManihotEsculenta_Manes.05G182400.1.p
scaffold-PHOQ-2075373-Orobanche_fasciculata-stem
CitrusSinensis_orange1.1g043542m
PrunusPersica_Prupe.1G421800.1.p
CinnamomumMicranthum_RWR77956.1
CinnamomumMicranthum_RWR82921.1
HelianthusAnnuus_OTF96513.1
HelianthusAnnuus_OTF96512.1
Actinidiaeriantha_DTZ79_21g04610
VacciniumCorymbosum_VaccDscaff12-snap-gene-114.36-mRNA-1
OryzaSativa_LOC_Os07g39710.1
SorghumBicolor_Sobic.002G356400.1
AmborellaTrichopoda_ERN18625.1
0.1
