# Supplementary material for: Altered Mitochondrial Respiration Is Associated With Loss of Nuclear‐Encoded OXPHOS Genes in Parasitic Broomrapes
Source: Ecol Evol. 2025 Jul 6;15(7):e71737. doi: 10.1002/ece3.71737 (PMC12230201; doi:10.1002/ece3.71737)
Supplement: Supplementary file 1 — Appendix S1. [file ECE3-15-e71737-s003.tgz › Orobanchaceae_O2K_Supplementary_v3/Data_S1/DataS1/CI/OG0007849.filter.fas.treefile.pdf]

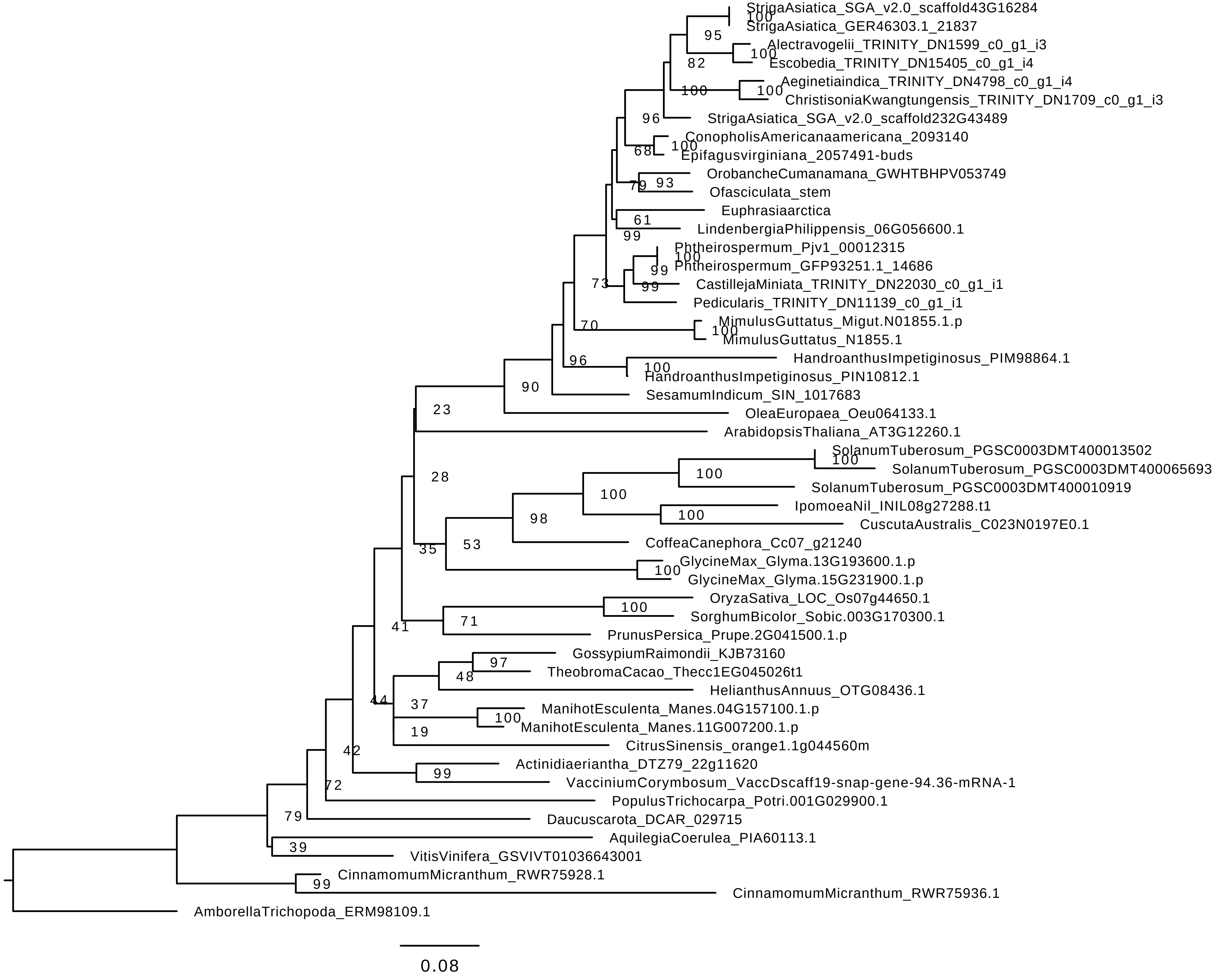
StrigaAsiatica_SGA_v2.0_scaffold43G16284
StrigaAsiatica_GER46303.1_21837
Alectravogelii_TRINITY_DN1599_c0_g1_i3
Escobedia_TRINITY_DN15405_c0_g1_i4
Aeginetiaindica_TRINITY_DN4798_c0_g1_i4
ChristisoniaKwangtungensis_TRINITY_DN1709_c0_g1_i3
StrigaAsiatica_SGA_v2.0_scaffold232G43489
ConopholisAmericanaamericana_2093140
Epifagusvirginiana_2057491-buds
OrobancheCumanamana_GWHTBHPV053749
Ofasciculata_stem
Euphrasiaarctica
LindenbergiaPhilippensis_06G056600.1
Phtheirospermum_Pjv1_00012315
Phtheirospermum_GFP93251.1_14686
CastillejaMiniata_TRINITY_DN22030_c0_g1_i1
Pedicularis_TRINITY_DN11139_c0_g1_i1
MimulusGuttatus_Migut.N01855.1.p
MimulusGuttatus_N1855.1
HandroanthusImpetiginosus_PIM98864.1
HandroanthusImpetiginosus_PIN10812.1
SesamumIndicum_SIN_1017683
OleaEuropaea_Oeu064133.1
ArabidopsisThaliana_AT3G12260.1
SolanumTuberosum_PGSC0003DMT400013502
SolanumTuberosum_PGSC0003DMT400065693
SolanumTuberosum_PGSC0003DMT400010919
IpomoeaNil_INIL08g27288.t1
CuscutaAustralis_C023N0197E0.1
CoffeaCanephora_Cc07_g21240
GlycineMax_Glyma.13G193600.1.p
GlycineMax_Glyma.15G231900.1.p
OryzaSativa_LOC_Os07g44650.1
SorghumBicolor_Sobic.003G170300.1
PrunusPersica_Prupe.2G041500.1.p
GossypiumRaimondii_KJB73160
TheobromaCacao_Thecc1EG045026t1
HelianthusAnnuus_OTG08436.1
ManihotEsculenta_Manes.04G157100.1.p
ManihotEsculenta_Manes.11G007200.1.p
CitrusSinensis_orange1.1g044560m
Actinidiaeriantha_DTZ79_22g11620
VacciniumCorymbosum_VaccDscaff19-snap-gene-94.36-mRNA-1
PopulusTrichocarpa_Potri.001G029900.1
Daucuscarota_DCAR_029715
AquilegiaCoerulea_PIA60113.1
VitisVinifera_GSVIVT01036643001
CinnamomumMicranthum_RWR75928.1
CinnamomumMicranthum_RWR75936.1
AmborellaTrichopoda_ERM98109.1
0.08
