# Supplementary material for: Altered Mitochondrial Respiration Is Associated With Loss of Nuclear‐Encoded OXPHOS Genes in Parasitic Broomrapes
Source: Ecol Evol. 2025 Jul 6;15(7):e71737. doi: 10.1002/ece3.71737 (PMC12230201; doi:10.1002/ece3.71737)
Supplement: Supplementary file 1 — Appendix S1. [file ECE3-15-e71737-s003.tgz › Orobanchaceae_O2K_Supplementary_v3/Data_S1/DataS1/CI/OG0005677.filter.fas.treefile.pdf]

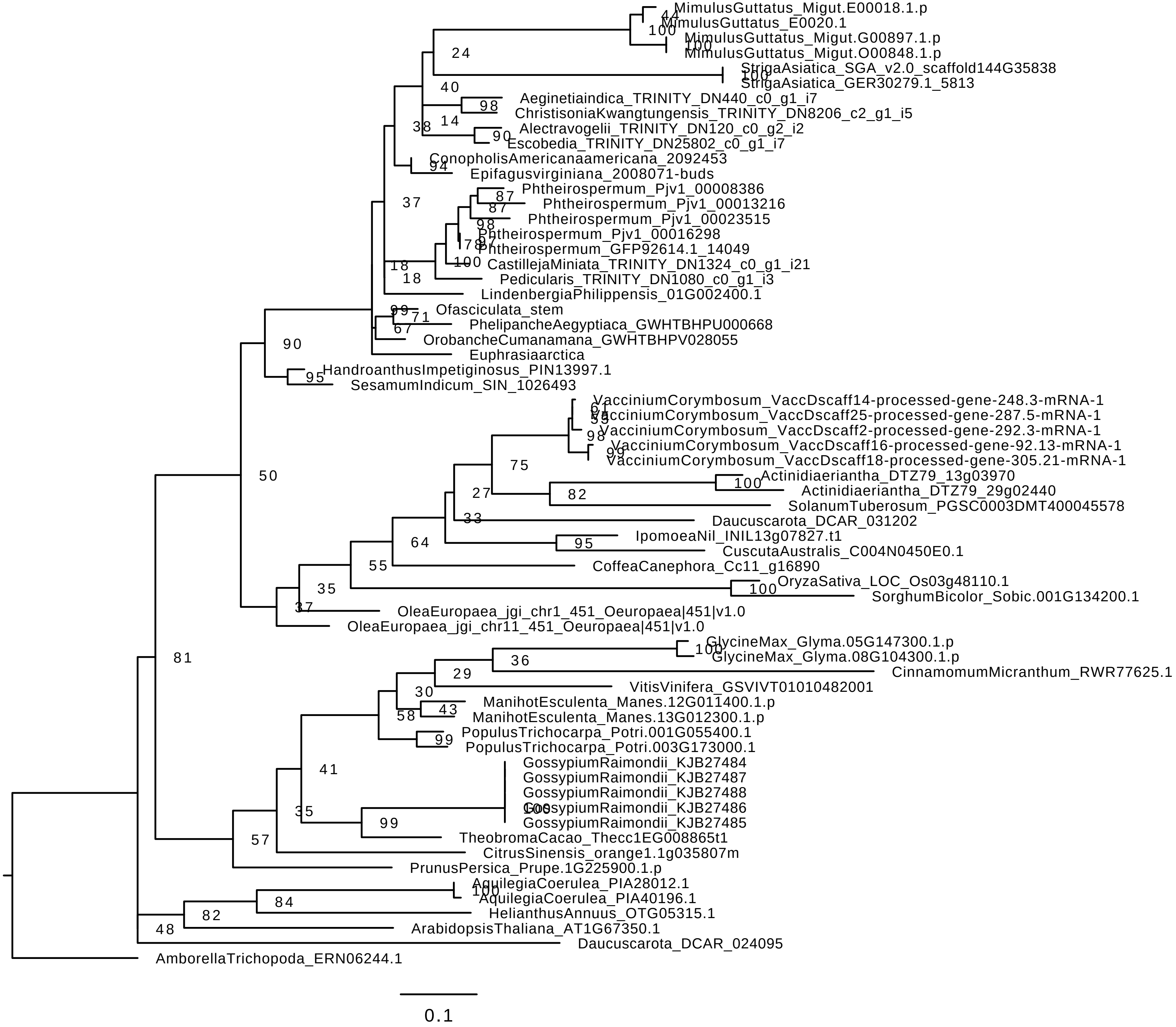
MimulusGuttatus_Migut.E00018.1.p
MimulusGuttatus_E0020.1
MimulusGuttatus_Migut.G00897.1.p
MimulusGuttatus_Migut.O00848.1.p
StrigaAsiatica_SGA_v2.0_scaffold144G35838
StrigaAsiatica_GER30279.1_5813
Aeginetiaindica_TRINITY_DN440_c0_g1_i7
ChristisoniaKwangtungensis_TRINITY_DN8206_c2_g1_i5
Alectravogelii_TRINITY_DN120_c0_g2_i2
Escobedia_TRINITY_DN25802_c0_g1_i7
ConopholisAmericanaamericana_2092453
Epifagusvirginiana_2008071-buds
Phtheirospermum_Pjv1_00008386
Phtheirospermum_Pjv1_00013216
Phtheirospermum_Pjv1_00023515
Phtheirospermum_Pjv1_00016298
Phtheirospermum_GFP92614.1_14049
CastillejaMiniata_TRINITY_DN1324_c0_g1_i21
Pedicularis_TRINITY_DN1080_c0_g1_i3
LindenbergiaPhilippensis_01G002400.1
Ofasciculata_stem
PhelipancheAegyptiaca_GWHTBHPU000668
OrobancheCumanamana_GWHTBHPV028055
Euphrasiaarctica
HandroanthusImpetiginosus_PIN13997.1
SesamumIndicum_SIN_1026493
VacciniumCorymbosum_VaccDscaff14-processed-gene-248.3-mRNA-1
VacciniumCorymbosum_VaccDscaff25-processed-gene-287.5-mRNA-1
VacciniumCorymbosum_VaccDscaff2-processed-gene-292.3-mRNA-1
VacciniumCorymbosum_VaccDscaff16-processed-gene-92.13-mRNA-1
VacciniumCorymbosum_VaccDscaff18-processed-gene-305.21-mRNA-1
Actinidiaeriantha_DTZ79_13g03970
Actinidiaeriantha_DTZ79_29g02440
SolanumTuberosum_PGSC0003DMT400045578
Daucuscarota_DCAR_031202
IpomoeaNil_INIL13g07827.t1
CuscutaAustralis_C004N0450E0.1
CoffeaCanephora_Cc11_g16890
OryzaSativa_LOC_Os03g48110.1
SorghumBicolor_Sobic.001G134200.1
OleaEuropaea_jgi_chr1_451_Oeuropaea|451|v1.0
OleaEuropaea_jgi_chr11_451_Oeuropaea|451|v1.0
GlycineMax_Glyma.05G147300.1.p
GlycineMax_Glyma.08G104300.1.p
CinnamomumMicranthum_RWR77625.1
VitisVinifera_GSVIVT01010482001
ManihotEsculenta_Manes.12G011400.1.p
ManihotEsculenta_Manes.13G012300.1.p
PopulusTrichocarpa_Potri.001G055400.1
PopulusTrichocarpa_Potri.003G173000.1
GossypiumRaimondii_KJB27484
GossypiumRaimondii_KJB27487
GossypiumRaimondii_KJB27488
GossypiumRaimondii_KJB27486
GossypiumRaimondii_KJB27485
TheobromaCacao_Thecc1EG008865t1
CitrusSinensis_orange1.1g035807m
PrunusPersica_Prupe.1G225900.1.p
AquilegiaCoerulea_PIA28012.1
AquilegiaCoerulea_PIA40196.1
HelianthusAnnuus_OTG05315.1
ArabidopsisThaliana_AT1G67350.1
Daucuscarota_DCAR_024095
AmborellaTrichopoda_ERN06244.1
0.1
