# Supplementary material for: Altered Mitochondrial Respiration Is Associated With Loss of Nuclear‐Encoded OXPHOS Genes in Parasitic Broomrapes
Source: Ecol Evol. 2025 Jul 6;15(7):e71737. doi: 10.1002/ece3.71737 (PMC12230201; doi:10.1002/ece3.71737)
Supplement: Supplementary file 1 — Appendix S1. [file ECE3-15-e71737-s003.tgz › Orobanchaceae_O2K_Supplementary_v3/Data_S1/DataS1/CI/OG0008473.filter.fas.treefile.pdf]

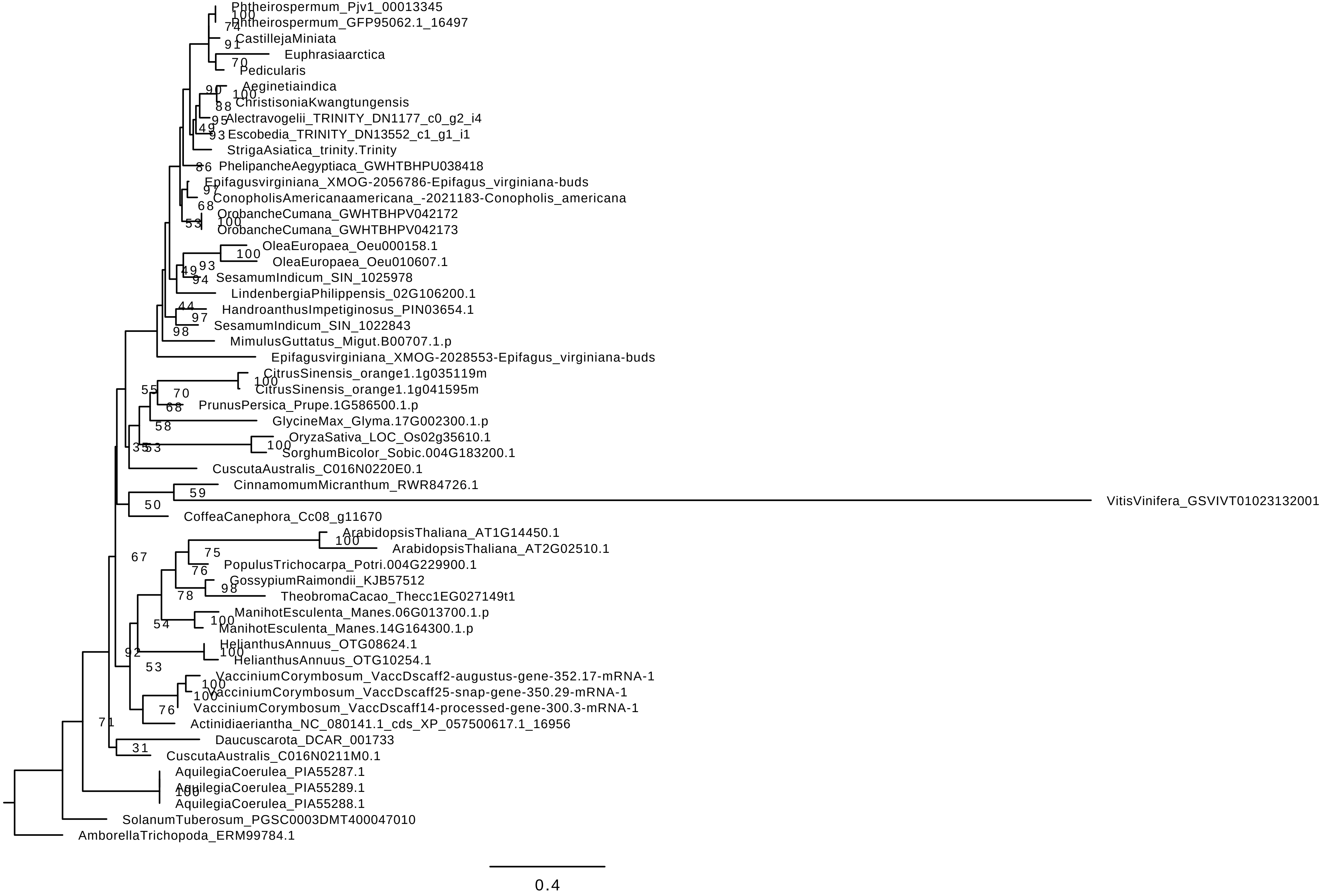

Phtheirospermum_Pjv1_00013345
Phtheirospermum_GFP95062.1_16497
CastillejaMiniata
Euphrasiaarctica
Pedicularis
Aeginetiaindica
ChristisoniaKwangtungensis
Alectravogelii_TRINITY_DN1177_c0_g2_i4
Escobedia_TRINITY_DN13552_c1_g1_i1
StrigaAsiatica_trinity.Trinity
PhelipancheAegyptiaca_GWHTBHPU038418
Epifagusvirginiana_XMOG-2056786-Epifagus_virginiana-buds
ConopholisAmericanaamericana_-2021183-Conopholis_americana
OrobancheCumana_GWHTBHPV042172
OrobancheCumana_GWHTBHPV042173
OleaEuropaea_Oeu000158.1
OleaEuropaea_Oeu010607.1
SesamumIndicum_SIN_1025978
LindenbergiaPhilippensis_02G106200.1
HandroanthusImpetiginosus_PIN03654.1
SesamumIndicum_SIN_1022843
MimulusGuttatus_Migut.B00707.1.p
Epifagusvirginiana_XMOG-2028553-Epifagus_virginiana-buds
CitrusSinensis_orange1.1g035119m
CitrusSinensis_orange1.1g041595m
PrunusPersica_Prupe.1G586500.1.p
GlycineMax_Glyma.17G002300.1.p
OryzaSativa_LOC_Os02g35610.1
SorghumBicolor_Sobic.004G183200.1
CuscutaAustralis_C016N0220E0.1
CinnamomumMicranthum_RWR84726.1
VitisVinifera_GSVIVT01023132001
CoffeaCanephora_Cc08_g11670
ArabidopsisThaliana_AT1G14450.1
ArabidopsisThaliana_AT2G02510.1
PopulusTrichocarpa_Potri.004G229900.1
GossypiumRaimondii_KJB57512
TheobromaCacao_Thecc1EG027149t1
ManihotEsculenta_Manes.06G013700.1.p
ManihotEsculenta_Manes.14G164300.1.p
HelianthusAnnuus_OTG08624.1
HelianthusAnnuus_OTG10254.1
VacciniumCorymbosum_VaccDscaff2-augustus-gene-352.17-mRNA-1
VacciniumCorymbosum_VaccDscaff25-snap-gene-350.29-mRNA-1
VacciniumCorymbosum_VaccDscaff14-processed-gene-300.3-mRNA-1
Actinidiaeriantha_NC_080141.1_cds_XP_057500617.1_16956
Daucuscarota_DCAR_001733
CuscutaAustralis_C016N0211M0.1
AquilegiaCoerulea_PIA55287.1
AquilegiaCoerulea_PIA55289.1
AquilegiaCoerulea_PIA55288.1
SolanumTuberosum_PGSC0003DMT400047010
AmborellaTrichopoda_ERM99784.1
0.4
