# Supplementary material for: Altered Mitochondrial Respiration Is Associated With Loss of Nuclear‐Encoded OXPHOS Genes in Parasitic Broomrapes
Source: Ecol Evol. 2025 Jul 6;15(7):e71737. doi: 10.1002/ece3.71737 (PMC12230201; doi:10.1002/ece3.71737)
Supplement: Supplementary file 1 — Appendix S1. [file ECE3-15-e71737-s003.tgz › Orobanchaceae_O2K_Supplementary_v3/Data_S1/DataS1/CI/OG0005731.filter.fas.treefile.pdf]

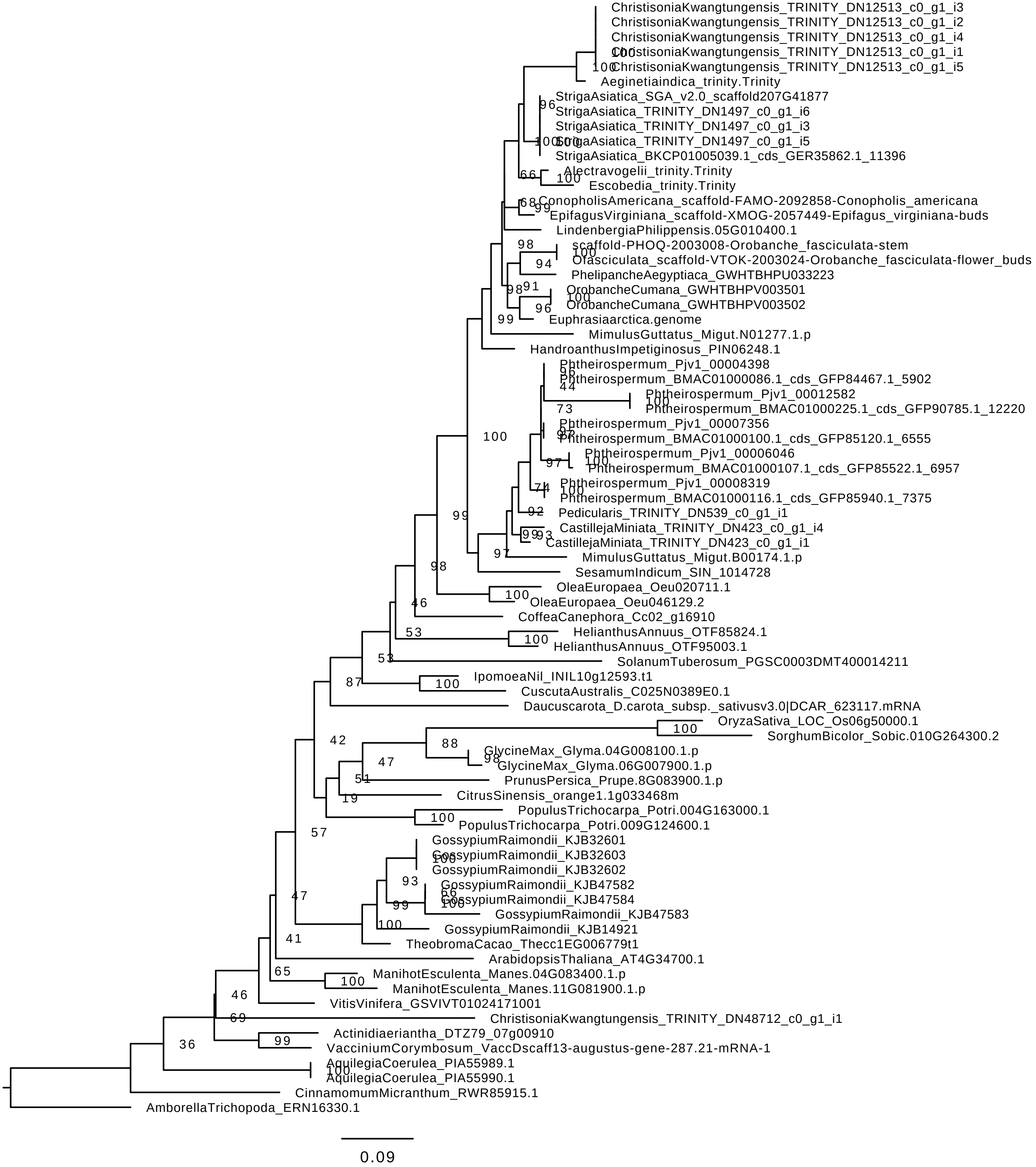
ChristisoniaKwangtungensis_TRINITY_DN12513_c0_g1_i3
ChristisoniaKwangtungensis_TRINITY_DN12513_c0_g1_i2
ChristisoniaKwangtungensis_TRINITY_DN12513_c0_g1_i4
ChristisoniaKwangtungensis_TRINITY_DN12513_c0_g1_i1
ChristisoniaKwangtungensis_TRINITY_DN12513_c0_g1_i5
Aeginetiaindica_trinity.Trinity
StrigaAsiatica_SGA_v2.0_scaffold207G41877
StrigaAsiatica_TRINITY_DN1497_c0_g1_i6
StrigaAsiatica_TRINITY_DN1497_c0_g1_i3
StrigaAsiatica_TRINITY_DN1497_c0_g1_i5
StrigaAsiatica_BKCP01005039.1_cds_GER35862.1_11396
Alectravogelii_trinity.Trinity
Escobedia_trinity.Trinity
ConopholisAmericana_scaffold-FAMO-2092858-Conopholis_americana
EpifagusVirginiana_scaffold-XMOG-2057449-Epifagus_virginiana-buds
LindenbergiaPhilippensis.05G010400.1
scaffold-PHOQ-2003008-Orobanche_fasciculata-stem
Ofasciculata_scaffold-VTOK-2003024-Orobanche_fasciculata-flower_buds
PhelipancheAegyptiaca_GWHTBHPU033223
OrobancheCumana_GWHTBHPV003501
OrobancheCumana_GWHTBHPV003502
Euphrasiaarctica.genome
MimulusGuttatus_Migut.N01277.1.p
HandroanthusImpetiginosus_PIN06248.1
Phtheirospermum_Pjv1_00004398
Phtheirospermum_BMAC01000086.1_cds_GFP84467.1_5902
Phtheirospermum_Pjv1_00012582
Phtheirospermum_BMAC01000225.1_cds_GFP90785.1_12220
Phtheirospermum_Pjv1_00007356
Phtheirospermum_BMAC01000100.1_cds_GFP85120.1_6555
Phtheirospermum_Pjv1_00006046
Phtheirospermum_BMAC01000107.1_cds_GFP85522.1_6957
Phtheirospermum_Pjv1_00008319
Phtheirospermum_BMAC01000116.1_cds_GFP85940.1_7375
Pedicularis_TRINITY_DN539_c0_g1_i1
CastillejaMiniata_TRINITY_DN423_c0_g1_i4
CastillejaMiniata_TRINITY_DN423_c0_g1_i1
MimulusGuttatus_Migut.B00174.1.p
SesamumIndicum_SIN_1014728
OleaEuropaea_Oeu020711.1
OleaEuropaea_Oeu046129.2
CoffeaCanephora_Cc02_g16910
HelianthusAnnuus_OTF85824.1
HelianthusAnnuus_OTF95003.1
SolanumTuberosum_PGSC0003DMT400014211
IpomoeaNil_INIL10g12593.t1
CuscutaAustralis_C025N0389E0.1
Daucuscarota_D.carota_subsp._sativusv3.0|DCAR_623117.mRNA
OryzaSativa_LOC_Os06g50000.1
SorghumBicolor_Sobic.010G264300.2
GlycineMax_Glyma.04G008100.1.p
GlycineMax_Glyma.06G007900.1.p
PrunusPersica_Prupe.8G083900.1.p
CitrusSinensis_orange1.1g033468m
PopulusTrichocarpa_Potri.004G163000.1
PopulusTrichocarpa_Potri.009G124600.1
GossypiumRaimondii_KJB32601
GossypiumRaimondii_KJB32603
GossypiumRaimondii_KJB32602
GossypiumRaimondii_KJB47582
GossypiumRaimondii_KJB47584
GossypiumRaimondii_KJB47583
GossypiumRaimondii_KJB14921
TheobromaCacao_Thecc1EG006779t1
ArabidopsisThaliana_AT4G34700.1
ManihotEsculenta_Manes.04G083400.1.p
ManihotEsculenta_Manes.11G081900.1.p
VitisVinifera_GSVIVT01024171001
ChristisoniaKwangtungensis_TRINITY_DN48712_c0_g1_i1
Actinidiaeriantha_DTZ79_07g00910
VacciniumCorymbosum_VaccDscaff13-augustus-gene-287.21-mRNA-1
AquilegiaCoerulea_PIA55989.1
AquilegiaCoerulea_PIA55990.1
CinnamomumMicranthum_RWR85915.1
AmborellaTrichopoda_ERN16330.1
0.09
