# Supplementary material for: Altered Mitochondrial Respiration Is Associated With Loss of Nuclear‐Encoded OXPHOS Genes in Parasitic Broomrapes
Source: Ecol Evol. 2025 Jul 6;15(7):e71737. doi: 10.1002/ece3.71737 (PMC12230201; doi:10.1002/ece3.71737)
Supplement: Supplementary file 1 — Appendix S1. [file ECE3-15-e71737-s003.tgz › Orobanchaceae_O2K_Supplementary_v3/Data_S1/DataS1/CI/OG0005588.filter.fas.treefile.pdf]

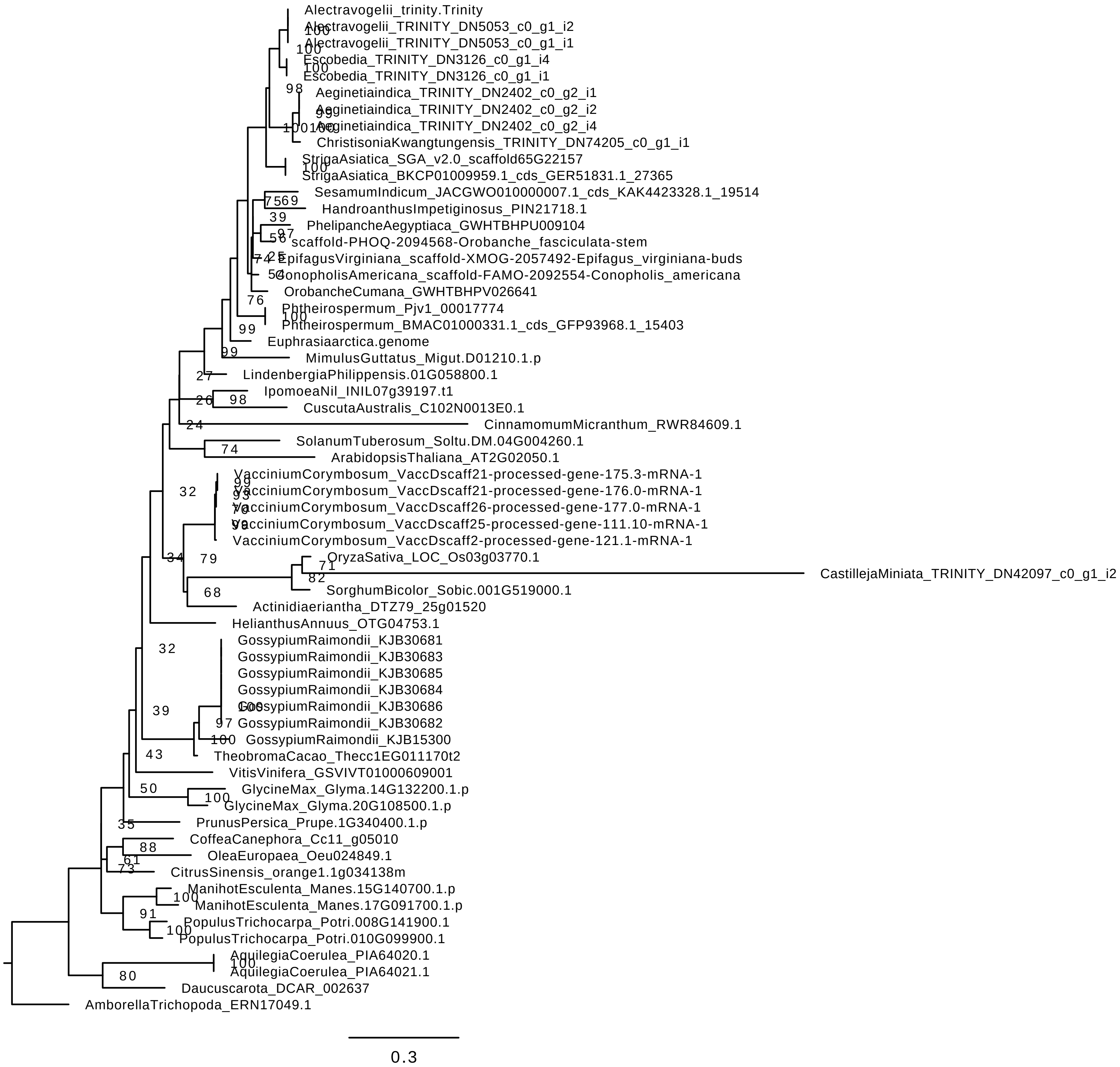
Alectravogelii_trinity.Trinity
Alectravogelii_TRINITY_DN5053_c0_g1_i2
Alectravogelii_TRINITY_DN5053_c0_g1_i1
Escobedia_TRINITY_DN3126_c0_g1_i4
Escobedia_TRINITY_DN3126_c0_g1_i1
Aeginetiaindica_TRINITY_DN2402_c0_g2_i1
Aeginetiaindica_TRINITY_DN2402_c0_g2_i2
Aeginetiaindica_TRINITY_DN2402_c0_g2_i4
ChristisoniaKwangtungensis_TRINITY_DN74205_c0_g1_i1
StrigaAsiatica_SGA_v2.0_scaffold65G22157
StrigaAsiatica_BKCP01009959.1_cds_GER51831.1_27365
SesamumIndicum_JACGWO010000007.1_cds_KAK4423328.1_19514
HandroanthusImpetiginosus_PIN21718.1
PhelipancheAegyptiaca_GWHTBHPU009104
scaffold-PHOQ-2094568-Orobanche_fasciculata-stem
EpifagusVirginiana_scaffold-XMOG-2057492-Epifagus_virginiana-buds
ConopholisAmericana_scaffold-FAMO-2092554-Conopholis_americana
OrobancheCumana_GWHTBHPV026641
Phtheirospermum_Pjv1_00017774
Phtheirospermum_BMAC01000331.1_cds_GFP93968.1_15403
Euphrasiaarctica.genome
MimulusGuttatus_Migut.D01210.1.p
LindenbergiaPhilippensis.01G058800.1
IpomoeaNil_INIL07g39197.t1
CuscutaAustralis_C102N0013E0.1
CinnamomumMicranthum_RWR84609.1
SolanumTuberosum_Soltu.DM.04G004260.1
ArabidopsisThaliana_AT2G02050.1
VacciniumCorymbosum_VaccDscaff21-processed-gene-175.3-mRNA-1
VacciniumCorymbosum_VaccDscaff21-processed-gene-176.0-mRNA-1
VacciniumCorymbosum_VaccDscaff26-processed-gene-177.0-mRNA-1
VacciniumCorymbosum_VaccDscaff25-processed-gene-111.10-mRNA-1
VacciniumCorymbosum_VaccDscaff2-processed-gene-121.1-mRNA-1
OryzaSativa_LOC_Os03g03770.1
CastillejaMiniata_TRINITY_DN42097_c0_g1_i2
SorghumBicolor_Sobic.001G519000.1
Actinidiaeriantha_DTZ79_25g01520
HelianthusAnnuus_OTG04753.1
GossypiumRaimondii_KJB30681
GossypiumRaimondii_KJB30683
GossypiumRaimondii_KJB30685
GossypiumRaimondii_KJB30684
GossypiumRaimondii_KJB30686
GossypiumRaimondii_KJB30682
GossypiumRaimondii_KJB15300
TheobromaCacao_Thecc1EG011170t2
VitisVinifera_GSVIVT01000609001
GlycineMax_Glyma.14G132200.1.p
GlycineMax_Glyma.20G108500.1.p
PrunusPersica_Prupe.1G340400.1.p
CoffeaCanephora_Cc11_g05010
OleaEuropaea_Oeu024849.1
CitrusSinensis_orange1.1g034138m
ManihotEsculenta_Manes.15G140700.1.p
ManihotEsculenta_Manes.17G091700.1.p
PopulusTrichocarpa_Potri.008G141900.1
PopulusTrichocarpa_Potri.010G099900.1
AquilegiaCoerulea_PIA64020.1
AquilegiaCoerulea_PIA64021.1
Daucuscarota_DCAR_002637
AmborellaTrichopoda_ERN17049.1
0.3
