# Supplementary material for: Altered Mitochondrial Respiration Is Associated With Loss of Nuclear‐Encoded OXPHOS Genes in Parasitic Broomrapes
Source: Ecol Evol. 2025 Jul 6;15(7):e71737. doi: 10.1002/ece3.71737 (PMC12230201; doi:10.1002/ece3.71737)
Supplement: Supplementary file 1 — Appendix S1. [file ECE3-15-e71737-s003.tgz › Orobanchaceae_O2K_Supplementary_v3/Data_S1/DataS1/CI/OG0007140.filter.fas.treefile.pdf]

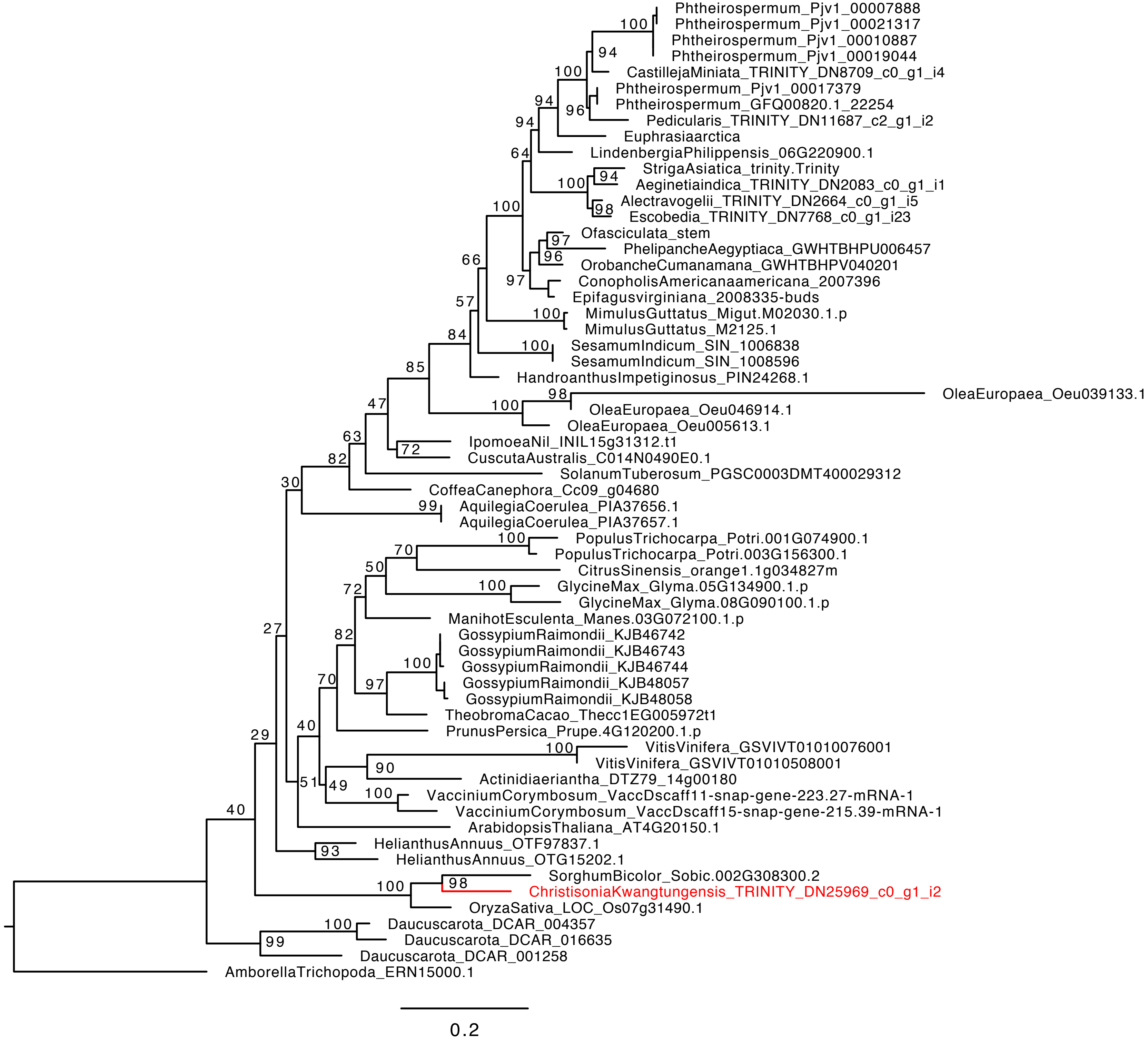
Phtheirospermum_Pjv1_00007888
Phtheirospermum_Pjv1_00021317
Phtheirospermum_Pjv1_00010887
Phtheirospermum_Pjv1_00019044
CastillejaMiniata_TRINITY_DN8709_c0_g1_i4
Phtheirospermum_Pjv1_00017379
Phtheirospermum_GFQ00820.1_22254
Pedicularis_TRINITY_DN11687_c2_g1_i2
Euphrasiaarctica
LindenbergiaPhilippensis_06G220900.1
StrigaAsiatica_trinity.Trinity
Aeginetiaindica_TRINITY_DN2083_c0_g1_i1
Alectravogelii_TRINITY_DN2664_c0_g1_i5
Escobedia_TRINITY_DN7768_c0_g1_i23
Ofasciculata_stem
PhelipancheAegyptiaca_GWHTBHPU006457
OrobancheCumanamana_GWHTBHPV040201
ConopholisAmericanaamericana_2007396
Epifagusvirginiana_2008335-buds
MimulusGuttatus_Migut.M02030.1.p
MimulusGuttatus_M2125.1
SesamumIndicum_SIN_1006838
SesamumIndicum_SIN_1008596
HandroanthusImpetiginosus_PIN24268.1
OleaEuropaea_Oeu039133.1
OleaEuropaea_Oeu046914.1
OleaEuropaea_Oeu005613.1
IpomoeaNil_INIL15g31312.t1
CuscutaAustralis_C014N0490E0.1
SolanumTuberosum_PGSC0003DMT400029312
CoffeaCanephora_Cc09_g04680
AquilegiaCoerulea_PIA37656.1
AquilegiaCoerulea_PIA37657.1
PopulusTrichocarpa_Potri.001G074900.1
PopulusTrichocarpa_Potri.003G156300.1
CitrusSinensis_orange1.1g034827m
GlycineMax_Glyma.05G134900.1.p
GlycineMax_Glyma.08G090100.1.p
ManihotEsculenta_Manes.03G072100.1.p
GossypiumRaimondii_KJB46742
GossypiumRaimondii_KJB46743
GossypiumRaimondii_KJB46744
GossypiumRaimondii_KJB48057
GossypiumRaimondii_KJB48058
TheobromaCacao_Thecc1EG005972t1
PrunusPersica_Prupe.4G120200.1.p
VitisVinifera_GSVIVT01010076001
VitisVinifera_GSVIVT01010508001
Actinidiaeriantha_DTZ79_14g00180
VacciniumCorymbosum_VaccDscaff11-snap-gene-223.27-mRNA-1
VacciniumCorymbosum_VaccDscaff15-snap-gene-215.39-mRNA-1
ArabidopsisThaliana_AT4G20150.1
HelianthusAnnuus_OTF97837.1
HelianthusAnnuus_OTG15202.1
SorghumBicolor_Sobic.002G308300.2
ChristisoniaKwangtungensis_TRINITY_DN25969_c0_g1_i2
OryzaSativa_LOC_Os07g31490.1
Daucuscarota_DCAR_004357
Daucuscarota_DCAR_016635
Daucuscarota_DCAR_001258
AmborellaTrichopoda_ERN15000.1
0.2
