# Supplementary material for: Altered Mitochondrial Respiration Is Associated With Loss of Nuclear‐Encoded OXPHOS Genes in Parasitic Broomrapes
Source: Ecol Evol. 2025 Jul 6;15(7):e71737. doi: 10.1002/ece3.71737 (PMC12230201; doi:10.1002/ece3.71737)
Supplement: Supplementary file 1 — Appendix S1. [file ECE3-15-e71737-s003.tgz › Orobanchaceae_O2K_Supplementary_v3/Data_S1/DataS1/CI/OG0005290.filter.fas.treefile.pdf]

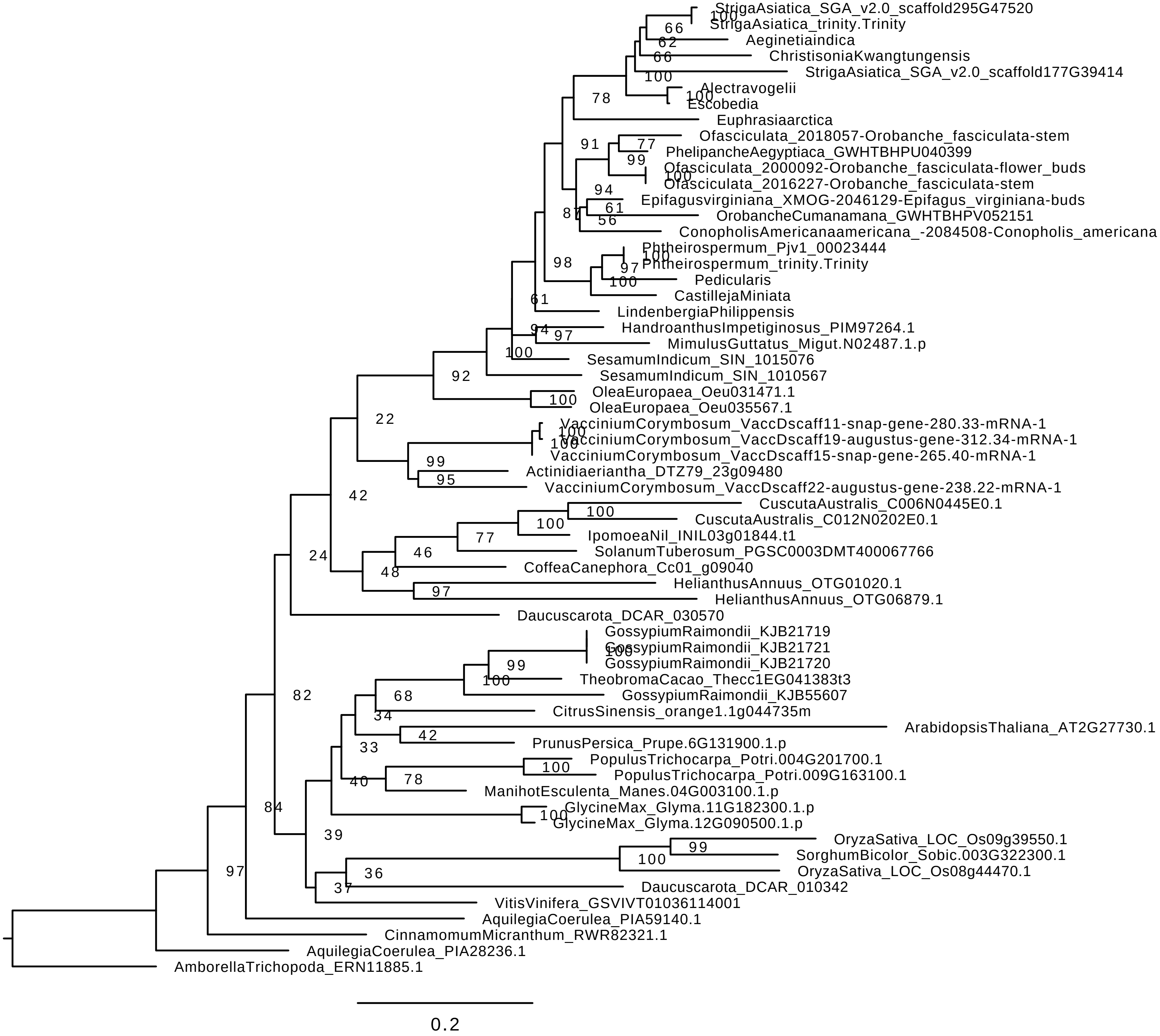
StrigaAsiatica_SGA_v2.0_scaffold295G47520
StrigaAsiatica_trinity.Trinity
Aeginetiaindica
ChristisoniaKwangtungensis
StrigaAsiatica_SGA_v2.0_scaffold177G39414
Alectravogelii
Escobedia
Euphrasiaarctica
Ofasciculata_2018057-Orobanche_fasciculata-stem
PhelipancheAegyptiaca_GWHTBHPU040399
Ofasciculata_2000092-Orobanche_fasciculata-flower_buds
Ofasciculata_2016227-Orobanche_fasciculata-stem
Epifagusvirginiana_XMOG-2046129-Epifagus_virginiana-buds
OrobancheCumanamana_GWHTBHPV052151
ConopholisAmericanaamericana_-2084508-Conopholis_americana
Phtheirospermum_Pjv1_00023444
Phtheirospermum_trinity.Trinity
Pedicularis
CastillejaMiniata
LindenbergiaPhilippensis
HandroanthusImpetiginosus_PIM97264.1
MimulusGuttatus_Migut.N02487.1.p
SesamumIndicum_SIN_1015076
SesamumIndicum_SIN_1010567
OleaEuropaea_Oeu031471.1
OleaEuropaea_Oeu035567.1
VacciniumCorymbosum_VaccDscaff11-snap-gene-280.33-mRNA-1
VacciniumCorymbosum_VaccDscaff19-augustus-gene-312.34-mRNA-1
VacciniumCorymbosum_VaccDscaff15-snap-gene-265.40-mRNA-1
Actinidiaeriantha_DTZ79_23g09480
VacciniumCorymbosum_VaccDscaff22-augustus-gene-238.22-mRNA-1
CuscutaAustralis_C006N0445E0.1
CuscutaAustralis_C012N0202E0.1
IpomoeaNil_INIL03g01844.t1
SolanumTuberosum_PGSC0003DMT400067766
CoffeaCanephora_Cc01_g09040
HelianthusAnnuus_OTG01020.1
HelianthusAnnuus_OTG06879.1
Daucuscarota_DCAR_030570
GossypiumRaimondii_KJB21719
GossypiumRaimondii_KJB21721
GossypiumRaimondii_KJB21720
TheobromaCacao_Thecc1EG041383t3
GossypiumRaimondii_KJB55607
CitrusSinensis_orange1.1g044735m
ArabidopsisThaliana_AT2G27730.1
PrunusPersica_Prupe.6G131900.1.p
PopulusTrichocarpa_Potri.004G201700.1
PopulusTrichocarpa_Potri.009G163100.1
ManihotEsculenta_Manes.04G003100.1.p
GlycineMax_Glyma.11G182300.1.p
GlycineMax_Glyma.12G090500.1.p
OryzaSativa_LOC_Os09g39550.1
SorghumBicolor_Sobic.003G322300.1
OryzaSativa_LOC_Os08g44470.1
Daucuscarota_DCAR_010342
VitisVinifera_GSVIVT01036114001
AquilegiaCoerulea_PIA59140.1
CinnamomumMicranthum_RWR82321.1
AquilegiaCoerulea_PIA28236.1
AmborellaTrichopoda_ERN11885.1
0.2
