# Supplementary material for: Altered Mitochondrial Respiration Is Associated With Loss of Nuclear‐Encoded OXPHOS Genes in Parasitic Broomrapes
Source: Ecol Evol. 2025 Jul 6;15(7):e71737. doi: 10.1002/ece3.71737 (PMC12230201; doi:10.1002/ece3.71737)
Supplement: Supplementary file 1 — Appendix S1. [file ECE3-15-e71737-s003.tgz › Orobanchaceae_O2K_Supplementary_v3/Data_S1/DataS1/CI/OG0002485.filter.fas.treefile.pdf]

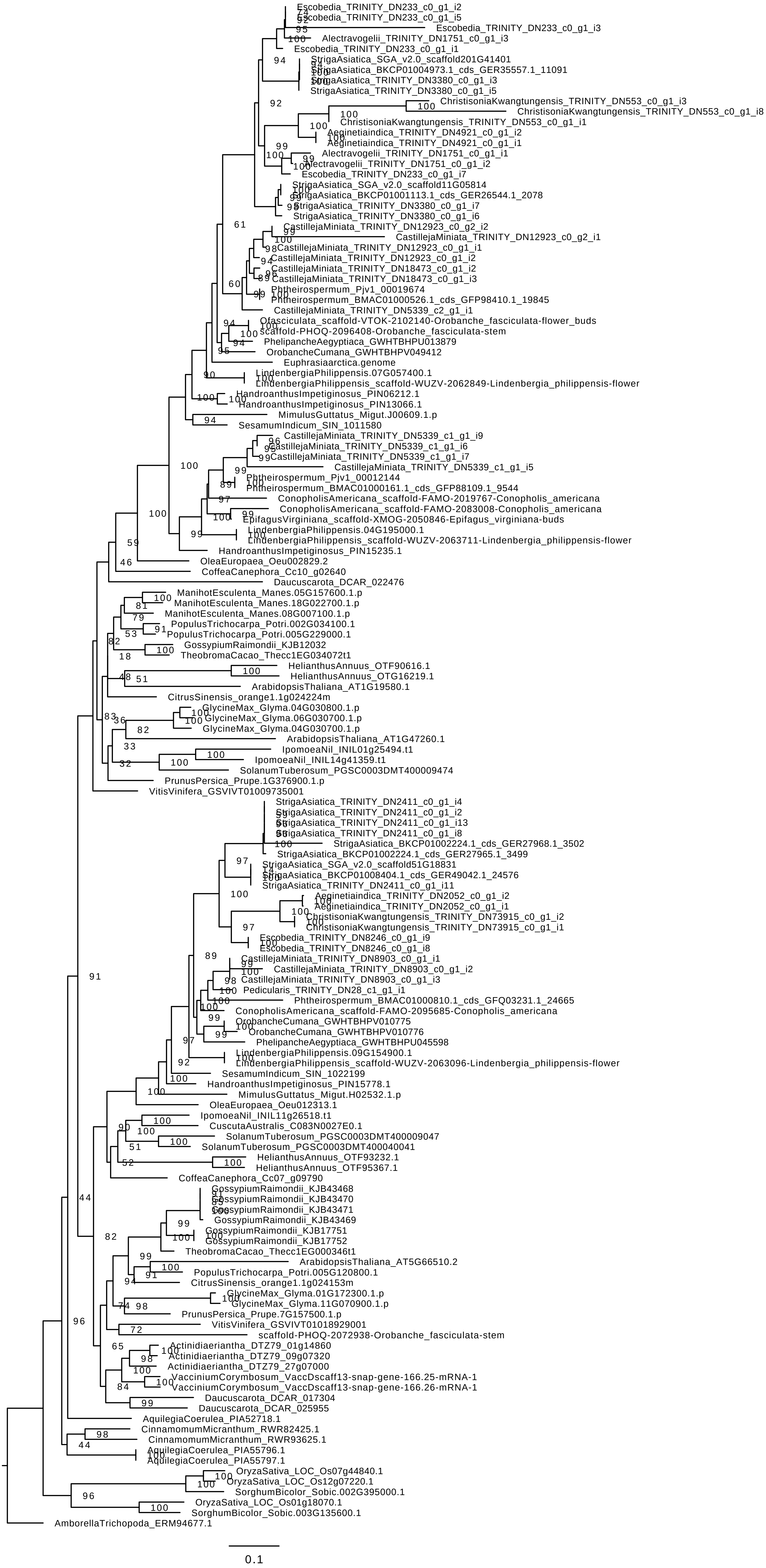
Escobedia_TRINITY_DN233_c0_g1_i2
Escobedia_TRINITY_DN233_c0_g1_i5
Escobedia_TRINITY_DN233_c0_g1_i3
Alectravogelii_TRINITY_DN1751_c0_g1_i3
Escobedia_TRINITY_DN233_c0_g1_i1
StrigaAsiatica_SGA_v2.0_scaffold201G41401
StrigaAsiatica_BKCP01004973.1_cds_GER35557.1_11091
StrigaAsiatica_TRINITY_DN3380_c0_g1_i3
StrigaAsiatica_TRINITY_DN3380_c0_g1_i5
ChristisoniaKwangtungensis_TRINITY_DN553_c0_g1_i3
ChristisoniaKwangtungensis_TRINITY_DN553_c0_g1_i8
ChristisoniaKwangtungensis_TRINITY_DN553_c0_g1_i1
Aeginetiaindica_TRINITY_DN4921_c0_g1_i2
Aeginetiaindica_TRINITY_DN4921_c0_g1_i1
Alectravogelii_TRINITY_DN1751_c0_g1_i1
Alectravogelii_TRINITY_DN1751_c0_g1_i2
Escobedia_TRINITY_DN233_c0_g1_i7
StrigaAsiatica_SGA_v2.0_scaffold11G05814
StrigaAsiatica_BKCP01001113.1_cds_GER26544.1_2078
StrigaAsiatica_TRINITY_DN3380_c0_g1_i7
StrigaAsiatica_TRINITY_DN3380_c0_g1_i6
CastillejaMiniata_TRINITY_DN12923_c0_g2_i2
CastillejaMiniata_TRINITY_DN12923_c0_g2_i1
CastillejaMiniata_TRINITY_DN12923_c0_g1_i1
CastillejaMiniata_TRINITY_DN12923_c0_g1_i2
CastillejaMiniata_TRINITY_DN18473_c0_g1_i2
CastillejaMiniata_TRINITY_DN18473_c0_g1_i3
Phtheirospermum_Pjv1_00019674
Phtheirospermum_BMAC01000526.1_cds_GFP98410.1_19845
CastillejaMiniata_TRINITY_DN5339_c2_g1_i1
Ofasciculata_scaffold-VTOK-2102140-Orobanche_fasciculata-flower_buds
scaffold-PHOQ-2096408-Orobanche_fasciculata-stem
PhelipancheAegyptiaca_GWHTBHPU013879
OrobancheCumana_GWHTBHPV049412
Euphrasiaarctica.genome
LindenbergiaPhilippensis.07G057400.1
LindenbergiaPhilippensis_scaffold-WUZV-2062849-Lindenbergia_philippensis-flower
HandroanthusImpetiginosus_PIN06212.1
HandroanthusImpetiginosus_PIN13066.1
MimulusGuttatus_Migut.J00609.1.p
SesamumIndicum_SIN_1011580
CastillejaMiniata_TRINITY_DN5339_c1_g1_i9
CastillejaMiniata_TRINITY_DN5339_c1_g1_i6
CastillejaMiniata_TRINITY_DN5339_c1_g1_i7
CastillejaMiniata_TRINITY_DN5339_c1_g1_i5
Phtheirospermum_Pjv1_00012144
Phtheirospermum_BMAC01000161.1_cds_GFP88109.1_9544
ConopholisAmericana_scaffold-FAMO-2019767-Conopholis_americana
ConopholisAmericana_scaffold-FAMO-2083008-Conopholis_americana
EpifagusVirginiana_scaffold-XMOG-2050846-Epifagus_virginiana-buds
LindenbergiaPhilippensis.04G195000.1
LindenbergiaPhilippensis_scaffold-WUZV-2063711-Lindenbergia_philippensis-flower
HandroanthusImpetiginosus_PIN15235.1
OleaEuropaea_Oeu002829.2
CoffeaCanephora_Cc10_g02640
Daucuscarota_DCAR_022476
ManihotEsculenta_Manes.05G157600.1.p
ManihotEsculenta_Manes.18G022700.1.p
ManihotEsculenta_Manes.08G007100.1.p
PopulusTrichocarpa_Potri.002G034100.1
PopulusTrichocarpa_Potri.005G229000.1
GossypiumRaimondii_KJB12032
TheobromaCacao_Thecc1EG034072t1
HelianthusAnnuus_OTF90616.1
HelianthusAnnuus_OTG16219.1
ArabidopsisThaliana_AT1G19580.1
CitrusSinensis_orange1.1g024224m
GlycineMax_Glyma.04G030800.1.p
GlycineMax_Glyma.06G030700.1.p
GlycineMax_Glyma.04G030700.1.p
ArabidopsisThaliana_AT1G47260.1
IpomoeaNil_INIL01g25494.t1
IpomoeaNil_INIL14g41359.t1
SolanumTuberosum_PGSC0003DMT400009474
PrunusPersica_Prupe.1G376900.1.p
VitisVinifera_GSVIVT01009735001
StrigaAsiatica_TRINITY_DN2411_c0_g1_i4
StrigaAsiatica_TRINITY_DN2411_c0_g1_i2
StrigaAsiatica_TRINITY_DN2411_c0_g1_i13
StrigaAsiatica_TRINITY_DN2411_c0_g1_i8
StrigaAsiatica_BKCP01002224.1_cds_GER27968.1_3502
StrigaAsiatica_BKCP01002224.1_cds_GER27965.1_3499
StrigaAsiatica_SGA_v2.0_scaffold51G18831
StrigaAsiatica_BKCP01008404.1_cds_GER49042.1_24576
StrigaAsiatica_TRINITY_DN2411_c0_g1_i11
Aeginetiaindica_TRINITY_DN2052_c0_g1_i2
Aeginetiaindica_TRINITY_DN2052_c0_g1_i1
ChristisoniaKwangtungensis_TRINITY_DN73915_c0_g1_i2
ChristisoniaKwangtungensis_TRINITY_DN73915_c0_g1_i1
Escobedia_TRINITY_DN8246_c0_g1_i9
Escobedia_TRINITY_DN8246_c0_g1_i8
CastillejaMiniata_TRINITY_DN8903_c0_g1_i1
CastillejaMiniata_TRINITY_DN8903_c0_g1_i2
CastillejaMiniata_TRINITY_DN8903_c0_g1_i3
Pedicularis_TRINITY_DN28_c1_g1_i1
Phtheirospermum_BMAC01000810.1_cds_GFQ03231.1_24665
ConopholisAmericana_scaffold-FAMO-2095685-Conopholis_americana
OrobancheCumana_GWHTBHPV010775
OrobancheCumana_GWHTBHPV010776
PhelipancheAegyptiaca_GWHTBHPU045598
LindenbergiaPhilippensis.09G154900.1
LindenbergiaPhilippensis_scaffold-WUZV-2063096-Lindenbergia_philippensis-flower
SesamumIndicum_SIN_1022199
HandroanthusImpetiginosus_PIN15778.1
MimulusGuttatus_Migut.H02532.1.p
OleaEuropaea_Oeu012313.1
IpomoeaNil_INIL11g26518.t1
CuscutaAustralis_C083N0027E0.1
SolanumTuberosum_PGSC0003DMT400009047
SolanumTuberosum_PGSC0003DMT400040041
HelianthusAnnuus_OTF93232.1
HelianthusAnnuus_OTF95367.1
CoffeaCanephora_Cc07_g09790
GossypiumRaimondii_KJB43468
GossypiumRaimondii_KJB43470
GossypiumRaimondii_KJB43471
GossypiumRaimondii_KJB43469
GossypiumRaimondii_KJB17751
GossypiumRaimondii_KJB17752
TheobromaCacao_Thecc1EG000346t1
ArabidopsisThaliana_AT5G66510.2
PopulusTrichocarpa_Potri.005G120800.1
CitrusSinensis_orange1.1g024153m
GlycineMax_Glyma.01G172300.1.p
GlycineMax_Glyma.11G070900.1.p
PrunusPersica_Prupe.7G157500.1.p
VitisVinifera_GSVIVT01018929001
scaffold-PHOQ-2072938-Orobanche_fasciculata-stem
Actinidiaeriantha_DTZ79_01g14860
Actinidiaeriantha_DTZ79_09g07320
Actinidiaeriantha_DTZ79_27g07000
VacciniumCorymbosum_VaccDscaff13-snap-gene-166.25-mRNA-1
VacciniumCorymbosum_VaccDscaff13-snap-gene-166.26-mRNA-1
Daucuscarota_DCAR_017304
Daucuscarota_DCAR_025955
AquilegiaCoerulea_PIA52718.1
CinnamomumMicranthum_RWR82425.1
CinnamomumMicranthum_RWR93625.1
AquilegiaCoerulea_PIA55796.1
AquilegiaCoerulea_PIA55797.1
OryzaSativa_LOC_Os07g44840.1
OryzaSativa_LOC_Os12g07220.1
SorghumBicolor_Sobic.002G395000.1
OryzaSativa_LOC_Os01g18070.1
SorghumBicolor_Sobic.003G135600.1
AmborellaTrichopoda_ERM94677.1
0.1
