# Supplementary material for: Altered Mitochondrial Respiration Is Associated With Loss of Nuclear‐Encoded OXPHOS Genes in Parasitic Broomrapes
Source: Ecol Evol. 2025 Jul 6;15(7):e71737. doi: 10.1002/ece3.71737 (PMC12230201; doi:10.1002/ece3.71737)
Supplement: Supplementary file 1 — Appendix S1. [file ECE3-15-e71737-s003.tgz › Orobanchaceae_O2K_Supplementary_v3/Data_S1/DataS1/CI/OG0005817.filter.fas.treefile.pdf]

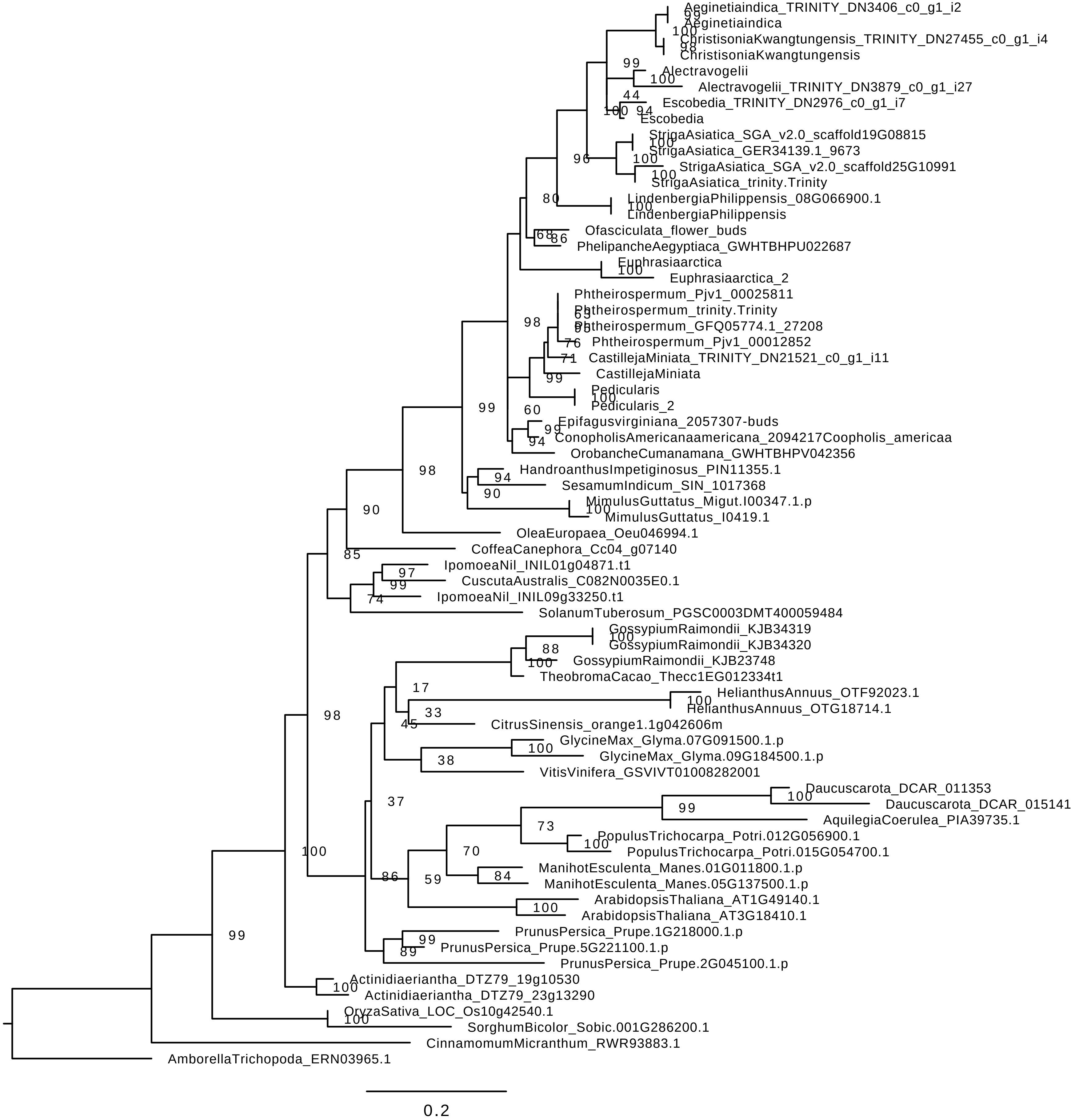
Aeginetiaindica_TRINITY_DN3406_c0_g1_i2
Aeginetiaindica
ChristisoniaKwangtungensis_TRINITY_DN27455_c0_g1_i4
ChristisoniaKwangtungensis
Alectravogelii
Alectravogelii_TRINITY_DN3879_c0_g1_i27
Escobedia_TRINITY_DN2976_c0_g1_i7
Escobedia
StrigaAsiatica_SGA_v2.0_scaffold19G08815
StrigaAsiatica_GER34139.1_9673
StrigaAsiatica_SGA_v2.0_scaffold25G10991
StrigaAsiatica_trinity.Trinity
LindenbergiaPhilippensis_08G066900.1
LindenbergiaPhilippensis
Ofasciculata_flower_buds
PhelipancheAegyptiaca_GWHTBHPU022687
Euphrasiaarctica
Euphrasiaarctica_2
Phtheirospermum_Pjv1_00025811
Phtheirospermum_trinity.Trinity
Phtheirospermum_GFQ05774.1_27208
Phtheirospermum_Pjv1_00012852
CastillejaMiniata_TRINITY_DN21521_c0_g1_i11
CastillejaMiniata
Pedicularis
Pedicularis_2
Epifagusvirginiana_2057307-buds
ConopholisAmericanaamericana_2094217Coopholis_americaa
OrobancheCumanamana_GWHTBHPV042356
HandroanthusImpetiginosus_PIN11355.1
SesamumIndicum_SIN_1017368
MimulusGuttatus_Migut.I00347.1.p
MimulusGuttatus_I0419.1
OleaEuropaea_Oeu046994.1
CoffeaCanephora_Cc04_g07140
IpomoeaNil_INIL01g04871.t1
CuscutaAustralis_C082N0035E0.1
IpomoeaNil_INIL09g33250.t1
SolanumTuberosum_PGSC0003DMT400059484
GossypiumRaimondii_KJB34319
GossypiumRaimondii_KJB34320
GossypiumRaimondii_KJB23748
TheobromaCacao_Thecc1EG012334t1
HelianthusAnnuus_OTF92023.1
HelianthusAnnuus_OTG18714.1
CitrusSinensis_orange1.1g042606m
GlycineMax_Glyma.07G091500.1.p
GlycineMax_Glyma.09G184500.1.p
VitisVinifera_GSVIVT01008282001
Daucuscarota_DCAR_011353
Daucuscarota_DCAR_015141
AquilegiaCoerulea_PIA39735.1
PopulusTrichocarpa_Potri.012G056900.1
PopulusTrichocarpa_Potri.015G054700.1
ManihotEsculenta_Manes.01G011800.1.p
ManihotEsculenta_Manes.05G137500.1.p
ArabidopsisThaliana_AT1G49140.1
ArabidopsisThaliana_AT3G18410.1
PrunusPersica_Prupe.1G218000.1.p
PrunusPersica_Prupe.5G221100.1.p
PrunusPersica_Prupe.2G045100.1.p
Actinidiaeriantha_DTZ79_19g10530
Actinidiaeriantha_DTZ79_23g13290
OryzaSativa_LOC_Os10g42540.1
SorghumBicolor_Sobic.001G286200.1
CinnamomumMicranthum_RWR93883.1
AmborellaTrichopoda_ERN03965.1
0.2
