# Supplementary material for: Altered Mitochondrial Respiration Is Associated With Loss of Nuclear‐Encoded OXPHOS Genes in Parasitic Broomrapes
Source: Ecol Evol. 2025 Jul 6;15(7):e71737. doi: 10.1002/ece3.71737 (PMC12230201; doi:10.1002/ece3.71737)
Supplement: Supplementary file 1 — Appendix S1. [file ECE3-15-e71737-s003.tgz › Orobanchaceae_O2K_Supplementary_v3/Data_S1/DataS1/CI/OG0007666.filter.fas.treefile.pdf]

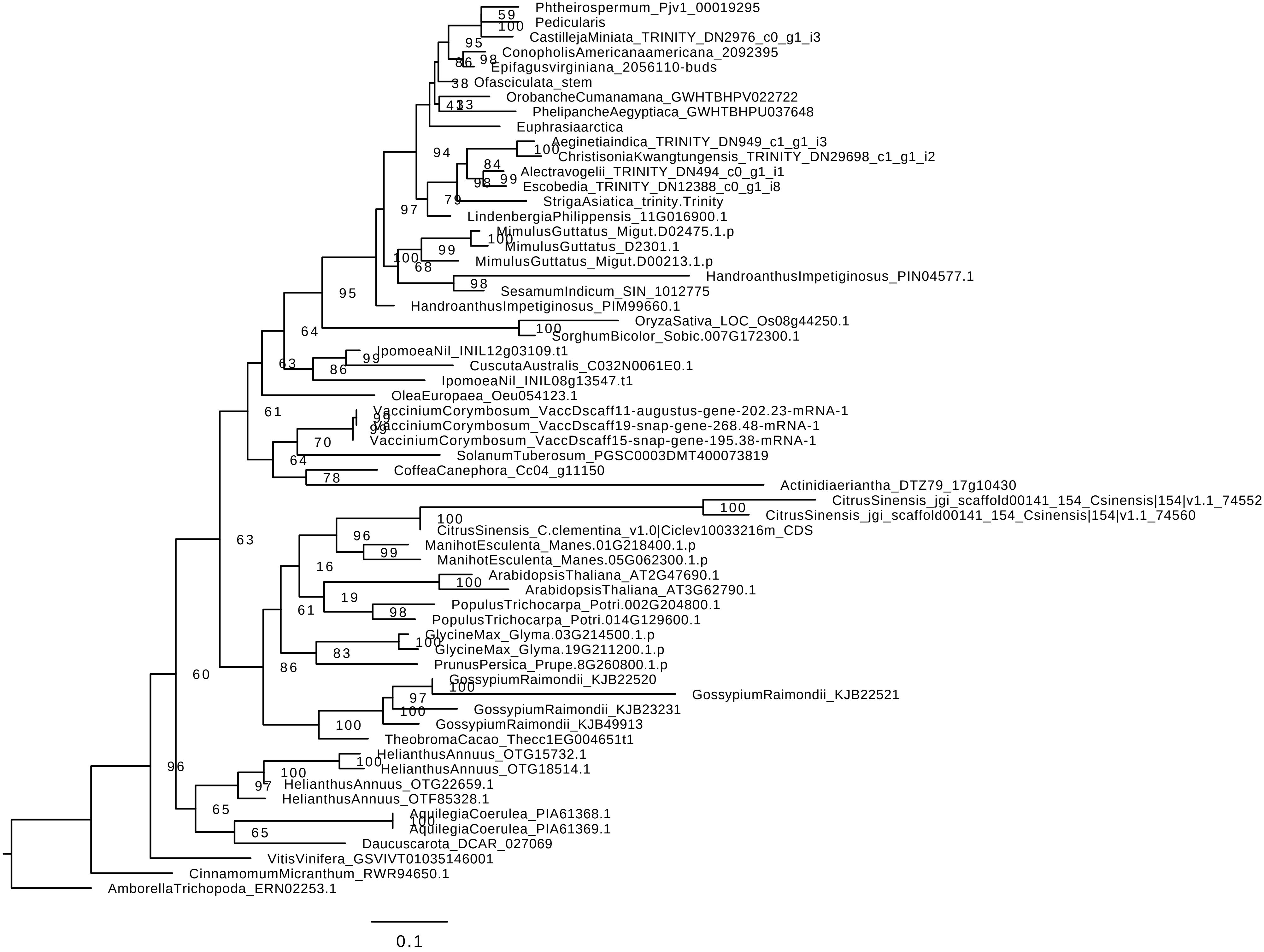
Phtheirospermum_Pjv1_00019295
Pedicularis
CastillejaMiniata_TRINITY_DN2976_c0_g1_i3
ConopholisAmericanaamericana_2092395
Epifagusvirginiana_2056110-buds
Ofasciculata_stem
OrobancheCumanamana_GWHTBHPV022722
PhelipancheAegyptiaca_GWHTBHPU037648
Euphrasiaarctica
Aeginetiaindica_TRINITY_DN949_c1_g1_i3
ChristisoniaKwangtungensis_TRINITY_DN29698_c1_g1_i2
Alectravogelii_TRINITY_DN494_c0_g1_i1
Escobedia_TRINITY_DN12388_c0_g1_i8
StrigaAsiatica_trinity.Trinity
LindenbergiaPhilippensis_11G016900.1
MimulusGuttatus_Migut.D02475.1.p
MimulusGuttatus_D2301.1
MimulusGuttatus_Migut.D00213.1.p
HandroanthusImpetiginosus_PIN04577.1
SesamumIndicum_SIN_1012775
HandroanthusImpetiginosus_PIM99660.1
OryzaSativa_LOC_Os08g44250.1
SorghumBicolor_Sobic.007G172300.1
IpomoeaNil_INIL12g03109.t1
CuscutaAustralis_C032N0061E0.1
IpomoeaNil_INIL08g13547.t1
OleaEuropaea_Oeu054123.1
VacciniumCorymbosum_VaccDscaff11-augustus-gene-202.23-mRNA-1
VacciniumCorymbosum_VaccDscaff19-snap-gene-268.48-mRNA-1
VacciniumCorymbosum_VaccDscaff15-snap-gene-195.38-mRNA-1
SolanumTuberosum_PGSC0003DMT400073819
CoffeaCanephora_Cc04_g11150
Actinidiaeriantha_DTZ79_17g10430
CitrusSinensis_jgi_scaffold00141_154_Csinensis|154|v1.1_74552
CitrusSinensis_jgi_scaffold00141_154_Csinensis|154|v1.1_74560
CitrusSinensis_C.clementina_v1.0|Ciclev10033216m_CDS
ManihotEsculenta_Manes.01G218400.1.p
ManihotEsculenta_Manes.05G062300.1.p
ArabidopsisThaliana_AT2G47690.1
ArabidopsisThaliana_AT3G62790.1
PopulusTrichocarpa_Potri.002G204800.1
PopulusTrichocarpa_Potri.014G129600.1
GlycineMax_Glyma.03G214500.1.p
GlycineMax_Glyma.19G211200.1.p
PrunusPersica_Prupe.8G260800.1.p
GossypiumRaimondii_KJB22520
GossypiumRaimondii_KJB22521
GossypiumRaimondii_KJB23231
GossypiumRaimondii_KJB49913
TheobromaCacao_Thecc1EG004651t1
HelianthusAnnuus_OTG15732.1
HelianthusAnnuus_OTG18514.1
HelianthusAnnuus_OTG22659.1
HelianthusAnnuus_OTF85328.1
AquilegiaCoerulea_PIA61368.1
AquilegiaCoerulea_PIA61369.1
Daucuscarota_DCAR_027069
VitisVinifera_GSVIVT01035146001
CinnamomumMicranthum_RWR94650.1
AmborellaTrichopoda_ERN02253.1
0.1
