# Supplementary material for: Altered Mitochondrial Respiration Is Associated With Loss of Nuclear‐Encoded OXPHOS Genes in Parasitic Broomrapes
Source: Ecol Evol. 2025 Jul 6;15(7):e71737. doi: 10.1002/ece3.71737 (PMC12230201; doi:10.1002/ece3.71737)
Supplement: Supplementary file 1 — Appendix S1. [file ECE3-15-e71737-s003.tgz › Orobanchaceae_O2K_Supplementary_v3/Data_S1/DataS1/CI/OG0005370.filter.fas.treefile.pdf]

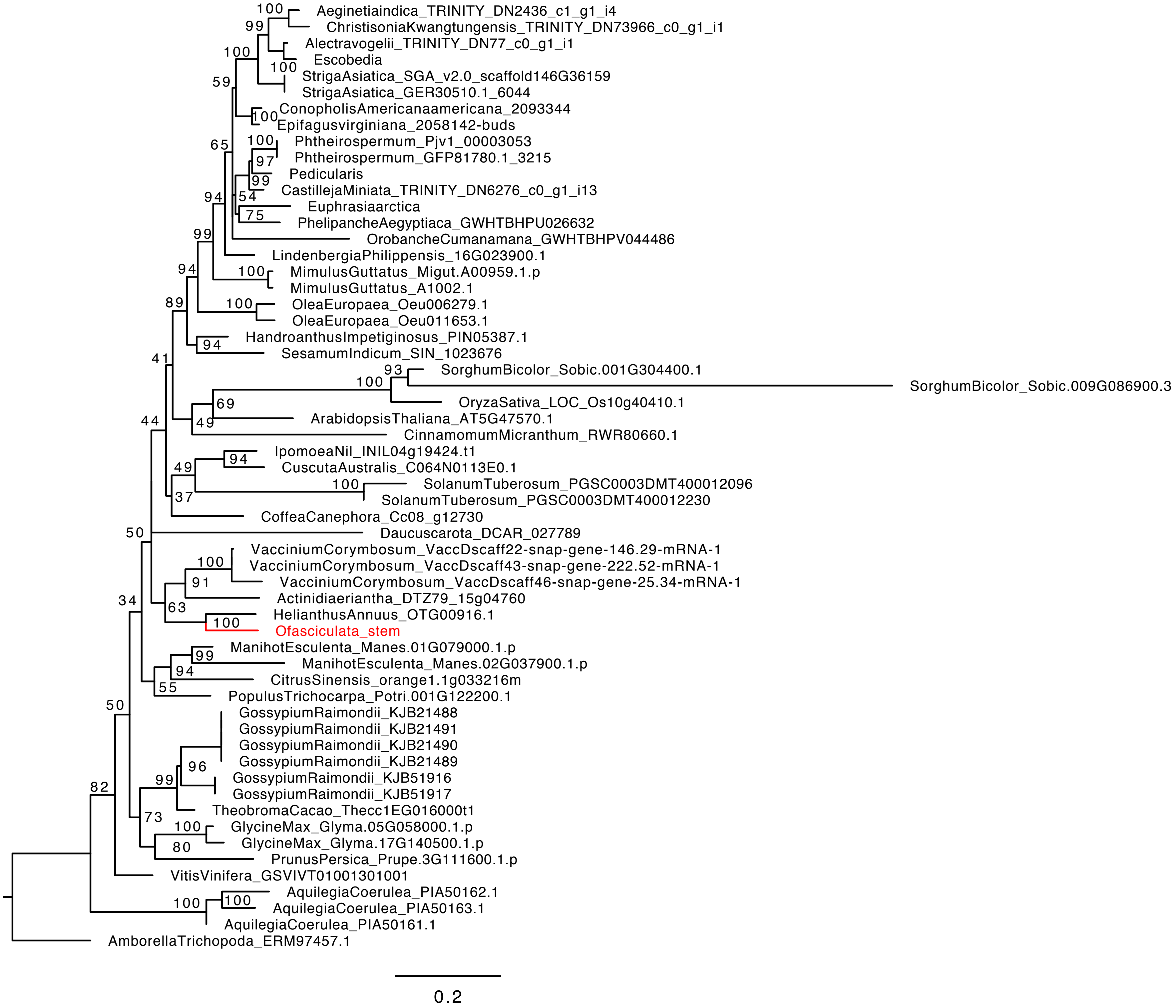
Aeginetiaindica_TRINITY_DN2436_c1_g1_i4
ChristisoniaKwangtungensis_TRINITY_DN73966_c0_g1_i1
Alectravogelii_TRINITY_DN77_c0_g1_i1
Escobedia
StrigaAsiatica_SGA_v2.0_scaffold146G36159
StrigaAsiatica_GER30510.1_6044
ConopholisAmericanaamericana_2093344
Epifagusvirginiana_2058142-buds
Phtheirospermum_Pjv1_00003053
Phtheirospermum_GFP81780.1_3215
Pedicularis
CastillejaMiniata_TRINITY_DN6276_c0_g1_i13
Euphrasiaarctica
PhelipancheAegyptiaca_GWHTBHPU026632
OrobancheCumanamana_GWHTBHPV044486
LindenbergiaPhilippensis_16G023900.1
MimulusGuttatus_Migut.A00959.1.p
MimulusGuttatus_A1002.1
OleaEuropaea_Oeu006279.1
OleaEuropaea_Oeu011653.1
HandroanthusImpetiginosus_PIN05387.1
SesamumIndicum_SIN_1023676
SorghumBicolor_Sobic.001G304400.1
SorghumBicolor_Sobic.009G086900.3
OryzaSativa_LOC_Os10g40410.1
ArabidopsisThaliana_AT5G47570.1
CinnamomumMicranthum_RWR80660.1
IpomoeaNil_INIL04g19424.t1
CuscutaAustralis_C064N0113E0.1
SolanumTuberosum_PGSC0003DMT400012096
SolanumTuberosum_PGSC0003DMT400012230
CoffeaCanephora_Cc08_g12730
Daucuscarota_DCAR_027789
VacciniumCorymbosum_VaccDscaff22-snap-gene-146.29-mRNA-1
VacciniumCorymbosum_VaccDscaff43-snap-gene-222.52-mRNA-1
VacciniumCorymbosum_VaccDscaff46-snap-gene-25.34-mRNA-1
Actinidiaeriantha_DTZ79_15g04760
HelianthusAnnuus_OTG00916.1
Ofasciculata_stem
ManihotEsculenta_Manes.01G079000.1.p
ManihotEsculenta_Manes.02G037900.1.p
CitrusSinensis_orange1.1g033216m
PopulusTrichocarpa_Potri.001G122200.1
GossypiumRaimondii_KJB21488
GossypiumRaimondii_KJB21491
GossypiumRaimondii_KJB21490
GossypiumRaimondii_KJB21489
GossypiumRaimondii_KJB51916
GossypiumRaimondii_KJB51917
TheobromaCacao_Thecc1EG016000t1
GlycineMax_Glyma.05G058000.1.p
GlycineMax_Glyma.17G140500.1.p
PrunusPersica_Prupe.3G111600.1.p
VitisVinifera_GSVIVT01001301001
AquilegiaCoerulea_PIA50162.1
AquilegiaCoerulea_PIA50163.1
AquilegiaCoerulea_PIA50161.1
AmborellaTrichopoda_ERM97457.1
0.2
