# Supplementary material for: Altered Mitochondrial Respiration Is Associated With Loss of Nuclear‐Encoded OXPHOS Genes in Parasitic Broomrapes
Source: Ecol Evol. 2025 Jul 6;15(7):e71737. doi: 10.1002/ece3.71737 (PMC12230201; doi:10.1002/ece3.71737)
Supplement: Supplementary file 1 — Appendix S1. [file ECE3-15-e71737-s003.tgz › Orobanchaceae_O2K_Supplementary_v3/Data_S1/DataS1/CI/OG0005288.filter.fas.treefile.pdf]

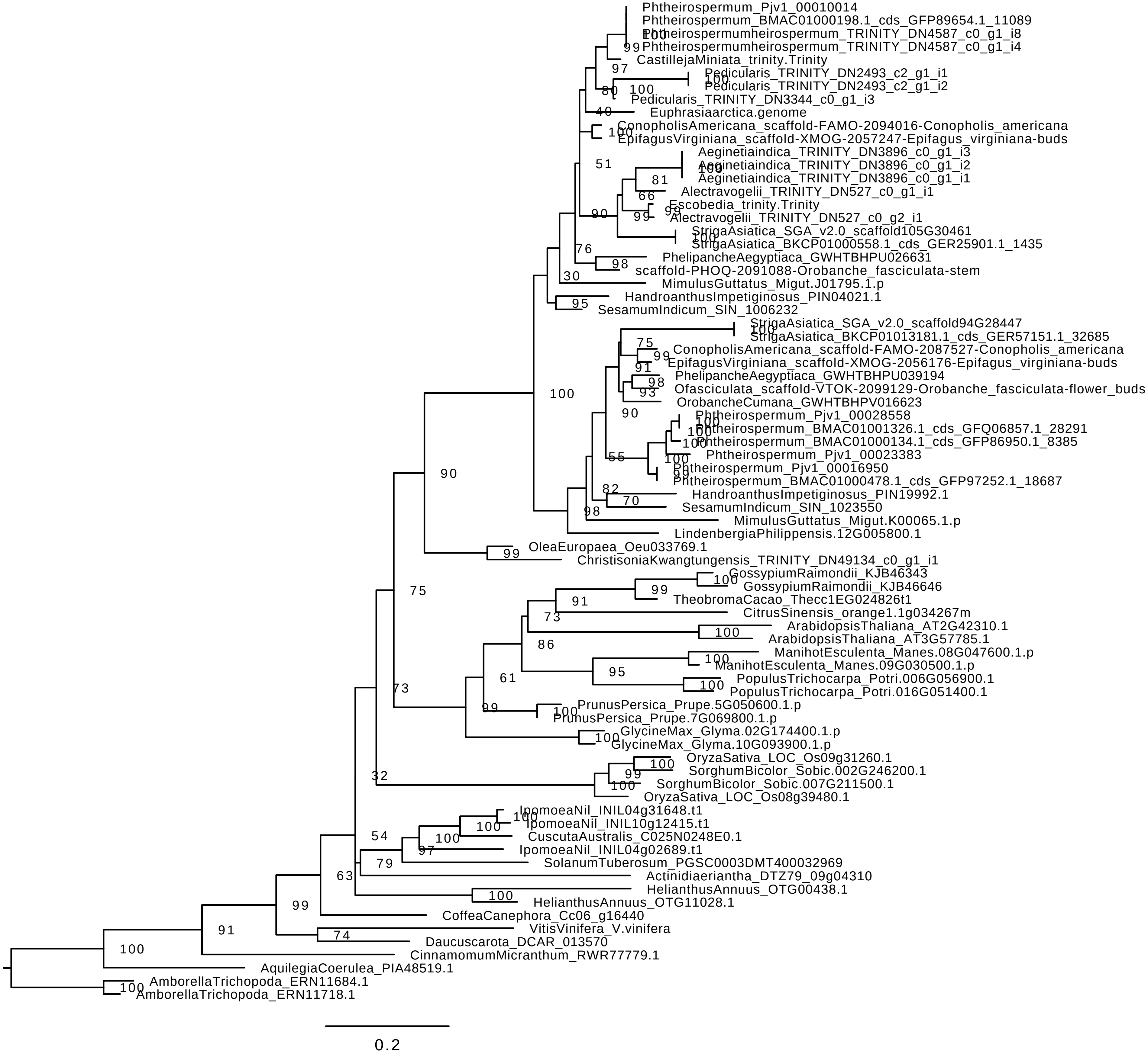
Phtheirospermum_Pjv1_00010014
Phtheirospermum_BMAC01000198.1_cds_GFP89654.1_11089
Phtheirospermumheirospermum_TRINITY_DN4587_c0_g1_i8
Phtheirospermumheirospermum_TRINITY_DN4587_c0_g1_i4
CastillejaMiniata_trinity.Trinity
Pedicularis_TRINITY_DN2493_c2_g1_i1
Pedicularis_TRINITY_DN2493_c2_g1_i2
Pedicularis_TRINITY_DN3344_c0_g1_i3
Euphrasiaarctica.genome
ConopholisAmericana_scaffold-FAMO-2094016-Conopholis_americana
EpifagusVirginiana_scaffold-XMOG-2057247-Epifagus_virginiana-buds
Aeginetiaindica_TRINITY_DN3896_c0_g1_i3
Aeginetiaindica_TRINITY_DN3896_c0_g1_i2
Aeginetiaindica_TRINITY_DN3896_c0_g1_i1
Alectravogelii_TRINITY_DN527_c0_g1_i1
Escobedia_trinity.Trinity
Alectravogelii_TRINITY_DN527_c0_g2_i1
StrigaAsiatica_SGA_v2.0_scaffold105G30461
StrigaAsiatica_BKCP01000558.1_cds_GER25901.1_1435
PhelipancheAegyptiaca_GWHTBHPU026631
scaffold-PHOQ-2091088-Orobanche_fasciculata-stem
MimulusGuttatus_Migut.J01795.1.p
HandroanthusImpetiginosus_PIN04021.1
SesamumIndicum_SIN_1006232
StrigaAsiatica_SGA_v2.0_scaffold94G28447
StrigaAsiatica_BKCP01013181.1_cds_GER57151.1_32685
ConopholisAmericana_scaffold-FAMO-2087527-Conopholis_americana
EpifagusVirginiana_scaffold-XMOG-2056176-Epifagus_virginiana-buds
PhelipancheAegyptiaca_GWHTBHPU039194
Ofasciculata_scaffold-VTOK-2099129-Orobanche_fasciculata-flower_buds
OrobancheCumana_GWHTBHPV016623
Phtheirospermum_Pjv1_00028558
Phtheirospermum_BMAC01001326.1_cds_GFQ06857.1_28291
Phtheirospermum_BMAC01000134.1_cds_GFP86950.1_8385
Phtheirospermum_Pjv1_00023383
Phtheirospermum_Pjv1_00016950
Phtheirospermum_BMAC01000478.1_cds_GFP97252.1_18687
HandroanthusImpetiginosus_PIN19992.1
SesamumIndicum_SIN_1023550
MimulusGuttatus_Migut.K00065.1.p
LindenbergiaPhilippensis.12G005800.1
OleaEuropaea_Oeu033769.1
ChristisoniaKwangtungensis_TRINITY_DN49134_c0_g1_i1
GossypiumRaimondii_KJB46343
GossypiumRaimondii_KJB46646
TheobromaCacao_Thecc1EG024826t1
CitrusSinensis_orange1.1g034267m
ArabidopsisThaliana_AT2G42310.1
ArabidopsisThaliana_AT3G57785.1
ManihotEsculenta_Manes.08G047600.1.p
ManihotEsculenta_Manes.09G030500.1.p
PopulusTrichocarpa_Potri.006G056900.1
PopulusTrichocarpa_Potri.016G051400.1
PrunusPersica_Prupe.5G050600.1.p
PrunusPersica_Prupe.7G069800.1.p
GlycineMax_Glyma.02G174400.1.p
GlycineMax_Glyma.10G093900.1.p
OryzaSativa_LOC_Os09g31260.1
SorghumBicolor_Sobic.002G246200.1
SorghumBicolor_Sobic.007G211500.1
OryzaSativa_LOC_Os08g39480.1
IpomoeaNil_INIL04g31648.t1
IpomoeaNil_INIL10g12415.t1
CuscutaAustralis_C025N0248E0.1
IpomoeaNil_INIL04g02689.t1
SolanumTuberosum_PGSC0003DMT400032969
Actinidiaeriantha_DTZ79_09g04310
HelianthusAnnuus_OTG00438.1
HelianthusAnnuus_OTG11028.1
CoffeaCanephora_Cc06_g16440
VitisVinifera_V.vinifera
Daucuscarota_DCAR_013570
CinnamomumMicranthum_RWR77779.1
AquilegiaCoerulea_PIA48519.1
AmborellaTrichopoda_ERN11684.1
AmborellaTrichopoda_ERN11718.1
0.2
