# Supplementary material for: Altered Mitochondrial Respiration Is Associated With Loss of Nuclear‐Encoded OXPHOS Genes in Parasitic Broomrapes
Source: Ecol Evol. 2025 Jul 6;15(7):e71737. doi: 10.1002/ece3.71737 (PMC12230201; doi:10.1002/ece3.71737)
Supplement: Supplementary file 1 — Appendix S1. [file ECE3-15-e71737-s003.tgz › Orobanchaceae_O2K_Supplementary_v3/Data_S1/DataS1/CI/OG0007811.filter.fas.treefile.pdf]

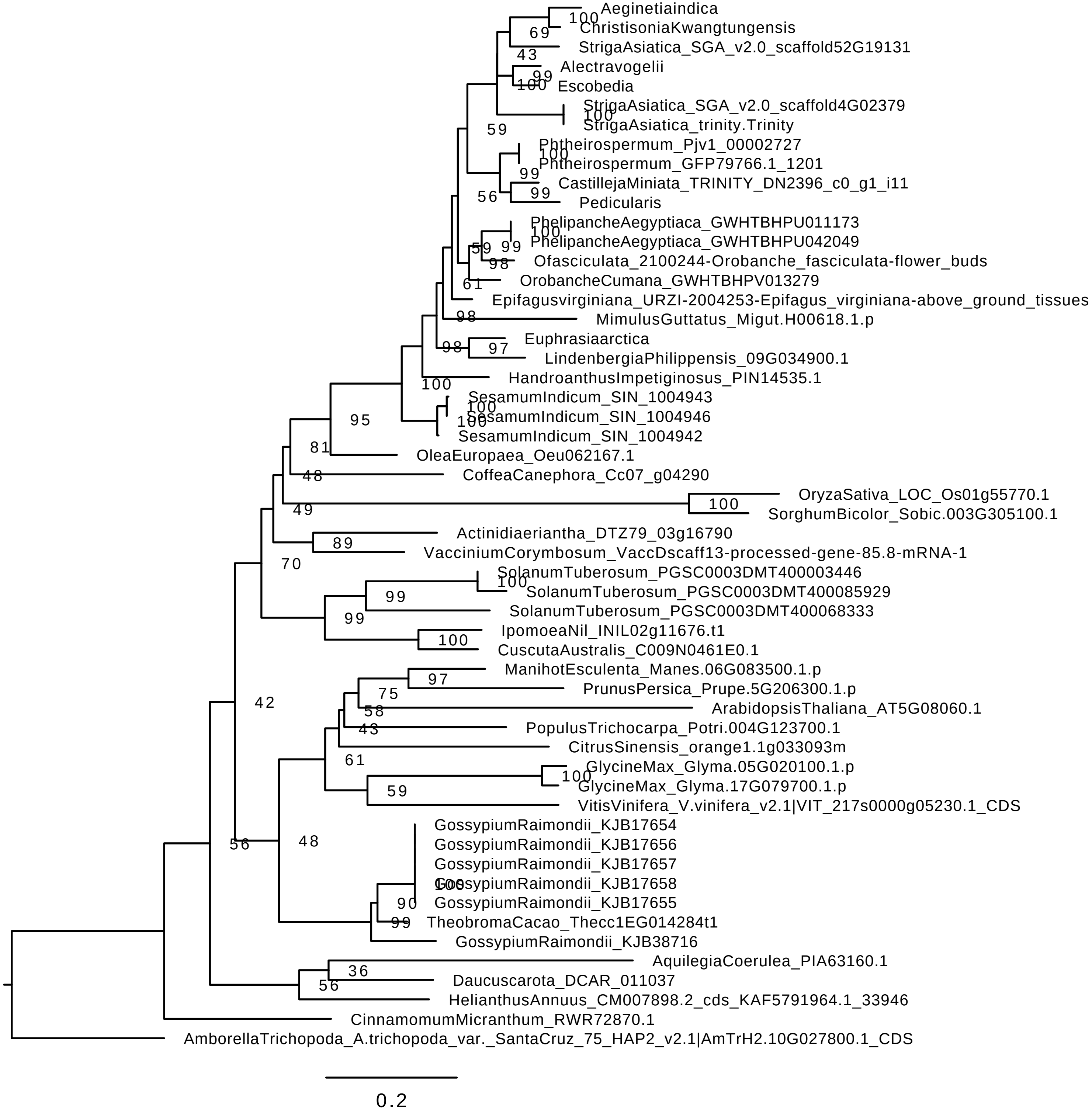
Aeginetiaindica
ChristisoniaKwangtungensis
StrigaAsiatica_SGA_v2.0_scaffold52G19131
Alectravogelii
Escobedia
StrigaAsiatica_SGA_v2.0_scaffold4G02379
StrigaAsiatica_trinity.Trinity
Phtheirospermum_Pjv1_00002727
Phtheirospermum_GFP79766.1_1201
CastillejaMiniata_TRINITY_DN2396_c0_g1_i11
Pedicularis
PhelipancheAegyptiaca_GWHTBHPU011173
PhelipancheAegyptiaca_GWHTBHPU042049
Ofasciculata_2100244-Orobanche_fasciculata-flower_buds
OrobancheCumana_GWHTBHPV013279
Epifagusvirginiana_URZI-2004253-Epifagus_virginiana-above_ground_tissues
MimulusGuttatus_Migut.H00618.1.p
Euphrasiaarctica
LindenbergiaPhilippensis_09G034900.1
HandroanthusImpetiginosus_PIN14535.1
SesamumIndicum_SIN_1004943
SesamumIndicum_SIN_1004946
SesamumIndicum_SIN_1004942
OleaEuropaea_Oeu062167.1
CoffeaCanephora_Cc07_g04290
OryzaSativa_LOC_Os01g55770.1
SorghumBicolor_Sobic.003G305100.1
Actinidiaeriantha_DTZ79_03g16790
VacciniumCorymbosum_VaccDscaff13-processed-gene-85.8-mRNA-1
SolanumTuberosum_PGSC0003DMT400003446
SolanumTuberosum_PGSC0003DMT400085929
SolanumTuberosum_PGSC0003DMT400068333
IpomoeaNil_INIL02g11676.t1
CuscutaAustralis_C009N0461E0.1
ManihotEsculenta_Manes.06G083500.1.p
PrunusPersica_Prupe.5G206300.1.p
ArabidopsisThaliana_AT5G08060.1
PopulusTrichocarpa_Potri.004G123700.1
CitrusSinensis_orange1.1g033093m
GlycineMax_Glyma.05G020100.1.p
GlycineMax_Glyma.17G079700.1.p
VitisVinifera_V.vinifera_v2.1|VIT_217s0000g05230.1_CDS
GossypiumRaimondii_KJB17654
GossypiumRaimondii_KJB17656
GossypiumRaimondii_KJB17657
GossypiumRaimondii_KJB17658
GossypiumRaimondii_KJB17655
TheobromaCacao_Thecc1EG014284t1
GossypiumRaimondii_KJB38716
AquilegiaCoerulea_PIA63160.1
Daucuscarota_DCAR_011037
HelianthusAnnuus_CM007898.2_cds_KAF5791964.1_33946
CinnamomumMicranthum_RWR72870.1
AmborellaTrichopoda_A.trichopoda_var._SantaCruz_75_HAP2_v2.1|AmTrH2.10G027800.1_CDS
0.2
