# Supplementary material for: Altered Mitochondrial Respiration Is Associated With Loss of Nuclear‐Encoded OXPHOS Genes in Parasitic Broomrapes
Source: Ecol Evol. 2025 Jul 6;15(7):e71737. doi: 10.1002/ece3.71737 (PMC12230201; doi:10.1002/ece3.71737)
Supplement: Supplementary file 1 — Appendix S1. [file ECE3-15-e71737-s003.tgz › Orobanchaceae_O2K_Supplementary_v3/Data_S1/DataS1/CI/OG0007321.filter.fas.treefile.pdf]

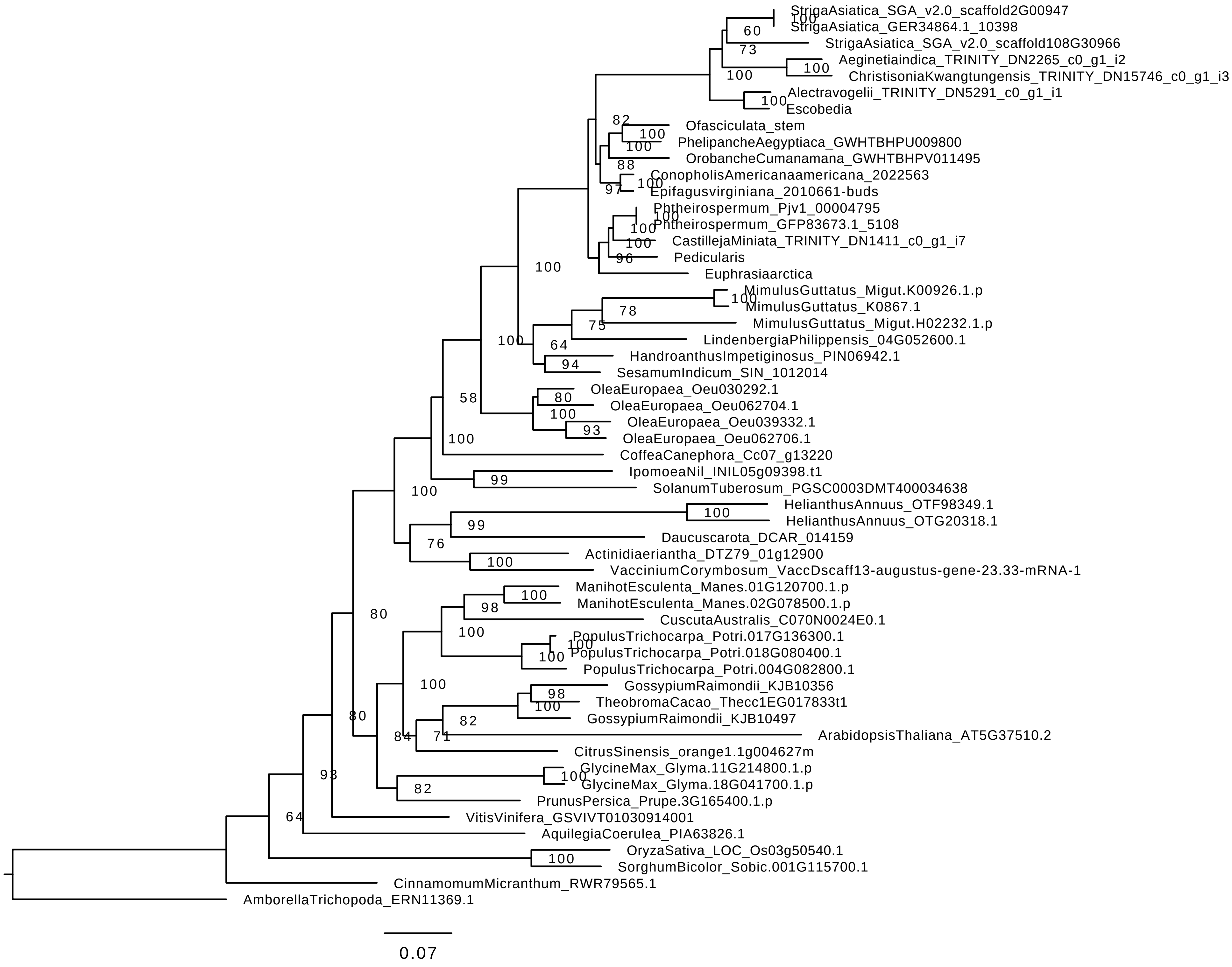
StrigaAsiatica_SGA_v2.0_scaffold2G00947
StrigaAsiatica_GER34864.1_10398
StrigaAsiatica_SGA_v2.0_scaffold108G30966
Aeginetiaindica_TRINITY_DN2265_c0_g1_i2
ChristisoniaKwangtungensis_TRINITY_DN15746_c0_g1_i3
Alectravogelii_TRINITY_DN5291_c0_g1_i1
Escobedia
Ofasciculata_stem
PhelipancheAegyptiaca_GWHTBHPU009800
OrobancheCumanamana_GWHTBHPV011495
ConopholisAmericanaamericana_2022563
Epifagusvirginiana_2010661-buds
Phtheirospermum_Pjv1_00004795
Phtheirospermum_GFP83673.1_5108
CastillejaMiniata_TRINITY_DN1411_c0_g1_i7
Pedicularis
Euphrasiaarctica
MimulusGuttatus_Migut.K00926.1.p
MimulusGuttatus_K0867.1
MimulusGuttatus_Migut.H02232.1.p
LindenbergiaPhilippensis_04G052600.1
HandroanthusImpetiginosus_PIN06942.1
SesamumIndicum_SIN_1012014
OleaEuropaea_Oeu030292.1
OleaEuropaea_Oeu062704.1
OleaEuropaea_Oeu039332.1
OleaEuropaea_Oeu062706.1
CoffeaCanephora_Cc07_g13220
IpomoeaNil_INIL05g09398.t1
SolanumTuberosum_PGSC0003DMT400034638
HelianthusAnnuus_OTF98349.1
HelianthusAnnuus_OTG20318.1
Daucuscarota_DCAR_014159
Actinidiaeriantha_DTZ79_01g12900
VacciniumCorymbosum_VaccDscaff13-augustus-gene-23.33-mRNA-1
ManihotEsculenta_Manes.01G120700.1.p
ManihotEsculenta_Manes.02G078500.1.p
CuscutaAustralis_C070N0024E0.1
PopulusTrichocarpa_Potri.017G136300.1
PopulusTrichocarpa_Potri.018G080400.1
PopulusTrichocarpa_Potri.004G082800.1
GossypiumRaimondii_KJB10356
TheobromaCacao_Thecc1EG017833t1
GossypiumRaimondii_KJB10497
ArabidopsisThaliana_AT5G37510.2
CitrusSinensis_orange1.1g004627m
GlycineMax_Glyma.11G214800.1.p
GlycineMax_Glyma.18G041700.1.p
PrunusPersica_Prupe.3G165400.1.p
VitisVinifera_GSVIVT01030914001
AquilegiaCoerulea_PIA63826.1
OryzaSativa_LOC_Os03g50540.1
SorghumBicolor_Sobic.001G115700.1
CinnamomumMicranthum_RWR79565.1
AmborellaTrichopoda_ERN11369.1
0.07
