# Supplementary material for: Altered Mitochondrial Respiration Is Associated With Loss of Nuclear‐Encoded OXPHOS Genes in Parasitic Broomrapes
Source: Ecol Evol. 2025 Jul 6;15(7):e71737. doi: 10.1002/ece3.71737 (PMC12230201; doi:10.1002/ece3.71737)
Supplement: Supplementary file 1 — Appendix S1. [file ECE3-15-e71737-s003.tgz › Orobanchaceae_O2K_Supplementary_v3/Data_S1/DataS1/CI/OG0007274.filter.fas.treefile.pdf]

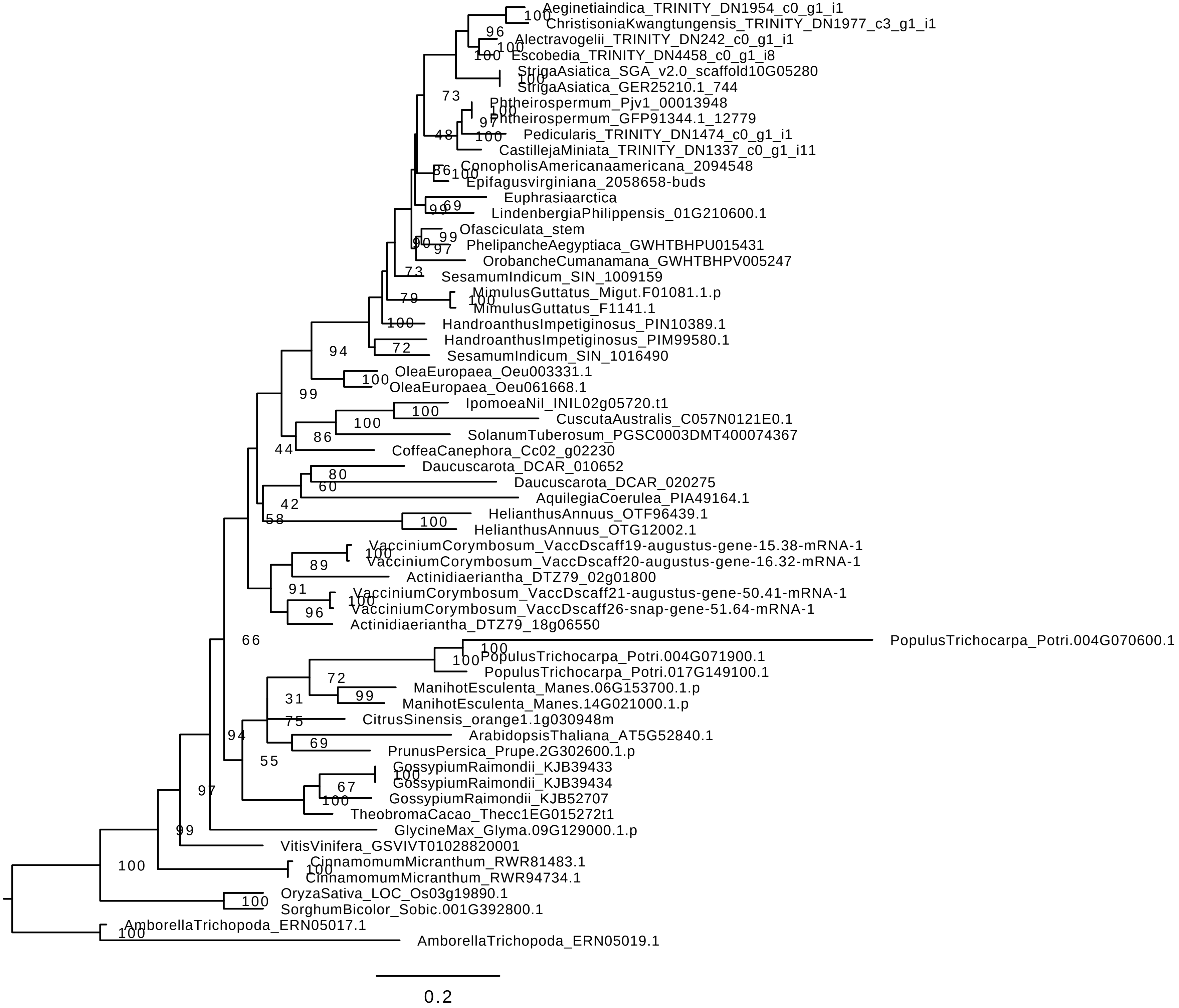
Aeginetiaindica_TRINITY_DN1954_c0_g1_i1
ChristisoniaKwangtungensis_TRINITY_DN1977_c3_g1_i1
Alectravogelii_TRINITY_DN242_c0_g1_i1
Escobedia_TRINITY_DN4458_c0_g1_i8
StrigaAsiatica_SGA_v2.0_scaffold10G05280
StrigaAsiatica_GER25210.1_744
Phtheirospermum_Pjv1_00013948
Phtheirospermum_GFP91344.1_12779
Pedicularis_TRINITY_DN1474_c0_g1_i1
CastillejaMiniata_TRINITY_DN1337_c0_g1_i11
ConopholisAmericanaamericana_2094548
Epifagusvirginiana_2058658-buds
Euphrasiaarctica
LindenbergiaPhilippensis_01G210600.1
Ofasciculata_stem
PhelipancheAegyptiaca_GWHTBHPU015431
OrobancheCumanamana_GWHTBHPV005247
SesamumIndicum_SIN_1009159
MimulusGuttatus_Migut.F01081.1.p
MimulusGuttatus_F1141.1
HandroanthusImpetiginosus_PIN10389.1
HandroanthusImpetiginosus_PIM99580.1
SesamumIndicum_SIN_1016490
OleaEuropaea_Oeu003331.1
OleaEuropaea_Oeu061668.1
IpomoeaNil_INIL02g05720.t1
CuscutaAustralis_C057N0121E0.1
SolanumTuberosum_PGSC0003DMT400074367
CoffeaCanephora_Cc02_g02230
Daucuscarota_DCAR_010652
Daucuscarota_DCAR_020275
AquilegiaCoerulea_PIA49164.1
HelianthusAnnuus_OTF96439.1
HelianthusAnnuus_OTG12002.1
VacciniumCorymbosum_VaccDscaff19-augustus-gene-15.38-mRNA-1
VacciniumCorymbosum_VaccDscaff20-augustus-gene-16.32-mRNA-1
Actinidiaeriantha_DTZ79_02g01800
VacciniumCorymbosum_VaccDscaff21-augustus-gene-50.41-mRNA-1
VacciniumCorymbosum_VaccDscaff26-snap-gene-51.64-mRNA-1
Actinidiaeriantha_DTZ79_18g06550
PopulusTrichocarpa_Potri.004G070600.1
PopulusTrichocarpa_Potri.004G071900.1
PopulusTrichocarpa_Potri.017G149100.1
ManihotEsculenta_Manes.06G153700.1.p
ManihotEsculenta_Manes.14G021000.1.p
CitrusSinensis_orange1.1g030948m
ArabidopsisThaliana_AT5G52840.1
PrunusPersica_Prupe.2G302600.1.p
GossypiumRaimondii_KJB39433
GossypiumRaimondii_KJB39434
GossypiumRaimondii_KJB52707
TheobromaCacao_Thecc1EG015272t1
GlycineMax_Glyma.09G129000.1.p
VitisVinifera_GSVIVT01028820001
CinnamomumMicranthum_RWR81483.1
CinnamomumMicranthum_RWR94734.1
OryzaSativa_LOC_Os03g19890.1
SorghumBicolor_Sobic.001G392800.1
AmborellaTrichopoda_ERN05017.1
AmborellaTrichopoda_ERN05019.1
0.2
