# Supplementary material for: Altered Mitochondrial Respiration Is Associated With Loss of Nuclear‐Encoded OXPHOS Genes in Parasitic Broomrapes
Source: Ecol Evol. 2025 Jul 6;15(7):e71737. doi: 10.1002/ece3.71737 (PMC12230201; doi:10.1002/ece3.71737)
Supplement: Supplementary file 1 — Appendix S1. [file ECE3-15-e71737-s003.tgz › Orobanchaceae_O2K_Supplementary_v3/Data_S1/DataS1/CI/OG0006125.filter.fas.treefile.pdf]

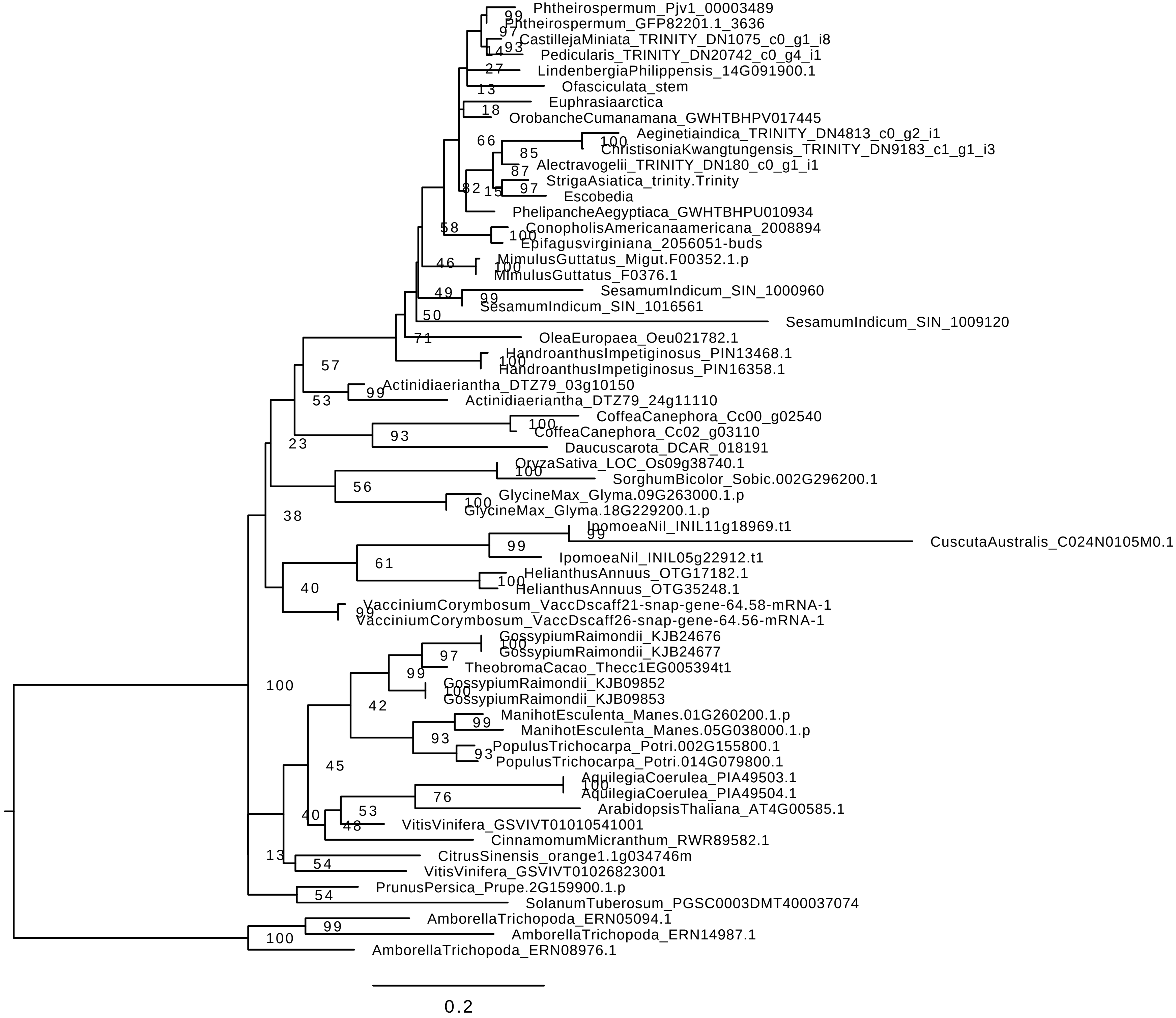
Phtheirospermum_Pjv1_00003489
Phtheirospermum_GFP82201.1_3636
CastillejaMiniata_TRINITY_DN1075_c0_g1_i8
Pedicularis_TRINITY_DN20742_c0_g4_i1
LindenbergiaPhilippensis_14G091900.1
Ofasciculata_stem
Euphrasiaarctica
OrobancheCumanamana_GWHTBHPV017445
Aeginetiaindica_TRINITY_DN4813_c0_g2_i1
ChristisoniaKwangtungensis_TRINITY_DN9183_c1_g1_i3
Alectravogelii_TRINITY_DN180_c0_g1_i1
StrigaAsiatica_trinity.Trinity
Escobedia
PhelipancheAegyptiaca_GWHTBHPU010934
ConopholisAmericanaamericana_2008894
Epifagusvirginiana_2056051-buds
MimulusGuttatus_Migut.F00352.1.p
MimulusGuttatus_F0376.1
SesamumIndicum_SIN_1000960
SesamumIndicum_SIN_1016561
SesamumIndicum_SIN_1009120
OleaEuropaea_Oeu021782.1
HandroanthusImpetiginosus_PIN13468.1
HandroanthusImpetiginosus_PIN16358.1
Actinidiaeriantha_DTZ79_03g10150
Actinidiaeriantha_DTZ79_24g11110
CoffeaCanephora_Cc00_g02540
CoffeaCanephora_Cc02_g03110
Daucuscarota_DCAR_018191
OryzaSativa_LOC_Os09g38740.1
SorghumBicolor_Sobic.002G296200.1
GlycineMax_Glyma.09G263000.1.p
GlycineMax_Glyma.18G229200.1.p
IpomoeaNil_INIL11g18969.t1
CuscutaAustralis_C024N0105M0.1
IpomoeaNil_INIL05g22912.t1
HelianthusAnnuus_OTG17182.1
HelianthusAnnuus_OTG35248.1
VacciniumCorymbosum_VaccDscaff21-snap-gene-64.58-mRNA-1
VacciniumCorymbosum_VaccDscaff26-snap-gene-64.56-mRNA-1
GossypiumRaimondii_KJB24676
GossypiumRaimondii_KJB24677
TheobromaCacao_Thecc1EG005394t1
GossypiumRaimondii_KJB09852
GossypiumRaimondii_KJB09853
ManihotEsculenta_Manes.01G260200.1.p
ManihotEsculenta_Manes.05G038000.1.p
PopulusTrichocarpa_Potri.002G155800.1
PopulusTrichocarpa_Potri.014G079800.1
AquilegiaCoerulea_PIA49503.1
AquilegiaCoerulea_PIA49504.1
ArabidopsisThaliana_AT4G00585.1
VitisVinifera_GSVIVT01010541001
CinnamomumMicranthum_RWR89582.1
CitrusSinensis_orange1.1g034746m
VitisVinifera_GSVIVT01026823001
PrunusPersica_Prupe.2G159900.1.p
SolanumTuberosum_PGSC0003DMT400037074
AmborellaTrichopoda_ERN05094.1
AmborellaTrichopoda_ERN14987.1
AmborellaTrichopoda_ERN08976.1
0.2
