# Supplementary material for: Altered Mitochondrial Respiration Is Associated With Loss of Nuclear‐Encoded OXPHOS Genes in Parasitic Broomrapes
Source: Ecol Evol. 2025 Jul 6;15(7):e71737. doi: 10.1002/ece3.71737 (PMC12230201; doi:10.1002/ece3.71737)
Supplement: Supplementary file 1 — Appendix S1. [file ECE3-15-e71737-s003.tgz › Orobanchaceae_O2K_Supplementary_v3/Data_S1/DataS1/CIII/OG0002709.filter.fas.treefile.pdf]

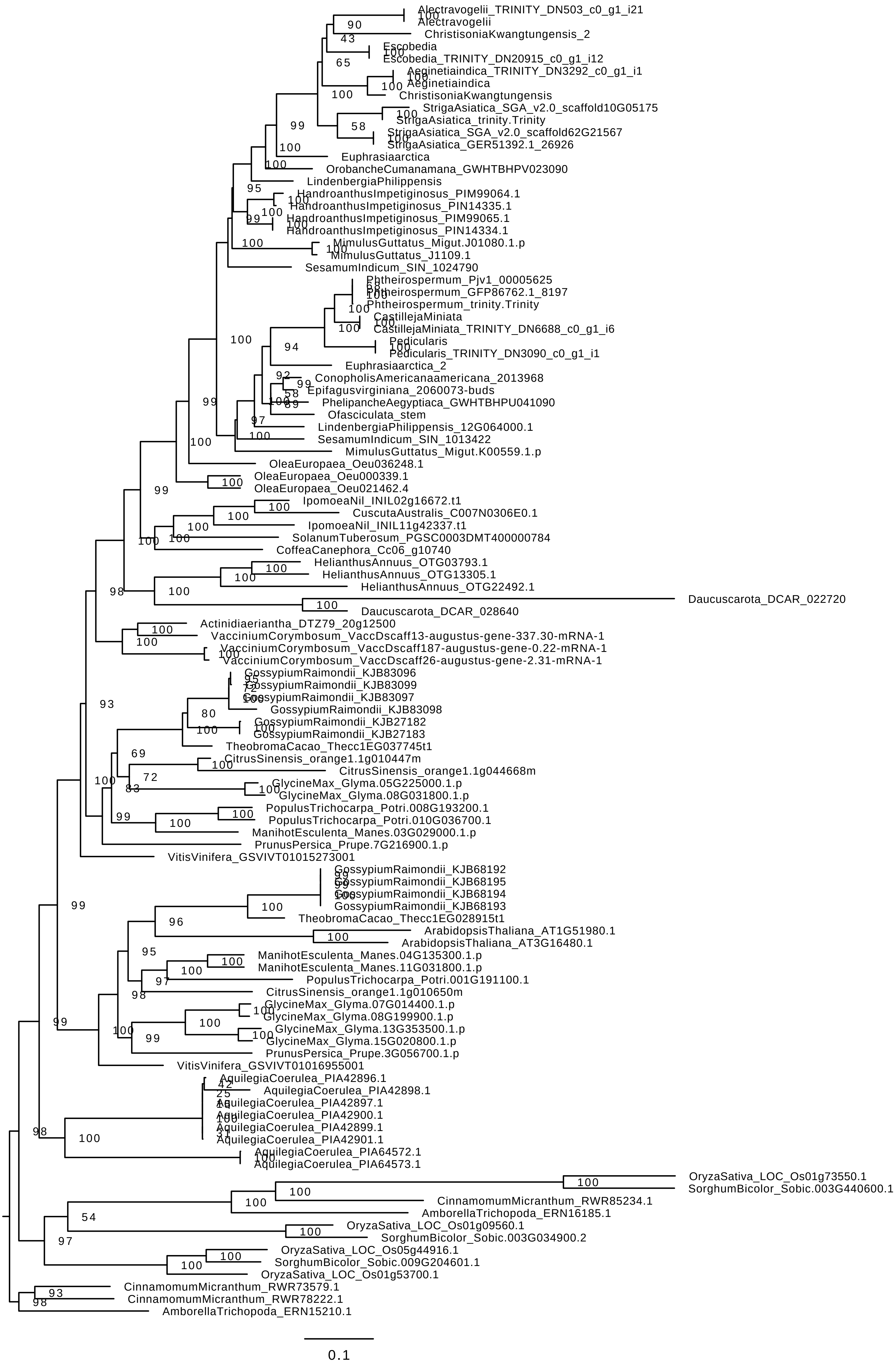
Alectravogelii_TRINITY_DN503_c0_g1_i21
Alectravogelii
ChristisoniaKwangtungensis_2
Escobedia
Escobedia_TRINITY_DN20915_c0_g1_i12
Aeginetiaindica_TRINITY_DN3292_c0_g1_i1
Aeginetiaindica
ChristisoniaKwangtungensis
StrigaAsiatica_SGA_v2.0_scaffold10G05175
StrigaAsiatica_trinity.Trinity
StrigaAsiatica_SGA_v2.0_scaffold62G21567
StrigaAsiatica_GER51392.1_26926
Euphrasiaarctica
OrobancheCumanamana_GWHTBHPV023090
LindenbergiaPhilippensis
HandroanthusImpetiginosus_PIM99064.1
HandroanthusImpetiginosus_PIN14335.1
HandroanthusImpetiginosus_PIM99065.1
HandroanthusImpetiginosus_PIN14334.1
MimulusGuttatus_Migut.J01080.1.p
MimulusGuttatus_J1109.1
SesamumIndicum_SIN_1024790
Phtheirospermum_Pjv1_00005625
Phtheirospermum_GFP86762.1_8197
Phtheirospermum_trinity.Trinity
CastillejaMiniata
CastillejaMiniata_TRINITY_DN6688_c0_g1_i6
Pedicularis
Pedicularis_TRINITY_DN3090_c0_g1_i1
Euphrasiaarctica_2
ConopholisAmericanaamericana_2013968
Epifagusvirginiana_2060073-buds
PhelipancheAegyptiaca_GWHTBHPU041090
Ofasciculata_stem
LindenbergiaPhilippensis_12G064000.1
SesamumIndicum_SIN_1013422
MimulusGuttatus_Migut.K00559.1.p
OleaEuropaea_Oeu036248.1
OleaEuropaea_Oeu000339.1
OleaEuropaea_Oeu021462.4
IpomoeaNil_INIL02g16672.t1
CuscutaAustralis_C007N0306E0.1
IpomoeaNil_INIL11g42337.t1
SolanumTuberosum_PGSC0003DMT400000784
CoffeaCanephora_Cc06_g10740
HelianthusAnnuus_OTG03793.1
HelianthusAnnuus_OTG13305.1
HelianthusAnnuus_OTG22492.1
Daucuscarota_DCAR_022720
Daucuscarota_DCAR_028640
Actinidiaeriantha_DTZ79_20g12500
VacciniumCorymbosum_VaccDscaff13-augustus-gene-337.30-mRNA-1
VacciniumCorymbosum_VaccDscaff187-augustus-gene-0.22-mRNA-1
VacciniumCorymbosum_VaccDscaff26-augustus-gene-2.31-mRNA-1
GossypiumRaimondii_KJB83096
GossypiumRaimondii_KJB83099
GossypiumRaimondii_KJB83097
GossypiumRaimondii_KJB83098
GossypiumRaimondii_KJB27182
GossypiumRaimondii_KJB27183
TheobromaCacao_Thecc1EG037745t1
CitrusSinensis_orange1.1g010447m
CitrusSinensis_orange1.1g044668m
GlycineMax_Glyma.05G225000.1.p
GlycineMax_Glyma.08G031800.1.p
PopulusTrichocarpa_Potri.008G193200.1
PopulusTrichocarpa_Potri.010G036700.1
ManihotEsculenta_Manes.03G029000.1.p
PrunusPersica_Prupe.7G216900.1.p
VitisVinifera_GSVIVT01015273001
GossypiumRaimondii_KJB68192
GossypiumRaimondii_KJB68195
GossypiumRaimondii_KJB68194
GossypiumRaimondii_KJB68193
TheobromaCacao_Thecc1EG028915t1
ArabidopsisThaliana_AT1G51980.1
ArabidopsisThaliana_AT3G16480.1
ManihotEsculenta_Manes.04G135300.1.p
ManihotEsculenta_Manes.11G031800.1.p
PopulusTrichocarpa_Potri.001G191100.1
CitrusSinensis_orange1.1g010650m
GlycineMax_Glyma.07G014400.1.p
GlycineMax_Glyma.08G199900.1.p
GlycineMax_Glyma.13G353500.1.p
GlycineMax_Glyma.15G020800.1.p
PrunusPersica_Prupe.3G056700.1.p
VitisVinifera_GSVIVT01016955001
AquilegiaCoerulea_PIA42896.1
AquilegiaCoerulea_PIA42898.1
AquilegiaCoerulea_PIA42897.1
AquilegiaCoerulea_PIA42900.1
AquilegiaCoerulea_PIA42899.1
AquilegiaCoerulea_PIA42901.1
AquilegiaCoerulea_PIA64572.1
AquilegiaCoerulea_PIA64573.1
OryzaSativa_LOC_Os01g73550.1
SorghumBicolor_Sobic.003G440600.1
CinnamomumMicranthum_RWR85234.1
AmborellaTrichopoda_ERN16185.1
OryzaSativa_LOC_Os01g09560.1
SorghumBicolor_Sobic.003G034900.2
OryzaSativa_LOC_Os05g44916.1
SorghumBicolor_Sobic.009G204601.1
OryzaSativa_LOC_Os01g53700.1
CinnamomumMicranthum_RWR73579.1
CinnamomumMicranthum_RWR78222.1
AmborellaTrichopoda_ERN15210.1
0.1
