# Supplementary material for: Altered Mitochondrial Respiration Is Associated With Loss of Nuclear‐Encoded OXPHOS Genes in Parasitic Broomrapes
Source: Ecol Evol. 2025 Jul 6;15(7):e71737. doi: 10.1002/ece3.71737 (PMC12230201; doi:10.1002/ece3.71737)
Supplement: Supplementary file 1 — Appendix S1. [file ECE3-15-e71737-s003.tgz › Orobanchaceae_O2K_Supplementary_v3/Data_S1/DataS1/CIII/OG0003410.filter.fas.treefile.pdf]

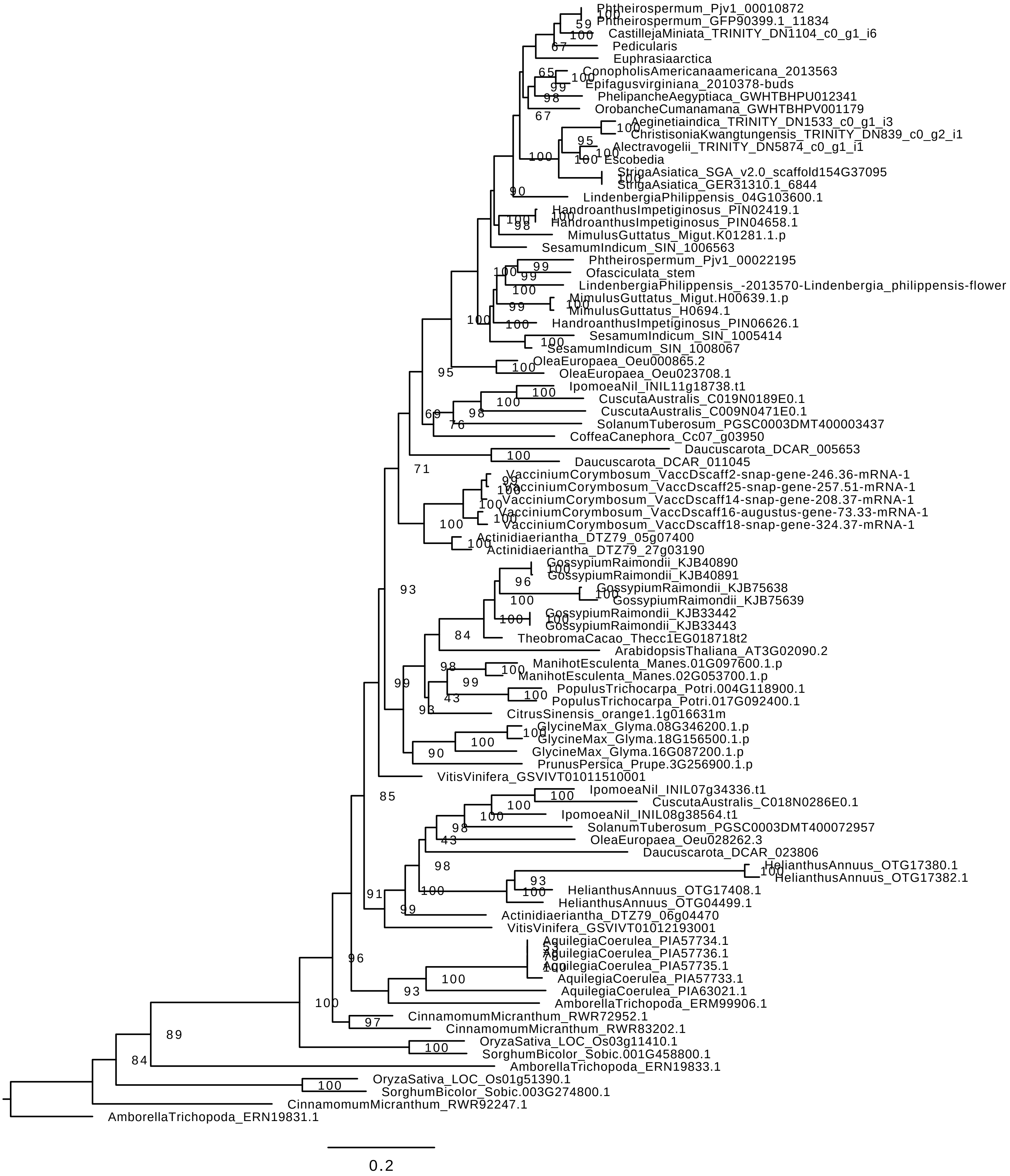

Phtheirospermum_Pjv1_00010872
Phtheirospermum_GFP90399.1_11834
CastillejaMiniata_TRINITY_DN1104_c0_g1_i6
Pedicularis
Euphrasiaarctica
ConopholisAmericanaamericana_2013563
Epifagusvirginiana_2010378-buds
PhelipancheAegyptiaca_GWHTBHPU012341
OrobancheCumanamana_GWHTBHPV001179
Aeginetiaindica_TRINITY_DN1533_c0_g1_i3
ChristisoniaKwangtungensis_TRINITY_DN839_c0_g2_i1
Alectravogelii_TRINITY_DN5874_c0_g1_i1
Escobedia
StrigaAsiatica_SGA_v2.0_scaffold154G37095
StrigaAsiatica_GER31310.1_6844
LindenbergiaPhilippensis_04G103600.1
HandroanthusImpetiginosus_PIN02419.1
HandroanthusImpetiginosus_PIN04658.1
MimulusGuttatus_Migut.K01281.1.p
SesamumIndicum_SIN_1006563
Phtheirospermum_Pjv1_00022195
Ofasciculata_stem
LindenbergiaPhilippensis_-2013570-Lindenbergia_philippensis-flower
MimulusGuttatus_Migut.H00639.1.p
MimulusGuttatus_H0694.1
HandroanthusImpetiginosus_PIN06626.1
SesamumIndicum_SIN_1005414
SesamumIndicum_SIN_1008067
OleaEuropaea_Oeu000865.2
OleaEuropaea_Oeu023708.1
IpomoeaNil_INIL11g18738.t1
CuscutaAustralis_C019N0189E0.1
CuscutaAustralis_C009N0471E0.1
SolanumTuberosum_PGSC0003DMT400003437
CoffeaCanephora_Cc07_g03950
Daucuscarota_DCAR_005653
Daucuscarota_DCAR_011045
VacciniumCorymbosum_VaccDscaff2-snap-gene-246.36-mRNA-1
VacciniumCorymbosum_VaccDscaff25-snap-gene-257.51-mRNA-1
VacciniumCorymbosum_VaccDscaff14-snap-gene-208.37-mRNA-1
VacciniumCorymbosum_VaccDscaff16-augustus-gene-73.33-mRNA-1
VacciniumCorymbosum_VaccDscaff18-snap-gene-324.37-mRNA-1
Actinidiaerianthа_DTZ79_05g07400
Actinidiaerianthа_DTZ79_27g03190
GossypiumRaimondii_KJB40890
GossypiumRaimondii_KJB40891
GossypiumRaimondii_KJB75638
GossypiumRaimondii_KJB75639
GossypiumRaimondii_KJB33442
GossypiumRaimondii_KJB33443
TheobromaCacao_Thecc1EG018718t2
ArabidopsisThaliana_AT3G02090.2
ManihotEsculenta_Manes.01G097600.1.p
ManihotEsculenta_Manes.02G053700.1.p
PopulusTrichocarpa_Potri.004G118900.1
PopulusTrichocarpa_Potri.017G092400.1
CitrusSinensis_orange1.1g016631m
GlycineMax_Glyma.08G346200.1.p
GlycineMax_Glyma.18G156500.1.p
GlycineMax_Glyma.16G087200.1.p
PrunusPersica_Prupe.3G256900.1.p
VitisVinifera_GSVIVT01011510001
IpomoeaNil_INIL07g34336.t1
CuscutaAustralis_C018N0286E0.1
IpomoeaNil_INIL08g38564.t1
SolanumTuberosum_PGSC0003DMT400072957
OleaEuropaea_Oeu028262.3
Daucuscarota_DCAR_023806
HelianthusAnnuus_OTG17380.1
HelianthusAnnuus_OTG17382.1
HelianthusAnnuus_OTG17408.1
HelianthusAnnuus_OTG04499.1
Actinidiaerianthа_DTZ79_06g04470
VitisVinifera_GSVIVT01012193001
AquilegiaCoerulea_PIA57734.1
AquilegiaCoerulea_PIA57736.1
AquilegiaCoerulea_PIA57735.1
AquilegiaCoerulea_PIA57733.1
AquilegiaCoerulea_PIA63021.1
AmborellaTrichopoda_ERM99906.1
CinnamomumMicranthum_RWR72952.1
CinnamomumMicranthum_RWR83202.1
OryzaSativa_LOC_Os03g11410.1
SorghumBicolor_Sobic.001G458800.1
AmborellaTrichopoda_ERN19833.1
OryzaSativa_LOC_Os01g51390.1
SorghumBicolor_Sobic.003G274800.1
CinnamomumMicranthum_RWR92247.1
AmborellaTrichopoda_ERN19831.1
0.2
