# Supplementary material for: Altered Mitochondrial Respiration Is Associated With Loss of Nuclear‐Encoded OXPHOS Genes in Parasitic Broomrapes
Source: Ecol Evol. 2025 Jul 6;15(7):e71737. doi: 10.1002/ece3.71737 (PMC12230201; doi:10.1002/ece3.71737)
Supplement: Supplementary file 1 — Appendix S1. [file ECE3-15-e71737-s003.tgz › Orobanchaceae_O2K_Supplementary_v3/Data_S1/DataS1/CIII/OG0004776.filter.fas.treefile.pdf]

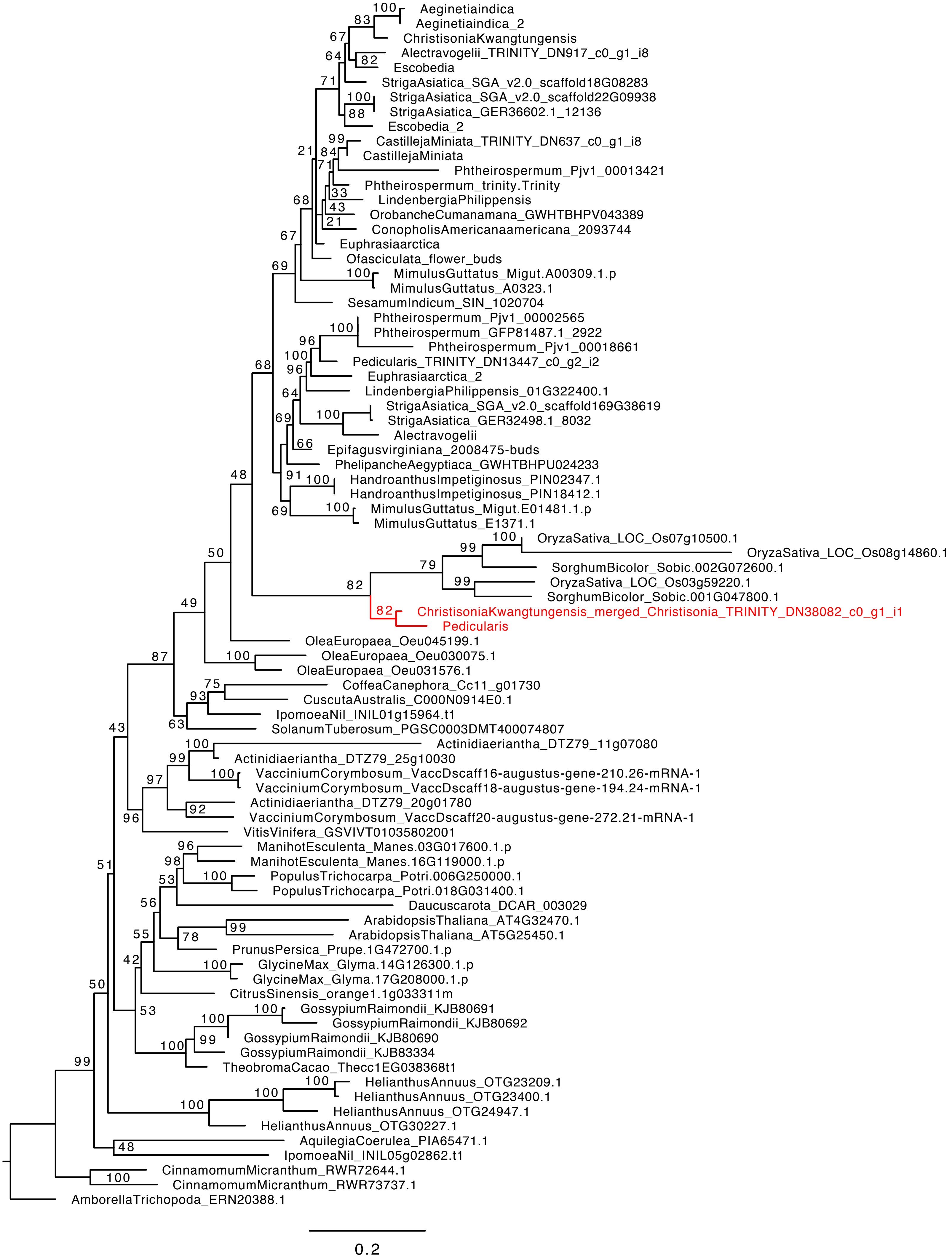

Aeginetiaindica
Aeginetiaindica_2
ChristisoniaKwangtungensis
Alectravogelii_TRINITY_DN917_c0_g1_i8
Escobedia
StrigaAsiatica_SGA_v2.0_scaffold18G08283
StrigaAsiatica_SGA_v2.0_scaffold22G09938
StrigaAsiatica_GER36602.1_12136
Escobedia_2
CastillejaMiniata_TRINITY_DN637_c0_g1_i8
CastillejaMiniata
Phtheirospermum_Pjv1_00013421
Phtheirospermum_trinity.Trinity
LindenbergiaPhilippensis
OrobancheCumanamana_GWHTBHPV043389
ConopholisAmericanaamericana_2093744
Euphrasiaarctica
Ofasciculata_flower_buds
MimulusGuttatus_Migut.A00309.1.p
MimulusGuttatus_A0323.1
SesamumIndicum_SIN_1020704
Phtheirospermum_Pjv1_00002565
Phtheirospermum_GFP81487.1_2922
Phtheirospermum_Pjv1_00018661
Pedicularis_TRINITY_DN13447_c0_g2_i2
Euphrasiaarctica_2
LindenbergiaPhilippensis_01G322400.1
StrigaAsiatica_SGA_v2.0_scaffold169G38619
StrigaAsiatica_GER32498.1_8032
Alectravogelii
Epifagusvirginiana_2008475-buds
PhelipancheAegyptiaca_GWHTBHPU024233
HandroanthusImpetiginosus_PIN02347.1
HandroanthusImpetiginosus_PIN18412.1
MimulusGuttatus_Migut.E01481.1.p
MimulusGuttatus_E1371.1
OryzaSativa_LOC_Os07g10500.1
OryzaSativa_LOC_Os08g14860.1
SorghumBicolor_Sobic.002G072600.1
OryzaSativa_LOC_Os03g59220.1
SorghumBicolor_Sobic.001G047800.1
ChristisoniaKwangtungensis_merged_Christisonia_TRINITY_DN38082_c0_g1_i1
Pedicularis
OleaEuropaea_Oeu045199.1
OleaEuropaea_Oeu030075.1
OleaEuropaea_Oeu031576.1
CoffeaCanephora_Cc11_g01730
CuscutaAustralis_C000N0914E0.1
IpomoeaNil_INIL01g15964.t1
SolanumTuberosum_PGSC0003DMT400074807
Actinidiaeriantha_DTZ79_11g07080
Actinidiaeriantha_DTZ79_25g10030
VacciniumCorymbosum_VaccDscaff16-augustus-gene-210.26-mRNA-1
VacciniumCorymbosum_VaccDscaff18-augustus-gene-194.24-mRNA-1
Actinidiaeriantha_DTZ79_20g01780
VacciniumCorymbosum_VaccDscaff20-augustus-gene-272.21-mRNA-1
VitisVinifera_GSVIVT01035802001
ManihotEsculenta_Manes.03G017600.1.p
ManihotEsculenta_Manes.16G119000.1.p
PopulusTrichocarpa_Potri.006G250000.1
PopulusTrichocarpa_Potri.018G031400.1
Daucuscarota_DCAR_003029
ArabidopsisThaliana_AT4G32470.1
ArabidopsisThaliana_AT5G25450.1
PrunusPersica_Prupe.1G472700.1.p
GlycineMax_Glyma.14G126300.1.p
GlycineMax_Glyma.17G208000.1.p
CitrusSinensis_orange1.1g033311m
GossypiumRaimondii_KJB80691
GossypiumRaimondii_KJB80692
GossypiumRaimondii_KJB80690
GossypiumRaimondii_KJB83334
TheobromaCacao_Thecc1EG038368t1
HelianthusAnnuus_OTG23209.1
HelianthusAnnuus_OTG23400.1
HelianthusAnnuus_OTG24947.1
HelianthusAnnuus_OTG30227.1
AquilegiaCoerulea_PIA65471.1
IpomoeaNil_INIL05g02862.t1
CinnamomumMicranthum_RWR72644.1
CinnamomumMicranthum_RWR73737.1
AmborellaTrichopoda_ERN20388.1
0.2
